# Supplementary material for: Pattern of SARS-CoV-2 variant B.1.1.519 emergence in Alaska
Source: Sci Rep. 2022 Nov 30;12:20662. doi: 10.1038/s41598-022-25373-1 (PMC9712339; doi:10.1038/s41598-022-25373-1)
Supplement: Supplementary file 1 — Supplementary Information. [file 41598_2022_25373_MOESM1_ESM.pdf]

Pattern of SARS-CoV-2 variant B.1.1.519 emergence in Alaska

Tracie J. Haan[1], Lisa K. Smith[2], Stephanie DeRonde[2], Elva House[2], Jacob Zidek[2], Diana Puhak[2], Matthew Redlinger[3], Jayme Parker[2], Brian M. Barnes[1], Jason L. Burkhead[3], Cindy Knall[4], Eric Bortz[3,4,*], Jack Chen[1,2,5,*], and Devin M. Drown[1,5,*]

[1] Institute of Arctic Biology, University of Alaska Fairbanks, Fairbanks, Alaska, USA
[2] Alaska Division of Public Health, State of Alaska, Fairbanks, Alaska, USA
[3] Department of Biological Sciences, University of Alaska Anchorage, Anchorage, Alaska, USA
[4] WWAMI School of Medical Education, University of Alaska Anchorage, Anchorage, Alaska, USA
[5] Department of Biology and Wildlife, University of Alaska Fairbanks, Fairbanks, Alaska, USA

* Corresponding authors: Devin M. Drown, dmdrown@alaska.edu; Eric Bortz ebortz@alaska.edu; Jack Chen, jchen15@alaska.edu

Supplemental Figures.

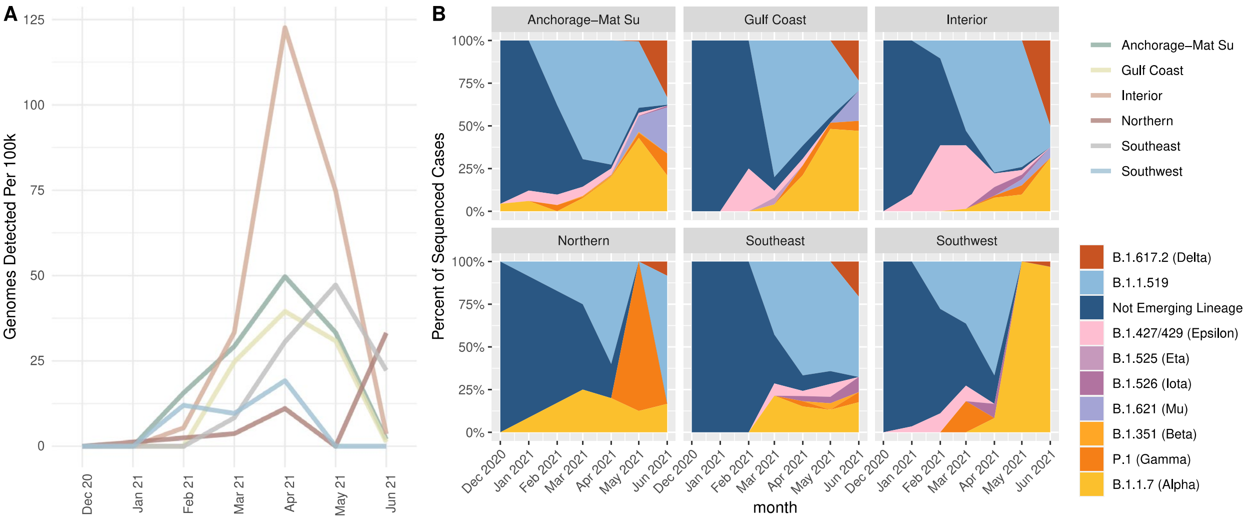

Figure S1. (A) Number of detected B.1.1.519 genomes per 100k people by Alaska economic region over time. Lines are colored by Alaska economic region. (B) Estimated prevalence of emerging lineages by Alaska economic region from December 2020 through June 2021.

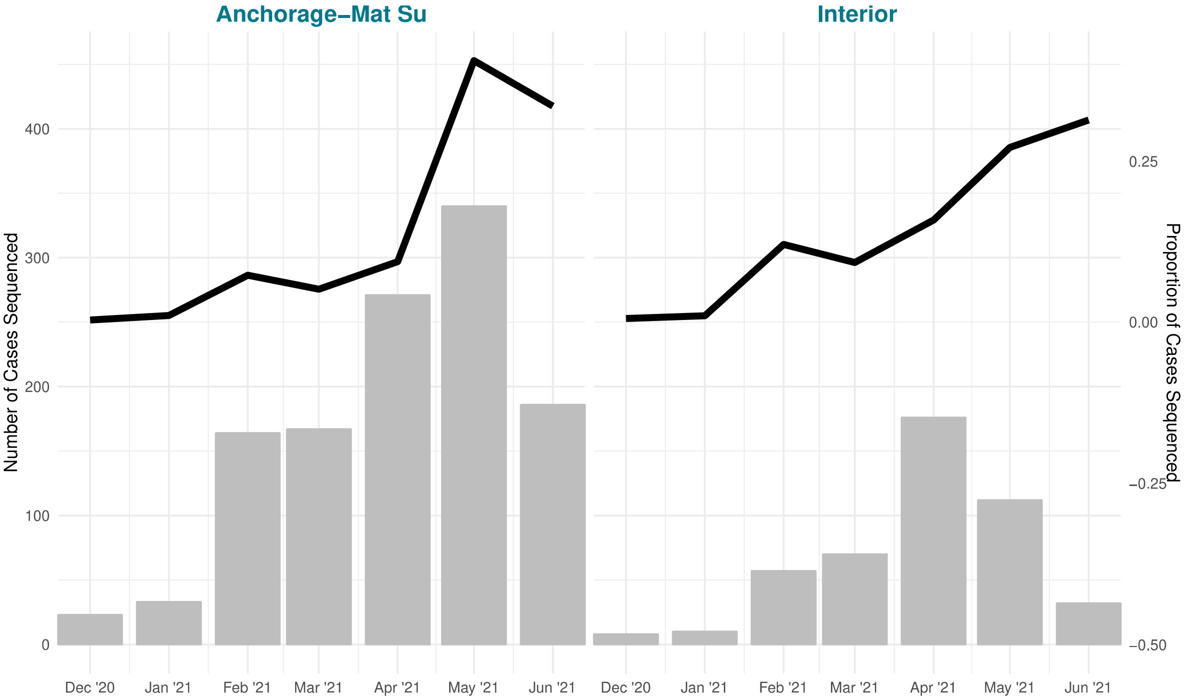

Figure S2. Number of cases sequenced (bars) and the proportion of total cases sequenced in the Anchorage-Mat Su and Interior economic regions of Alaska.

We gratefully acknowledge the authors, originating and submitting laboratories of the sequences from GISAIDÕs EpiFlu. All submitters of data may be contacted directly via www.gisaid.org

Virus Name	Accession ID	Collection Date	Originating Lab	Submitting Lab	Authors
hCoV-19/USA/AK-CDC-ASC210010031/2021	EPI_ISL_1649624	3/18/2021	Aegis Sciences Corporation	Centers for Disease Control and Prevention Division of Viral Diseases, Pathogen Discovery	Dakota Howard, Dhwani Batra, Peter W. Cook, Kara Moser, Adrian Paskey, Jason Caravas, Benjamin Rambo-Martin, Shatavia Morrison, Christopher Gulvick, Scott Sammons, Yvette Unoarumhi, Darlene Wagner, Matthew Schmerer, Cyndi Clark, Patrick Campbell, Rob Case, Vikramsinha Ghorpade, Holly Houdeshell, Ola Kvalvaag, Dillon Nall, Ethan Sanders, Alec Vest, Shaun Westlund, Matthew Hardison, Clinton R. Paden, Duncan MacCannell
hCoV-19/USA/AK-PHL7461/2021	EPI_ISL_1652201	3/5/2021	Alaska State Virology Laboratory	Alaska State Virology Laboratory	Stephanie DeRonde, Elva House, Lisa Smith, Ph.D., Jack Chen, Ph.D.
hCoV-19/USA/AK-PHL7462/2021	EPI_ISL_1652202	3/5/2021	Alaska State Virology Laboratory	Alaska State Virology Laboratory	Stephanie DeRonde, Elva House, Lisa Smith, Ph.D., Jack Chen, Ph.D.
hCoV-19/USA/AK-PHL7463/2021	EPI_ISL_1652203	3/5/2021	Alaska State Virology Laboratory	Alaska State Virology Laboratory	Stephanie DeRonde, Elva House, Lisa Smith, Ph.D., Jack Chen, Ph.D.
hCoV-19/USA/AK-PHL7465/2021	EPI_ISL_1652204	3/5/2021	Alaska State Virology Laboratory	Alaska State Virology Laboratory	Stephanie DeRonde, Elva House, Lisa Smith, Ph.D., Jack Chen, Ph.D.
hCoV-19/USA/AK-PHL7466/2021	EPI_ISL_1652205	3/5/2021	Alaska State Virology Laboratory	Alaska State Virology Laboratory	Stephanie DeRonde, Elva House, Lisa Smith, Ph.D., Jack Chen, Ph.D.
hCoV-19/USA/AK-PHL7467/2021	EPI_ISL_1652206	3/5/2021	Alaska State Virology Laboratory	Alaska State Virology Laboratory	Stephanie DeRonde, Elva House, Lisa Smith, Ph.D., Jack Chen, Ph.D.
hCoV-19/USA/AK-PHL7470/2021	EPI_ISL_1652207	3/6/2021	Alaska State Virology Laboratory	Alaska State Virology Laboratory	Stephanie DeRonde, Elva House, Lisa Smith, Ph.D., Jack Chen, Ph.D.
hCoV-19/USA/AK-PHL7471/2021	EPI_ISL_1652208	3/6/2021	Alaska State Virology Laboratory	Alaska State Virology Laboratory	Stephanie DeRonde, Elva House, Lisa Smith, Ph.D., Jack Chen, Ph.D.
hCoV-19/USA/AK-PHL7472/2021	EPI_ISL_1652209	3/6/2021	Alaska State Virology Laboratory	Alaska State Virology Laboratory	Stephanie DeRonde, Elva House, Lisa Smith, Ph.D., Jack Chen, Ph.D.
hCoV-19/USA/AK-PHL7474/2021	EPI_ISL_1652211	3/6/2021	Alaska State Virology Laboratory	Alaska State Virology Laboratory	Stephanie DeRonde, Elva House, Lisa Smith, Ph.D., Jack Chen, Ph.D.
hCoV-19/USA/AK-PHL7475/2021	EPI_ISL_1652212	3/6/2021	Alaska State Virology Laboratory	Alaska State Virology Laboratory	Stephanie DeRonde, Elva House, Lisa Smith, Ph.D., Jack Chen, Ph.D.
hCoV-19/USA/AK-PHL7476/2021	EPI_ISL_1652213	3/6/2021	Alaska State Virology Laboratory	Alaska State Virology Laboratory	Stephanie DeRonde, Elva House, Lisa Smith, Ph.D., Jack Chen, Ph.D.
hCoV-19/USA/AK-PHL7478/2021	EPI_ISL_1652214	3/6/2021	Alaska State Virology Laboratory	Alaska State Virology Laboratory	Stephanie DeRonde, Elva House, Lisa Smith, Ph.D., Jack Chen, Ph.D.
hCoV-19/USA/AK-PHL7479/2021	EPI_ISL_1652215	3/6/2021	Alaska State Virology Laboratory	Alaska State Virology Laboratory	Stephanie DeRonde, Elva House, Lisa Smith, Ph.D., Jack Chen, Ph.D.
hCoV-19/USA/AK-PHL7480/2021	EPI_ISL_1652216	3/6/2021	Alaska State Virology Laboratory	Alaska State Virology Laboratory	Stephanie DeRonde, Elva House, Lisa Smith, Ph.D., Jack Chen, Ph.D.
hCoV-19/USA/AK-PHL7481/2021	EPI_ISL_1652217	3/6/2021	Alaska State Virology Laboratory	Alaska State Virology Laboratory	Stephanie DeRonde, Elva House, Lisa Smith, Ph.D., Jack Chen, Ph.D.
hCoV-19/USA/AK-PHL7485/2021	EPI_ISL_1652220	3/6/2021	Alaska State Virology Laboratory	Alaska State Virology Laboratory	Stephanie DeRonde, Elva House, Lisa Smith, Ph.D., Jack Chen, Ph.D.
hCoV-19/USA/AK-PHL7486/2021	EPI_ISL_1652221	3/5/2021	Alaska State Virology Laboratory	Alaska State Virology Laboratory	Stephanie DeRonde, Elva House, Lisa Smith, Ph.D., Jack Chen, Ph.D.
hCoV-19/USA/AK-PHL7487/2021	EPI_ISL_1652222	3/6/2021	Alaska State Virology Laboratory	Alaska State Virology Laboratory	Stephanie DeRonde, Elva House, Lisa Smith, Ph.D., Jack Chen, Ph.D.
hCoV-19/USA/AK-PHL7489/2021	EPI_ISL_1652224	3/6/2021	Alaska State Virology Laboratory	Alaska State Virology Laboratory	Stephanie DeRonde, Elva House, Lisa Smith, Ph.D., Jack Chen, Ph.D.
hCoV-19/USA/AK-PHL7491/2021	EPI_ISL_1652225	3/5/2021	Alaska State Virology Laboratory	Alaska State Virology Laboratory	Stephanie DeRonde, Elva House, Lisa Smith, Ph.D., Jack Chen, Ph.D.
hCoV-19/USA/AK-PHL7493/2021	EPI_ISL_1652227	3/6/2021	Alaska State Virology Laboratory	Alaska State Virology Laboratory	Stephanie DeRonde, Elva House, Lisa Smith, Ph.D., Jack Chen, Ph.D.
hCoV-19/USA/AK-PHL7495/2021	EPI_ISL_1652228	3/6/2021	Alaska State Virology Laboratory	Alaska State Virology Laboratory	Stephanie DeRonde, Elva House, Lisa Smith, Ph.D., Jack Chen, Ph.D.
hCoV-19/USA/AK-PHL7497/2021	EPI_ISL_1652229	3/6/2021	Alaska State Virology Laboratory	Alaska State Virology Laboratory	Stephanie DeRonde, Elva House, Lisa Smith, Ph.D., Jack Chen, Ph.D.
hCoV-19/USA/AK-PHL7498/2021	EPI_ISL_1652230	3/6/2021	Alaska State Virology Laboratory	Alaska State Virology Laboratory	Stephanie DeRonde, Elva House, Lisa Smith, Ph.D., Jack Chen, Ph.D.
hCoV-19/USA/AK-PHL7503/2021	EPI_ISL_1652234	3/6/2021	Alaska State Virology Laboratory	Alaska State Virology Laboratory	Stephanie DeRonde, Elva House, Lisa Smith, Ph.D., Jack Chen, Ph.D.
hCoV-19/USA/AK-PHL7504/2021	EPI_ISL_1652235	3/6/2021	Alaska State Virology Laboratory	Alaska State Virology Laboratory	Stephanie DeRonde, Elva House, Lisa Smith, Ph.D., Jack Chen, Ph.D.
hCoV-19/USA/AK-PHL7506/2021	EPI_ISL_1652236	3/4/2021	Alaska State Virology Laboratory	Alaska State Virology Laboratory	Stephanie DeRonde, Elva House, Lisa Smith, Ph.D., Jack Chen, Ph.D.
hCoV-19/USA/AK-PHL7970/2021	EPI_ISL_1653895	4/2/2021	Alaska State Virology Laboratory	Alaska State Virology Laboratory	Stephanie DeRonde, Elva House, Lisa Smith, Ph.D., Jack Chen, Ph.D.
hCoV-19/USA/AK-PHL7971/2021	EPI_ISL_1653896	4/4/2021	Alaska State Virology Laboratory	Alaska State Virology Laboratory	Stephanie DeRonde, Elva House, Lisa Smith, Ph.D., Jack Chen, Ph.D.
hCoV-19/USA/AK-PHL7973/2021	EPI_ISL_1653897	4/4/2021	Alaska State Virology Laboratory	Alaska State Virology Laboratory	Stephanie DeRonde, Elva House, Lisa Smith, Ph.D., Jack Chen, Ph.D.
hCoV-19/USA/AK-PHL7975/2021	EPI_ISL_1653898	4/5/2021	Alaska State Virology Laboratory	Alaska State Virology Laboratory	Stephanie DeRonde, Elva House, Lisa Smith, Ph.D., Jack Chen, Ph.D.
hCoV-19/USA/AK-PHL7992/2021	EPI_ISL_1653899	4/7/2021	Alaska State Virology Laboratory	Alaska State Virology Laboratory	Stephanie DeRonde, Elva House, Lisa Smith, Ph.D., Jack Chen, Ph.D.
hCoV-19/USA/AK-PHL7993/2021	EPI_ISL_1653900	4/7/2021	Alaska State Virology Laboratory	Alaska State Virology Laboratory	Stephanie DeRonde, Elva House, Lisa Smith, Ph.D., Jack Chen, Ph.D.
hCoV-19/USA/AK-PHL7996/2021	EPI_ISL_1653903	4/7/2021	Alaska State Virology Laboratory	Alaska State Virology Laboratory	Stephanie DeRonde, Elva House, Lisa Smith, Ph.D., Jack Chen, Ph.D.
hCoV-19/USA/AK-PHL7998/2021	EPI_ISL_1653904	4/7/2021	Alaska State Virology Laboratory	Alaska State Virology Laboratory	Stephanie DeRonde, Elva House, Lisa Smith, Ph.D., Jack Chen, Ph.D.
hCoV-19/USA/AK-PHL8000/2021	EPI_ISL_1653906	4/7/2021	Alaska State Virology Laboratory	Alaska State Virology Laboratory	Stephanie DeRonde, Elva House, Lisa Smith, Ph.D., Jack Chen, Ph.D.
hCoV-19/USA/AK-PHL8001/2021	EPI_ISL_1653907	4/7/2021	Alaska State Virology Laboratory	Alaska State Virology Laboratory	Stephanie DeRonde, Elva House, Lisa Smith, Ph.D., Jack Chen, Ph.D.
hCoV-19/USA/AK-PHL8002/2021	EPI_ISL_1653908	4/7/2021	Alaska State Virology Laboratory	Alaska State Virology Laboratory	Stephanie DeRonde, Elva House, Lisa Smith, Ph.D., Jack Chen, Ph.D.
hCoV-19/USA/AK-PHL8003/2021	EPI_ISL_1653909	4/7/2021	Alaska State Virology Laboratory	Alaska State Virology Laboratory	Stephanie DeRonde, Elva House, Lisa Smith, Ph.D., Jack Chen, Ph.D.
hCoV-19/USA/AK-PHL8004/2021	EPI_ISL_1653910	4/7/2021	Alaska State Virology Laboratory	Alaska State Virology Laboratory	Stephanie DeRonde, Elva House, Lisa Smith, Ph.D., Jack Chen, Ph.D.
hCoV-19/USA/AK-PHL8005/2021	EPI_ISL_1653911	4/7/2021	Alaska State Virology Laboratory	Alaska State Virology Laboratory	Stephanie DeRonde, Elva House, Lisa Smith, Ph.D., Jack Chen, Ph.D.
hCoV-19/USA/AK-PHL8006/2021	EPI_ISL_1653912	4/6/2021	Alaska State Virology Laboratory	Alaska State Virology Laboratory	Stephanie DeRonde, Elva House, Lisa Smith, Ph.D., Jack Chen, Ph.D.
hCoV-19/USA/AK-PHL8007/2021	EPI_ISL_1653913	4/5/2021	Alaska State Virology Laboratory	Alaska State Virology Laboratory	Stephanie DeRonde, Elva House, Lisa Smith, Ph.D., Jack Chen, Ph.D.

hCoV-19/USA/AK-PHL8008/2021	EPI_ISL_1653914	4/6/2021	Alaska State Virology Laboratory	Alaska State Virology Laboratory	Stephanie DeRonde, Elva House, Lisa Smith, Ph.D., Jack Chen, Ph.D.
hCoV-19/USA/AK-PHL8009/2021	EPI_ISL_1653915	4/6/2021	Alaska State Virology Laboratory	Alaska State Virology Laboratory	Stephanie DeRonde, Elva House, Lisa Smith, Ph.D., Jack Chen, Ph.D.
hCoV-19/USA/AK-PHL8010/2021	EPI_ISL_1653916	4/6/2021	Alaska State Virology Laboratory	Alaska State Virology Laboratory	Stephanie DeRonde, Elva House, Lisa Smith, Ph.D., Jack Chen, Ph.D.
hCoV-19/USA/AK-PHL8011/2021	EPI_ISL_1653917	4/6/2021	Alaska State Virology Laboratory	Alaska State Virology Laboratory	Stephanie DeRonde, Elva House, Lisa Smith, Ph.D., Jack Chen, Ph.D.
hCoV-19/USA/AK-PHL8012/2021	EPI_ISL_1653918	4/6/2021	Alaska State Virology Laboratory	Alaska State Virology Laboratory	Stephanie DeRonde, Elva House, Lisa Smith, Ph.D., Jack Chen, Ph.D.
hCoV-19/USA/AK-PHL8013/2021	EPI_ISL_1653919	4/6/2021	Alaska State Virology Laboratory	Alaska State Virology Laboratory	Stephanie DeRonde, Elva House, Lisa Smith, Ph.D., Jack Chen, Ph.D.
hCoV-19/USA/AK-PHL7261/2021	EPI_ISL_1182706	2/18/2021	Alaska State Virology Laboratory	Alaska State Virology Laboratory	Stephanie DeRonde, Lisa Smith, Ph.D., Jack Chen, Ph.D.
hCoV-19/USA/AK-PHL7250/2021	EPI_ISL_1182711	2/17/2021	Alaska State Virology Laboratory	Alaska State Virology Laboratory	Stephanie DeRonde, Lisa Smith, Ph.D., Jack Chen, Ph.D.
hCoV-19/USA/AK-PHL7256/2021	EPI_ISL_1182712	2/17/2021	Alaska State Virology Laboratory	Alaska State Virology Laboratory	Stephanie DeRonde, Lisa Smith, Ph.D., Jack Chen, Ph.D.
hCoV-19/USA/AK-PHL7252/2021	EPI_ISL_1182731	2/16/2021	Alaska State Virology Laboratory	Alaska State Virology Laboratory	Stephanie DeRonde, Lisa Smith, Ph.D., Jack Chen, Ph.D.
hCoV-19/USA/AK-PHL7287/2021	EPI_ISL_1182751	2/21/2021	Alaska State Virology Laboratory	Alaska State Virology Laboratory	Stephanie DeRonde, Lisa Smith, Ph.D., Jack Chen, Ph.D.
hCoV-19/USA/AK-PHL7296/2021	EPI_ISL_1182754	2/21/2021	Alaska State Virology Laboratory	Alaska State Virology Laboratory	Stephanie DeRonde, Lisa Smith, Ph.D., Jack Chen, Ph.D.
hCoV-19/USA/AK-PHL7146/2021	EPI_ISL_1182786	2/15/2021	Alaska State Virology Laboratory	Alaska State Virology Laboratory	Stephanie DeRonde, Lisa Smith, Ph.D., Jack Chen, Ph.D.
hCoV-19/USA/AK-PHL7148/2021	EPI_ISL_1182788	2/15/2021	Alaska State Virology Laboratory	Alaska State Virology Laboratory	Stephanie DeRonde, Lisa Smith, Ph.D., Jack Chen, Ph.D.
hCoV-19/USA/AK-PHL7169/2021	EPI_ISL_1182806	2/15/2021	Alaska State Virology Laboratory	Alaska State Virology Laboratory	Stephanie DeRonde, Lisa Smith, Ph.D., Jack Chen, Ph.D.
hCoV-19/USA/AK-CDC-ASC210039224/2021	EPI_ISL_1668068	3/31/2021	Aegis Sciences Corporation	Centers for Disease Control and Prevention Division of Viral Diseases, Pathogen Discovery	Dakota Howard, Dhwani Batra, Peter W. Cook, Kara Moser, Adrian Paskey, Jason Caravas, Benjamin Rambo-Martin, Shatavia Morrison, Christopher Gulvick, Scott Sammons, Yvette Unoarumhi, Darlene Wagner, Matthew Schmerer, Cyndi Clark, Patrick Campbell, Rob Case, Vikramsinha Ghorpade, Holly Houdeshell, Ola Kvalvaag, Dillon Nall, Ethan Sanders, Alec Vest, Shaun Westlund, Matthew Hardison, Clinton R. Paden, Duncan MacCannell
hCoV-19/USA/AK-CDC-ASC210039226/2021	EPI_ISL_1668069	3/31/2021	Aegis Sciences Corporation	Centers for Disease Control and Prevention Division of Viral Diseases, Pathogen Discovery	Dakota Howard, Dhwani Batra, Peter W. Cook, Kara Moser, Adrian Paskey, Jason Caravas, Benjamin Rambo-Martin, Shatavia Morrison, Christopher Gulvick, Scott Sammons, Yvette Unoarumhi, Darlene Wagner, Matthew Schmerer, Cyndi Clark, Patrick Campbell, Rob Case, Vikramsinha Ghorpade, Holly Houdeshell, Ola Kvalvaag, Dillon Nall, Ethan Sanders, Alec Vest, Shaun Westlund, Matthew Hardison, Clinton R. Paden, Duncan MacCannell
hCoV-19/USA/AK-CDC-ASC210039229/2021	EPI_ISL_1668072	3/30/2021	Aegis Sciences Corporation	Centers for Disease Control and Prevention Division of Viral Diseases, Pathogen Discovery	Dakota Howard, Dhwani Batra, Peter W. Cook, Kara Moser, Adrian Paskey, Jason Caravas, Benjamin Rambo-Martin, Shatavia Morrison, Christopher Gulvick, Scott Sammons, Yvette Unoarumhi, Darlene Wagner, Matthew Schmerer, Cyndi Clark, Patrick Campbell, Rob Case, Vikramsinha Ghorpade, Holly Houdeshell, Ola Kvalvaag, Dillon Nall, Ethan Sanders, Alec Vest, Shaun Westlund, Matthew Hardison, Clinton R. Paden, Duncan MacCannell
hCoV-19/USA/AK-PHL7864/2021	EPI_ISL_1675099	4/2/2021	Alaska State Virology Laboratory	Alaska State Virology Laboratory	Stephanie DeRonde, Elva House, Lisa Smith, Ph.D., Jack Chen, Ph.D.
hCoV-19/USA/AK-PHL7865/2021	EPI_ISL_1675100	4/2/2021	Alaska State Virology Laboratory	Alaska State Virology Laboratory	Stephanie DeRonde, Elva House, Lisa Smith, Ph.D., Jack Chen, Ph.D.
hCoV-19/USA/AK-PHL7866/2021	EPI_ISL_1675101	3/31/2021	Alaska State Virology Laboratory	Alaska State Virology Laboratory	Stephanie DeRonde, Elva House, Lisa Smith, Ph.D., Jack Chen, Ph.D.
hCoV-19/USA/AK-PHL7868/2021	EPI_ISL_1675102	3/29/2021	Alaska State Virology Laboratory	Alaska State Virology Laboratory	Stephanie DeRonde, Elva House, Lisa Smith, Ph.D., Jack Chen, Ph.D.
hCoV-19/USA/AK-PHL7862/2021	EPI_ISL_1675097	4/2/2021	Alaska State Virology Laboratory	Alaska State Virology Laboratory	Stephanie DeRonde, Elva House, Lisa Smith, Ph.D., Jack Chen, Ph.D.
hCoV-19/USA/AK-PHL7863/2021	EPI_ISL_1675098	4/1/2021	Alaska State Virology Laboratory	Alaska State Virology Laboratory	Stephanie DeRonde, Elva House, Lisa Smith, Ph.D., Jack Chen, Ph.D.
hCoV-19/USA/AK-PHL7870/2021	EPI_ISL_1675103	3/31/2021	Alaska State Virology Laboratory	Alaska State Virology Laboratory	Stephanie DeRonde, Elva House, Lisa Smith, Ph.D., Jack Chen, Ph.D.
hCoV-19/USA/AK-PHL7872/2021	EPI_ISL_1675105	3/29/2021	Alaska State Virology Laboratory	Alaska State Virology Laboratory	Stephanie DeRonde, Elva House, Lisa Smith, Ph.D., Jack Chen, Ph.D.
hCoV-19/USA/AK-PHL7873/2021	EPI_ISL_1675106	3/30/2021	Alaska State Virology Laboratory	Alaska State Virology Laboratory	Stephanie DeRonde, Elva House, Lisa Smith, Ph.D., Jack Chen, Ph.D.
hCoV-19/USA/AK-PHL7874/2021	EPI_ISL_1675107	3/29/2021	Alaska State Virology Laboratory	Alaska State Virology Laboratory	Stephanie DeRonde, Elva House, Lisa Smith, Ph.D., Jack Chen, Ph.D.
hCoV-19/USA/AK-PHL7875/2021	EPI_ISL_1675108	3/28/2021	Alaska State Virology Laboratory	Alaska State Virology Laboratory	Stephanie DeRonde, Elva House, Lisa Smith, Ph.D., Jack Chen, Ph.D.
hCoV-19/USA/AK-PHL7877/2021	EPI_ISL_1675110	3/29/2021	Alaska State Virology Laboratory	Alaska State Virology Laboratory	Stephanie DeRonde, Elva House, Lisa Smith, Ph.D., Jack Chen, Ph.D.
hCoV-19/USA/AK-PHL7878/2021	EPI_ISL_1675111	3/29/2021	Alaska State Virology Laboratory	Alaska State Virology Laboratory	Stephanie DeRonde, Elva House, Lisa Smith, Ph.D., Jack Chen, Ph.D.
hCoV-19/USA/AK-PHL7880/2021	EPI_ISL_1675112	3/30/2021	Alaska State Virology Laboratory	Alaska State Virology Laboratory	Stephanie DeRonde, Elva House, Lisa Smith, Ph.D., Jack Chen, Ph.D.
hCoV-19/USA/AK-PHL7881/2021	EPI_ISL_1675113	3/29/2021	Alaska State Virology Laboratory	Alaska State Virology Laboratory	Stephanie DeRonde, Elva House, Lisa Smith, Ph.D., Jack Chen, Ph.D.
hCoV-19/USA/AK-PHL7882/2021	EPI_ISL_1675114	4/3/2021	Alaska State Virology Laboratory	Alaska State Virology Laboratory	Stephanie DeRonde, Elva House, Lisa Smith, Ph.D., Jack Chen, Ph.D.
hCoV-19/USA/AK-PHL7886/2021	EPI_ISL_1675116	3/31/2021	Alaska State Virology Laboratory	Alaska State Virology Laboratory	Stephanie DeRonde, Elva House, Lisa Smith, Ph.D., Jack Chen, Ph.D.
hCoV-19/USA/AK-PHL7888/2021	EPI_ISL_1675117	4/2/2021	Alaska State Virology Laboratory	Alaska State Virology Laboratory	Stephanie DeRonde, Elva House, Lisa Smith, Ph.D., Jack Chen, Ph.D.
hCoV-19/USA/AK-PHL7889/2021	EPI_ISL_1675118	4/2/2021	Alaska State Virology Laboratory	Alaska State Virology Laboratory	Stephanie DeRonde, Elva House, Lisa Smith, Ph.D., Jack Chen, Ph.D.

hCoV-19/USA/AK-PHL7890/2021	EPI_ISL_1675119	4/2/2021	Alaska State Virology Laboratory	Alaska State Virology Laboratory	Stephanie DeRonde, Elva House, Lisa Smith, Ph.D., Jack Chen, Ph.D.
hCoV-19/USA/AK-PHL7891/2021	EPI_ISL_1675120	4/2/2021	Alaska State Virology Laboratory	Alaska State Virology Laboratory	Stephanie DeRonde, Elva House, Lisa Smith, Ph.D., Jack Chen, Ph.D.
hCoV-19/USA/AK-PHL7892/2021	EPI_ISL_1675121	4/3/2021	Alaska State Virology Laboratory	Alaska State Virology Laboratory	Stephanie DeRonde, Elva House, Lisa Smith, Ph.D., Jack Chen, Ph.D.
hCoV-19/USA/AK-PHL7893/2021	EPI_ISL_1675122	4/3/2021	Alaska State Virology Laboratory	Alaska State Virology Laboratory	Stephanie DeRonde, Elva House, Lisa Smith, Ph.D., Jack Chen, Ph.D.
hCoV-19/USA/AK-PHL7894/2021	EPI_ISL_1675123	4/3/2021	Alaska State Virology Laboratory	Alaska State Virology Laboratory	Stephanie DeRonde, Elva House, Lisa Smith, Ph.D., Jack Chen, Ph.D.
hCoV-19/USA/AK-PHL7895/2021	EPI_ISL_1675124	3/31/2021	Alaska State Virology Laboratory	Alaska State Virology Laboratory	Stephanie DeRonde, Elva House, Lisa Smith, Ph.D., Jack Chen, Ph.D.
hCoV-19/USA/AK-PHL7896/2021	EPI_ISL_1675125	3/30/2021	Alaska State Virology Laboratory	Alaska State Virology Laboratory	Stephanie DeRonde, Elva House, Lisa Smith, Ph.D., Jack Chen, Ph.D.
hCoV-19/USA/AK-PHL7898/2021	EPI_ISL_1675126	3/29/2021	Alaska State Virology Laboratory	Alaska State Virology Laboratory	Stephanie DeRonde, Elva House, Lisa Smith, Ph.D., Jack Chen, Ph.D.
hCoV-19/USA/AK-PHL7900/2021	EPI_ISL_1675128	3/26/2021	Alaska State Virology Laboratory	Alaska State Virology Laboratory	Stephanie DeRonde, Elva House, Lisa Smith, Ph.D., Jack Chen, Ph.D.
hCoV-19/USA/AK-PHL7901/2021	EPI_ISL_1675129	4/1/2021	Alaska State Virology Laboratory	Alaska State Virology Laboratory	Stephanie DeRonde, Elva House, Lisa Smith, Ph.D., Jack Chen, Ph.D.
hCoV-19/USA/AK-PHL7903/2021	EPI_ISL_1675130	4/5/2021	Alaska State Virology Laboratory	Alaska State Virology Laboratory	Stephanie DeRonde, Elva House, Lisa Smith, Ph.D., Jack Chen, Ph.D.
hCoV-19/USA/AK-PHL7904/2021	EPI_ISL_1675131	3/28/2021	Alaska State Virology Laboratory	Alaska State Virology Laboratory	Stephanie DeRonde, Elva House, Lisa Smith, Ph.D., Jack Chen, Ph.D.
hCoV-19/USA/AK-PHL7179/2021	EPI_ISL_1195913	2/15/2021	Alaska State Virology Laboratory	Alaska State Virology Laboratory	Stephanie DeRonde, Elva House, Lisa Smith, Ph.D., Jack Chen, Ph.D.
hCoV-19/USA/AK-PHL7183/2021	EPI_ISL_1195916	2/15/2021	Alaska State Virology Laboratory	Alaska State Virology Laboratory	Stephanie DeRonde, Elva House, Lisa Smith, Ph.D., Jack Chen, Ph.D.
hCoV-19/USA/AK-PHL7191/2021	EPI_ISL_1195924	2/15/2021	Alaska State Virology Laboratory	Alaska State Virology Laboratory	Stephanie DeRonde, Elva House, Lisa Smith, Ph.D., Jack Chen, Ph.D.
hCoV-19/USA/AK-PHL7200/2021	EPI_ISL_1195925	2/16/2021	Alaska State Virology Laboratory	Alaska State Virology Laboratory	Stephanie DeRonde, Elva House, Lisa Smith, Ph.D., Jack Chen, Ph.D.
hCoV-19/USA/AK-PHL7203/2021	EPI_ISL_1195928	2/16/2021	Alaska State Virology Laboratory	Alaska State Virology Laboratory	Stephanie DeRonde, Elva House, Lisa Smith, Ph.D., Jack Chen, Ph.D.
hCoV-19/USA/AK-PHL7205/2021	EPI_ISL_1195930	2/16/2021	Alaska State Virology Laboratory	Alaska State Virology Laboratory	Stephanie DeRonde, Elva House, Lisa Smith, Ph.D., Jack Chen, Ph.D.
hCoV-19/USA/AK-PHL7319/2021	EPI_ISL_1195966	2/16/2021	Alaska State Virology Laboratory	Alaska State Virology Laboratory	Stephanie DeRonde, Elva House, Lisa Smith, Ph.D., Jack Chen, Ph.D.
hCoV-19/USA/AK-PHL7320/2021	EPI_ISL_1195967	2/15/2021	Alaska State Virology Laboratory	Alaska State Virology Laboratory	Stephanie DeRonde, Elva House, Lisa Smith, Ph.D., Jack Chen, Ph.D.
hCoV-19/USA/AK-PHL7323/2021	EPI_ISL_1195970	2/18/2021	Alaska State Virology Laboratory	Alaska State Virology Laboratory	Stephanie DeRonde, Elva House, Lisa Smith, Ph.D., Jack Chen, Ph.D.
hCoV-19/USA/AK-PHL7348/2021	EPI_ISL_1195989	2/20/2021	Alaska State Virology Laboratory	Alaska State Virology Laboratory	Stephanie DeRonde, Elva House, Lisa Smith, Ph.D., Jack Chen, Ph.D.
hCoV-19/USA/AK-CDC-LC0042932/2021	EPI_ISL_1683846	4/6/2021	Laboratory Corporation of America	Centers for Disease Control and Prevention Division of Viral Diseases, Pathogen Discovery	Dakota Howard, Dhwani Batra, Peter W. Cook, Kara Moser, Adrian Paskey, Jason Caravas, Benjamin Rambo-Martin, Shatavia Morrison, Christopher Gulvick, Scott Sammons, Yvette Unoarumhi, Darlene Wagner, Matthew Schmerer, Minoo Agarwal, Eyad Almasri, Debbie Boles, Ayla Burns, Nuthawin Charoensri, Oren Cohen, Susan Countryman, Mary Ann Cristobal, Bobbi Croy, Suzanne Dale, Hrushikesh Deshmukh, Amanda Douglas, Vincent Drouillon, Marcia Eisenberg, Howard Engler, Rama Ghatti, Prashant Gupta, Susan Hicks, Jake Humphrey, Lax Iyer, Manoj Jain, Mohan Kolli, Brian Krueger, Tim Kuphal, Stanley Letovsky, Michael Levandoski, Craig Lukasik, Jonathan Meltzer, Brian Norvell, Mindy Nye, Scott Parker, Christos Petropoulos, John Pruitt, Steven Ragan, Scott Ryan, Mike Sapeta, Jana Schroth, Suresh Babu Selvaraju, Goran Stevovic, Amanda Suchanek, Andrea Throop, Lyndon Tilson, Thomas Urban, Joe Voshell, Kimberly Wagner, Jonathan Williams, Mary Williamson, Qian Zeng, Tricia Zwiefelhofer, Clinton R. Paden, Duncan MacCannell
hCoV-19/USA/AK-CDC-ASC210028278/2021	EPI_ISL_1688223	3/28/2021	Aegis Sciences Corporation	Centers for Disease Control and Prevention Division of Viral Diseases, Pathogen Discovery	Dakota Howard, Dhwani Batra, Peter W. Cook, Kara Moser, Adrian Paskey, Jason Caravas, Benjamin Rambo-Martin, Shatavia Morrison, Christopher Gulvick, Scott Sammons, Yvette Unoarumhi, Darlene Wagner, Matthew Schmerer, Cyndi Clark, Patrick Campbell, Rob Case, Vikramsinha Ghorpade, Holly Houdeshell, Ola Kvalvaag, Dillon Nall, Ethan Sanders, Alec Vest, Shaun Westlund, Matthew Hardison, Clinton R. Paden, Duncan MacCannell
hCoV-19/USA/AK-PHL7861/2021	EPI_ISL_1675096	4/1/2021	Alaska State Virology Laboratory	Alaska State Virology Laboratory	Stephanie DeRonde, Elva House, Lisa Smith, Ph.D., Jack Chen, Ph.D.
hCoV-19/USA/AK-PHL7871/2021	EPI_ISL_1675104	4/1/2021	Alaska State Virology Laboratory	Alaska State Virology Laboratory	Stephanie DeRonde, Elva House, Lisa Smith, Ph.D., Jack Chen, Ph.D.
hCoV-19/USA/AK-PHL7876/2021	EPI_ISL_1675109	3/29/2021	Alaska State Virology Laboratory	Alaska State Virology Laboratory	Stephanie DeRonde, Elva House, Lisa Smith, Ph.D., Jack Chen, Ph.D.
hCoV-19/USA/AK-PHL7885/2021	EPI_ISL_1675115	3/31/2021	Alaska State Virology Laboratory	Alaska State Virology Laboratory	Stephanie DeRonde, Elva House, Lisa Smith, Ph.D., Jack Chen, Ph.D.
hCoV-19/USA/AK-PHL7899/2021	EPI_ISL_1675127	4/5/2021	Alaska State Virology Laboratory	Alaska State Virology Laboratory	Stephanie DeRonde, Elva House, Lisa Smith, Ph.D., Jack Chen, Ph.D.
hCoV-19/USA/AK-PHL8014/2021	EPI_ISL_1701683	4/5/2021	Alaska State Virology Laboratory	Alaska State Virology Laboratory	Stephanie DeRonde, Elva House, Lisa Smith, Ph.D., Jack Chen, Ph.D.
hCoV-19/USA/AK-PHL8019/2021	EPI_ISL_1701685	4/5/2021	Alaska State Virology Laboratory	Alaska State Virology Laboratory	Stephanie DeRonde, Elva House, Lisa Smith, Ph.D., Jack Chen, Ph.D.
hCoV-19/USA/AK-PHL8021/2021	EPI_ISL_1701687	4/5/2021	Alaska State Virology Laboratory	Alaska State Virology Laboratory	Stephanie DeRonde, Elva House, Lisa Smith, Ph.D., Jack Chen, Ph.D.
hCoV-19/USA/AK-PHL8023/2021	EPI_ISL_1701689	4/6/2021	Alaska State Virology Laboratory	Alaska State Virology Laboratory	Stephanie DeRonde, Elva House, Lisa Smith, Ph.D., Jack Chen, Ph.D.
hCoV-19/USA/AK-PHL8030/2021	EPI_ISL_1701692	4/7/2021	Alaska State Virology Laboratory	Alaska State Virology Laboratory	Stephanie DeRonde, Elva House, Lisa Smith, Ph.D., Jack Chen, Ph.D.
hCoV-19/USA/AK-PHL8032/2021	EPI_ISL_1701693	3/7/2021	Alaska State Virology Laboratory	Alaska State Virology Laboratory	Stephanie DeRonde, Elva House, Lisa Smith, Ph.D., Jack Chen, Ph.D.

hCoV-19/USA/AK-PHL8037/2021	EPI_ISL_1701696	3/27/2021	Alaska State Virology Laboratory	Alaska State Virology Laboratory	Stephanie DeRonde, Elva House, Lisa Smith, Ph.D., Jack Chen, Ph.D.
hCoV-19/USA/AK-PHL8041/2021	EPI_ISL_1701699	3/26/2021	Alaska State Virology Laboratory	Alaska State Virology Laboratory	Stephanie DeRonde, Elva House, Lisa Smith, Ph.D., Jack Chen, Ph.D.
hCoV-19/USA/AK-PHL8042/2021	EPI_ISL_1701700	3/26/2021	Alaska State Virology Laboratory	Alaska State Virology Laboratory	Stephanie DeRonde, Elva House, Lisa Smith, Ph.D., Jack Chen, Ph.D.
hCoV-19/USA/AK-PHL8043/2021	EPI_ISL_1701701	3/26/2021	Alaska State Virology Laboratory	Alaska State Virology Laboratory	Stephanie DeRonde, Elva House, Lisa Smith, Ph.D., Jack Chen, Ph.D.
hCoV-19/USA/AK-PHL8044/2021	EPI_ISL_1701702	3/26/2021	Alaska State Virology Laboratory	Alaska State Virology Laboratory	Stephanie DeRonde, Elva House, Lisa Smith, Ph.D., Jack Chen, Ph.D.
hCoV-19/USA/AK-PHL8045/2021	EPI_ISL_1701703	4/10/2021	Alaska State Virology Laboratory	Alaska State Virology Laboratory	Stephanie DeRonde, Elva House, Lisa Smith, Ph.D., Jack Chen, Ph.D.
hCoV-19/USA/AK-PHL8046/2021	EPI_ISL_1701704	4/8/2021	Alaska State Virology Laboratory	Alaska State Virology Laboratory	Stephanie DeRonde, Elva House, Lisa Smith, Ph.D., Jack Chen, Ph.D.
hCoV-19/USA/AK-PHL8047/2021	EPI_ISL_1701705	4/12/2021	Alaska State Virology Laboratory	Alaska State Virology Laboratory	Stephanie DeRonde, Elva House, Lisa Smith, Ph.D., Jack Chen, Ph.D.
hCoV-19/USA/AK-PHL8048/2021	EPI_ISL_1701706	4/13/2021	Alaska State Virology Laboratory	Alaska State Virology Laboratory	Stephanie DeRonde, Elva House, Lisa Smith, Ph.D., Jack Chen, Ph.D.
hCoV-19/USA/AK-PHL8050/2021	EPI_ISL_1701708	4/14/2021	Alaska State Virology Laboratory	Alaska State Virology Laboratory	Stephanie DeRonde, Elva House, Lisa Smith, Ph.D., Jack Chen, Ph.D.
hCoV-19/USA/AK-PHL8051/2021	EPI_ISL_1701709	4/11/2021	Alaska State Virology Laboratory	Alaska State Virology Laboratory	Stephanie DeRonde, Elva House, Lisa Smith, Ph.D., Jack Chen, Ph.D.
hCoV-19/USA/AK-PHL8053/2021	EPI_ISL_1701710	4/10/2021	Alaska State Virology Laboratory	Alaska State Virology Laboratory	Stephanie DeRonde, Elva House, Lisa Smith, Ph.D., Jack Chen, Ph.D.
hCoV-19/USA/AK-PHL8054/2021	EPI_ISL_1701711	4/15/2021	Alaska State Virology Laboratory	Alaska State Virology Laboratory	Stephanie DeRonde, Elva House, Lisa Smith, Ph.D., Jack Chen, Ph.D.
hCoV-19/USA/AK-PHL8055/2021	EPI_ISL_1701712	4/14/2021	Alaska State Virology Laboratory	Alaska State Virology Laboratory	Stephanie DeRonde, Elva House, Lisa Smith, Ph.D., Jack Chen, Ph.D.
hCoV-19/USA/AK-PHL8056/2021	EPI_ISL_1701713	4/15/2021	Alaska State Virology Laboratory	Alaska State Virology Laboratory	Stephanie DeRonde, Elva House, Lisa Smith, Ph.D., Jack Chen, Ph.D.
hCoV-19/USA/AK-PHL8058/2021	EPI_ISL_1701715	4/12/2021	Alaska State Virology Laboratory	Alaska State Virology Laboratory	Stephanie DeRonde, Elva House, Lisa Smith, Ph.D., Jack Chen, Ph.D.
hCoV-19/USA/AK-PHL8059/2021	EPI_ISL_1701716	4/12/2021	Alaska State Virology Laboratory	Alaska State Virology Laboratory	Stephanie DeRonde, Elva House, Lisa Smith, Ph.D., Jack Chen, Ph.D.
hCoV-19/USA/AK-PHL6659/2020	EPI_ISL_806837	12/3/2020	Alaska State Virology Laboratory	Alaska State Virology Laboratory	Stephanie DeRonde, Lisa Smith, Ph.D., Devin M. Drown, Ph.D., Jack Chen, Ph.D.
hCoV-19/USA/AK-PHL6664/2020	EPI_ISL_806842	12/6/2020	Alaska State Virology Laboratory	Alaska State Virology Laboratory	Stephanie DeRonde, Lisa Smith, Ph.D., Devin M. Drown, Ph.D., Jack Chen, Ph.D.
hCoV-19/USA/AK-PHL6667/2020	EPI_ISL_806845	12/10/2020	Alaska State Virology Laboratory	Alaska State Virology Laboratory	Stephanie DeRonde, Lisa Smith, Ph.D., Devin M. Drown, Ph.D., Jack Chen, Ph.D.
hCoV-19/USA/AK-PHL6668/2020	EPI_ISL_806846	12/18/2020	Alaska State Virology Laboratory	Alaska State Virology Laboratory	Stephanie DeRonde, Lisa Smith, Ph.D., Devin M. Drown, Ph.D., Jack Chen, Ph.D.
hCoV-19/USA/AK-PHL6672/2020	EPI_ISL_806850	12/10/2020	Alaska State Virology Laboratory	Alaska State Virology Laboratory	Stephanie DeRonde, Lisa Smith, Ph.D., Devin M. Drown, Ph.D., Jack Chen, Ph.D.
hCoV-19/USA/AK-PHL8106/2021	EPI_ISL_1893798	4/6/2021	Alaska State Virology Laboratory	Alaska State Virology Laboratory	Stephanie DeRonde, Elva House, Jacob Zidek, Lisa Smith, Ph.D., Jack Chen, Ph.D.
hCoV-19/USA/AK-PHL8107/2021	EPI_ISL_1893800	4/9/2021	Alaska State Virology Laboratory	Alaska State Virology Laboratory	Stephanie DeRonde, Elva House, Jacob Zidek, Lisa Smith, Ph.D., Jack Chen, Ph.D.
hCoV-19/USA/AK-PHL8108/2021	EPI_ISL_1893802	4/8/2021	Alaska State Virology Laboratory	Alaska State Virology Laboratory	Stephanie DeRonde, Elva House, Jacob Zidek, Lisa Smith, Ph.D., Jack Chen, Ph.D.
hCoV-19/USA/AK-PHL8109/2021	EPI_ISL_1893805	4/12/2021	Alaska State Virology Laboratory	Alaska State Virology Laboratory	Stephanie DeRonde, Elva House, Jacob Zidek, Lisa Smith, Ph.D., Jack Chen, Ph.D.
hCoV-19/USA/AK-PHL8111/2021	EPI_ISL_1893811	4/7/2021	Alaska State Virology Laboratory	Alaska State Virology Laboratory	Stephanie DeRonde, Elva House, Jacob Zidek, Lisa Smith, Ph.D., Jack Chen, Ph.D.
hCoV-19/USA/AK-PHL8118/2021	EPI_ISL_1893818	4/12/2021	Alaska State Virology Laboratory	Alaska State Virology Laboratory	Stephanie DeRonde, Elva House, Jacob Zidek, Lisa Smith, Ph.D., Jack Chen, Ph.D.
hCoV-19/USA/AK-PHL8120/2021	EPI_ISL_1893820	4/14/2021	Alaska State Virology Laboratory	Alaska State Virology Laboratory	Stephanie DeRonde, Elva House, Jacob Zidek, Lisa Smith, Ph.D., Jack Chen, Ph.D.
hCoV-19/USA/AK-PHL8121/2021	EPI_ISL_1893822	4/14/2021	Alaska State Virology Laboratory	Alaska State Virology Laboratory	Stephanie DeRonde, Elva House, Jacob Zidek, Lisa Smith, Ph.D., Jack Chen, Ph.D.
hCoV-19/USA/AK-PHL8125/2021	EPI_ISL_1893843	4/13/2021	Alaska State Virology Laboratory	Alaska State Virology Laboratory	Stephanie DeRonde, Elva House, Jacob Zidek, Lisa Smith, Ph.D., Jack Chen, Ph.D.
hCoV-19/USA/AK-PHL8127/2021	EPI_ISL_1893849	4/17/2021	Alaska State Virology Laboratory	Alaska State Virology Laboratory	Stephanie DeRonde, Elva House, Jacob Zidek, Lisa Smith, Ph.D., Jack Chen, Ph.D.
hCoV-19/USA/AK-PHL8131/2021	EPI_ISL_1893869	4/14/2021	Alaska State Virology Laboratory	Alaska State Virology Laboratory	Stephanie DeRonde, Elva House, Jacob Zidek, Lisa Smith, Ph.D., Jack Chen, Ph.D.
hCoV-19/USA/AK-PHL8132/2021	EPI_ISL_1893872	4/14/2021	Alaska State Virology Laboratory	Alaska State Virology Laboratory	Stephanie DeRonde, Elva House, Jacob Zidek, Lisa Smith, Ph.D., Jack Chen, Ph.D.
hCoV-19/USA/AK-PHL8133/2021	EPI_ISL_1893875	4/14/2021	Alaska State Virology Laboratory	Alaska State Virology Laboratory	Stephanie DeRonde, Elva House, Jacob Zidek, Lisa Smith, Ph.D., Jack Chen, Ph.D.
hCoV-19/USA/AK-PHL8136/2021	EPI_ISL_1893879	4/7/2021	Alaska State Virology Laboratory	Alaska State Virology Laboratory	Stephanie DeRonde, Elva House, Jacob Zidek, Lisa Smith, Ph.D., Jack Chen, Ph.D.
hCoV-19/USA/AK-PHL8137/2021	EPI_ISL_1893881	4/16/2021	Alaska State Virology Laboratory	Alaska State Virology Laboratory	Stephanie DeRonde, Elva House, Jacob Zidek, Lisa Smith, Ph.D., Jack Chen, Ph.D.
hCoV-19/USA/AK-PHL8138/2021	EPI_ISL_1893883	4/15/2021	Alaska State Virology Laboratory	Alaska State Virology Laboratory	Stephanie DeRonde, Elva House, Jacob Zidek, Lisa Smith, Ph.D., Jack Chen, Ph.D.
hCoV-19/USA/AK-PHL8139/2021	EPI_ISL_1893886	4/12/2021	Alaska State Virology Laboratory	Alaska State Virology Laboratory	Stephanie DeRonde, Elva House, Jacob Zidek, Lisa Smith, Ph.D., Jack Chen, Ph.D.
hCoV-19/USA/AK-PHL8140/2021	EPI_ISL_1893888	4/15/2021	Alaska State Virology Laboratory	Alaska State Virology Laboratory	Stephanie DeRonde, Elva House, Jacob Zidek, Lisa Smith, Ph.D., Jack Chen, Ph.D.
hCoV-19/USA/AK-PHL8141/2021	EPI_ISL_1893890	4/15/2021	Alaska State Virology Laboratory	Alaska State Virology Laboratory	Stephanie DeRonde, Elva House, Jacob Zidek, Lisa Smith, Ph.D., Jack Chen, Ph.D.
hCoV-19/USA/AK-PHL6820/2021	EPI_ISL_1039734	1/27/2021	Alaska State Virology Laboratory	Alaska State Virology Laboratory	Stephanie DeRonde, Lisa Smith, Ph.D., Jack Chen, Ph.D.
hCoV-19/USA/AK-PHL6808/2021	EPI_ISL_1039736	2/2/2021	Alaska State Virology Laboratory	Alaska State Virology Laboratory	Stephanie DeRonde, Lisa Smith, Ph.D., Jack Chen, Ph.D.
hCoV-19/USA/AK-RD555-CV0047/2021	EPI_ISL_1068640	2/4/2021	Fulgent Genetics	Fulgent Genetics	Harry Gao, Mickey Li, John Gao, Joseph Fierro, Benafsh Sapra, Becky Tsai, Yan Meng, Doreen Ng, James Xie
hCoV-19/USA/AK-PHL7370/2021	EPI_ISL_1743688	2/25/2021	Alaska State Virology Laboratory	Alaska State Virology Laboratory	Stephanie DeRonde, Elva House, Lisa Smith, Ph.D., Jack Chen, Ph.D.
hCoV-19/USA/AK-PHL7380/2021	EPI_ISL_1743689	2/4/2021	Alaska State Virology Laboratory	Alaska State Virology Laboratory	Stephanie DeRonde, Elva House, Lisa Smith, Ph.D., Jack Chen, Ph.D.
hCoV-19/USA/AK-PHL7381/2021	EPI_ISL_1743690	2/22/2021	Alaska State Virology Laboratory	Alaska State Virology Laboratory	Stephanie DeRonde, Elva House, Lisa Smith, Ph.D., Jack Chen, Ph.D.
hCoV-19/USA/AK-PHL7382/2021	EPI_ISL_1743691	2/8/2021	Alaska State Virology Laboratory	Alaska State Virology Laboratory	Stephanie DeRonde, Elva House, Lisa Smith, Ph.D., Jack Chen, Ph.D.
hCoV-19/USA/AK-PHL7383/2021	EPI_ISL_1743692	2/11/2021	Alaska State Virology Laboratory	Alaska State Virology Laboratory	Stephanie DeRonde, Elva House, Lisa Smith, Ph.D., Jack Chen, Ph.D.
hCoV-19/USA/AK-PHL7384/2021	EPI_ISL_1743693	2/2/2021	Alaska State Virology Laboratory	Alaska State Virology Laboratory	Stephanie DeRonde, Elva House, Lisa Smith, Ph.D., Jack Chen, Ph.D.

hCoV-19/USA/AK-PHL7386/2021	EPI_ISL_1743694	2/25/2021	Alaska State Virology Laboratory	Alaska State Virology Laboratory	Stephanie DeRonde, Elva House, Lisa Smith, Ph.D., Jack Chen, Ph.D.
hCoV-19/USA/AK-PHL7387/2021	EPI_ISL_1743695	2/24/2021	Alaska State Virology Laboratory	Alaska State Virology Laboratory	Stephanie DeRonde, Elva House, Lisa Smith, Ph.D., Jack Chen, Ph.D.
hCoV-19/USA/AK-PHL7389/2021	EPI_ISL_1743696	2/11/2021	Alaska State Virology Laboratory	Alaska State Virology Laboratory	Stephanie DeRonde, Elva House, Lisa Smith, Ph.D., Jack Chen, Ph.D.
hCoV-19/USA/AK-PHL7390/2021	EPI_ISL_1743697	2/11/2021	Alaska State Virology Laboratory	Alaska State Virology Laboratory	Stephanie DeRonde, Elva House, Lisa Smith, Ph.D., Jack Chen, Ph.D.
hCoV-19/USA/AK-PHL7394/2021	EPI_ISL_1743700	2/4/2021	Alaska State Virology Laboratory	Alaska State Virology Laboratory	Stephanie DeRonde, Elva House, Lisa Smith, Ph.D., Jack Chen, Ph.D.
hCoV-19/USA/AK-PHL7396/2021	EPI_ISL_1743701	2/4/2021	Alaska State Virology Laboratory	Alaska State Virology Laboratory	Stephanie DeRonde, Elva House, Lisa Smith, Ph.D., Jack Chen, Ph.D.
hCoV-19/USA/AK-PHL7397/2021	EPI_ISL_1743702	2/5/2021	Alaska State Virology Laboratory	Alaska State Virology Laboratory	Stephanie DeRonde, Elva House, Lisa Smith, Ph.D., Jack Chen, Ph.D.
hCoV-19/USA/AK-PHL7403/2021	EPI_ISL_1743705	2/27/2021	Alaska State Virology Laboratory	Alaska State Virology Laboratory	Stephanie DeRonde, Elva House, Lisa Smith, Ph.D., Jack Chen, Ph.D.
hCoV-19/USA/AK-PHL7404/2021	EPI_ISL_1743706	2/27/2021	Alaska State Virology Laboratory	Alaska State Virology Laboratory	Stephanie DeRonde, Elva House, Lisa Smith, Ph.D., Jack Chen, Ph.D.
hCoV-19/USA/AK-PHL7405/2021	EPI_ISL_1743707	2/27/2021	Alaska State Virology Laboratory	Alaska State Virology Laboratory	Stephanie DeRonde, Elva House, Lisa Smith, Ph.D., Jack Chen, Ph.D.
hCoV-19/USA/AK-PHL7406/2021	EPI_ISL_1743708	2/26/2021	Alaska State Virology Laboratory	Alaska State Virology Laboratory	Stephanie DeRonde, Elva House, Lisa Smith, Ph.D., Jack Chen, Ph.D.
hCoV-19/USA/AK-PHL7407/2021	EPI_ISL_1743709	2/18/2021	Alaska State Virology Laboratory	Alaska State Virology Laboratory	Stephanie DeRonde, Elva House, Lisa Smith, Ph.D., Jack Chen, Ph.D.
hCoV-19/USA/AK-PHL7408/2021	EPI_ISL_1743710	2/18/2021	Alaska State Virology Laboratory	Alaska State Virology Laboratory	Stephanie DeRonde, Elva House, Lisa Smith, Ph.D., Jack Chen, Ph.D.
hCoV-19/USA/AK-PHL7459/2021	EPI_ISL_1743713	3/6/2021	Alaska State Virology Laboratory	Alaska State Virology Laboratory	Stephanie DeRonde, Elva House, Lisa Smith, Ph.D., Jack Chen, Ph.D.
hCoV-19/USA/AK-PHL7460/2021	EPI_ISL_1743714	3/6/2021	Alaska State Virology Laboratory	Alaska State Virology Laboratory	Stephanie DeRonde, Elva House, Lisa Smith, Ph.D., Jack Chen, Ph.D.
hCoV-19/USA/AK-PHL8060/2021	EPI_ISL_1754868	4/12/2021	Alaska State Virology Laboratory	Alaska State Virology Laboratory	Stephanie DeRonde, Elva House, Lisa Smith, Ph.D., Jack Chen, Ph.D.
hCoV-19/USA/AK-PHL8061/2021	EPI_ISL_1754869	4/12/2021	Alaska State Virology Laboratory	Alaska State Virology Laboratory	Stephanie DeRonde, Elva House, Lisa Smith, Ph.D., Jack Chen, Ph.D.
hCoV-19/USA/AK-PHL8064/2021	EPI_ISL_1754870	4/12/2021	Alaska State Virology Laboratory	Alaska State Virology Laboratory	Stephanie DeRonde, Elva House, Lisa Smith, Ph.D., Jack Chen, Ph.D.
hCoV-19/USA/AK-PHL8065/2021	EPI_ISL_1754871	4/9/2021	Alaska State Virology Laboratory	Alaska State Virology Laboratory	Stephanie DeRonde, Elva House, Lisa Smith, Ph.D., Jack Chen, Ph.D.
hCoV-19/USA/AK-PHL8067/2021	EPI_ISL_1754872	4/10/2021	Alaska State Virology Laboratory	Alaska State Virology Laboratory	Stephanie DeRonde, Elva House, Lisa Smith, Ph.D., Jack Chen, Ph.D.
hCoV-19/USA/AK-PHL8070/2021	EPI_ISL_1754874	4/8/2021	Alaska State Virology Laboratory	Alaska State Virology Laboratory	Stephanie DeRonde, Elva House, Lisa Smith, Ph.D., Jack Chen, Ph.D.
hCoV-19/USA/AK-PHL8071/2021	EPI_ISL_1754875	4/7/2021	Alaska State Virology Laboratory	Alaska State Virology Laboratory	Stephanie DeRonde, Elva House, Lisa Smith, Ph.D., Jack Chen, Ph.D.
hCoV-19/USA/AK-PHL8076/2021	EPI_ISL_1754876	4/7/2021	Alaska State Virology Laboratory	Alaska State Virology Laboratory	Stephanie DeRonde, Elva House, Lisa Smith, Ph.D., Jack Chen, Ph.D.
hCoV-19/USA/AK-PHL8073/2021	EPI_ISL_1754877	4/13/2021	Alaska State Virology Laboratory	Alaska State Virology Laboratory	Stephanie DeRonde, Elva House, Lisa Smith, Ph.D., Jack Chen, Ph.D.
hCoV-19/USA/AK-PHL8077/2021	EPI_ISL_1754878	4/6/2021	Alaska State Virology Laboratory	Alaska State Virology Laboratory	Stephanie DeRonde, Elva House, Lisa Smith, Ph.D., Jack Chen, Ph.D.
hCoV-19/USA/AK-PHL8078/2021	EPI_ISL_1754879	4/7/2021	Alaska State Virology Laboratory	Alaska State Virology Laboratory	Stephanie DeRonde, Elva House, Lisa Smith, Ph.D., Jack Chen, Ph.D.
hCoV-19/USA/AK-PHL8079/2021	EPI_ISL_1754880	4/8/2021	Alaska State Virology Laboratory	Alaska State Virology Laboratory	Stephanie DeRonde, Elva House, Lisa Smith, Ph.D., Jack Chen, Ph.D.
hCoV-19/USA/AK-PHL8081/2021	EPI_ISL_1754881	4/15/2021	Alaska State Virology Laboratory	Alaska State Virology Laboratory	Stephanie DeRonde, Elva House, Lisa Smith, Ph.D., Jack Chen, Ph.D.
hCoV-19/USA/AK-PHL8085/2021	EPI_ISL_1754884	4/15/2021	Alaska State Virology Laboratory	Alaska State Virology Laboratory	Stephanie DeRonde, Elva House, Lisa Smith, Ph.D., Jack Chen, Ph.D.
hCoV-19/USA/AK-PHL8088/2021	EPI_ISL_1754887	4/9/2021	Alaska State Virology Laboratory	Alaska State Virology Laboratory	Stephanie DeRonde, Elva House, Lisa Smith, Ph.D., Jack Chen, Ph.D.
hCoV-19/USA/AK-PHL8089/2021	EPI_ISL_1754888	4/9/2021	Alaska State Virology Laboratory	Alaska State Virology Laboratory	Stephanie DeRonde, Elva House, Lisa Smith, Ph.D., Jack Chen, Ph.D.
hCoV-19/USA/AK-PHL8090/2021	EPI_ISL_1754889	4/13/2021	Alaska State Virology Laboratory	Alaska State Virology Laboratory	Stephanie DeRonde, Elva House, Lisa Smith, Ph.D., Jack Chen, Ph.D.
hCoV-19/USA/AK-PHL8092/2021	EPI_ISL_1754891	4/6/2021	Alaska State Virology Laboratory	Alaska State Virology Laboratory	Stephanie DeRonde, Elva House, Lisa Smith, Ph.D., Jack Chen, Ph.D.
hCoV-19/USA/AK-PHL8093/2021	EPI_ISL_1754892	4/8/2021	Alaska State Virology Laboratory	Alaska State Virology Laboratory	Stephanie DeRonde, Elva House, Lisa Smith, Ph.D., Jack Chen, Ph.D.
hCoV-19/USA/AK-PHL8099/2021	EPI_ISL_1754894	4/12/2021	Alaska State Virology Laboratory	Alaska State Virology Laboratory	Stephanie DeRonde, Elva House, Lisa Smith, Ph.D., Jack Chen, Ph.D.
hCoV-19/USA/AK-PHL8100/2021	EPI_ISL_1754895	4/11/2021	Alaska State Virology Laboratory	Alaska State Virology Laboratory	Stephanie DeRonde, Elva House, Lisa Smith, Ph.D., Jack Chen, Ph.D.
hCoV-19/USA/AK-PHL8103/2021	EPI_ISL_1754896	3/31/2021	Alaska State Virology Laboratory	Alaska State Virology Laboratory	Stephanie DeRonde, Elva House, Lisa Smith, Ph.D., Jack Chen, Ph.D.
hCoV-19/USA/AK-PHL8197/2021	EPI_ISL_1789630	3/31/2021	Alaska State Virology Laboratory	Alaska State Virology Laboratory	Stephanie DeRonde, Elva House, Lisa Smith, Ph.D., Jack Chen, Ph.D.
hCoV-19/USA/AK-PHL8199/2021	EPI_ISL_1789631	4/6/2021	Alaska State Virology Laboratory	Alaska State Virology Laboratory	Stephanie DeRonde, Elva House, Lisa Smith, Ph.D., Jack Chen, Ph.D.
hCoV-19/USA/AK-PHL8200/2021	EPI_ISL_1789632	4/18/2021	Alaska State Virology Laboratory	Alaska State Virology Laboratory	Stephanie DeRonde, Elva House, Lisa Smith, Ph.D., Jack Chen, Ph.D.
hCoV-19/USA/AK-PHL8202/2021	EPI_ISL_1789634	4/16/2021	Alaska State Virology Laboratory	Alaska State Virology Laboratory	Stephanie DeRonde, Elva House, Lisa Smith, Ph.D., Jack Chen, Ph.D.
hCoV-19/USA/AK-PHL8203/2021	EPI_ISL_1789635	4/16/2021	Alaska State Virology Laboratory	Alaska State Virology Laboratory	Stephanie DeRonde, Elva House, Lisa Smith, Ph.D., Jack Chen, Ph.D.
hCoV-19/USA/AK-PHL8204/2021	EPI_ISL_1789636	4/14/2021	Alaska State Virology Laboratory	Alaska State Virology Laboratory	Stephanie DeRonde, Elva House, Lisa Smith, Ph.D., Jack Chen, Ph.D.
hCoV-19/USA/AK-PHL8205/2021	EPI_ISL_1789637	4/17/2021	Alaska State Virology Laboratory	Alaska State Virology Laboratory	Stephanie DeRonde, Elva House, Lisa Smith, Ph.D., Jack Chen, Ph.D.
hCoV-19/USA/AK-PHL8206/2021	EPI_ISL_1789638	4/17/2021	Alaska State Virology Laboratory	Alaska State Virology Laboratory	Stephanie DeRonde, Elva House, Lisa Smith, Ph.D., Jack Chen, Ph.D.
hCoV-19/USA/AK-PHL8207/2021	EPI_ISL_1789639	4/17/2021	Alaska State Virology Laboratory	Alaska State Virology Laboratory	Stephanie DeRonde, Elva House, Lisa Smith, Ph.D., Jack Chen, Ph.D.
hCoV-19/USA/AK-PHL8208/2021	EPI_ISL_1789640	4/17/2021	Alaska State Virology Laboratory	Alaska State Virology Laboratory	Stephanie DeRonde, Elva House, Lisa Smith, Ph.D., Jack Chen, Ph.D.
hCoV-19/USA/AK-PHL8209/2021	EPI_ISL_1789641	4/17/2021	Alaska State Virology Laboratory	Alaska State Virology Laboratory	Stephanie DeRonde, Elva House, Lisa Smith, Ph.D., Jack Chen, Ph.D.
hCoV-19/USA/AK-PHL8210/2021	EPI_ISL_1789642	4/17/2021	Alaska State Virology Laboratory	Alaska State Virology Laboratory	Stephanie DeRonde, Elva House, Lisa Smith, Ph.D., Jack Chen, Ph.D.
hCoV-19/USA/AK-PHL8212/2021	EPI_ISL_1789643	4/21/2021	Alaska State Virology Laboratory	Alaska State Virology Laboratory	Stephanie DeRonde, Elva House, Lisa Smith, Ph.D., Jack Chen, Ph.D.
hCoV-19/USA/AK-PHL8213/2021	EPI_ISL_1789644	4/20/2021	Alaska State Virology Laboratory	Alaska State Virology Laboratory	Stephanie DeRonde, Elva House, Lisa Smith, Ph.D., Jack Chen, Ph.D.

hCoV-19/USA/AK-PHL8217/2021	EPI_ISL_1789645	4/14/2021	Alaska State Virology Laboratory	Alaska State Virology Laboratory	Stephanie DeRonde, Elva House, Lisa Smith, Ph.D., Jack Chen, Ph.D.
hCoV-19/USA/AK-PHL8218/2021	EPI_ISL_1789646	4/13/2021	Alaska State Virology Laboratory	Alaska State Virology Laboratory	Stephanie DeRonde, Elva House, Lisa Smith, Ph.D., Jack Chen, Ph.D.
hCoV-19/USA/AK-PHL8219/2021	EPI_ISL_1789647	4/13/2021	Alaska State Virology Laboratory	Alaska State Virology Laboratory	Stephanie DeRonde, Elva House, Lisa Smith, Ph.D., Jack Chen, Ph.D.
hCoV-19/USA/AK-PHL8221/2021	EPI_ISL_1789649	4/16/2021	Alaska State Virology Laboratory	Alaska State Virology Laboratory	Stephanie DeRonde, Elva House, Lisa Smith, Ph.D., Jack Chen, Ph.D.
hCoV-19/USA/AK-PHL8222/2021	EPI_ISL_1789650	4/15/2021	Alaska State Virology Laboratory	Alaska State Virology Laboratory	Stephanie DeRonde, Elva House, Lisa Smith, Ph.D., Jack Chen, Ph.D.
hCoV-19/USA/AK-PHL8223/2021	EPI_ISL_1789651	4/19/2021	Alaska State Virology Laboratory	Alaska State Virology Laboratory	Stephanie DeRonde, Elva House, Lisa Smith, Ph.D., Jack Chen, Ph.D.
hCoV-19/USA/AK-PHL8224/2021	EPI_ISL_1789652	4/17/2021	Alaska State Virology Laboratory	Alaska State Virology Laboratory	Stephanie DeRonde, Elva House, Lisa Smith, Ph.D., Jack Chen, Ph.D.
hCoV-19/USA/AK-PHL8225/2021	EPI_ISL_1789653	4/14/2021	Alaska State Virology Laboratory	Alaska State Virology Laboratory	Stephanie DeRonde, Elva House, Lisa Smith, Ph.D., Jack Chen, Ph.D.
hCoV-19/USA/AK-PHL8226/2021	EPI_ISL_1789654	4/15/2021	Alaska State Virology Laboratory	Alaska State Virology Laboratory	Stephanie DeRonde, Elva House, Lisa Smith, Ph.D., Jack Chen, Ph.D.
hCoV-19/USA/AK-PHL8227/2021	EPI_ISL_1789655	4/15/2021	Alaska State Virology Laboratory	Alaska State Virology Laboratory	Stephanie DeRonde, Elva House, Lisa Smith, Ph.D., Jack Chen, Ph.D.
hCoV-19/USA/AK-PHL8228/2021	EPI_ISL_1789657	4/19/2021	Alaska State Virology Laboratory	Alaska State Virology Laboratory	Stephanie DeRonde, Elva House, Lisa Smith, Ph.D., Jack Chen, Ph.D.
hCoV-19/USA/AK-PHL8229/2021	EPI_ISL_1789658	4/19/2021	Alaska State Virology Laboratory	Alaska State Virology Laboratory	Stephanie DeRonde, Elva House, Lisa Smith, Ph.D., Jack Chen, Ph.D.
hCoV-19/USA/AK-PHL8230/2021	EPI_ISL_1789659	4/19/2021	Alaska State Virology Laboratory	Alaska State Virology Laboratory	Stephanie DeRonde, Elva House, Lisa Smith, Ph.D., Jack Chen, Ph.D.
hCoV-19/USA/AK-PHL8232/2021	EPI_ISL_1789661	4/19/2021	Alaska State Virology Laboratory	Alaska State Virology Laboratory	Stephanie DeRonde, Elva House, Lisa Smith, Ph.D., Jack Chen, Ph.D.
hCoV-19/USA/AK-PHL8233/2021	EPI_ISL_1789662	4/19/2021	Alaska State Virology Laboratory	Alaska State Virology Laboratory	Stephanie DeRonde, Elva House, Lisa Smith, Ph.D., Jack Chen, Ph.D.
hCoV-19/USA/AK-PHL8234/2021	EPI_ISL_1789663	4/19/2021	Alaska State Virology Laboratory	Alaska State Virology Laboratory	Stephanie DeRonde, Elva House, Lisa Smith, Ph.D., Jack Chen, Ph.D.
hCoV-19/USA/AK-PHL8235/2021	EPI_ISL_1789664	4/19/2021	Alaska State Virology Laboratory	Alaska State Virology Laboratory	Stephanie DeRonde, Elva House, Lisa Smith, Ph.D., Jack Chen, Ph.D.
hCoV-19/USA/AK-PHL8236/2021	EPI_ISL_1789665	4/19/2021	Alaska State Virology Laboratory	Alaska State Virology Laboratory	Stephanie DeRonde, Elva House, Lisa Smith, Ph.D., Jack Chen, Ph.D.
hCoV-19/USA/AK-PHL8237/2021	EPI_ISL_1789666	4/19/2021	Alaska State Virology Laboratory	Alaska State Virology Laboratory	Stephanie DeRonde, Elva House, Lisa Smith, Ph.D., Jack Chen, Ph.D.
hCoV-19/USA/AK-PHL8238/2021	EPI_ISL_1789667	4/19/2021	Alaska State Virology Laboratory	Alaska State Virology Laboratory	Stephanie DeRonde, Elva House, Lisa Smith, Ph.D., Jack Chen, Ph.D.
hCoV-19/USA/AK-PHL8239/2021	EPI_ISL_1789668	4/19/2021	Alaska State Virology Laboratory	Alaska State Virology Laboratory	Stephanie DeRonde, Elva House, Lisa Smith, Ph.D., Jack Chen, Ph.D.
hCoV-19/USA/AK-PHL8241/2021	EPI_ISL_1789669	4/19/2021	Alaska State Virology Laboratory	Alaska State Virology Laboratory	Stephanie DeRonde, Elva House, Lisa Smith, Ph.D., Jack Chen, Ph.D.
hCoV-19/USA/AK-PHL8242/2021	EPI_ISL_1789670	4/19/2021	Alaska State Virology Laboratory	Alaska State Virology Laboratory	Stephanie DeRonde, Elva House, Lisa Smith, Ph.D., Jack Chen, Ph.D.
hCoV-19/USA/AK-CDC-IBX785023940557/2021	EPI_ISL_1796050	4/6/2021	Infinity Biologix	Centers for Disease Control and Prevention Division of Viral Diseases, Pathogen Discovery	Dakota Howard, Dhwani Batra, Peter W. Cook, Kara Moser, Adrian Paskey, Jason Caravas, Benjamin Rambo-Martin, Shatavia Morrison, Christopher Gulvick, Scott Sammons, Yvette Unoarumhi, Darlene Wagner, Matthew Schmerer, Christian Bixby, Yihe Wang, Jonathan Schultz, Chirayu Goswami, Russ Hager, Robin Grimwood, Clinton R. Paden, Duncan MacCannell
hCoV-19/USA/AK-CDC-LC0044709/2021	EPI_ISL_1798718	4/9/2021	Laboratory Corporation of America	Centers for Disease Control and Prevention Division of Viral Diseases, Pathogen Discovery	Dakota Howard, Dhwani Batra, Peter W. Cook, Kara Moser, Adrian Paskey, Jason Caravas, Benjamin Rambo-Martin, Shatavia Morrison, Christopher Gulvick, Scott Sammons, Yvette Unoarumhi, Darlene Wagner, Matthew Schmerer, Minoo Agarwal, Eyad Almasri, Debbie Boles, Ayla Burns, Nuthawin Charoensri, Oren Cohen, Susan Countryman, Mary Ann Cristobal, Bobbi Croy, Suzanne Dale, Hrushikesh Deshmukh, Amanda Douglas, Vincent Drouillon, Marcia Eisenberg, Howard Engler, Rama Ghatti, Prashant Gupta, Susan Hicks, Jake Humphrey, Lax Iyer, Manoj Jain, Mohan Kolli, Brian Krueger, Tim Kuphal, Stanley Letovsky, Michael Levandoski, Craig Lukasik, Jonathan Meltzer, Brian Norvell, Mindy Nye, Scott Parker, Christos Petropoulos, John Pruitt, Steven Ragan, Scott Ryan, Mike Sapeta, Jana Schroth, Suresh Babu Selvaraju, Goran Stevovic, Amanda Suchanek, Andrea Throop, Lyndon Tilson, Thomas Urban, Joe Voshell, Kimberly Wagner, Jonathan Williams, Mary Williamson, Qian Zeng, Tricia Zwiefelhofer, Clinton R. Paden, Duncan MacCannell
hCoV-19/USA/AK-PHL8244/2021	EPI_ISL_1821886	3/27/2021	Alaska State Virology Laboratory	Alaska State Virology Laboratory	Stephanie DeRonde, Elva House, Jacob Zidek, Lisa Smith, Ph.D., Jack Chen, Ph.D.
hCoV-19/USA/AK-PHL8245/2021	EPI_ISL_1821887	4/3/2021	Alaska State Virology Laboratory	Alaska State Virology Laboratory	Stephanie DeRonde, Elva House, Jacob Zidek, Lisa Smith, Ph.D., Jack Chen, Ph.D.
hCoV-19/USA/AK-PHL8247/2021	EPI_ISL_1821888	4/12/2021	Alaska State Virology Laboratory	Alaska State Virology Laboratory	Stephanie DeRonde, Elva House, Jacob Zidek, Lisa Smith, Ph.D., Jack Chen, Ph.D.
hCoV-19/USA/AK-PHL8248/2021	EPI_ISL_1821889	4/13/2021	Alaska State Virology Laboratory	Alaska State Virology Laboratory	Stephanie DeRonde, Elva House, Jacob Zidek, Lisa Smith, Ph.D., Jack Chen, Ph.D.
hCoV-19/USA/AK-PHL7984/2021	EPI_ISL_1821890	4/6/2021	Alaska State Virology Laboratory	Alaska State Virology Laboratory	Stephanie DeRonde, Elva House, Jacob Zidek, Lisa Smith, Ph.D., Jack Chen, Ph.D.
hCoV-19/USA/AK-PHL8249/2021	EPI_ISL_1821891	4/19/2021	Alaska State Virology Laboratory	Alaska State Virology Laboratory	Stephanie DeRonde, Elva House, Jacob Zidek, Lisa Smith, Ph.D., Jack Chen, Ph.D.
hCoV-19/USA/AK-PHL8251/2021	EPI_ISL_1821892	4/17/2021	Alaska State Virology Laboratory	Alaska State Virology Laboratory	Stephanie DeRonde, Elva House, Jacob Zidek, Lisa Smith, Ph.D., Jack Chen, Ph.D.
hCoV-19/USA/AK-PHL8252/2021	EPI_ISL_1821893	4/17/2021	Alaska State Virology Laboratory	Alaska State Virology Laboratory	Stephanie DeRonde, Elva House, Jacob Zidek, Lisa Smith, Ph.D., Jack Chen, Ph.D.
hCoV-19/USA/AK-PHL8254/2021	EPI_ISL_1821894	4/19/2021	Alaska State Virology Laboratory	Alaska State Virology Laboratory	Stephanie DeRonde, Elva House, Jacob Zidek, Lisa Smith, Ph.D., Jack Chen, Ph.D.
hCoV-19/USA/AK-PHL8255/2021	EPI_ISL_1821895	4/18/2021	Alaska State Virology Laboratory	Alaska State Virology Laboratory	Stephanie DeRonde, Elva House, Jacob Zidek, Lisa Smith, Ph.D., Jack Chen, Ph.D.
hCoV-19/USA/AK-PHL8256/2021	EPI_ISL_1821896	4/14/2021	Alaska State Virology Laboratory	Alaska State Virology Laboratory	Stephanie DeRonde, Elva House, Jacob Zidek, Lisa Smith, Ph.D., Jack Chen, Ph.D.

hCoV-19/USA/AK-PHL8257/2021	EPI_ISL_1821897	4/14/2021	Alaska State Virology Laboratory	Alaska State Virology Laboratory	Stephanie DeRonde, Elva House, Jacob Zidek, Lisa Smith, Ph.D., Jack Chen, Ph.D.
hCoV-19/USA/AK-PHL8258/2021	EPI_ISL_1821898	4/17/2021	Alaska State Virology Laboratory	Alaska State Virology Laboratory	Stephanie DeRonde, Elva House, Jacob Zidek, Lisa Smith, Ph.D., Jack Chen, Ph.D.
hCoV-19/USA/AK-PHL8261/2021	EPI_ISL_1821900	4/20/2021	Alaska State Virology Laboratory	Alaska State Virology Laboratory	Stephanie DeRonde, Elva House, Jacob Zidek, Lisa Smith, Ph.D., Jack Chen, Ph.D.
hCoV-19/USA/AK-PHL8263/2021	EPI_ISL_1821901	4/22/2021	Alaska State Virology Laboratory	Alaska State Virology Laboratory	Stephanie DeRonde, Elva House, Jacob Zidek, Lisa Smith, Ph.D., Jack Chen, Ph.D.
hCoV-19/USA/AK-PHL8269/2021	EPI_ISL_1821905	4/17/2021	Alaska State Virology Laboratory	Alaska State Virology Laboratory	Stephanie DeRonde, Elva House, Jacob Zidek, Lisa Smith, Ph.D., Jack Chen, Ph.D.
hCoV-19/USA/AK-PHL8270/2021	EPI_ISL_1821906	4/19/2021	Alaska State Virology Laboratory	Alaska State Virology Laboratory	Stephanie DeRonde, Elva House, Jacob Zidek, Lisa Smith, Ph.D., Jack Chen, Ph.D.
hCoV-19/USA/AK-PHL8272/2021	EPI_ISL_1821908	4/19/2021	Alaska State Virology Laboratory	Alaska State Virology Laboratory	Stephanie DeRonde, Elva House, Jacob Zidek, Lisa Smith, Ph.D., Jack Chen, Ph.D.
hCoV-19/USA/AK-PHL8273/2021	EPI_ISL_1821909	4/14/2021	Alaska State Virology Laboratory	Alaska State Virology Laboratory	Stephanie DeRonde, Elva House, Jacob Zidek, Lisa Smith, Ph.D., Jack Chen, Ph.D.
hCoV-19/USA/AK-PHL8274/2021	EPI_ISL_1821910	4/14/2021	Alaska State Virology Laboratory	Alaska State Virology Laboratory	Stephanie DeRonde, Elva House, Jacob Zidek, Lisa Smith, Ph.D., Jack Chen, Ph.D.
hCoV-19/USA/AK-PHL8275/2021	EPI_ISL_1821911	4/19/2021	Alaska State Virology Laboratory	Alaska State Virology Laboratory	Stephanie DeRonde, Elva House, Jacob Zidek, Lisa Smith, Ph.D., Jack Chen, Ph.D.
hCoV-19/USA/AK-PHL8276/2021	EPI_ISL_1821912	4/19/2021	Alaska State Virology Laboratory	Alaska State Virology Laboratory	Stephanie DeRonde, Elva House, Jacob Zidek, Lisa Smith, Ph.D., Jack Chen, Ph.D.
hCoV-19/USA/AK-PHL8278/2021	EPI_ISL_1821914	4/22/2021	Alaska State Virology Laboratory	Alaska State Virology Laboratory	Stephanie DeRonde, Elva House, Jacob Zidek, Lisa Smith, Ph.D., Jack Chen, Ph.D.
hCoV-19/USA/AK-PHL8279/2021	EPI_ISL_1821915	4/21/2021	Alaska State Virology Laboratory	Alaska State Virology Laboratory	Stephanie DeRonde, Elva House, Jacob Zidek, Lisa Smith, Ph.D., Jack Chen, Ph.D.
hCoV-19/USA/AK-PHL8280/2021	EPI_ISL_1821916	4/21/2021	Alaska State Virology Laboratory	Alaska State Virology Laboratory	Stephanie DeRonde, Elva House, Jacob Zidek, Lisa Smith, Ph.D., Jack Chen, Ph.D.
hCoV-19/USA/AK-PHL8281/2021	EPI_ISL_1821917	4/21/2021	Alaska State Virology Laboratory	Alaska State Virology Laboratory	Stephanie DeRonde, Elva House, Jacob Zidek, Lisa Smith, Ph.D., Jack Chen, Ph.D.
hCoV-19/USA/AK-PHL8282/2021	EPI_ISL_1821918	4/14/2021	Alaska State Virology Laboratory	Alaska State Virology Laboratory	Stephanie DeRonde, Elva House, Jacob Zidek, Lisa Smith, Ph.D., Jack Chen, Ph.D.
hCoV-19/USA/AK-CDC-2-4242684/2021	EPI_ISL_1823929	4/6/2021	AK State Public Health Lab, State Health Department	Centers for Disease Control and Prevention Division of Viral Diseases, Pathogen Discovery	Mili Sheth, Sarah Nobles, Jasmine Padilla, Mark Burroughs, Shoshona Le, Katie Dillon, Peter Cook, Clinton R. Paden, Dhwani Batra, Krista Queen, Kristen Knipe, Dakota Howard, Yvette Unoarumhi, Darlene Wagner, Matthew Schmerer, Ben L. Rambo-Martin, Kristine Lacek, Sam Shepard, Alison Laufer Halpin, Dave Wentworth, Vivien Dugan, Suxiang Tong, Justin Lee
hCoV-19/USA/AK-CDC-2-4242591/2021	EPI_ISL_1823930	4/1/2021	AK State Public Health Lab, State Health Department	Centers for Disease Control and Prevention Division of Viral Diseases, Pathogen Discovery	Mili Sheth, Sarah Nobles, Jasmine Padilla, Mark Burroughs, Shoshona Le, Katie Dillon, Peter Cook, Clinton R. Paden, Dhwani Batra, Krista Queen, Kristen Knipe, Dakota Howard, Yvette Unoarumhi, Darlene Wagner, Matthew Schmerer, Ben L. Rambo-Martin, Kristine Lacek, Sam Shepard, Alison Laufer Halpin, Dave Wentworth, Vivien Dugan, Suxiang Tong, Justin Lee
hCoV-19/USA/AK-CDC-2-4242689/2021	EPI_ISL_1823931	4/3/2021	AK State Public Health Lab, State Health Department	Centers for Disease Control and Prevention Division of Viral Diseases, Pathogen Discovery	Mili Sheth, Sarah Nobles, Jasmine Padilla, Mark Burroughs, Shoshona Le, Katie Dillon, Peter Cook, Clinton R. Paden, Dhwani Batra, Krista Queen, Kristen Knipe, Dakota Howard, Yvette Unoarumhi, Darlene Wagner, Matthew Schmerer, Ben L. Rambo-Martin, Kristine Lacek, Sam Shepard, Alison Laufer Halpin, Dave Wentworth, Vivien Dugan, Suxiang Tong, Justin Lee
hCoV-19/USA/AK-CDC-2-4242669/2021	EPI_ISL_1823932	4/7/2021	AK State Public Health Lab, State Health Department	Centers for Disease Control and Prevention Division of Viral Diseases, Pathogen Discovery	Mili Sheth, Sarah Nobles, Jasmine Padilla, Mark Burroughs, Shoshona Le, Katie Dillon, Peter Cook, Clinton R. Paden, Dhwani Batra, Krista Queen, Kristen Knipe, Dakota Howard, Yvette Unoarumhi, Darlene Wagner, Matthew Schmerer, Ben L. Rambo-Martin, Kristine Lacek, Sam Shepard, Alison Laufer Halpin, Dave Wentworth, Vivien Dugan, Suxiang Tong, Justin Lee
hCoV-19/USA/AK-CDC-2-4242582/2021	EPI_ISL_1823933	4/1/2021	AK State Public Health Lab, State Health Department	Centers for Disease Control and Prevention Division of Viral Diseases, Pathogen Discovery	Mili Sheth, Sarah Nobles, Jasmine Padilla, Mark Burroughs, Shoshona Le, Katie Dillon, Peter Cook, Clinton R. Paden, Dhwani Batra, Krista Queen, Kristen Knipe, Dakota Howard, Yvette Unoarumhi, Darlene Wagner, Matthew Schmerer, Ben L. Rambo-Martin, Kristine Lacek, Sam Shepard, Alison Laufer Halpin, Dave Wentworth, Vivien Dugan, Suxiang Tong, Justin Lee
hCoV-19/USA/AK-CDC-2-4242670/2021	EPI_ISL_1823934	4/1/2021	AK State Public Health Lab, State Health Department	Centers for Disease Control and Prevention Division of Viral Diseases, Pathogen Discovery	Mili Sheth, Sarah Nobles, Jasmine Padilla, Mark Burroughs, Shoshona Le, Katie Dillon, Peter Cook, Clinton R. Paden, Dhwani Batra, Krista Queen, Kristen Knipe, Dakota Howard, Yvette Unoarumhi, Darlene Wagner, Matthew Schmerer, Ben L. Rambo-Martin, Kristine Lacek, Sam Shepard, Alison Laufer Halpin, Dave Wentworth, Vivien Dugan, Suxiang Tong, Justin Lee
hCoV-19/USA/AK-CDC-2-4242569/2021	EPI_ISL_1823935	4/1/2021	AK State Public Health Lab, State Health Department	Centers for Disease Control and Prevention Division of Viral Diseases, Pathogen Discovery	Mili Sheth, Sarah Nobles, Jasmine Padilla, Mark Burroughs, Shoshona Le, Katie Dillon, Peter Cook, Clinton R. Paden, Dhwani Batra, Krista Queen, Kristen Knipe, Dakota Howard, Yvette Unoarumhi, Darlene Wagner, Matthew Schmerer, Ben L. Rambo-Martin, Kristine Lacek, Sam Shepard, Alison Laufer Halpin, Dave Wentworth, Vivien Dugan, Suxiang Tong, Justin Lee

hCoV-19/USA/AK-CDC-2-4242588/2021	EPI_ISL_1823936	4/5/2021	AK State Public Health Lab, State Health Department	Centers for Disease Control and Prevention Division of Viral Diseases, Pathogen Discovery	Mili Sheth, Sarah Nobles, Jasmine Padilla, Mark Burroughs, Shoshona Le, Katie Dillon, Peter Cook, Clinton R. Paden, Dhwani Batra, Krista Queen, Kristen Knipe, Dakota Howard, Yvette Unoarumhi, Darlene Wagner, Matthew Schmerer, Ben L. Rambo-Martin, Kristine Lacek, Sam Shepard, Alison Laufer Halpin, Dave Wentworth, Vivien Dugan, Suxiang Tong, Justin Lee
hCoV-19/USA/AK-CDC-2-4242556/2021	EPI_ISL_1823937	4/3/2021	AK State Public Health Lab, State Health Department	Centers for Disease Control and Prevention Division of Viral Diseases, Pathogen Discovery	Mili Sheth, Sarah Nobles, Jasmine Padilla, Mark Burroughs, Shoshona Le, Katie Dillon, Peter Cook, Clinton R. Paden, Dhwani Batra, Krista Queen, Kristen Knipe, Dakota Howard, Yvette Unoarumhi, Darlene Wagner, Matthew Schmerer, Ben L. Rambo-Martin, Kristine Lacek, Sam Shepard, Alison Laufer Halpin, Dave Wentworth, Vivien Dugan, Suxiang Tong, Justin Lee
hCoV-19/USA/AK-CDC-2-4242660/2021	EPI_ISL_1823938	4/1/2021	AK State Public Health Lab, State Health Department	Centers for Disease Control and Prevention Division of Viral Diseases, Pathogen Discovery	Mili Sheth, Sarah Nobles, Jasmine Padilla, Mark Burroughs, Shoshona Le, Katie Dillon, Peter Cook, Clinton R. Paden, Dhwani Batra, Krista Queen, Kristen Knipe, Dakota Howard, Yvette Unoarumhi, Darlene Wagner, Matthew Schmerer, Ben L. Rambo-Martin, Kristine Lacek, Sam Shepard, Alison Laufer Halpin, Dave Wentworth, Vivien Dugan, Suxiang Tong, Justin Lee
hCoV-19/USA/AK-CDC-2-4242678/2021	EPI_ISL_1823940	4/6/2021	AK State Public Health Lab, State Health Department	Centers for Disease Control and Prevention Division of Viral Diseases, Pathogen Discovery	Mili Sheth, Sarah Nobles, Jasmine Padilla, Mark Burroughs, Shoshona Le, Katie Dillon, Peter Cook, Clinton R. Paden, Dhwani Batra, Krista Queen, Kristen Knipe, Dakota Howard, Yvette Unoarumhi, Darlene Wagner, Matthew Schmerer, Ben L. Rambo-Martin, Kristine Lacek, Sam Shepard, Alison Laufer Halpin, Dave Wentworth, Vivien Dugan, Suxiang Tong, Justin Lee
hCoV-19/USA/AK-CDC-2-4242568/2021	EPI_ISL_1823941	4/6/2021	AK State Public Health Lab, State Health Department	Centers for Disease Control and Prevention Division of Viral Diseases, Pathogen Discovery	Mili Sheth, Sarah Nobles, Jasmine Padilla, Mark Burroughs, Shoshona Le, Katie Dillon, Peter Cook, Clinton R. Paden, Dhwani Batra, Krista Queen, Kristen Knipe, Dakota Howard, Yvette Unoarumhi, Darlene Wagner, Matthew Schmerer, Ben L. Rambo-Martin, Kristine Lacek, Sam Shepard, Alison Laufer Halpin, Dave Wentworth, Vivien Dugan, Suxiang Tong, Justin Lee
hCoV-19/USA/AK-CDC-2-4242562/2021	EPI_ISL_1823942	4/5/2021	AK State Public Health Lab, State Health Department	Centers for Disease Control and Prevention Division of Viral Diseases, Pathogen Discovery	Mili Sheth, Sarah Nobles, Jasmine Padilla, Mark Burroughs, Shoshona Le, Katie Dillon, Peter Cook, Clinton R. Paden, Dhwani Batra, Krista Queen, Kristen Knipe, Dakota Howard, Yvette Unoarumhi, Darlene Wagner, Matthew Schmerer, Ben L. Rambo-Martin, Kristine Lacek, Sam Shepard, Alison Laufer Halpin, Dave Wentworth, Vivien Dugan, Suxiang Tong, Justin Lee
hCoV-19/USA/AK-CDC-2-4242583/2021	EPI_ISL_1823943	4/6/2021	AK State Public Health Lab, State Health Department	Centers for Disease Control and Prevention Division of Viral Diseases, Pathogen Discovery	Mili Sheth, Sarah Nobles, Jasmine Padilla, Mark Burroughs, Shoshona Le, Katie Dillon, Peter Cook, Clinton R. Paden, Dhwani Batra, Krista Queen, Kristen Knipe, Dakota Howard, Yvette Unoarumhi, Darlene Wagner, Matthew Schmerer, Ben L. Rambo-Martin, Kristine Lacek, Sam Shepard, Alison Laufer Halpin, Dave Wentworth, Vivien Dugan, Suxiang Tong, Justin Lee
hCoV-19/USA/AK-CDC-ASC210041459/2021	EPI_ISL_1835209	4/1/2021	Aegis Sciences Corporation	Centers for Disease Control and Prevention Division of Viral Diseases, Pathogen Discovery	Dakota Howard, Dhwani Batra, Peter W. Cook, Kara Moser, Adrian Paskey, Jason Caravas, Benjamin Rambo-Martin, Shatavia Morrison, Christopher Gulvick, Scott Sammons, Yvette Unoarumhi, Darlene Wagner, Matthew Schmerer, Cyndi Clark, Patrick Campbell, Rob Case, Vikramsinha Ghorpade, Holly Houdeshell, Ola Kvalvaag, Dillon Nall, Ethan Sanders, Alec Vest, Shaun Westlund, Matthew Hardison, Clinton R. Paden, Duncan MacCannell
hCoV-19/USA/AK-CDC-ASC210049916/2021	EPI_ISL_1835480	4/7/2021	Aegis Sciences Corporation	Centers for Disease Control and Prevention Division of Viral Diseases, Pathogen Discovery	Dakota Howard, Dhwani Batra, Peter W. Cook, Kara Moser, Adrian Paskey, Jason Caravas, Benjamin Rambo-Martin, Shatavia Morrison, Christopher Gulvick, Scott Sammons, Yvette Unoarumhi, Darlene Wagner, Matthew Schmerer, Cyndi Clark, Patrick Campbell, Rob Case, Vikramsinha Ghorpade, Holly Houdeshell, Ola Kvalvaag, Dillon Nall, Ethan Sanders, Alec Vest, Shaun Westlund, Matthew Hardison, Clinton R. Paden, Duncan MacCannell
hCoV-19/USA/AK-CDC-ASC210049917/2021	EPI_ISL_1835481	4/7/2021	Aegis Sciences Corporation	Centers for Disease Control and Prevention Division of Viral Diseases, Pathogen Discovery	Dakota Howard, Dhwani Batra, Peter W. Cook, Kara Moser, Adrian Paskey, Jason Caravas, Benjamin Rambo-Martin, Shatavia Morrison, Christopher Gulvick, Scott Sammons, Yvette Unoarumhi, Darlene Wagner, Matthew Schmerer, Cyndi Clark, Patrick Campbell, Rob Case, Vikramsinha Ghorpade, Holly Houdeshell, Ola Kvalvaag, Dillon Nall, Ethan Sanders, Alec Vest, Shaun Westlund, Matthew Hardison, Clinton R. Paden, Duncan MacCannell

hCoV-19/USA/AK-CDC-ASC210049382/2021	EPI_ISL_1837645	4/5/2021	Aegis Sciences Corporation	Centers for Disease Control and Prevention Division of Viral Diseases, Pathogen Discovery	Dakota Howard, Dhwani Batra, Peter W. Cook, Kara Moser, Adrian Paskey, Jason Caravas, Benjamin Rambo-Martin, Shatavia Morrison, Christopher Gulvick, Scott Sammons, Yvette Unoarumhi, Darlene Wagner, Matthew Schmerer, Cyndi Clark, Patrick Campbell, Rob Case, Vikramsinha Ghorpade, Holly Houdeshell, Ola Kvalvaag, Dillon Nall, Ethan Sanders, Alec Vest, Shaun Westlund, Matthew Hardison, Clinton R. Paden, Duncan MacCannell
hCoV-19/USA/AK-CDC-ASC210049384/2021	EPI_ISL_1837646	4/6/2021	Aegis Sciences Corporation	Centers for Disease Control and Prevention Division of Viral Diseases, Pathogen Discovery	Dakota Howard, Dhwani Batra, Peter W. Cook, Kara Moser, Adrian Paskey, Jason Caravas, Benjamin Rambo-Martin, Shatavia Morrison, Christopher Gulvick, Scott Sammons, Yvette Unoarumhi, Darlene Wagner, Matthew Schmerer, Cyndi Clark, Patrick Campbell, Rob Case, Vikramsinha Ghorpade, Holly Houdeshell, Ola Kvalvaag, Dillon Nall, Ethan Sanders, Alec Vest, Shaun Westlund, Matthew Hardison, Clinton R. Paden, Duncan MacCannell
hCoV-19/USA/AK-CDC-ASC210053157/2021	EPI_ISL_1838090	4/7/2021	Aegis Sciences Corporation	Centers for Disease Control and Prevention Division of Viral Diseases, Pathogen Discovery	Dakota Howard, Dhwani Batra, Peter W. Cook, Kara Moser, Adrian Paskey, Jason Caravas, Benjamin Rambo-Martin, Shatavia Morrison, Christopher Gulvick, Scott Sammons, Yvette Unoarumhi, Darlene Wagner, Matthew Schmerer, Cyndi Clark, Patrick Campbell, Rob Case, Vikramsinha Ghorpade, Holly Houdeshell, Ola Kvalvaag, Dillon Nall, Ethan Sanders, Alec Vest, Shaun Westlund, Matthew Hardison, Clinton R. Paden, Duncan MacCannell
hCoV-19/USA/AK-PHL7928/2021	EPI_ISL_1621162	4/6/2021	Alaska State Virology Laboratory	Alaska State Virology Laboratory	Stephanie DeRonde, Elva House, Lisa Smith, Ph.D., Jack Chen, Ph.D.
hCoV-19/USA/AK-PHL8231/2021	EPI_ISL_1789660	4/19/2021	Alaska State Virology Laboratory	Alaska State Virology Laboratory	Stephanie DeRonde, Elva House, Lisa Smith, Ph.D., Jack Chen, Ph.D.
hCoV-19/USA/AK-PHL8271/2021	EPI_ISL_1821907	4/19/2021	Alaska State Virology Laboratory	Alaska State Virology Laboratory	Stephanie DeRonde, Elva House, Jacob Zidek, Lisa Smith, Ph.D., Jack Chen, Ph.D.
hCoV-19/USA/AK-PHL8201/2021	EPI_ISL_1789633	4/16/2021	Alaska State Virology Laboratory	Alaska State Virology Laboratory	Stephanie DeRonde, Elva House, Lisa Smith, Ph.D., Jack Chen, Ph.D.
hCoV-19/USA/AK-PHL8220/2021	EPI_ISL_1789648	4/13/2021	Alaska State Virology Laboratory	Alaska State Virology Laboratory	Stephanie DeRonde, Elva House, Lisa Smith, Ph.D., Jack Chen, Ph.D.
hCoV-19/USA/AK-PHL7631/2021	EPI_ISL_1529088	3/16/2021	Alaska State Virology Laboratory	Alaska State Virology Laboratory	Stephanie DeRonde, Elva House, Lisa Smith, Ph.D., Jack Chen, Ph.D.
hCoV-19/USA/AK-PHL8105/2021	EPI_ISL_1893796	4/1/2021	Alaska State Virology Laboratory	Alaska State Virology Laboratory	Stephanie DeRonde, Elva House, Jacob Zidek, Lisa Smith, Ph.D., Jack Chen, Ph.D.
hCoV-19/USA/AK-PHL8112/2021	EPI_ISL_1893813	4/12/2021	Alaska State Virology Laboratory	Alaska State Virology Laboratory	Stephanie DeRonde, Elva House, Jacob Zidek, Lisa Smith, Ph.D., Jack Chen, Ph.D.
hCoV-19/USA/AK-PHL8113/2021	EPI_ISL_1893815	4/8/2021	Alaska State Virology Laboratory	Alaska State Virology Laboratory	Stephanie DeRonde, Elva House, Jacob Zidek, Lisa Smith, Ph.D., Jack Chen, Ph.D.
hCoV-19/USA/AK-PHL8122/2021	EPI_ISL_1893829	4/16/2021	Alaska State Virology Laboratory	Alaska State Virology Laboratory	Stephanie DeRonde, Elva House, Jacob Zidek, Lisa Smith, Ph.D., Jack Chen, Ph.D.
hCoV-19/USA/AK-PHL8124/2021	EPI_ISL_1893836	4/13/2021	Alaska State Virology Laboratory	Alaska State Virology Laboratory	Stephanie DeRonde, Elva House, Jacob Zidek, Lisa Smith, Ph.D., Jack Chen, Ph.D.
hCoV-19/USA/AK-PHL8128/2021	EPI_ISL_1893856	4/17/2021	Alaska State Virology Laboratory	Alaska State Virology Laboratory	Stephanie DeRonde, Elva House, Jacob Zidek, Lisa Smith, Ph.D., Jack Chen, Ph.D.
hCoV-19/USA/AK-PHL8129/2021	EPI_ISL_1893861	4/15/2021	Alaska State Virology Laboratory	Alaska State Virology Laboratory	Stephanie DeRonde, Elva House, Jacob Zidek, Lisa Smith, Ph.D., Jack Chen, Ph.D.
hCoV-19/USA/AK-PHL8130/2021	EPI_ISL_1893867	4/15/2021	Alaska State Virology Laboratory	Alaska State Virology Laboratory	Stephanie DeRonde, Elva House, Jacob Zidek, Lisa Smith, Ph.D., Jack Chen, Ph.D.
hCoV-19/USA/AK-PHL8135/2021	EPI_ISL_1893877	4/14/2021	Alaska State Virology Laboratory	Alaska State Virology Laboratory	Stephanie DeRonde, Elva House, Jacob Zidek, Lisa Smith, Ph.D., Jack Chen, Ph.D.
hCoV-19/USA/AK-PHL8143/2021	EPI_ISL_1893892	4/12/2021	Alaska State Virology Laboratory	Alaska State Virology Laboratory	Stephanie DeRonde, Elva House, Jacob Zidek, Lisa Smith, Ph.D., Jack Chen, Ph.D.
hCoV-19/USA/AK-PHL7821/2021	EPI_ISL_1575043	4/1/2021	Alaska State Virology Laboratory	Alaska State Virology Laboratory	Stephanie DeRonde, Elva House, Lisa Smith, Ph.D., Jack Chen, Ph.D.
hCoV-19/USA/AK-PHL7826/2021	EPI_ISL_1575047	3/29/2021	Alaska State Virology Laboratory	Alaska State Virology Laboratory	Stephanie DeRonde, Elva House, Lisa Smith, Ph.D., Jack Chen, Ph.D.
hCoV-19/USA/AK-PHL7832/2021	EPI_ISL_1575053	3/30/2021	Alaska State Virology Laboratory	Alaska State Virology Laboratory	Stephanie DeRonde, Elva House, Lisa Smith, Ph.D., Jack Chen, Ph.D.
hCoV-19/USA/AK-PHL7837/2021	EPI_ISL_1575058	3/30/2021	Alaska State Virology Laboratory	Alaska State Virology Laboratory	Stephanie DeRonde, Elva House, Lisa Smith, Ph.D., Jack Chen, Ph.D.
hCoV-19/USA/AK-PHL7838/2021	EPI_ISL_1575059	3/29/2021	Alaska State Virology Laboratory	Alaska State Virology Laboratory	Stephanie DeRonde, Elva House, Lisa Smith, Ph.D., Jack Chen, Ph.D.
hCoV-19/USA/AK-PHL7841/2021	EPI_ISL_1575062	3/29/2021	Alaska State Virology Laboratory	Alaska State Virology Laboratory	Stephanie DeRonde, Elva House, Lisa Smith, Ph.D., Jack Chen, Ph.D.
hCoV-19/USA/AK-PHL7845/2021	EPI_ISL_1575066	3/30/2021	Alaska State Virology Laboratory	Alaska State Virology Laboratory	Stephanie DeRonde, Elva House, Lisa Smith, Ph.D., Jack Chen, Ph.D.
hCoV-19/USA/AK-PHL7848/2021	EPI_ISL_1575069	3/29/2021	Alaska State Virology Laboratory	Alaska State Virology Laboratory	Stephanie DeRonde, Elva House, Lisa Smith, Ph.D., Jack Chen, Ph.D.
hCoV-19/USA/AK-PHL7853/2021	EPI_ISL_1575074	3/30/2021	Alaska State Virology Laboratory	Alaska State Virology Laboratory	Stephanie DeRonde, Elva House, Lisa Smith, Ph.D., Jack Chen, Ph.D.
hCoV-19/USA/AK-PHL7913/2021	EPI_ISL_1621159	4/2/2021	Alaska State Virology Laboratory	Alaska State Virology Laboratory	Stephanie DeRonde, Elva House, Lisa Smith, Ph.D., Jack Chen, Ph.D.
hCoV-19/USA/AK-PHL7914/2021	EPI_ISL_1621160	4/3/2021	Alaska State Virology Laboratory	Alaska State Virology Laboratory	Stephanie DeRonde, Elva House, Lisa Smith, Ph.D., Jack Chen, Ph.D.
hCoV-19/USA/AK-PHL7918/2021	EPI_ISL_1621161	4/3/2021	Alaska State Virology Laboratory	Alaska State Virology Laboratory	Stephanie DeRonde, Elva House, Lisa Smith, Ph.D., Jack Chen, Ph.D.
hCoV-19/USA/AK-PHL7936/2021	EPI_ISL_1621163	4/5/2021	Alaska State Virology Laboratory	Alaska State Virology Laboratory	Stephanie DeRonde, Elva House, Lisa Smith, Ph.D., Jack Chen, Ph.D.
hCoV-19/USA/AK-PHL7941/2021	EPI_ISL_1621164	4/5/2021	Alaska State Virology Laboratory	Alaska State Virology Laboratory	Stephanie DeRonde, Elva House, Lisa Smith, Ph.D., Jack Chen, Ph.D.
hCoV-19/USA/AK-PHL7908/2021	EPI_ISL_1621166	3/31/2021	Alaska State Virology Laboratory	Alaska State Virology Laboratory	Stephanie DeRonde, Elva House, Lisa Smith, Ph.D., Jack Chen, Ph.D.
hCoV-19/USA/AK-PHL7924/2021	EPI_ISL_1621169	4/4/2021	Alaska State Virology Laboratory	Alaska State Virology Laboratory	Stephanie DeRonde, Elva House, Lisa Smith, Ph.D., Jack Chen, Ph.D.
hCoV-19/USA/AK-PHL7905/2021	EPI_ISL_1621170	4/7/2021	Alaska State Virology Laboratory	Alaska State Virology Laboratory	Stephanie DeRonde, Elva House, Lisa Smith, Ph.D., Jack Chen, Ph.D.
hCoV-19/USA/AK-PHL7906/2021	EPI_ISL_1621171	4/7/2021	Alaska State Virology Laboratory	Alaska State Virology Laboratory	Stephanie DeRonde, Elva House, Lisa Smith, Ph.D., Jack Chen, Ph.D.

hCoV-19/USA/AK-PHL7907/2021	EPI_ISL_1621172	3/31/2021	Alaska State Virology Laboratory	Alaska State Virology Laboratory	Stephanie DeRonde, Elva House, Lisa Smith, Ph.D., Jack Chen, Ph.D.
hCoV-19/USA/AK-PHL7909/2021	EPI_ISL_1621173	3/31/2021	Alaska State Virology Laboratory	Alaska State Virology Laboratory	Stephanie DeRonde, Elva House, Lisa Smith, Ph.D., Jack Chen, Ph.D.
hCoV-19/USA/AK-PHL7912/2021	EPI_ISL_1621175	4/3/2021	Alaska State Virology Laboratory	Alaska State Virology Laboratory	Stephanie DeRonde, Elva House, Lisa Smith, Ph.D., Jack Chen, Ph.D.
hCoV-19/USA/AK-PHL7916/2021	EPI_ISL_1621176	4/3/2021	Alaska State Virology Laboratory	Alaska State Virology Laboratory	Stephanie DeRonde, Elva House, Lisa Smith, Ph.D., Jack Chen, Ph.D.
hCoV-19/USA/AK-PHL7920/2021	EPI_ISL_1621177	4/4/2021	Alaska State Virology Laboratory	Alaska State Virology Laboratory	Stephanie DeRonde, Elva House, Lisa Smith, Ph.D., Jack Chen, Ph.D.
hCoV-19/USA/AK-PHL7922/2021	EPI_ISL_1621178	4/4/2021	Alaska State Virology Laboratory	Alaska State Virology Laboratory	Stephanie DeRonde, Elva House, Lisa Smith, Ph.D., Jack Chen, Ph.D.
hCoV-19/USA/AK-PHL7925/2021	EPI_ISL_1621179	4/2/2021	Alaska State Virology Laboratory	Alaska State Virology Laboratory	Stephanie DeRonde, Elva House, Lisa Smith, Ph.D., Jack Chen, Ph.D.
hCoV-19/USA/AK-PHL7926/2021	EPI_ISL_1621180	4/2/2021	Alaska State Virology Laboratory	Alaska State Virology Laboratory	Stephanie DeRonde, Elva House, Lisa Smith, Ph.D., Jack Chen, Ph.D.
hCoV-19/USA/AK-PHL7927/2021	EPI_ISL_1621181	4/7/2021	Alaska State Virology Laboratory	Alaska State Virology Laboratory	Stephanie DeRonde, Elva House, Lisa Smith, Ph.D., Jack Chen, Ph.D.
hCoV-19/USA/AK-PHL7930/2021	EPI_ISL_1621182	4/3/2021	Alaska State Virology Laboratory	Alaska State Virology Laboratory	Stephanie DeRonde, Elva House, Lisa Smith, Ph.D., Jack Chen, Ph.D.
hCoV-19/USA/AK-PHL7931/2021	EPI_ISL_1621183	4/3/2021	Alaska State Virology Laboratory	Alaska State Virology Laboratory	Stephanie DeRonde, Elva House, Lisa Smith, Ph.D., Jack Chen, Ph.D.
hCoV-19/USA/AK-PHL7932/2021	EPI_ISL_1621184	4/7/2021	Alaska State Virology Laboratory	Alaska State Virology Laboratory	Stephanie DeRonde, Elva House, Lisa Smith, Ph.D., Jack Chen, Ph.D.
hCoV-19/USA/AK-PHL7933/2021	EPI_ISL_1621185	4/5/2021	Alaska State Virology Laboratory	Alaska State Virology Laboratory	Stephanie DeRonde, Elva House, Lisa Smith, Ph.D., Jack Chen, Ph.D.
hCoV-19/USA/AK-PHL7940/2021	EPI_ISL_1621188	4/5/2021	Alaska State Virology Laboratory	Alaska State Virology Laboratory	Stephanie DeRonde, Elva House, Lisa Smith, Ph.D., Jack Chen, Ph.D.
hCoV-19/USA/AK-PHL7942/2021	EPI_ISL_1621189	4/5/2021	Alaska State Virology Laboratory	Alaska State Virology Laboratory	Stephanie DeRonde, Elva House, Lisa Smith, Ph.D., Jack Chen, Ph.D.
hCoV-19/USA/AK-PHL7943/2021	EPI_ISL_1621190	4/5/2021	Alaska State Virology Laboratory	Alaska State Virology Laboratory	Stephanie DeRonde, Elva House, Lisa Smith, Ph.D., Jack Chen, Ph.D.
hCoV-19/USA/AK-PHL7944/2021	EPI_ISL_1621191	4/5/2021	Alaska State Virology Laboratory	Alaska State Virology Laboratory	Stephanie DeRonde, Elva House, Lisa Smith, Ph.D., Jack Chen, Ph.D.
hCoV-19/USA/AK-PHL7945/2021	EPI_ISL_1621192	4/5/2021	Alaska State Virology Laboratory	Alaska State Virology Laboratory	Stephanie DeRonde, Elva House, Lisa Smith, Ph.D., Jack Chen, Ph.D.
hCoV-19/USA/AK-PHL7946/2021	EPI_ISL_1621193	4/5/2021	Alaska State Virology Laboratory	Alaska State Virology Laboratory	Stephanie DeRonde, Elva House, Lisa Smith, Ph.D., Jack Chen, Ph.D.
hCoV-19/USA/AK-PHL7947/2021	EPI_ISL_1621194	4/5/2021	Alaska State Virology Laboratory	Alaska State Virology Laboratory	Stephanie DeRonde, Elva House, Lisa Smith, Ph.D., Jack Chen, Ph.D.
hCoV-19/USA/AK-PHL7948/2021	EPI_ISL_1621195	4/5/2021	Alaska State Virology Laboratory	Alaska State Virology Laboratory	Stephanie DeRonde, Elva House, Lisa Smith, Ph.D., Jack Chen, Ph.D.
hCoV-19/USA/AK-PHL7949/2021	EPI_ISL_1621196	4/5/2021	Alaska State Virology Laboratory	Alaska State Virology Laboratory	Stephanie DeRonde, Elva House, Lisa Smith, Ph.D., Jack Chen, Ph.D.
hCoV-19/USA/AK-CDC-LC0044931/2021	EPI_ISL_1799072	4/17/2021	Laboratory Corporation of America	Centers for Disease Control and Prevention Division of Viral Diseases, Pathogen Discovery	Dakota Howard, Dhwani Batra, Peter W. Cook, Kara Moser, Adrian Paskey, Jason Caravas, Benjamin Rambo-Martin, Shatavia Morrison, Christopher Gulvick, Scott Sammons, Yvette Unoarumhi, Darlene Wagner, Matthew Schmerer, Minoo Agarwal, Eyad Almasri, Debbie Boles, Ayla Burns, Nuthawin Charoensri, Oren Cohen, Susan Countryman, Mary Ann Cristobal, Bobbi Croy, Suzanne Dale, Hrushikesh Deshmukh, Amanda Douglas, Vincent Drouillon, Marcia Eisenberg, Howard Engler, Rama Ghatti, Prashant Gupta, Susan Hicks, Jake Humphrey, Lax Iyer, Manoj Jain, Mohan Kolli, Brian Krueger, Tim Kuphal, Stanley Letovsky, Michael Levandoski, Craig Lukasik, Jonathan Meltzer, Brian Norvell, Mindy Nye, Scott Parker, Christos Petropoulos, John Pruitt, Steven Ragan, Scott Ryan, Mike Sapeta, Jana Schroth, Suresh Babu Selvaraju, Goran Stevovic, Amanda Suchanek, Andrea Throop, Lyndon Tilson, Thomas Urban, Joe Voshell, Kimberly Wagner, Jonathan Williams, Mary Williamson, Qian Zeng, Tricia Zwiefelhofer, Clinton R. Paden, Duncan MacCannell
hCoV-19/USA/AK-PHL7937/2021	EPI_ISL_1621165	4/5/2021	Alaska State Virology Laboratory	Alaska State Virology Laboratory	Stephanie DeRonde, Elva House, Lisa Smith, Ph.D., Jack Chen, Ph.D.
hCoV-19/USA/AK-PHL7910/2021	EPI_ISL_1621167	3/29/2021	Alaska State Virology Laboratory	Alaska State Virology Laboratory	Stephanie DeRonde, Elva House, Lisa Smith, Ph.D., Jack Chen, Ph.D.
hCoV-19/USA/AK-PHL7939/2021	EPI_ISL_1621168	4/5/2021	Alaska State Virology Laboratory	Alaska State Virology Laboratory	Stephanie DeRonde, Elva House, Lisa Smith, Ph.D., Jack Chen, Ph.D.
hCoV-19/USA/AK-PHL7911/2021	EPI_ISL_1621174	4/2/2021	Alaska State Virology Laboratory	Alaska State Virology Laboratory	Stephanie DeRonde, Elva House, Lisa Smith, Ph.D., Jack Chen, Ph.D.
hCoV-19/USA/AK-PHL7934/2021	EPI_ISL_1621186	4/7/2021	Alaska State Virology Laboratory	Alaska State Virology Laboratory	Stephanie DeRonde, Elva House, Lisa Smith, Ph.D., Jack Chen, Ph.D.
hCoV-19/USA/AK-PHL7938/2021	EPI_ISL_1621187	4/5/2021	Alaska State Virology Laboratory	Alaska State Virology Laboratory	Stephanie DeRonde, Elva House, Lisa Smith, Ph.D., Jack Chen, Ph.D.
hCoV-19/USA/AK-PHL8260/2021	EPI_ISL_1821899	4/20/2021	Alaska State Virology Laboratory	Alaska State Virology Laboratory	Stephanie DeRonde, Elva House, Jacob Zidek, Lisa Smith, Ph.D., Jack Chen, Ph.D.
hCoV-19/USA/AK-PHL8264/2021	EPI_ISL_1821902	4/21/2021	Alaska State Virology Laboratory	Alaska State Virology Laboratory	Stephanie DeRonde, Elva House, Jacob Zidek, Lisa Smith, Ph.D., Jack Chen, Ph.D.
hCoV-19/USA/AK-PHL8266/2021	EPI_ISL_1821903	4/20/2021	Alaska State Virology Laboratory	Alaska State Virology Laboratory	Stephanie DeRonde, Elva House, Jacob Zidek, Lisa Smith, Ph.D., Jack Chen, Ph.D.
hCoV-19/USA/AK-PHL8268/2021	EPI_ISL_1821904	4/18/2021	Alaska State Virology Laboratory	Alaska State Virology Laboratory	Stephanie DeRonde, Elva House, Jacob Zidek, Lisa Smith, Ph.D., Jack Chen, Ph.D.
hCoV-19/USA/AK-PHL8277/2021	EPI_ISL_1821913	4/19/2021	Alaska State Virology Laboratory	Alaska State Virology Laboratory	Stephanie DeRonde, Elva House, Jacob Zidek, Lisa Smith, Ph.D., Jack Chen, Ph.D.
hCoV-19/USA/AK-CDC-2-4242683/2021	EPI_ISL_1823939	4/7/2021	AK State Public Health Lab, State Health Department	Centers for Disease Control and Prevention Division of Viral Diseases, Pathogen Discovery	Mili Sheth, Sarah Nobles, Jasmine Padilla, Mark Burroughs, Shoshona Le, Katie Dillon, Peter Cook, Clinton R. Paden, Dhwani Batra, Krista Queen, Kristen Knipe, Dakota Howard, Yvette Unoarumhi, Darlene Wagner, Matthew Schmerer, Ben L. Rambo-Martin, Kristine Lacek, Sam Shepard, Alison Laufer Halpin, Dave Wentworth, Vivien Dugan, Suxiang Tong, Justin Lee
hCoV-19/USA/AK-PHL7473/2021	EPI_ISL_1652210	3/6/2021	Alaska State Virology Laboratory	Alaska State Virology Laboratory	Stephanie DeRonde, Elva House, Lisa Smith, Ph.D., Jack Chen, Ph.D.

hCoV-19/USA/AK-PHL7482/2021	EPI_ISL_1652218	3/6/2021	Alaska State Virology Laboratory	Alaska State Virology Laboratory	Stephanie DeRonde, Elva House, Lisa Smith, Ph.D., Jack Chen, Ph.D.
hCoV-19/USA/AK-PHL7483/2021	EPI_ISL_1652219	3/6/2021	Alaska State Virology Laboratory	Alaska State Virology Laboratory	Stephanie DeRonde, Elva House, Lisa Smith, Ph.D., Jack Chen, Ph.D.
hCoV-19/USA/AK-PHL7488/2021	EPI_ISL_1652223	3/6/2021	Alaska State Virology Laboratory	Alaska State Virology Laboratory	Stephanie DeRonde, Elva House, Lisa Smith, Ph.D., Jack Chen, Ph.D.
hCoV-19/USA/AK-PHL7492/2021	EPI_ISL_1652226	3/6/2021	Alaska State Virology Laboratory	Alaska State Virology Laboratory	Stephanie DeRonde, Elva House, Lisa Smith, Ph.D., Jack Chen, Ph.D.
hCoV-19/USA/AK-PHL7499/2021	EPI_ISL_1652231	3/6/2021	Alaska State Virology Laboratory	Alaska State Virology Laboratory	Stephanie DeRonde, Elva House, Lisa Smith, Ph.D., Jack Chen, Ph.D.
hCoV-19/USA/AK-PHL7500/2021	EPI_ISL_1652232	3/5/2021	Alaska State Virology Laboratory	Alaska State Virology Laboratory	Stephanie DeRonde, Elva House, Lisa Smith, Ph.D., Jack Chen, Ph.D.
hCoV-19/USA/AK-PHL7502/2021	EPI_ISL_1652233	3/5/2021	Alaska State Virology Laboratory	Alaska State Virology Laboratory	Stephanie DeRonde, Elva House, Lisa Smith, Ph.D., Jack Chen, Ph.D.
hCoV-19/USA/AK-PHL8068/2021	EPI_ISL_1754873	4/9/2021	Alaska State Virology Laboratory	Alaska State Virology Laboratory	Stephanie DeRonde, Elva House, Lisa Smith, Ph.D., Jack Chen, Ph.D.
hCoV-19/USA/AK-PHL8083/2021	EPI_ISL_1754882	4/10/2021	Alaska State Virology Laboratory	Alaska State Virology Laboratory	Stephanie DeRonde, Elva House, Lisa Smith, Ph.D., Jack Chen, Ph.D.
hCoV-19/USA/AK-PHL8084/2021	EPI_ISL_1754883	4/6/2021	Alaska State Virology Laboratory	Alaska State Virology Laboratory	Stephanie DeRonde, Elva House, Lisa Smith, Ph.D., Jack Chen, Ph.D.
hCoV-19/USA/AK-PHL8086/2021	EPI_ISL_1754885	4/11/2021	Alaska State Virology Laboratory	Alaska State Virology Laboratory	Stephanie DeRonde, Elva House, Lisa Smith, Ph.D., Jack Chen, Ph.D.
hCoV-19/USA/AK-PHL8087/2021	EPI_ISL_1754886	4/13/2021	Alaska State Virology Laboratory	Alaska State Virology Laboratory	Stephanie DeRonde, Elva House, Lisa Smith, Ph.D., Jack Chen, Ph.D.
hCoV-19/USA/AK-PHL8091/2021	EPI_ISL_1754890	4/6/2021	Alaska State Virology Laboratory	Alaska State Virology Laboratory	Stephanie DeRonde, Elva House, Lisa Smith, Ph.D., Jack Chen, Ph.D.
hCoV-19/USA/AK-PHL8098/2021	EPI_ISL_1754893	4/11/2021	Alaska State Virology Laboratory	Alaska State Virology Laboratory	Stephanie DeRonde, Elva House, Lisa Smith, Ph.D., Jack Chen, Ph.D.
hCoV-19/USA/AK-CDC-ASC210041461/2021	EPI_ISL_1835211	4/1/2021	Aegis Sciences Corporation	Centers for Disease Control and Prevention Division of Viral Diseases, Pathogen Discovery	Dakota Howard, Dhwani Batra, Peter W. Cook, Kara Moser, Adrian Paskey, Jason Caravas, Benjamin Rambo-Martin, Shatavia Morrison, Christopher Gulvick, Scott Sammons, Yvette Unoarumhi, Darlene Wagner, Matthew Schmerer, Cyndi Clark, Patrick Campbell, Rob Case, Vikramsinha Ghorpade, Holly Houdeshell, Ola Kvalvaag, Dillon Nall, Ethan Sanders, Alec Vest, Shaun Westlund, Matthew Hardison, Clinton R. Paden, Duncan MacCannell
hCoV-19/USA/AK-CDC-ASC210041460/2021	EPI_ISL_1835210	4/1/2021	Aegis Sciences Corporation	Centers for Disease Control and Prevention Division of Viral Diseases, Pathogen Discovery	Dakota Howard, Dhwani Batra, Peter W. Cook, Kara Moser, Adrian Paskey, Jason Caravas, Benjamin Rambo-Martin, Shatavia Morrison, Christopher Gulvick, Scott Sammons, Yvette Unoarumhi, Darlene Wagner, Matthew Schmerer, Cyndi Clark, Patrick Campbell, Rob Case, Vikramsinha Ghorpade, Holly Houdeshell, Ola Kvalvaag, Dillon Nall, Ethan Sanders, Alec Vest, Shaun Westlund, Matthew Hardison, Clinton R. Paden, Duncan MacCannell
hCoV-19/USA/AK-PHL7583/2021	EPI_ISL_1483596	3/13/2021	Alaska State Virology Laboratory	Alaska State Virology Laboratory	Stephanie DeRonde, Elva House, Lisa Smith, Ph.D., Jack Chen, Ph.D.
hCoV-19/USA/AK-PHL7700/2021	EPI_ISL_1483656	3/20/2021	Alaska State Virology Laboratory	Alaska State Virology Laboratory	Stephanie DeRonde, Elva House, Lisa Smith, Ph.D., Jack Chen, Ph.D.
hCoV-19/USA/AK-PHL7751/2021	EPI_ISL_1483685	3/25/2021	Alaska State Virology Laboratory	Alaska State Virology Laboratory	Stephanie DeRonde, Elva House, Lisa Smith, Ph.D., Jack Chen, Ph.D.
hCoV-19/USA/AK-PHL7220/2021	EPI_ISL_1195944	2/16/2021	Alaska State Virology Laboratory	Alaska State Virology Laboratory	Stephanie DeRonde, Elva House, Lisa Smith, Ph.D., Jack Chen, Ph.D.
hCoV-19/USA/AK-PHL7358/2021	EPI_ISL_1195998	2/20/2021	Alaska State Virology Laboratory	Alaska State Virology Laboratory	Stephanie DeRonde, Elva House, Lisa Smith, Ph.D., Jack Chen, Ph.D.
hCoV-19/USA/AK-CDC-ASC210028420/2021	EPI_ISL_1687563	3/30/2021	Aegis Sciences Corporation	Centers for Disease Control and Prevention Division of Viral Diseases, Pathogen Discovery	Dakota Howard, Dhwani Batra, Peter W. Cook, Kara Moser, Adrian Paskey, Jason Caravas, Benjamin Rambo-Martin, Shatavia Morrison, Christopher Gulvick, Scott Sammons, Yvette Unoarumhi, Darlene Wagner, Matthew Schmerer, Cyndi Clark, Patrick Campbell, Rob Case, Vikramsinha Ghorpade, Holly Houdeshell, Ola Kvalvaag, Dillon Nall, Ethan Sanders, Alec Vest, Shaun Westlund, Matthew Hardison, Clinton R. Paden, Duncan MacCannell
hCoV-19/USA/AK-PHL8040/2021	EPI_ISL_1701698	3/26/2021	Alaska State Virology Laboratory	Alaska State Virology Laboratory	Stephanie DeRonde, Elva House, Lisa Smith, Ph.D., Jack Chen, Ph.D.
hCoV-19/USA/AK-PHL8018/2021	EPI_ISL_1701684	4/5/2021	Alaska State Virology Laboratory	Alaska State Virology Laboratory	Stephanie DeRonde, Elva House, Lisa Smith, Ph.D., Jack Chen, Ph.D.
hCoV-19/USA/AK-PHL8020/2021	EPI_ISL_1701686	4/5/2021	Alaska State Virology Laboratory	Alaska State Virology Laboratory	Stephanie DeRonde, Elva House, Lisa Smith, Ph.D., Jack Chen, Ph.D.
hCoV-19/USA/AK-PHL8022/2021	EPI_ISL_1701688	4/6/2021	Alaska State Virology Laboratory	Alaska State Virology Laboratory	Stephanie DeRonde, Elva House, Lisa Smith, Ph.D., Jack Chen, Ph.D.
hCoV-19/USA/AK-PHL8024/2021	EPI_ISL_1701690	4/6/2021	Alaska State Virology Laboratory	Alaska State Virology Laboratory	Stephanie DeRonde, Elva House, Lisa Smith, Ph.D., Jack Chen, Ph.D.
hCoV-19/USA/AK-PHL8028/2021	EPI_ISL_1701691	4/6/2021	Alaska State Virology Laboratory	Alaska State Virology Laboratory	Stephanie DeRonde, Elva House, Lisa Smith, Ph.D., Jack Chen, Ph.D.
hCoV-19/USA/AK-PHL8033/2021	EPI_ISL_1701694	3/4/2021	Alaska State Virology Laboratory	Alaska State Virology Laboratory	Stephanie DeRonde, Elva House, Lisa Smith, Ph.D., Jack Chen, Ph.D.
hCoV-19/USA/AK-PHL8035/2021	EPI_ISL_1701695	3/26/2021	Alaska State Virology Laboratory	Alaska State Virology Laboratory	Stephanie DeRonde, Elva House, Lisa Smith, Ph.D., Jack Chen, Ph.D.
hCoV-19/USA/AK-PHL8038/2021	EPI_ISL_1701697	3/26/2021	Alaska State Virology Laboratory	Alaska State Virology Laboratory	Stephanie DeRonde, Elva House, Lisa Smith, Ph.D., Jack Chen, Ph.D.
hCoV-19/USA/AK-PHL8049/2021	EPI_ISL_1701707	4/13/2021	Alaska State Virology Laboratory	Alaska State Virology Laboratory	Stephanie DeRonde, Elva House, Lisa Smith, Ph.D., Jack Chen, Ph.D.
hCoV-19/USA/AK-PHL8057/2021	EPI_ISL_1701714	4/14/2021	Alaska State Virology Laboratory	Alaska State Virology Laboratory	Stephanie DeRonde, Elva House, Lisa Smith, Ph.D., Jack Chen, Ph.D.

hCoV-19/USA/AK-CDC-ASC210046465/2021	EPI_ISL_1702140	4/4/2021	Aegis Sciences Corporation	Centers for Disease Control and Prevention Division of Viral Diseases, Pathogen Discovery	Dakota Howard, Dhwani Batra, Peter W. Cook, Kara Moser, Adrian Paskey, Jason Caravas, Benjamin Rambo-Martin, Shatavia Morrison, Christopher Gulvick, Scott Sammons, Yvette Unoarumhi, Darlene Wagner, Matthew Schmerer, Cyndi Clark, Patrick Campbell, Rob Case, Vikramsinha Ghorpade, Holly Houdeshell, Ola Kvalvaag, Dillon Nall, Ethan Sanders, Alec Vest, Shaun Westlund, Matthew Hardison, Clinton R. Paden, Duncan MacCannell
hCoV-19/USA/AK-PHL7735/2021	EPI_ISL_1483671	3/22/2021	Alaska State Virology Laboratory	Alaska State Virology Laboratory	Stephanie DeRonde, Elva House, Lisa Smith, Ph.D., Jack Chen, Ph.D.
hCoV-19/USA/AK-PHL7736/2021	EPI_ISL_1483672	3/22/2021	Alaska State Virology Laboratory	Alaska State Virology Laboratory	Stephanie DeRonde, Elva House, Lisa Smith, Ph.D., Jack Chen, Ph.D.
hCoV-19/USA/AK-PHL7574/2021	EPI_ISL_1483589	2/13/2021	Alaska State Virology Laboratory	Alaska State Virology Laboratory	Stephanie DeRonde, Elva House, Lisa Smith, Ph.D., Jack Chen, Ph.D.
hCoV-19/USA/AK-PHL7578/2021	EPI_ISL_1483592	2/13/2021	Alaska State Virology Laboratory	Alaska State Virology Laboratory	Stephanie DeRonde, Elva House, Lisa Smith, Ph.D., Jack Chen, Ph.D.
hCoV-19/USA/AK-PHL7579/2021	EPI_ISL_1483593	3/12/2021	Alaska State Virology Laboratory	Alaska State Virology Laboratory	Stephanie DeRonde, Elva House, Lisa Smith, Ph.D., Jack Chen, Ph.D.
hCoV-19/USA/AK-PHL7581/2021	EPI_ISL_1483594	3/2/2021	Alaska State Virology Laboratory	Alaska State Virology Laboratory	Stephanie DeRonde, Elva House, Lisa Smith, Ph.D., Jack Chen, Ph.D.
hCoV-19/USA/AK-PHL7582/2021	EPI_ISL_1483595	3/8/2021	Alaska State Virology Laboratory	Alaska State Virology Laboratory	Stephanie DeRonde, Elva House, Lisa Smith, Ph.D., Jack Chen, Ph.D.
hCoV-19/USA/AK-PHL7584/2021	EPI_ISL_1483597	3/10/2021	Alaska State Virology Laboratory	Alaska State Virology Laboratory	Stephanie DeRonde, Elva House, Lisa Smith, Ph.D., Jack Chen, Ph.D.
hCoV-19/USA/AK-PHL7586/2021	EPI_ISL_1483598	3/5/2021	Alaska State Virology Laboratory	Alaska State Virology Laboratory	Stephanie DeRonde, Elva House, Lisa Smith, Ph.D., Jack Chen, Ph.D.
hCoV-19/USA/AK-PHL7587/2021	EPI_ISL_1483599	3/5/2021	Alaska State Virology Laboratory	Alaska State Virology Laboratory	Stephanie DeRonde, Elva House, Lisa Smith, Ph.D., Jack Chen, Ph.D.
hCoV-19/USA/AK-PHL7589/2021	EPI_ISL_1483600	3/13/2021	Alaska State Virology Laboratory	Alaska State Virology Laboratory	Stephanie DeRonde, Elva House, Lisa Smith, Ph.D., Jack Chen, Ph.D.
hCoV-19/USA/AK-PHL7593/2021	EPI_ISL_1483604	3/10/2021	Alaska State Virology Laboratory	Alaska State Virology Laboratory	Stephanie DeRonde, Elva House, Lisa Smith, Ph.D., Jack Chen, Ph.D.
hCoV-19/USA/AK-PHL7594/2021	EPI_ISL_1483605	3/5/2021	Alaska State Virology Laboratory	Alaska State Virology Laboratory	Stephanie DeRonde, Elva House, Lisa Smith, Ph.D., Jack Chen, Ph.D.
hCoV-19/USA/AK-PHL7596/2021	EPI_ISL_1483606	3/5/2021	Alaska State Virology Laboratory	Alaska State Virology Laboratory	Stephanie DeRonde, Elva House, Lisa Smith, Ph.D., Jack Chen, Ph.D.
hCoV-19/USA/AK-PHL7597/2021	EPI_ISL_1483607	3/10/2021	Alaska State Virology Laboratory	Alaska State Virology Laboratory	Stephanie DeRonde, Elva House, Lisa Smith, Ph.D., Jack Chen, Ph.D.
hCoV-19/USA/AK-PHL7598/2021	EPI_ISL_1483608	3/10/2021	Alaska State Virology Laboratory	Alaska State Virology Laboratory	Stephanie DeRonde, Elva House, Lisa Smith, Ph.D., Jack Chen, Ph.D.
hCoV-19/USA/AK-PHL7599/2021	EPI_ISL_1483609	3/10/2021	Alaska State Virology Laboratory	Alaska State Virology Laboratory	Stephanie DeRonde, Elva House, Lisa Smith, Ph.D., Jack Chen, Ph.D.
hCoV-19/USA/AK-PHL7600/2021	EPI_ISL_1483610	3/11/2021	Alaska State Virology Laboratory	Alaska State Virology Laboratory	Stephanie DeRonde, Elva House, Lisa Smith, Ph.D., Jack Chen, Ph.D.
hCoV-19/USA/AK-PHL7601/2021	EPI_ISL_1483611	3/12/2021	Alaska State Virology Laboratory	Alaska State Virology Laboratory	Stephanie DeRonde, Elva House, Lisa Smith, Ph.D., Jack Chen, Ph.D.
hCoV-19/USA/AK-PHL7602/2021	EPI_ISL_1483612	3/13/2021	Alaska State Virology Laboratory	Alaska State Virology Laboratory	Stephanie DeRonde, Elva House, Lisa Smith, Ph.D., Jack Chen, Ph.D.
hCoV-19/USA/AK-PHL7603/2021	EPI_ISL_1483613	3/13/2021	Alaska State Virology Laboratory	Alaska State Virology Laboratory	Stephanie DeRonde, Elva House, Lisa Smith, Ph.D., Jack Chen, Ph.D.
hCoV-19/USA/AK-PHL7604/2021	EPI_ISL_1483614	3/13/2021	Alaska State Virology Laboratory	Alaska State Virology Laboratory	Stephanie DeRonde, Elva House, Lisa Smith, Ph.D., Jack Chen, Ph.D.
hCoV-19/USA/AK-PHL7605/2021	EPI_ISL_1483615	3/12/2021	Alaska State Virology Laboratory	Alaska State Virology Laboratory	Stephanie DeRonde, Elva House, Lisa Smith, Ph.D., Jack Chen, Ph.D.
hCoV-19/USA/AK-PHL7606/2021	EPI_ISL_1483616	3/9/2021	Alaska State Virology Laboratory	Alaska State Virology Laboratory	Stephanie DeRonde, Elva House, Lisa Smith, Ph.D., Jack Chen, Ph.D.
hCoV-19/USA/AK-PHL7607/2021	EPI_ISL_1483617	3/10/2021	Alaska State Virology Laboratory	Alaska State Virology Laboratory	Stephanie DeRonde, Elva House, Lisa Smith, Ph.D., Jack Chen, Ph.D.
hCoV-19/USA/AK-PHL7608/2021	EPI_ISL_1483618	3/11/2021	Alaska State Virology Laboratory	Alaska State Virology Laboratory	Stephanie DeRonde, Elva House, Lisa Smith, Ph.D., Jack Chen, Ph.D.
hCoV-19/USA/AK-PHL7609/2021	EPI_ISL_1483619	3/10/2021	Alaska State Virology Laboratory	Alaska State Virology Laboratory	Stephanie DeRonde, Elva House, Lisa Smith, Ph.D., Jack Chen, Ph.D.
hCoV-19/USA/AK-PHL7610/2021	EPI_ISL_1483620	3/9/2021	Alaska State Virology Laboratory	Alaska State Virology Laboratory	Stephanie DeRonde, Elva House, Lisa Smith, Ph.D., Jack Chen, Ph.D.
hCoV-19/USA/AK-PHL7659/2021	EPI_ISL_1483621	3/21/2021	Alaska State Virology Laboratory	Alaska State Virology Laboratory	Stephanie DeRonde, Elva House, Lisa Smith, Ph.D., Jack Chen, Ph.D.
hCoV-19/USA/AK-PHL7703/2021	EPI_ISL_1483622	3/21/2021	Alaska State Virology Laboratory	Alaska State Virology Laboratory	Stephanie DeRonde, Elva House, Lisa Smith, Ph.D., Jack Chen, Ph.D.
hCoV-19/USA/AK-PHL7660/2021	EPI_ISL_1483623	3/20/2021	Alaska State Virology Laboratory	Alaska State Virology Laboratory	Stephanie DeRonde, Elva House, Lisa Smith, Ph.D., Jack Chen, Ph.D.
hCoV-19/USA/AK-PHL7661/2021	EPI_ISL_1483624	3/20/2021	Alaska State Virology Laboratory	Alaska State Virology Laboratory	Stephanie DeRonde, Elva House, Lisa Smith, Ph.D., Jack Chen, Ph.D.
hCoV-19/USA/AK-PHL7662/2021	EPI_ISL_1483625	3/21/2021	Alaska State Virology Laboratory	Alaska State Virology Laboratory	Stephanie DeRonde, Elva House, Lisa Smith, Ph.D., Jack Chen, Ph.D.
hCoV-19/USA/AK-PHL7663/2021	EPI_ISL_1483626	3/17/2021	Alaska State Virology Laboratory	Alaska State Virology Laboratory	Stephanie DeRonde, Elva House, Lisa Smith, Ph.D., Jack Chen, Ph.D.
hCoV-19/USA/AK-PHL7664/2021	EPI_ISL_1483627	3/17/2021	Alaska State Virology Laboratory	Alaska State Virology Laboratory	Stephanie DeRonde, Elva House, Lisa Smith, Ph.D., Jack Chen, Ph.D.
hCoV-19/USA/AK-PHL7667/2021	EPI_ISL_1483628	3/17/2021	Alaska State Virology Laboratory	Alaska State Virology Laboratory	Stephanie DeRonde, Elva House, Lisa Smith, Ph.D., Jack Chen, Ph.D.
hCoV-19/USA/AK-PHL7668/2021	EPI_ISL_1483629	3/17/2021	Alaska State Virology Laboratory	Alaska State Virology Laboratory	Stephanie DeRonde, Elva House, Lisa Smith, Ph.D., Jack Chen, Ph.D.
hCoV-19/USA/AK-PHL7669/2021	EPI_ISL_1483630	3/17/2021	Alaska State Virology Laboratory	Alaska State Virology Laboratory	Stephanie DeRonde, Elva House, Lisa Smith, Ph.D., Jack Chen, Ph.D.
hCoV-19/USA/AK-PHL7670/2021	EPI_ISL_1483631	3/17/2021	Alaska State Virology Laboratory	Alaska State Virology Laboratory	Stephanie DeRonde, Elva House, Lisa Smith, Ph.D., Jack Chen, Ph.D.
hCoV-19/USA/AK-PHL7671/2021	EPI_ISL_1483632	3/19/2021	Alaska State Virology Laboratory	Alaska State Virology Laboratory	Stephanie DeRonde, Elva House, Lisa Smith, Ph.D., Jack Chen, Ph.D.
hCoV-19/USA/AK-PHL7672/2021	EPI_ISL_1483633	3/19/2021	Alaska State Virology Laboratory	Alaska State Virology Laboratory	Stephanie DeRonde, Elva House, Lisa Smith, Ph.D., Jack Chen, Ph.D.
hCoV-19/USA/AK-PHL7673/2021	EPI_ISL_1483634	3/21/2021	Alaska State Virology Laboratory	Alaska State Virology Laboratory	Stephanie DeRonde, Elva House, Lisa Smith, Ph.D., Jack Chen, Ph.D.
hCoV-19/USA/AK-PHL7674/2021	EPI_ISL_1483635	3/20/2021	Alaska State Virology Laboratory	Alaska State Virology Laboratory	Stephanie DeRonde, Elva House, Lisa Smith, Ph.D., Jack Chen, Ph.D.
hCoV-19/USA/AK-PHL7679/2021	EPI_ISL_1483636	3/16/2021	Alaska State Virology Laboratory	Alaska State Virology Laboratory	Stephanie DeRonde, Elva House, Lisa Smith, Ph.D., Jack Chen, Ph.D.
hCoV-19/USA/AK-PHL7680/2021	EPI_ISL_1483637	3/18/2021	Alaska State Virology Laboratory	Alaska State Virology Laboratory	Stephanie DeRonde, Elva House, Lisa Smith, Ph.D., Jack Chen, Ph.D.

hCoV-19/USA/AK-PHL7681/2021	EPI_ISL_1483638	3/17/2021	Alaska State Virology Laboratory	Alaska State Virology Laboratory	Stephanie DeRonde, Elva House, Lisa Smith, Ph.D., Jack Chen, Ph.D.
hCoV-19/USA/AK-PHL7682/2021	EPI_ISL_1483639	3/16/2021	Alaska State Virology Laboratory	Alaska State Virology Laboratory	Stephanie DeRonde, Elva House, Lisa Smith, Ph.D., Jack Chen, Ph.D.
hCoV-19/USA/AK-PHL7683/2021	EPI_ISL_1483640	3/17/2021	Alaska State Virology Laboratory	Alaska State Virology Laboratory	Stephanie DeRonde, Elva House, Lisa Smith, Ph.D., Jack Chen, Ph.D.
hCoV-19/USA/AK-PHL7684/2021	EPI_ISL_1483641	3/19/2021	Alaska State Virology Laboratory	Alaska State Virology Laboratory	Stephanie DeRonde, Elva House, Lisa Smith, Ph.D., Jack Chen, Ph.D.
hCoV-19/USA/AK-PHL7685/2021	EPI_ISL_1483642	3/18/2021	Alaska State Virology Laboratory	Alaska State Virology Laboratory	Stephanie DeRonde, Elva House, Lisa Smith, Ph.D., Jack Chen, Ph.D.
hCoV-19/USA/AK-PHL7686/2021	EPI_ISL_1483643	3/20/2021	Alaska State Virology Laboratory	Alaska State Virology Laboratory	Stephanie DeRonde, Elva House, Lisa Smith, Ph.D., Jack Chen, Ph.D.
hCoV-19/USA/AK-PHL7687/2021	EPI_ISL_1483644	3/20/2021	Alaska State Virology Laboratory	Alaska State Virology Laboratory	Stephanie DeRonde, Elva House, Lisa Smith, Ph.D., Jack Chen, Ph.D.
hCoV-19/USA/AK-PHL7688/2021	EPI_ISL_1483645	3/20/2021	Alaska State Virology Laboratory	Alaska State Virology Laboratory	Stephanie DeRonde, Elva House, Lisa Smith, Ph.D., Jack Chen, Ph.D.
hCoV-19/USA/AK-PHL7689/2021	EPI_ISL_1483646	3/21/2021	Alaska State Virology Laboratory	Alaska State Virology Laboratory	Stephanie DeRonde, Elva House, Lisa Smith, Ph.D., Jack Chen, Ph.D.
hCoV-19/USA/AK-PHL7690/2021	EPI_ISL_1483647	3/19/2021	Alaska State Virology Laboratory	Alaska State Virology Laboratory	Stephanie DeRonde, Elva House, Lisa Smith, Ph.D., Jack Chen, Ph.D.
hCoV-19/USA/AK-PHL7691/2021	EPI_ISL_1483648	3/20/2021	Alaska State Virology Laboratory	Alaska State Virology Laboratory	Stephanie DeRonde, Elva House, Lisa Smith, Ph.D., Jack Chen, Ph.D.
hCoV-19/USA/AK-PHL7692/2021	EPI_ISL_1483649	3/20/2021	Alaska State Virology Laboratory	Alaska State Virology Laboratory	Stephanie DeRonde, Elva House, Lisa Smith, Ph.D., Jack Chen, Ph.D.
hCoV-19/USA/AK-PHL7694/2021	EPI_ISL_1483650	3/21/2021	Alaska State Virology Laboratory	Alaska State Virology Laboratory	Stephanie DeRonde, Elva House, Lisa Smith, Ph.D., Jack Chen, Ph.D.
hCoV-19/USA/AK-PHL7695/2021	EPI_ISL_1483651	3/20/2021	Alaska State Virology Laboratory	Alaska State Virology Laboratory	Stephanie DeRonde, Elva House, Lisa Smith, Ph.D., Jack Chen, Ph.D.
hCoV-19/USA/AK-PHL7696/2021	EPI_ISL_1483652	3/20/2021	Alaska State Virology Laboratory	Alaska State Virology Laboratory	Stephanie DeRonde, Elva House, Lisa Smith, Ph.D., Jack Chen, Ph.D.
hCoV-19/USA/AK-PHL7697/2021	EPI_ISL_1483653	3/20/2021	Alaska State Virology Laboratory	Alaska State Virology Laboratory	Stephanie DeRonde, Elva House, Lisa Smith, Ph.D., Jack Chen, Ph.D.
hCoV-19/USA/AK-PHL7698/2021	EPI_ISL_1483654	3/20/2021	Alaska State Virology Laboratory	Alaska State Virology Laboratory	Stephanie DeRonde, Elva House, Lisa Smith, Ph.D., Jack Chen, Ph.D.
hCoV-19/USA/AK-PHL7699/2021	EPI_ISL_1483655	3/19/2021	Alaska State Virology Laboratory	Alaska State Virology Laboratory	Stephanie DeRonde, Elva House, Lisa Smith, Ph.D., Jack Chen, Ph.D.
hCoV-19/USA/AK-PHL7702/2021	EPI_ISL_1483658	3/20/2021	Alaska State Virology Laboratory	Alaska State Virology Laboratory	Stephanie DeRonde, Elva House, Lisa Smith, Ph.D., Jack Chen, Ph.D.
hCoV-19/USA/AK-PHL7721/2021	EPI_ISL_1483659	3/22/2021	Alaska State Virology Laboratory	Alaska State Virology Laboratory	Stephanie DeRonde, Elva House, Lisa Smith, Ph.D., Jack Chen, Ph.D.
hCoV-19/USA/AK-PHL7723/2021	EPI_ISL_1483660	3/25/2021	Alaska State Virology Laboratory	Alaska State Virology Laboratory	Stephanie DeRonde, Elva House, Lisa Smith, Ph.D., Jack Chen, Ph.D.
hCoV-19/USA/AK-PHL7724/2021	EPI_ISL_1483661	3/24/2021	Alaska State Virology Laboratory	Alaska State Virology Laboratory	Stephanie DeRonde, Elva House, Lisa Smith, Ph.D., Jack Chen, Ph.D.
hCoV-19/USA/AK-PHL7725/2021	EPI_ISL_1483662	3/2/2021	Alaska State Virology Laboratory	Alaska State Virology Laboratory	Stephanie DeRonde, Elva House, Lisa Smith, Ph.D., Jack Chen, Ph.D.
hCoV-19/USA/AK-PHL7727/2021	EPI_ISL_1483664	3/24/2021	Alaska State Virology Laboratory	Alaska State Virology Laboratory	Stephanie DeRonde, Elva House, Lisa Smith, Ph.D., Jack Chen, Ph.D.
hCoV-19/USA/AK-PHL7728/2021	EPI_ISL_1483665	3/24/2021	Alaska State Virology Laboratory	Alaska State Virology Laboratory	Stephanie DeRonde, Elva House, Lisa Smith, Ph.D., Jack Chen, Ph.D.
hCoV-19/USA/AK-PHL7729/2021	EPI_ISL_1483666	3/24/2021	Alaska State Virology Laboratory	Alaska State Virology Laboratory	Stephanie DeRonde, Elva House, Lisa Smith, Ph.D., Jack Chen, Ph.D.
hCoV-19/USA/AK-PHL7730/2021	EPI_ISL_1483667	3/24/2021	Alaska State Virology Laboratory	Alaska State Virology Laboratory	Stephanie DeRonde, Elva House, Lisa Smith, Ph.D., Jack Chen, Ph.D.
hCoV-19/USA/AK-PHL7731/2021	EPI_ISL_1483668	3/19/2021	Alaska State Virology Laboratory	Alaska State Virology Laboratory	Stephanie DeRonde, Elva House, Lisa Smith, Ph.D., Jack Chen, Ph.D.
hCoV-19/USA/AK-PHL7733/2021	EPI_ISL_1483669	3/25/2021	Alaska State Virology Laboratory	Alaska State Virology Laboratory	Stephanie DeRonde, Elva House, Lisa Smith, Ph.D., Jack Chen, Ph.D.
hCoV-19/USA/AK-PHL7734/2021	EPI_ISL_1483670	3/22/2021	Alaska State Virology Laboratory	Alaska State Virology Laboratory	Stephanie DeRonde, Elva House, Lisa Smith, Ph.D., Jack Chen, Ph.D.
hCoV-19/USA/AK-PHL7737/2021	EPI_ISL_1483673	3/23/2021	Alaska State Virology Laboratory	Alaska State Virology Laboratory	Stephanie DeRonde, Elva House, Lisa Smith, Ph.D., Jack Chen, Ph.D.
hCoV-19/USA/AK-PHL7738/2021	EPI_ISL_1483674	3/23/2021	Alaska State Virology Laboratory	Alaska State Virology Laboratory	Stephanie DeRonde, Elva House, Lisa Smith, Ph.D., Jack Chen, Ph.D.
hCoV-19/USA/AK-PHL7740/2021	EPI_ISL_1483676	3/26/2021	Alaska State Virology Laboratory	Alaska State Virology Laboratory	Stephanie DeRonde, Elva House, Lisa Smith, Ph.D., Jack Chen, Ph.D.
hCoV-19/USA/AK-PHL7747/2021	EPI_ISL_1483681	3/26/2021	Alaska State Virology Laboratory	Alaska State Virology Laboratory	Stephanie DeRonde, Elva House, Lisa Smith, Ph.D., Jack Chen, Ph.D.
hCoV-19/USA/AK-PHL7748/2021	EPI_ISL_1483682	3/25/2021	Alaska State Virology Laboratory	Alaska State Virology Laboratory	Stephanie DeRonde, Elva House, Lisa Smith, Ph.D., Jack Chen, Ph.D.
hCoV-19/USA/AK-PHL7749/2021	EPI_ISL_1483683	3/26/2021	Alaska State Virology Laboratory	Alaska State Virology Laboratory	Stephanie DeRonde, Elva House, Lisa Smith, Ph.D., Jack Chen, Ph.D.
hCoV-19/USA/AK-PHL7750/2021	EPI_ISL_1483684	3/26/2021	Alaska State Virology Laboratory	Alaska State Virology Laboratory	Stephanie DeRonde, Elva House, Lisa Smith, Ph.D., Jack Chen, Ph.D.
hCoV-19/USA/AK-PHL7755/2021	EPI_ISL_1483689	3/25/2021	Alaska State Virology Laboratory	Alaska State Virology Laboratory	Stephanie DeRonde, Elva House, Lisa Smith, Ph.D., Jack Chen, Ph.D.
hCoV-19/USA/AK-PHL7756/2021	EPI_ISL_1483690	3/26/2021	Alaska State Virology Laboratory	Alaska State Virology Laboratory	Stephanie DeRonde, Elva House, Lisa Smith, Ph.D., Jack Chen, Ph.D.
hCoV-19/USA/AK-PHL7757/2021	EPI_ISL_1483691	3/26/2021	Alaska State Virology Laboratory	Alaska State Virology Laboratory	Stephanie DeRonde, Elva House, Lisa Smith, Ph.D., Jack Chen, Ph.D.
hCoV-19/USA/AK-PHL7758/2021	EPI_ISL_1483692	3/25/2021	Alaska State Virology Laboratory	Alaska State Virology Laboratory	Stephanie DeRonde, Elva House, Lisa Smith, Ph.D., Jack Chen, Ph.D.
hCoV-19/USA/AK-PHL7759/2021	EPI_ISL_1483693	3/25/2021	Alaska State Virology Laboratory	Alaska State Virology Laboratory	Stephanie DeRonde, Elva House, Lisa Smith, Ph.D., Jack Chen, Ph.D.
hCoV-19/USA/AK-PHL7760/2021	EPI_ISL_1483694	3/25/2021	Alaska State Virology Laboratory	Alaska State Virology Laboratory	Stephanie DeRonde, Elva House, Lisa Smith, Ph.D., Jack Chen, Ph.D.
hCoV-19/USA/AK-PHL7761/2021	EPI_ISL_1483695	3/25/2021	Alaska State Virology Laboratory	Alaska State Virology Laboratory	Stephanie DeRonde, Elva House, Lisa Smith, Ph.D., Jack Chen, Ph.D.
hCoV-19/USA/AK-PHL7762/2021	EPI_ISL_1483696	3/26/2021	Alaska State Virology Laboratory	Alaska State Virology Laboratory	Stephanie DeRonde, Elva House, Lisa Smith, Ph.D., Jack Chen, Ph.D.
hCoV-19/USA/AK-PHL7763/2021	EPI_ISL_1483697	3/26/2021	Alaska State Virology Laboratory	Alaska State Virology Laboratory	Stephanie DeRonde, Elva House, Lisa Smith, Ph.D., Jack Chen, Ph.D.
hCoV-19/USA/AK-PHL7765/2021	EPI_ISL_1483698	3/26/2021	Alaska State Virology Laboratory	Alaska State Virology Laboratory	Stephanie DeRonde, Elva House, Lisa Smith, Ph.D., Jack Chen, Ph.D.
hCoV-19/USA/AK-PHL7575/2021	EPI_ISL_1483590	2/4/2021	Alaska State Virology Laboratory	Alaska State Virology Laboratory	Stephanie DeRonde, Elva House, Lisa Smith, Ph.D., Jack Chen, Ph.D.
hCoV-19/USA/AK-PHL7576/2021	EPI_ISL_1483591	2/22/2021	Alaska State Virology Laboratory	Alaska State Virology Laboratory	Stephanie DeRonde, Elva House, Lisa Smith, Ph.D., Jack Chen, Ph.D.
hCoV-19/USA/AK-PHL7590/2021	EPI_ISL_1483601	3/13/2021	Alaska State Virology Laboratory	Alaska State Virology Laboratory	Stephanie DeRonde, Elva House, Lisa Smith, Ph.D., Jack Chen, Ph.D.
hCoV-19/USA/AK-PHL7591/2021	EPI_ISL_1483602	3/13/2021	Alaska State Virology Laboratory	Alaska State Virology Laboratory	Stephanie DeRonde, Elva House, Lisa Smith, Ph.D., Jack Chen, Ph.D.

hCoV-19/USA/AK-PHL7592/2021	EPI_ISL_1483603	3/11/2021	Alaska State Virology Laboratory	Alaska State Virology Laboratory	Stephanie DeRonde, Elva House, Lisa Smith, Ph.D., Jack Chen, Ph.D.
hCoV-19/USA/AK-PHL7701/2021	EPI_ISL_1483657	3/21/2021	Alaska State Virology Laboratory	Alaska State Virology Laboratory	Stephanie DeRonde, Elva House, Lisa Smith, Ph.D., Jack Chen, Ph.D.
hCoV-19/USA/AK-PHL7726/2021	EPI_ISL_1483663	3/23/2021	Alaska State Virology Laboratory	Alaska State Virology Laboratory	Stephanie DeRonde, Elva House, Lisa Smith, Ph.D., Jack Chen, Ph.D.
hCoV-19/USA/AK-PHL7739/2021	EPI_ISL_1483675	3/26/2021	Alaska State Virology Laboratory	Alaska State Virology Laboratory	Stephanie DeRonde, Elva House, Lisa Smith, Ph.D., Jack Chen, Ph.D.
hCoV-19/USA/AK-PHL7743/2021	EPI_ISL_1483677	3/26/2021	Alaska State Virology Laboratory	Alaska State Virology Laboratory	Stephanie DeRonde, Elva House, Lisa Smith, Ph.D., Jack Chen, Ph.D.
hCoV-19/USA/AK-PHL7744/2021	EPI_ISL_1483678	3/25/2021	Alaska State Virology Laboratory	Alaska State Virology Laboratory	Stephanie DeRonde, Elva House, Lisa Smith, Ph.D., Jack Chen, Ph.D.
hCoV-19/USA/AK-PHL7745/2021	EPI_ISL_1483679	3/26/2021	Alaska State Virology Laboratory	Alaska State Virology Laboratory	Stephanie DeRonde, Elva House, Lisa Smith, Ph.D., Jack Chen, Ph.D.
hCoV-19/USA/AK-PHL7746/2021	EPI_ISL_1483680	3/26/2021	Alaska State Virology Laboratory	Alaska State Virology Laboratory	Stephanie DeRonde, Elva House, Lisa Smith, Ph.D., Jack Chen, Ph.D.
hCoV-19/USA/AK-PHL7752/2021	EPI_ISL_1483686	3/26/2021	Alaska State Virology Laboratory	Alaska State Virology Laboratory	Stephanie DeRonde, Elva House, Lisa Smith, Ph.D., Jack Chen, Ph.D.
hCoV-19/USA/AK-PHL7753/2021	EPI_ISL_1483687	3/26/2021	Alaska State Virology Laboratory	Alaska State Virology Laboratory	Stephanie DeRonde, Elva House, Lisa Smith, Ph.D., Jack Chen, Ph.D.
hCoV-19/USA/AK-PHL7754/2021	EPI_ISL_1483688	3/26/2021	Alaska State Virology Laboratory	Alaska State Virology Laboratory	Stephanie DeRonde, Elva House, Lisa Smith, Ph.D., Jack Chen, Ph.D.
hCoV-19/USA/AK-PHL7766/2021	EPI_ISL_1483699	3/26/2021	Alaska State Virology Laboratory	Alaska State Virology Laboratory	Stephanie DeRonde, Elva House, Lisa Smith, Ph.D., Jack Chen, Ph.D.
hCoV-19/USA/AK-PHL7612/2021	EPI_ISL_1529075	3/11/2021	Alaska State Virology Laboratory	Alaska State Virology Laboratory	Stephanie DeRonde, Elva House, Lisa Smith, Ph.D., Jack Chen, Ph.D.
hCoV-19/USA/AK-PHL7613/2021	EPI_ISL_1529076	3/12/2021	Alaska State Virology Laboratory	Alaska State Virology Laboratory	Stephanie DeRonde, Elva House, Lisa Smith, Ph.D., Jack Chen, Ph.D.
hCoV-19/USA/AK-PHL7614/2021	EPI_ISL_1529077	3/15/2021	Alaska State Virology Laboratory	Alaska State Virology Laboratory	Stephanie DeRonde, Elva House, Lisa Smith, Ph.D., Jack Chen, Ph.D.
hCoV-19/USA/AK-PHL7615/2021	EPI_ISL_1529078	3/13/2021	Alaska State Virology Laboratory	Alaska State Virology Laboratory	Stephanie DeRonde, Elva House, Lisa Smith, Ph.D., Jack Chen, Ph.D.
hCoV-19/USA/AK-PHL7616/2021	EPI_ISL_1529079	3/13/2021	Alaska State Virology Laboratory	Alaska State Virology Laboratory	Stephanie DeRonde, Elva House, Lisa Smith, Ph.D., Jack Chen, Ph.D.
hCoV-19/USA/AK-PHL7619/2021	EPI_ISL_1529080	3/13/2021	Alaska State Virology Laboratory	Alaska State Virology Laboratory	Stephanie DeRonde, Elva House, Lisa Smith, Ph.D., Jack Chen, Ph.D.
hCoV-19/USA/AK-PHL7620/2021	EPI_ISL_1529081	3/14/2021	Alaska State Virology Laboratory	Alaska State Virology Laboratory	Stephanie DeRonde, Elva House, Lisa Smith, Ph.D., Jack Chen, Ph.D.
hCoV-19/USA/AK-PHL7621/2021	EPI_ISL_1529082	3/14/2021	Alaska State Virology Laboratory	Alaska State Virology Laboratory	Stephanie DeRonde, Elva House, Lisa Smith, Ph.D., Jack Chen, Ph.D.
hCoV-19/USA/AK-PHL7622/2021	EPI_ISL_1529083	3/14/2021	Alaska State Virology Laboratory	Alaska State Virology Laboratory	Stephanie DeRonde, Elva House, Lisa Smith, Ph.D., Jack Chen, Ph.D.
hCoV-19/USA/AK-PHL7623/2021	EPI_ISL_1529084	3/14/2021	Alaska State Virology Laboratory	Alaska State Virology Laboratory	Stephanie DeRonde, Elva House, Lisa Smith, Ph.D., Jack Chen, Ph.D.
hCoV-19/USA/AK-PHL7628/2021	EPI_ISL_1529085	3/15/2021	Alaska State Virology Laboratory	Alaska State Virology Laboratory	Stephanie DeRonde, Elva House, Lisa Smith, Ph.D., Jack Chen, Ph.D.
hCoV-19/USA/AK-PHL7629/2021	EPI_ISL_1529086	3/16/2021	Alaska State Virology Laboratory	Alaska State Virology Laboratory	Stephanie DeRonde, Elva House, Lisa Smith, Ph.D., Jack Chen, Ph.D.
hCoV-19/USA/AK-PHL7630/2021	EPI_ISL_1529087	3/16/2021	Alaska State Virology Laboratory	Alaska State Virology Laboratory	Stephanie DeRonde, Elva House, Lisa Smith, Ph.D., Jack Chen, Ph.D.
hCoV-19/USA/AK-PHL7633/2021	EPI_ISL_1529089	3/16/2021	Alaska State Virology Laboratory	Alaska State Virology Laboratory	Stephanie DeRonde, Elva House, Lisa Smith, Ph.D., Jack Chen, Ph.D.
hCoV-19/USA/AK-PHL7634/2021	EPI_ISL_1529090	3/9/2021	Alaska State Virology Laboratory	Alaska State Virology Laboratory	Stephanie DeRonde, Elva House, Lisa Smith, Ph.D., Jack Chen, Ph.D.
hCoV-19/USA/AK-PHL7636/2021	EPI_ISL_1529091	3/15/2021	Alaska State Virology Laboratory	Alaska State Virology Laboratory	Stephanie DeRonde, Elva House, Lisa Smith, Ph.D., Jack Chen, Ph.D.
hCoV-19/USA/AK-PHL7637/2021	EPI_ISL_1529092	3/16/2021	Alaska State Virology Laboratory	Alaska State Virology Laboratory	Stephanie DeRonde, Elva House, Lisa Smith, Ph.D., Jack Chen, Ph.D.
hCoV-19/USA/AK-PHL7638/2021	EPI_ISL_1529093	3/15/2021	Alaska State Virology Laboratory	Alaska State Virology Laboratory	Stephanie DeRonde, Elva House, Lisa Smith, Ph.D., Jack Chen, Ph.D.
hCoV-19/USA/AK-PHL7639/2021	EPI_ISL_1529094	3/15/2021	Alaska State Virology Laboratory	Alaska State Virology Laboratory	Stephanie DeRonde, Elva House, Lisa Smith, Ph.D., Jack Chen, Ph.D.
hCoV-19/USA/AK-PHL7650/2021	EPI_ISL_1529095	3/16/2021	Alaska State Virology Laboratory	Alaska State Virology Laboratory	Stephanie DeRonde, Elva House, Lisa Smith, Ph.D., Jack Chen, Ph.D.
hCoV-19/USA/AK-PHL7651/2021	EPI_ISL_1529096	3/15/2021	Alaska State Virology Laboratory	Alaska State Virology Laboratory	Stephanie DeRonde, Elva House, Lisa Smith, Ph.D., Jack Chen, Ph.D.
hCoV-19/USA/AK-PHL7652/2021	EPI_ISL_1529097	3/15/2021	Alaska State Virology Laboratory	Alaska State Virology Laboratory	Stephanie DeRonde, Elva House, Lisa Smith, Ph.D., Jack Chen, Ph.D.
hCoV-19/USA/AK-PHL7655/2021	EPI_ISL_1529098	3/14/2021	Alaska State Virology Laboratory	Alaska State Virology Laboratory	Stephanie DeRonde, Elva House, Lisa Smith, Ph.D., Jack Chen, Ph.D.
hCoV-19/USA/AK-6544/2020	EPI_ISL_884222	11/30/2020	Alaska Department of Health, Division of Public Health, Section of Laboratories	Alaska Department of Health, Division of Public Health, Section of Laboratories	Stephanie DeRonde, Lisa Smith PhD, Jack Chen PhD
hCoV-19/USA/AK-PHL7822/2021	EPI_ISL_1575044	4/1/2021	Alaska State Virology Laboratory	Alaska State Virology Laboratory	Stephanie DeRonde, Elva House, Lisa Smith, Ph.D., Jack Chen, Ph.D.
hCoV-19/USA/AK-PHL7823/2021	EPI_ISL_1575045	3/29/2021	Alaska State Virology Laboratory	Alaska State Virology Laboratory	Stephanie DeRonde, Elva House, Lisa Smith, Ph.D., Jack Chen, Ph.D.
hCoV-19/USA/AK-PHL7829/2021	EPI_ISL_1575050	3/30/2021	Alaska State Virology Laboratory	Alaska State Virology Laboratory	Stephanie DeRonde, Elva House, Lisa Smith, Ph.D., Jack Chen, Ph.D.
hCoV-19/USA/AK-PHL7830/2021	EPI_ISL_1575051	3/30/2021	Alaska State Virology Laboratory	Alaska State Virology Laboratory	Stephanie DeRonde, Elva House, Lisa Smith, Ph.D., Jack Chen, Ph.D.
hCoV-19/USA/AK-PHL7831/2021	EPI_ISL_1575052	3/30/2021	Alaska State Virology Laboratory	Alaska State Virology Laboratory	Stephanie DeRonde, Elva House, Lisa Smith, Ph.D., Jack Chen, Ph.D.
hCoV-19/USA/AK-PHL7833/2021	EPI_ISL_1575054	3/30/2021	Alaska State Virology Laboratory	Alaska State Virology Laboratory	Stephanie DeRonde, Elva House, Lisa Smith, Ph.D., Jack Chen, Ph.D.
hCoV-19/USA/AK-PHL7834/2021	EPI_ISL_1575055	3/30/2021	Alaska State Virology Laboratory	Alaska State Virology Laboratory	Stephanie DeRonde, Elva House, Lisa Smith, Ph.D., Jack Chen, Ph.D.
hCoV-19/USA/AK-PHL7836/2021	EPI_ISL_1575057	3/30/2021	Alaska State Virology Laboratory	Alaska State Virology Laboratory	Stephanie DeRonde, Elva House, Lisa Smith, Ph.D., Jack Chen, Ph.D.
hCoV-19/USA/AK-PHL7839/2021	EPI_ISL_1575060	3/25/2021	Alaska State Virology Laboratory	Alaska State Virology Laboratory	Stephanie DeRonde, Elva House, Lisa Smith, Ph.D., Jack Chen, Ph.D.
hCoV-19/USA/AK-PHL7840/2021	EPI_ISL_1575061	3/29/2021	Alaska State Virology Laboratory	Alaska State Virology Laboratory	Stephanie DeRonde, Elva House, Lisa Smith, Ph.D., Jack Chen, Ph.D.
hCoV-19/USA/AK-PHL7844/2021	EPI_ISL_1575065	3/31/2021	Alaska State Virology Laboratory	Alaska State Virology Laboratory	Stephanie DeRonde, Elva House, Lisa Smith, Ph.D., Jack Chen, Ph.D.
hCoV-19/USA/AK-PHL7846/2021	EPI_ISL_1575067	3/30/2021	Alaska State Virology Laboratory	Alaska State Virology Laboratory	Stephanie DeRonde, Elva House, Lisa Smith, Ph.D., Jack Chen, Ph.D.
hCoV-19/USA/AK-PHL7847/2021	EPI_ISL_1575068	3/30/2021	Alaska State Virology Laboratory	Alaska State Virology Laboratory	Stephanie DeRonde, Elva House, Lisa Smith, Ph.D., Jack Chen, Ph.D.
hCoV-19/USA/AK-PHL7849/2021	EPI_ISL_1575070	3/30/2021	Alaska State Virology Laboratory	Alaska State Virology Laboratory	Stephanie DeRonde, Elva House, Lisa Smith, Ph.D., Jack Chen, Ph.D.
hCoV-19/USA/AK-PHL7851/2021	EPI_ISL_1575072	3/30/2021	Alaska State Virology Laboratory	Alaska State Virology Laboratory	Stephanie DeRonde, Elva House, Lisa Smith, Ph.D., Jack Chen, Ph.D.

hCoV-19/USA/AK-PHL7852/2021	EPI_ISL_1575073	3/30/2021	Alaska State Virology Laboratory	Alaska State Virology Laboratory	Stephanie DeRonde, Elva House, Lisa Smith, Ph.D., Jack Chen, Ph.D.
hCoV-19/USA/AK-PHL7814/2021	EPI_ISL_1575037	3/30/2021	Alaska State Virology Laboratory	Alaska State Virology Laboratory	Stephanie DeRonde, Elva House, Lisa Smith, Ph.D., Jack Chen, Ph.D.
hCoV-19/USA/AK-PHL7815/2021	EPI_ISL_1575038	3/30/2021	Alaska State Virology Laboratory	Alaska State Virology Laboratory	Stephanie DeRonde, Elva House, Lisa Smith, Ph.D., Jack Chen, Ph.D.
hCoV-19/USA/AK-PHL7816/2021	EPI_ISL_1575039	3/31/2021	Alaska State Virology Laboratory	Alaska State Virology Laboratory	Stephanie DeRonde, Elva House, Lisa Smith, Ph.D., Jack Chen, Ph.D.
hCoV-19/USA/AK-PHL7819/2021	EPI_ISL_1575041	3/28/2021	Alaska State Virology Laboratory	Alaska State Virology Laboratory	Stephanie DeRonde, Elva House, Lisa Smith, Ph.D., Jack Chen, Ph.D.
hCoV-19/USA/AK-PHL7820/2021	EPI_ISL_1575042	3/31/2021	Alaska State Virology Laboratory	Alaska State Virology Laboratory	Stephanie DeRonde, Elva House, Lisa Smith, Ph.D., Jack Chen, Ph.D.
hCoV-19/USA/AK-PHL7855/2021	EPI_ISL_1575076	3/30/2021	Alaska State Virology Laboratory	Alaska State Virology Laboratory	Stephanie DeRonde, Elva House, Lisa Smith, Ph.D., Jack Chen, Ph.D.
hCoV-19/USA/AK-PHL7856/2021	EPI_ISL_1575077	3/30/2021	Alaska State Virology Laboratory	Alaska State Virology Laboratory	Stephanie DeRonde, Elva House, Lisa Smith, Ph.D., Jack Chen, Ph.D.
hCoV-19/USA/AK-PHL7767/2021	EPI_ISL_1575081	3/26/2021	Alaska State Virology Laboratory	Alaska State Virology Laboratory	Stephanie DeRonde, Elva House, Lisa Smith, Ph.D., Jack Chen, Ph.D.
hCoV-19/USA/AK-PHL7769/2021	EPI_ISL_1575083	3/25/2021	Alaska State Virology Laboratory	Alaska State Virology Laboratory	Stephanie DeRonde, Elva House, Lisa Smith, Ph.D., Jack Chen, Ph.D.
hCoV-19/USA/AK-PHL7774/2021	EPI_ISL_1575086	3/26/2021	Alaska State Virology Laboratory	Alaska State Virology Laboratory	Stephanie DeRonde, Elva House, Lisa Smith, Ph.D., Jack Chen, Ph.D.
hCoV-19/USA/AK-PHL7783/2021	EPI_ISL_1575091	3/25/2021	Alaska State Virology Laboratory	Alaska State Virology Laboratory	Stephanie DeRonde, Elva House, Lisa Smith, Ph.D., Jack Chen, Ph.D.
hCoV-19/USA/AK-PHL7784/2021	EPI_ISL_1575092	3/26/2021	Alaska State Virology Laboratory	Alaska State Virology Laboratory	Stephanie DeRonde, Elva House, Lisa Smith, Ph.D., Jack Chen, Ph.D.
hCoV-19/USA/AK-PHL7786/2021	EPI_ISL_1575094	3/25/2021	Alaska State Virology Laboratory	Alaska State Virology Laboratory	Stephanie DeRonde, Elva House, Lisa Smith, Ph.D., Jack Chen, Ph.D.
hCoV-19/USA/AK-PHL7790/2021	EPI_ISL_1575097	3/30/2021	Alaska State Virology Laboratory	Alaska State Virology Laboratory	Stephanie DeRonde, Elva House, Lisa Smith, Ph.D., Jack Chen, Ph.D.
hCoV-19/USA/AK-PHL7794/2021	EPI_ISL_1575099	3/29/2021	Alaska State Virology Laboratory	Alaska State Virology Laboratory	Stephanie DeRonde, Elva House, Lisa Smith, Ph.D., Jack Chen, Ph.D.
hCoV-19/USA/AK-PHL7795/2021	EPI_ISL_1575100	3/29/2021	Alaska State Virology Laboratory	Alaska State Virology Laboratory	Stephanie DeRonde, Elva House, Lisa Smith, Ph.D., Jack Chen, Ph.D.
hCoV-19/USA/AK-PHL7796/2021	EPI_ISL_1575101	3/29/2021	Alaska State Virology Laboratory	Alaska State Virology Laboratory	Stephanie DeRonde, Elva House, Lisa Smith, Ph.D., Jack Chen, Ph.D.
hCoV-19/USA/AK-PHL7797/2021	EPI_ISL_1575102	3/29/2021	Alaska State Virology Laboratory	Alaska State Virology Laboratory	Stephanie DeRonde, Elva House, Lisa Smith, Ph.D., Jack Chen, Ph.D.
hCoV-19/USA/AK-PHL7798/2021	EPI_ISL_1575103	3/29/2021	Alaska State Virology Laboratory	Alaska State Virology Laboratory	Stephanie DeRonde, Elva House, Lisa Smith, Ph.D., Jack Chen, Ph.D.
hCoV-19/USA/AK-PHL7803/2021	EPI_ISL_1575104	3/31/2021	Alaska State Virology Laboratory	Alaska State Virology Laboratory	Stephanie DeRonde, Elva House, Lisa Smith, Ph.D., Jack Chen, Ph.D.
hCoV-19/USA/AK-CDC-STM-000004274/2021	EPI_ISL_966629	1/22/2021	Helix/Illumina	Respiratory Viruses Branch, Division of Viral Diseases, Centers for Disease Control and Prevention	Peter W. Cook,Dakota Howard,Dhwani Batra,Ben L. Rambo-Martin,Eileen de Feo,Jan Antico,Christine Tran,Matthew Tolentino,Shannon Wickline,Kim Gietzen,Brad Sickler,Jingtao Liu,Eric Allen,Phil Febbo,Summer Galloway,Nicole L. Washington,Simon White,Geraint Levan,Kelly Schiabor Barrett,Elizabeth Cirulli,Alexandre Bolze,Ary Ascencio,Charlotte Rivera-Garcia,Ryan Cho,Jason Nguyen,Sherry Wang,Jimmy Ramirez,Tyler Cassens,Efren Sandoval,Magnus Isaksson,William Lee,David Becker,Marc Laurent,James Lu,Clinton R. Paden,Suxiang Tong,Duncan MacCannell,
hCoV-19/USA/AK-CDC-STM-000005026/2021	EPI_ISL_967154	1/22/2021	Helix/Illumina	Respiratory Viruses Branch, Division of Viral Diseases, Centers for Disease Control and Prevention	Peter W. Cook,Dakota Howard,Dhwani Batra,Ben L. Rambo-Martin,Eileen de Feo,Jan Antico,Christine Tran,Matthew Tolentino,Shannon Wickline,Kim Gietzen,Brad Sickler,Jingtao Liu,Eric Allen,Phil Febbo,Summer Galloway,Nicole L. Washington,Simon White,Geraint Levan,Kelly Schiabor Barrett,Elizabeth Cirulli,Alexandre Bolze,Ary Ascencio,Charlotte Rivera-Garcia,Ryan Cho,Jason Nguyen,Sherry Wang,Jimmy Ramirez,Tyler Cassens,Efren Sandoval,Magnus Isaksson,William Lee,David Becker,Marc Laurent,James Lu,Clinton R. Paden,Suxiang Tong,Duncan MacCannell,
hCoV-19/USA/AK-CDC-STM-000003657/2021	EPI_ISL_967496	1/18/2021	Helix/Illumina	Respiratory Viruses Branch, Division of Viral Diseases, Centers for Disease Control and Prevention	Peter W. Cook,Dakota Howard,Dhwani Batra,Ben L. Rambo-Martin,Eileen de Feo,Jan Antico,Christine Tran,Matthew Tolentino,Shannon Wickline,Kim Gietzen,Brad Sickler,Jingtao Liu,Eric Allen,Phil Febbo,Summer Galloway,Nicole L. Washington,Simon White,Geraint Levan,Kelly Schiabor Barrett,Elizabeth Cirulli,Alexandre Bolze,Ary Ascencio,Charlotte Rivera-Garcia,Ryan Cho,Jason Nguyen,Sherry Wang,Jimmy Ramirez,Tyler Cassens,Efren Sandoval,Magnus Isaksson,William Lee,David Becker,Marc Laurent,James Lu,Clinton R. Paden,Suxiang Tong,Duncan MacCannell,
hCoV-19/USA/AK-CDC-2-3920633/2020	EPI_ISL_1225874	12/22/2020	AK State Public Health Lab, State Health Department	Genomics and Discovery, Respiratory Viruses Branch, Division of Viral Diseases, Centers for Disease Control and Prevention	Krista Queen, Yan Li, Ying Tao, Jing Zhang, Anna Uehara, Anna Montmayeur, Clinton R. Paden, Kristen Knipe, Matthew Schmerer, Shoshona Le, Katie Dillon, Peter W. Cook, Rachel Marine, Mili Sheth, Jasmine Padilla, Sarah Nobles, Mark Burroughs, Lori Rowe, Haibin Wang, Ben L. Rambo-Martin, Kristine Lacek, Sam Shepard, Dhwani Batra, Suxiang Tong, Justin Lee

hCoV-19/USA/AK-CDC-2-3920718/2020	EPI_ISL_1225875	12/24/2020	AK State Public Health Lab, State Health Department	Genomics and Discovery, Respiratory Viruses Branch, Division of Viral Diseases, Centers for Disease Control and Prevention	Krista Queen, Yan Li, Ying Tao, Jing Zhang, Anna Uehara, Anna Montmayeur, Clinton R. Paden, Kristen Knipe, Matthew Schmerer, Shoshona Le, Katie Dillon, Peter W. Cook, Rachel Marine, Mili Sheth, Jasmine Padilla, Sarah Nobles, Mark Burroughs, Lori Rowe, Haibin Wang, Ben L. Rambo-Martin, Kristine Lacek, Sam Shepard, Dhwani Batra, Suxiang Tong, Justin Lee
hCoV-19/USA/AK-CDC-2-3920646/2020	EPI_ISL_1225876	12/1/2020	AK State Public Health Lab, State Health Department	Genomics and Discovery, Respiratory Viruses Branch, Division of Viral Diseases, Centers for Disease Control and Prevention	Krista Queen, Yan Li, Ying Tao, Jing Zhang, Anna Uehara, Anna Montmayeur, Clinton R. Paden, Kristen Knipe, Matthew Schmerer, Shoshona Le, Katie Dillon, Peter W. Cook, Rachel Marine, Mili Sheth, Jasmine Padilla, Sarah Nobles, Mark Burroughs, Lori Rowe, Haibin Wang, Ben L. Rambo-Martin, Kristine Lacek, Sam Shepard, Dhwani Batra, Suxiang Tong, Justin Lee
hCoV-19/USA/AK-CDC-2-3920727/2020	EPI_ISL_1225877	12/22/2020	AK State Public Health Lab, State Health Department	Genomics and Discovery, Respiratory Viruses Branch, Division of Viral Diseases, Centers for Disease Control and Prevention	Krista Queen, Yan Li, Ying Tao, Jing Zhang, Anna Uehara, Anna Montmayeur, Clinton R. Paden, Kristen Knipe, Matthew Schmerer, Shoshona Le, Katie Dillon, Peter W. Cook, Rachel Marine, Mili Sheth, Jasmine Padilla, Sarah Nobles, Mark Burroughs, Lori Rowe, Haibin Wang, Ben L. Rambo-Martin, Kristine Lacek, Sam Shepard, Dhwani Batra, Suxiang Tong, Justin Lee
hCoV-19/USA/AK-CDC-2-3920625/2020	EPI_ISL_1225878	12/4/2020	AK State Public Health Lab, State Health Department	Genomics and Discovery, Respiratory Viruses Branch, Division of Viral Diseases, Centers for Disease Control and Prevention	Krista Queen, Yan Li, Ying Tao, Jing Zhang, Anna Uehara, Anna Montmayeur, Clinton R. Paden, Kristen Knipe, Matthew Schmerer, Shoshona Le, Katie Dillon, Peter W. Cook, Rachel Marine, Mili Sheth, Jasmine Padilla, Sarah Nobles, Mark Burroughs, Lori Rowe, Haibin Wang, Ben L. Rambo-Martin, Kristine Lacek, Sam Shepard, Dhwani Batra, Suxiang Tong, Justin Lee
hCoV-19/USA/AK-CDC-2-3920631/2020	EPI_ISL_1225879	12/1/2020	AK State Public Health Lab, State Health Department	Genomics and Discovery, Respiratory Viruses Branch, Division of Viral Diseases, Centers for Disease Control and Prevention	Krista Queen, Yan Li, Ying Tao, Jing Zhang, Anna Uehara, Anna Montmayeur, Clinton R. Paden, Kristen Knipe, Matthew Schmerer, Shoshona Le, Katie Dillon, Peter W. Cook, Rachel Marine, Mili Sheth, Jasmine Padilla, Sarah Nobles, Mark Burroughs, Lori Rowe, Haibin Wang, Ben L. Rambo-Martin, Kristine Lacek, Sam Shepard, Dhwani Batra, Suxiang Tong, Justin Lee
hCoV-19/USA/AK-CDC-2-3920720/2020	EPI_ISL_1225880	12/9/2020	AK State Public Health Lab, State Health Department	Genomics and Discovery, Respiratory Viruses Branch, Division of Viral Diseases, Centers for Disease Control and Prevention	Krista Queen, Yan Li, Ying Tao, Jing Zhang, Anna Uehara, Anna Montmayeur, Clinton R. Paden, Kristen Knipe, Matthew Schmerer, Shoshona Le, Katie Dillon, Peter W. Cook, Rachel Marine, Mili Sheth, Jasmine Padilla, Sarah Nobles, Mark Burroughs, Lori Rowe, Haibin Wang, Ben L. Rambo-Martin, Kristine Lacek, Sam Shepard, Dhwani Batra, Suxiang Tong, Justin Lee
hCoV-19/USA/AK-CDC-2-3925289/2021	EPI_ISL_1226177	2/10/2021	AK State Public Health Lab, State Health Department	Genomics and Discovery, Respiratory Viruses Branch, Division of Viral Diseases, Centers for Disease Control and Prevention	Krista Queen, Yan Li, Ying Tao, Jing Zhang, Anna Uehara, Anna Montmayeur, Clinton R. Paden, Kristen Knipe, Matthew Schmerer, Shoshona Le, Katie Dillon, Peter W. Cook, Rachel Marine, Mili Sheth, Jasmine Padilla, Sarah Nobles, Mark Burroughs, Lori Rowe, Haibin Wang, Ben L. Rambo-Martin, Kristine Lacek, Sam Shepard, Dhwani Batra, Suxiang Tong, Justin Lee
hCoV-19/USA/AK-CDC-2-3925161/2021	EPI_ISL_1226178	2/10/2021	AK State Public Health Lab, State Health Department	Genomics and Discovery, Respiratory Viruses Branch, Division of Viral Diseases, Centers for Disease Control and Prevention	Krista Queen, Yan Li, Ying Tao, Jing Zhang, Anna Uehara, Anna Montmayeur, Clinton R. Paden, Kristen Knipe, Matthew Schmerer, Shoshona Le, Katie Dillon, Peter W. Cook, Rachel Marine, Mili Sheth, Jasmine Padilla, Sarah Nobles, Mark Burroughs, Lori Rowe, Haibin Wang, Ben L. Rambo-Martin, Kristine Lacek, Sam Shepard, Dhwani Batra, Suxiang Tong, Justin Lee
hCoV-19/USA/AK-CDC-2-3925195/2021	EPI_ISL_1226179	2/11/2021	AK State Public Health Lab, State Health Department	Genomics and Discovery, Respiratory Viruses Branch, Division of Viral Diseases, Centers for Disease Control and Prevention	Krista Queen, Yan Li, Ying Tao, Jing Zhang, Anna Uehara, Anna Montmayeur, Clinton R. Paden, Kristen Knipe, Matthew Schmerer, Shoshona Le, Katie Dillon, Peter W. Cook, Rachel Marine, Mili Sheth, Jasmine Padilla, Sarah Nobles, Mark Burroughs, Lori Rowe, Haibin Wang, Ben L. Rambo-Martin, Kristine Lacek, Sam Shepard, Dhwani Batra, Suxiang Tong, Justin Lee
hCoV-19/USA/AK-CDC-2-3925261/2021	EPI_ISL_1226180	2/11/2021	AK State Public Health Lab, State Health Department	Genomics and Discovery, Respiratory Viruses Branch, Division of Viral Diseases, Centers for Disease Control and Prevention	Krista Queen, Yan Li, Ying Tao, Jing Zhang, Anna Uehara, Anna Montmayeur, Clinton R. Paden, Kristen Knipe, Matthew Schmerer, Shoshona Le, Katie Dillon, Peter W. Cook, Rachel Marine, Mili Sheth, Jasmine Padilla, Sarah Nobles, Mark Burroughs, Lori Rowe, Haibin Wang, Ben L. Rambo-Martin, Kristine Lacek, Sam Shepard, Dhwani Batra, Suxiang Tong, Justin Lee
hCoV-19/USA/AK-CDC-2-3925173/2021	EPI_ISL_1226181	2/6/2021	AK State Public Health Lab, State Health Department	Genomics and Discovery, Respiratory Viruses Branch, Division of Viral Diseases, Centers for Disease Control and Prevention	Krista Queen, Yan Li, Ying Tao, Jing Zhang, Anna Uehara, Anna Montmayeur, Clinton R. Paden, Kristen Knipe, Matthew Schmerer, Shoshona Le, Katie Dillon, Peter W. Cook, Rachel Marine, Mili Sheth, Jasmine Padilla, Sarah Nobles, Mark Burroughs, Lori Rowe, Haibin Wang, Ben L. Rambo-Martin, Kristine Lacek, Sam Shepard, Dhwani Batra, Suxiang Tong, Justin Lee

hCoV-19/USA/AK-CDC-2-3925186/2021	EPI_ISL_1226182	2/6/2021	AK State Public Health Lab, State Health Department	Genomics and Discovery, Respiratory Viruses Branch, Division of Viral Diseases, Centers for Disease Control and Prevention	Krista Queen, Yan Li, Ying Tao, Jing Zhang, Anna Uehara, Anna Montmayeur, Clinton R. Paden, Kristen Knipe, Matthew Schmerer, Shoshona Le, Katie Dillon, Peter W. Cook, Rachel Marine, Mili Sheth, Jasmine Padilla, Sarah Nobles, Mark Burroughs, Lori Rowe, Haibin Wang, Ben L. Rambo-Martin, Kristine Lacek, Sam Shepard, Dhwani Batra, Suxiang Tong, Justin Lee
hCoV-19/USA/AK-CDC-2-3925270/2021	EPI_ISL_1226183	2/7/2021	AK State Public Health Lab, State Health Department	Genomics and Discovery, Respiratory Viruses Branch, Division of Viral Diseases, Centers for Disease Control and Prevention	Krista Queen, Yan Li, Ying Tao, Jing Zhang, Anna Uehara, Anna Montmayeur, Clinton R. Paden, Kristen Knipe, Matthew Schmerer, Shoshona Le, Katie Dillon, Peter W. Cook, Rachel Marine, Mili Sheth, Jasmine Padilla, Sarah Nobles, Mark Burroughs, Lori Rowe, Haibin Wang, Ben L. Rambo-Martin, Kristine Lacek, Sam Shepard, Dhwani Batra, Suxiang Tong, Justin Lee
hCoV-19/USA/AK-CDC-2-3925283/2021	EPI_ISL_1226184	2/7/2021	AK State Public Health Lab, State Health Department	Genomics and Discovery, Respiratory Viruses Branch, Division of Viral Diseases, Centers for Disease Control and Prevention	Krista Queen, Yan Li, Ying Tao, Jing Zhang, Anna Uehara, Anna Montmayeur, Clinton R. Paden, Kristen Knipe, Matthew Schmerer, Shoshona Le, Katie Dillon, Peter W. Cook, Rachel Marine, Mili Sheth, Jasmine Padilla, Sarah Nobles, Mark Burroughs, Lori Rowe, Haibin Wang, Ben L. Rambo-Martin, Kristine Lacek, Sam Shepard, Dhwani Batra, Suxiang Tong, Justin Lee
hCoV-19/USA/AK-CDC-2-3925167/2021	EPI_ISL_1226185	2/7/2021	AK State Public Health Lab, State Health Department	Genomics and Discovery, Respiratory Viruses Branch, Division of Viral Diseases, Centers for Disease Control and Prevention	Krista Queen, Yan Li, Ying Tao, Jing Zhang, Anna Uehara, Anna Montmayeur, Clinton R. Paden, Kristen Knipe, Matthew Schmerer, Shoshona Le, Katie Dillon, Peter W. Cook, Rachel Marine, Mili Sheth, Jasmine Padilla, Sarah Nobles, Mark Burroughs, Lori Rowe, Haibin Wang, Ben L. Rambo-Martin, Kristine Lacek, Sam Shepard, Dhwani Batra, Suxiang Tong, Justin Lee
hCoV-19/USA/AK-CDC-2-3925192/2021	EPI_ISL_1226186	2/8/2021	AK State Public Health Lab, State Health Department	Genomics and Discovery, Respiratory Viruses Branch, Division of Viral Diseases, Centers for Disease Control and Prevention	Krista Queen, Yan Li, Ying Tao, Jing Zhang, Anna Uehara, Anna Montmayeur, Clinton R. Paden, Kristen Knipe, Matthew Schmerer, Shoshona Le, Katie Dillon, Peter W. Cook, Rachel Marine, Mili Sheth, Jasmine Padilla, Sarah Nobles, Mark Burroughs, Lori Rowe, Haibin Wang, Ben L. Rambo-Martin, Kristine Lacek, Sam Shepard, Dhwani Batra, Suxiang Tong, Justin Lee
hCoV-19/USA/AK-CDC-2-3925278/2021	EPI_ISL_1226187	2/8/2021	AK State Public Health Lab, State Health Department	Genomics and Discovery, Respiratory Viruses Branch, Division of Viral Diseases, Centers for Disease Control and Prevention	Krista Queen, Yan Li, Ying Tao, Jing Zhang, Anna Uehara, Anna Montmayeur, Clinton R. Paden, Kristen Knipe, Matthew Schmerer, Shoshona Le, Katie Dillon, Peter W. Cook, Rachel Marine, Mili Sheth, Jasmine Padilla, Sarah Nobles, Mark Burroughs, Lori Rowe, Haibin Wang, Ben L. Rambo-Martin, Kristine Lacek, Sam Shepard, Dhwani Batra, Suxiang Tong, Justin Lee
hCoV-19/USA/AK-CDC-2-3925172/2021	EPI_ISL_1226188	2/7/2021	AK State Public Health Lab, State Health Department	Genomics and Discovery, Respiratory Viruses Branch, Division of Viral Diseases, Centers for Disease Control and Prevention	Krista Queen, Yan Li, Ying Tao, Jing Zhang, Anna Uehara, Anna Montmayeur, Clinton R. Paden, Kristen Knipe, Matthew Schmerer, Shoshona Le, Katie Dillon, Peter W. Cook, Rachel Marine, Mili Sheth, Jasmine Padilla, Sarah Nobles, Mark Burroughs, Lori Rowe, Haibin Wang, Ben L. Rambo-Martin, Kristine Lacek, Sam Shepard, Dhwani Batra, Suxiang Tong, Justin Lee
hCoV-19/USA/AK-CDC-2-3925187/2021	EPI_ISL_1226189	2/7/2021	AK State Public Health Lab, State Health Department	Genomics and Discovery, Respiratory Viruses Branch, Division of Viral Diseases, Centers for Disease Control and Prevention	Krista Queen, Yan Li, Ying Tao, Jing Zhang, Anna Uehara, Anna Montmayeur, Clinton R. Paden, Kristen Knipe, Matthew Schmerer, Shoshona Le, Katie Dillon, Peter W. Cook, Rachel Marine, Mili Sheth, Jasmine Padilla, Sarah Nobles, Mark Burroughs, Lori Rowe, Haibin Wang, Ben L. Rambo-Martin, Kristine Lacek, Sam Shepard, Dhwani Batra, Suxiang Tong, Justin Lee
hCoV-19/USA/AK-CDC-2-3925269/2021	EPI_ISL_1226190	2/7/2021	AK State Public Health Lab, State Health Department	Genomics and Discovery, Respiratory Viruses Branch, Division of Viral Diseases, Centers for Disease Control and Prevention	Krista Queen, Yan Li, Ying Tao, Jing Zhang, Anna Uehara, Anna Montmayeur, Clinton R. Paden, Kristen Knipe, Matthew Schmerer, Shoshona Le, Katie Dillon, Peter W. Cook, Rachel Marine, Mili Sheth, Jasmine Padilla, Sarah Nobles, Mark Burroughs, Lori Rowe, Haibin Wang, Ben L. Rambo-Martin, Kristine Lacek, Sam Shepard, Dhwani Batra, Suxiang Tong, Justin Lee
hCoV-19/USA/AK-CDC-2-3925284/2021	EPI_ISL_1226191	2/7/2021	AK State Public Health Lab, State Health Department	Genomics and Discovery, Respiratory Viruses Branch, Division of Viral Diseases, Centers for Disease Control and Prevention	Krista Queen, Yan Li, Ying Tao, Jing Zhang, Anna Uehara, Anna Montmayeur, Clinton R. Paden, Kristen Knipe, Matthew Schmerer, Shoshona Le, Katie Dillon, Peter W. Cook, Rachel Marine, Mili Sheth, Jasmine Padilla, Sarah Nobles, Mark Burroughs, Lori Rowe, Haibin Wang, Ben L. Rambo-Martin, Kristine Lacek, Sam Shepard, Dhwani Batra, Suxiang Tong, Justin Lee

hCoV-19/USA/AK-CDC-STM-000013926/2021	EPI_ISL_1267794	2/12/2021	Helix/Illumina	Centers for Disease Control and Prevention Division of Viral Diseases, Pathogen Discovery	Peter W. Cook, Dakota Howard, Dhwani Batra, Ben L. Rambo-Martin, Eileen de Feo, Jan Antico, Christine Tran, Matthew Tolentino, Shannon Wickline, Kim Gietzen, Brad Sickler, Jingtao Liu, Eric Allen, Phil Febbo, Summer Galloway, Nicole L. Washington, Simon White, Geraint Levan, Kelly Schiabor Barrett, Elizabeth Cirulli, Alexandre Bolze, Ary Ascencio, Charlotte Rivera-Garcia, Ryan Cho, Jason Nguyen, Sherry Wang, Jimmy Ramirez, Tyler Cassens, Efren Sandoval, Magnus Isaksson, William Lee, David Becker, Marc Laurent, James Lu, Clinton R. Paden, Suxiang Tong, Duncan MacCannell
hCoV-19/USA/AK-CDC-2-3980566/2021	EPI_ISL_1272681	2/25/2021	AK State Public Health Lab, State Health Department	Centers for Disease Control and Prevention Division of Viral Diseases, Pathogen Discovery	Krista Queen, Yan Li, Ying Tao, Jing Zhang, Anna Uehara, Anna Montmayeur, Clinton R. Paden, Kristen Knipe, Matthew Schmerer, Shoshona Le, Katie Dillon, Peter W. Cook, Rachel Marine, Mili Sheth, Jasmine Padilla, Sarah Nobles, Mark Burroughs, Lori Rowe, Haibin Wang, Ben L. Rambo-Martin, Kristine Lacek, Sam Shepard, Dhwani Batra, Suxiang Tong, Justin Lee
hCoV-19/USA/AK-CDC-2-3980468/2021	EPI_ISL_1272682	2/22/2021	AK State Public Health Lab, State Health Department	Centers for Disease Control and Prevention Division of Viral Diseases, Pathogen Discovery	Krista Queen, Yan Li, Ying Tao, Jing Zhang, Anna Uehara, Anna Montmayeur, Clinton R. Paden, Kristen Knipe, Matthew Schmerer, Shoshona Le, Katie Dillon, Peter W. Cook, Rachel Marine, Mili Sheth, Jasmine Padilla, Sarah Nobles, Mark Burroughs, Lori Rowe, Haibin Wang, Ben L. Rambo-Martin, Kristine Lacek, Sam Shepard, Dhwani Batra, Suxiang Tong, Justin Lee
hCoV-19/USA/AK-CDC-2-3980564/2021	EPI_ISL_1272683	2/25/2021	AK State Public Health Lab, State Health Department	Centers for Disease Control and Prevention Division of Viral Diseases, Pathogen Discovery	Krista Queen, Yan Li, Ying Tao, Jing Zhang, Anna Uehara, Anna Montmayeur, Clinton R. Paden, Kristen Knipe, Matthew Schmerer, Shoshona Le, Katie Dillon, Peter W. Cook, Rachel Marine, Mili Sheth, Jasmine Padilla, Sarah Nobles, Mark Burroughs, Lori Rowe, Haibin Wang, Ben L. Rambo-Martin, Kristine Lacek, Sam Shepard, Dhwani Batra, Suxiang Tong, Justin Lee
hCoV-19/USA/AK-CDC-2-3980558/2021	EPI_ISL_1272684	2/22/2021	AK State Public Health Lab, State Health Department	Centers for Disease Control and Prevention Division of Viral Diseases, Pathogen Discovery	Krista Queen, Yan Li, Ying Tao, Jing Zhang, Anna Uehara, Anna Montmayeur, Clinton R. Paden, Kristen Knipe, Matthew Schmerer, Shoshona Le, Katie Dillon, Peter W. Cook, Rachel Marine, Mili Sheth, Jasmine Padilla, Sarah Nobles, Mark Burroughs, Lori Rowe, Haibin Wang, Ben L. Rambo-Martin, Kristine Lacek, Sam Shepard, Dhwani Batra, Suxiang Tong, Justin Lee
hCoV-19/USA/AK-CDC-2-3980659/2021	EPI_ISL_1272685	2/22/2021	AK State Public Health Lab, State Health Department	Centers for Disease Control and Prevention Division of Viral Diseases, Pathogen Discovery	Krista Queen, Yan Li, Ying Tao, Jing Zhang, Anna Uehara, Anna Montmayeur, Clinton R. Paden, Kristen Knipe, Matthew Schmerer, Shoshona Le, Katie Dillon, Peter W. Cook, Rachel Marine, Mili Sheth, Jasmine Padilla, Sarah Nobles, Mark Burroughs, Lori Rowe, Haibin Wang, Ben L. Rambo-Martin, Kristine Lacek, Sam Shepard, Dhwani Batra, Suxiang Tong, Justin Lee
hCoV-19/USA/AK-CDC-2-3980479/2021	EPI_ISL_1272686	2/23/2021	AK State Public Health Lab, State Health Department	Centers for Disease Control and Prevention Division of Viral Diseases, Pathogen Discovery	Krista Queen, Yan Li, Ying Tao, Jing Zhang, Anna Uehara, Anna Montmayeur, Clinton R. Paden, Kristen Knipe, Matthew Schmerer, Shoshona Le, Katie Dillon, Peter W. Cook, Rachel Marine, Mili Sheth, Jasmine Padilla, Sarah Nobles, Mark Burroughs, Lori Rowe, Haibin Wang, Ben L. Rambo-Martin, Kristine Lacek, Sam Shepard, Dhwani Batra, Suxiang Tong, Justin Lee
hCoV-19/USA/AK-CDC-2-3980561/2021	EPI_ISL_1272687	2/22/2021	AK State Public Health Lab, State Health Department	Centers for Disease Control and Prevention Division of Viral Diseases, Pathogen Discovery	Krista Queen, Yan Li, Ying Tao, Jing Zhang, Anna Uehara, Anna Montmayeur, Clinton R. Paden, Kristen Knipe, Matthew Schmerer, Shoshona Le, Katie Dillon, Peter W. Cook, Rachel Marine, Mili Sheth, Jasmine Padilla, Sarah Nobles, Mark Burroughs, Lori Rowe, Haibin Wang, Ben L. Rambo-Martin, Kristine Lacek, Sam Shepard, Dhwani Batra, Suxiang Tong, Justin Lee
hCoV-19/USA/AK-CDC-2-3980499/2021	EPI_ISL_1272688	2/22/2021	AK State Public Health Lab, State Health Department	Centers for Disease Control and Prevention Division of Viral Diseases, Pathogen Discovery	Krista Queen, Yan Li, Ying Tao, Jing Zhang, Anna Uehara, Anna Montmayeur, Clinton R. Paden, Kristen Knipe, Matthew Schmerer, Shoshona Le, Katie Dillon, Peter W. Cook, Rachel Marine, Mili Sheth, Jasmine Padilla, Sarah Nobles, Mark Burroughs, Lori Rowe, Haibin Wang, Ben L. Rambo-Martin, Kristine Lacek, Sam Shepard, Dhwani Batra, Suxiang Tong, Justin Lee
hCoV-19/USA/AK-CDC-LC0000711/2020	EPI_ISL_1028447	12/21/2020	Laboratory Corporation of America	Respiratory Viruses Branch, Division of Viral Diseases, Centers for Disease Control and Prevention	Peter W. Cook, Dakota Howard, Dhwani Batra, Ben L. Rambo-Martin, Clinton R. Paden, Suxiang Tong, Duncan MacCannell
hCoV-19/USA/AK-CDC-LC0009308/2021	EPI_ISL_1031423	1/23/2021	Laboratory Corporation of America	Respiratory Viruses Branch, Division of Viral Diseases, Centers for Disease Control and Prevention	Peter W. Cook, Dakota Howard, Dhwani Batra, Ben L. Rambo-Martin, Clinton R. Paden, Suxiang Tong, Duncan MacCannell
hCoV-19/USA/AK-CDC-LC0012094/2021	EPI_ISL_1037676	1/29/2021	Laboratory Corporation of America	Respiratory Viruses Branch, Division of Viral Diseases, Centers for Disease Control and Prevention	Peter W. Cook, Dakota Howard, Dhwani Batra, Ben L. Rambo-Martin, Clinton R. Paden, Suxiang Tong, Duncan MacCannell
hCoV-19/USA/AK-CDC-LC0011667/2021	EPI_ISL_1038553	1/29/2021	Laboratory Corporation of America	Respiratory Viruses Branch, Division of Viral Diseases, Centers for Disease Control and Prevention	Peter W. Cook, Dakota Howard, Dhwani Batra, Ben L. Rambo-Martin, Clinton R. Paden, Suxiang Tong, Duncan MacCannell

hCoV-19/USA/AK-CDC-QDX21803543/2021	EPI_ISL_1091028	2/5/2021	Quest Diagnostics Incorporated	Respiratory Viruses Branch, Division of Viral Diseases, Centers for Disease Control and Prevention	Peter W. Cook, Dakota Howard, Dhwani Batra, Ben L. Rambo-Martin, S. H. Rosenthal, A. Gerasimova, R. M. Kagan, B. Anderson, M. Hua, Y. Liu, L.E. Bernstein, K.E. Livingston, A. Perez, I. A. Shlyakhter, R. V. Rolando, R. Owen, P. Tanpaiboon, F. Lacbawan, Clinton R. Paden, Suxiang Tong, Duncan MacCannell
hCoV-19/USA/AK-CDC-QDX21804109/2021	EPI_ISL_1091062	2/4/2021	Quest Diagnostics Incorporated	Respiratory Viruses Branch, Division of Viral Diseases, Centers for Disease Control and Prevention	Peter W. Cook, Dakota Howard, Dhwani Batra, Ben L. Rambo-Martin, S. H. Rosenthal, A. Gerasimova, R. M. Kagan, B. Anderson, M. Hua, Y. Liu, L.E. Bernstein, K.E. Livingston, A. Perez, I. A. Shlyakhter, R. V. Rolando, R. Owen, P. Tanpaiboon, F. Lacbawan, Clinton R. Paden, Suxiang Tong, Duncan MacCannell
hCoV-19/USA/AK-CDC-QDX21803962/2021	EPI_ISL_1091064	2/4/2021	Quest Diagnostics Incorporated	Respiratory Viruses Branch, Division of Viral Diseases, Centers for Disease Control and Prevention	Peter W. Cook, Dakota Howard, Dhwani Batra, Ben L. Rambo-Martin, S. H. Rosenthal, A. Gerasimova, R. M. Kagan, B. Anderson, M. Hua, Y. Liu, L.E. Bernstein, K.E. Livingston, A. Perez, I. A. Shlyakhter, R. V. Rolando, R. Owen, P. Tanpaiboon, F. Lacbawan, Clinton R. Paden, Suxiang Tong, Duncan MacCannell
hCoV-19/USA/AK-CDC-QDX21803856/2021	EPI_ISL_1091077	2/4/2021	Quest Diagnostics Incorporated	Respiratory Viruses Branch, Division of Viral Diseases, Centers for Disease Control and Prevention	Peter W. Cook, Dakota Howard, Dhwani Batra, Ben L. Rambo-Martin, S. H. Rosenthal, A. Gerasimova, R. M. Kagan, B. Anderson, M. Hua, Y. Liu, L.E. Bernstein, K.E. Livingston, A. Perez, I. A. Shlyakhter, R. V. Rolando, R. Owen, P. Tanpaiboon, F. Lacbawan, Clinton R. Paden, Suxiang Tong, Duncan MacCannell
hCoV-19/USA/AK-CDC-QDX21803437/2021	EPI_ISL_1091080	2/4/2021	Quest Diagnostics Incorporated	Respiratory Viruses Branch, Division of Viral Diseases, Centers for Disease Control and Prevention	Peter W. Cook, Dakota Howard, Dhwani Batra, Ben L. Rambo-Martin, S. H. Rosenthal, A. Gerasimova, R. M. Kagan, B. Anderson, M. Hua, Y. Liu, L.E. Bernstein, K.E. Livingston, A. Perez, I. A. Shlyakhter, R. V. Rolando, R. Owen, P. Tanpaiboon, F. Lacbawan, Clinton R. Paden, Suxiang Tong, Duncan MacCannell
hCoV-19/USA/AK-CDC-2-3845938/2021	EPI_ISL_1094238	1/11/2021	AK State Public Health Lab, State Health Department	Respiratory Viruses Branch, Division of Viral Diseases, Centers for Disease Control and Prevention	Krista Queen, Yan Li, Ying Tao, Jing Zhang, Anna Uehara, Anna Montmayeur, Clinton R. Paden, Peter W. Cook, Rachel Marine, Mili Sheth, Jasmine Padilla, Sarah Nobles, Mark Burroughs, Lori Rowe, Haibin Wang, Ben L. Rambo-Martin, Dhwani Batra, Justin Lee, Suxiang Tong
hCoV-19/USA/AK-CDC-2-3829823/2021	EPI_ISL_1094272	1/4/2021	AK State Public Health Lab, State Health Department	Respiratory Viruses Branch, Division of Viral Diseases, Centers for Disease Control and Prevention	Krista Queen, Yan Li, Ying Tao, Jing Zhang, Anna Uehara, Anna Montmayeur, Clinton R. Paden, Peter W. Cook, Rachel Marine, Mili Sheth, Jasmine Padilla, Sarah Nobles, Mark Burroughs, Lori Rowe, Haibin Wang, Ben L. Rambo-Martin, Dhwani Batra, Justin Lee, Suxiang Tong
hCoV-19/USA/AK-CDC-2-3845829/2021	EPI_ISL_1094319	1/20/2021	AK State Public Health Lab, State Health Department	Respiratory Viruses Branch, Division of Viral Diseases, Centers for Disease Control and Prevention	Krista Queen, Yan Li, Ying Tao, Jing Zhang, Anna Uehara, Anna Montmayeur, Clinton R. Paden, Peter W. Cook, Rachel Marine, Mili Sheth, Jasmine Padilla, Sarah Nobles, Mark Burroughs, Lori Rowe, Haibin Wang, Ben L. Rambo-Martin, Dhwani Batra, Justin Lee, Suxiang Tong
hCoV-19/USA/AK-CDC-2-3829838/2021	EPI_ISL_1094353	1/6/2021	AK State Public Health Lab, State Health Department	Respiratory Viruses Branch, Division of Viral Diseases, Centers for Disease Control and Prevention	Krista Queen, Yan Li, Ying Tao, Jing Zhang, Anna Uehara, Anna Montmayeur, Clinton R. Paden, Peter W. Cook, Rachel Marine, Mili Sheth, Jasmine Padilla, Sarah Nobles, Mark Burroughs, Lori Rowe, Haibin Wang, Ben L. Rambo-Martin, Dhwani Batra, Justin Lee, Suxiang Tong
hCoV-19/USA/AK-CDC-2-3829919/2021	EPI_ISL_1094366	1/4/2021	AK State Public Health Lab, State Health Department	Respiratory Viruses Branch, Division of Viral Diseases, Centers for Disease Control and Prevention	Krista Queen, Yan Li, Ying Tao, Jing Zhang, Anna Uehara, Anna Montmayeur, Clinton R. Paden, Peter W. Cook, Rachel Marine, Mili Sheth, Jasmine Padilla, Sarah Nobles, Mark Burroughs, Lori Rowe, Haibin Wang, Ben L. Rambo-Martin, Dhwani Batra, Justin Lee, Suxiang Tong
hCoV-19/USA/AK-CDC-2-3845936/2021	EPI_ISL_1094380	1/11/2021	AK State Public Health Lab, State Health Department	Respiratory Viruses Branch, Division of Viral Diseases, Centers for Disease Control and Prevention	Krista Queen, Yan Li, Ying Tao, Jing Zhang, Anna Uehara, Anna Montmayeur, Clinton R. Paden, Peter W. Cook, Rachel Marine, Mili Sheth, Jasmine Padilla, Sarah Nobles, Mark Burroughs, Lori Rowe, Haibin Wang, Ben L. Rambo-Martin, Dhwani Batra, Justin Lee, Suxiang Tong
hCoV-19/USA/AK-CDC-2-3829810/2021	EPI_ISL_1094707	1/8/2021	AK State Public Health Lab, State Health Department	Respiratory Viruses Branch, Division of Viral Diseases, Centers for Disease Control and Prevention	Krista Queen, Yan Li, Ying Tao, Jing Zhang, Anna Uehara, Anna Montmayeur, Clinton R. Paden, Peter W. Cook, Rachel Marine, Mili Sheth, Jasmine Padilla, Sarah Nobles, Mark Burroughs, Lori Rowe, Haibin Wang, Ben L. Rambo-Martin, Dhwani Batra, Justin Lee, Suxiang Tong
hCoV-19/USA/AK-CDC-2-3845943/2021	EPI_ISL_1094708	1/12/2021	AK State Public Health Lab, State Health Department	Respiratory Viruses Branch, Division of Viral Diseases, Centers for Disease Control and Prevention	Krista Queen, Yan Li, Ying Tao, Jing Zhang, Anna Uehara, Anna Montmayeur, Clinton R. Paden, Peter W. Cook, Rachel Marine, Mili Sheth, Jasmine Padilla, Sarah Nobles, Mark Burroughs, Lori Rowe, Haibin Wang, Ben L. Rambo-Martin, Dhwani Batra, Justin Lee, Suxiang Tong
hCoV-19/USA/AK-CDC-2-3845948/2021	EPI_ISL_1094709	1/11/2021	AK State Public Health Lab, State Health Department	Respiratory Viruses Branch, Division of Viral Diseases, Centers for Disease Control and Prevention	Krista Queen, Yan Li, Ying Tao, Jing Zhang, Anna Uehara, Anna Montmayeur, Clinton R. Paden, Peter W. Cook, Rachel Marine, Mili Sheth, Jasmine Padilla, Sarah Nobles, Mark Burroughs, Lori Rowe, Haibin Wang, Ben L. Rambo-Martin, Dhwani Batra, Justin Lee, Suxiang Tong

hCoV-19/USA/AK-CDC-2-3829835/2021	EPI_ISL_1094710	1/3/2021	AK State Public Health Lab, State Health Department	Respiratory Viruses Branch, Division of Viral Diseases, Centers for Disease Control and Prevention	Krista Queen, Yan Li, Ying Tao, Jing Zhang, Anna Uehara, Anna Montmayeur, Clinton R. Paden, Peter W. Cook, Rachel Marine, Mili Sheth, Jasmine Padilla, Sarah Nobles, Mark Burroughs, Lori Rowe, Haibin Wang, Ben L. Rambo-Martin, Dhwani Batra, Justin Lee, Suxiang Tong
hCoV-19/USA/AK-CDC-2-3829930/2021	EPI_ISL_1094711	1/8/2021	AK State Public Health Lab, State Health Department	Respiratory Viruses Branch, Division of Viral Diseases, Centers for Disease Control and Prevention	Krista Queen, Yan Li, Ying Tao, Jing Zhang, Anna Uehara, Anna Montmayeur, Clinton R. Paden, Peter W. Cook, Rachel Marine, Mili Sheth, Jasmine Padilla, Sarah Nobles, Mark Burroughs, Lori Rowe, Haibin Wang, Ben L. Rambo-Martin, Dhwani Batra, Justin Lee, Suxiang Tong
hCoV-19/USA/AK-CDC-2-3829906/2021	EPI_ISL_1094712	1/5/2021	AK State Public Health Lab, State Health Department	Respiratory Viruses Branch, Division of Viral Diseases, Centers for Disease Control and Prevention	Krista Queen, Yan Li, Ying Tao, Jing Zhang, Anna Uehara, Anna Montmayeur, Clinton R. Paden, Peter W. Cook, Rachel Marine, Mili Sheth, Jasmine Padilla, Sarah Nobles, Mark Burroughs, Lori Rowe, Haibin Wang, Ben L. Rambo-Martin, Dhwani Batra, Justin Lee, Suxiang Tong
hCoV-19/USA/AK-CDC-2-3845850/2021	EPI_ISL_1094713	1/11/2021	AK State Public Health Lab, State Health Department	Respiratory Viruses Branch, Division of Viral Diseases, Centers for Disease Control and Prevention	Krista Queen, Yan Li, Ying Tao, Jing Zhang, Anna Uehara, Anna Montmayeur, Clinton R. Paden, Peter W. Cook, Rachel Marine, Mili Sheth, Jasmine Padilla, Sarah Nobles, Mark Burroughs, Lori Rowe, Haibin Wang, Ben L. Rambo-Martin, Dhwani Batra, Justin Lee, Suxiang Tong
hCoV-19/USA/AK-CDC-2-3829937/2021	EPI_ISL_1094714	1/4/2021	AK State Public Health Lab, State Health Department	Respiratory Viruses Branch, Division of Viral Diseases, Centers for Disease Control and Prevention	Krista Queen, Yan Li, Ying Tao, Jing Zhang, Anna Uehara, Anna Montmayeur, Clinton R. Paden, Peter W. Cook, Rachel Marine, Mili Sheth, Jasmine Padilla, Sarah Nobles, Mark Burroughs, Lori Rowe, Haibin Wang, Ben L. Rambo-Martin, Dhwani Batra, Justin Lee, Suxiang Tong
hCoV-19/USA/AK-CDC-2-3829931/2021	EPI_ISL_1094715	1/6/2021	AK State Public Health Lab, State Health Department	Respiratory Viruses Branch, Division of Viral Diseases, Centers for Disease Control and Prevention	Krista Queen, Yan Li, Ying Tao, Jing Zhang, Anna Uehara, Anna Montmayeur, Clinton R. Paden, Peter W. Cook, Rachel Marine, Mili Sheth, Jasmine Padilla, Sarah Nobles, Mark Burroughs, Lori Rowe, Haibin Wang, Ben L. Rambo-Martin, Dhwani Batra, Justin Lee, Suxiang Tong
hCoV-19/USA/AK-CDC-2-3845949/2021	EPI_ISL_1094716	1/17/2021	AK State Public Health Lab, State Health Department	Respiratory Viruses Branch, Division of Viral Diseases, Centers for Disease Control and Prevention	Krista Queen, Yan Li, Ying Tao, Jing Zhang, Anna Uehara, Anna Montmayeur, Clinton R. Paden, Peter W. Cook, Rachel Marine, Mili Sheth, Jasmine Padilla, Sarah Nobles, Mark Burroughs, Lori Rowe, Haibin Wang, Ben L. Rambo-Martin, Dhwani Batra, Justin Lee, Suxiang Tong
hCoV-19/USA/AK-CDC-2-3845907/2021	EPI_ISL_1094717	1/15/2021	AK State Public Health Lab, State Health Department	Respiratory Viruses Branch, Division of Viral Diseases, Centers for Disease Control and Prevention	Krista Queen, Yan Li, Ying Tao, Jing Zhang, Anna Uehara, Anna Montmayeur, Clinton R. Paden, Peter W. Cook, Rachel Marine, Mili Sheth, Jasmine Padilla, Sarah Nobles, Mark Burroughs, Lori Rowe, Haibin Wang, Ben L. Rambo-Martin, Dhwani Batra, Justin Lee, Suxiang Tong
hCoV-19/USA/AK-CDC-2-3845950/2021	EPI_ISL_1094718	1/12/2021	AK State Public Health Lab, State Health Department	Respiratory Viruses Branch, Division of Viral Diseases, Centers for Disease Control and Prevention	Krista Queen, Yan Li, Ying Tao, Jing Zhang, Anna Uehara, Anna Montmayeur, Clinton R. Paden, Peter W. Cook, Rachel Marine, Mili Sheth, Jasmine Padilla, Sarah Nobles, Mark Burroughs, Lori Rowe, Haibin Wang, Ben L. Rambo-Martin, Dhwani Batra, Justin Lee, Suxiang Tong
hCoV-19/USA/AK-CDC-2-3829905/2021	EPI_ISL_1094719	1/12/2021	AK State Public Health Lab, State Health Department	Respiratory Viruses Branch, Division of Viral Diseases, Centers for Disease Control and Prevention	Krista Queen, Yan Li, Ying Tao, Jing Zhang, Anna Uehara, Anna Montmayeur, Clinton R. Paden, Peter W. Cook, Rachel Marine, Mili Sheth, Jasmine Padilla, Sarah Nobles, Mark Burroughs, Lori Rowe, Haibin Wang, Ben L. Rambo-Martin, Dhwani Batra, Justin Lee, Suxiang Tong
hCoV-19/USA/AK-CDC-2-3845836/2021	EPI_ISL_1095227	1/14/2021	AK State Public Health Lab, State Health Department	Respiratory Viruses Branch, Division of Viral Diseases, Centers for Disease Control and Prevention	Krista Queen, Yan Li, Ying Tao, Jing Zhang, Anna Uehara, Anna Montmayeur, Clinton R. Paden, Peter W. Cook, Rachel Marine, Mili Sheth, Jasmine Padilla, Sarah Nobles, Mark Burroughs, Lori Rowe, Haibin Wang, Ben L. Rambo-Martin, Dhwani Batra, Justin Lee, Suxiang Tong
hCoV-19/USA/AK-CDC-2-3845901/2021	EPI_ISL_1095228	1/22/2021	AK State Public Health Lab, State Health Department	Respiratory Viruses Branch, Division of Viral Diseases, Centers for Disease Control and Prevention	Krista Queen, Yan Li, Ying Tao, Jing Zhang, Anna Uehara, Anna Montmayeur, Clinton R. Paden, Peter W. Cook, Rachel Marine, Mili Sheth, Jasmine Padilla, Sarah Nobles, Mark Burroughs, Lori Rowe, Haibin Wang, Ben L. Rambo-Martin, Dhwani Batra, Justin Lee, Suxiang Tong
hCoV-19/USA/AK-CDC-2-3845927/2021	EPI_ISL_1095229	1/15/2021	AK State Public Health Lab, State Health Department	Respiratory Viruses Branch, Division of Viral Diseases, Centers for Disease Control and Prevention	Krista Queen, Yan Li, Ying Tao, Jing Zhang, Anna Uehara, Anna Montmayeur, Clinton R. Paden, Peter W. Cook, Rachel Marine, Mili Sheth, Jasmine Padilla, Sarah Nobles, Mark Burroughs, Lori Rowe, Haibin Wang, Ben L. Rambo-Martin, Dhwani Batra, Justin Lee, Suxiang Tong
hCoV-19/USA/AK-CDC-2-3845935/2021	EPI_ISL_1095230	1/25/2021	AK State Public Health Lab, State Health Department	Respiratory Viruses Branch, Division of Viral Diseases, Centers for Disease Control and Prevention	Krista Queen, Yan Li, Ying Tao, Jing Zhang, Anna Uehara, Anna Montmayeur, Clinton R. Paden, Peter W. Cook, Rachel Marine, Mili Sheth, Jasmine Padilla, Sarah Nobles, Mark Burroughs, Lori Rowe, Haibin Wang, Ben L. Rambo-Martin, Dhwani Batra, Justin Lee, Suxiang Tong

hCoV-19/USA/AK-CDC-2-3845906/2021	EPI_ISL_1095231	1/12/2021	AK State Public Health Lab, State Health Department	Respiratory Viruses Branch, Division of Viral Diseases, Centers for Disease Control and Prevention	Krista Queen, Yan Li, Ying Tao, Jing Zhang, Anna Uehara, Anna Montmayeur, Clinton R. Paden, Peter W. Cook, Rachel Marine, Mili Sheth, Jasmine Padilla, Sarah Nobles, Mark Burroughs, Lori Rowe, Haibin Wang, Ben L. Rambo-Martin, Dhwani Batra, Justin Lee, Suxiang Tong
hCoV-19/USA/AK-CDC-2-3845830/2021	EPI_ISL_1095232	1/11/2021	AK State Public Health Lab, State Health Department	Respiratory Viruses Branch, Division of Viral Diseases, Centers for Disease Control and Prevention	Krista Queen, Yan Li, Ying Tao, Jing Zhang, Anna Uehara, Anna Montmayeur, Clinton R. Paden, Peter W. Cook, Rachel Marine, Mili Sheth, Jasmine Padilla, Sarah Nobles, Mark Burroughs, Lori Rowe, Haibin Wang, Ben L. Rambo-Martin, Dhwani Batra, Justin Lee, Suxiang Tong
hCoV-19/USA/AK-CDC-2-4043715/2021	EPI_ISL_1446651	3/8/2021	AK State Public Health Lab, State Health Department	Centers for Disease Control and Prevention Division of Viral Diseases, Pathogen Discovery	Mili Sheth, Sarah Nobles, Jasmine Padilla, Mark Burroughs, Shoshona Le, Katie Dillon, Peter Cook, Clinton R. Paden, Dhwani Batra, Krista Queen, Kristen Knipe, Dakota Howard, Yvette Unoarumhi, Darlene Wagner, Matthew Schmerer, Ben L. Rambo-Martin, Kristine Lacek, Sam Shepard, Alison Laufer Halpin, Dave Wentworth, Vivien Dugan, Suxiang Tong, Justin Lee
hCoV-19/USA/AK-CDC-2-4043723/2021	EPI_ISL_1446653	3/7/2021	AK State Public Health Lab, State Health Department	Centers for Disease Control and Prevention Division of Viral Diseases, Pathogen Discovery	Mili Sheth, Sarah Nobles, Jasmine Padilla, Mark Burroughs, Shoshona Le, Katie Dillon, Peter Cook, Clinton R. Paden, Dhwani Batra, Krista Queen, Kristen Knipe, Dakota Howard, Yvette Unoarumhi, Darlene Wagner, Matthew Schmerer, Ben L. Rambo-Martin, Kristine Lacek, Sam Shepard, Alison Laufer Halpin, Dave Wentworth, Vivien Dugan, Suxiang Tong, Justin Lee
hCoV-19/USA/AK-CDC-2-4043812/2021	EPI_ISL_1446654	3/2/2021	AK State Public Health Lab, State Health Department	Centers for Disease Control and Prevention Division of Viral Diseases, Pathogen Discovery	Mili Sheth, Sarah Nobles, Jasmine Padilla, Mark Burroughs, Shoshona Le, Katie Dillon, Peter Cook, Clinton R. Paden, Dhwani Batra, Krista Queen, Kristen Knipe, Dakota Howard, Yvette Unoarumhi, Darlene Wagner, Matthew Schmerer, Ben L. Rambo-Martin, Kristine Lacek, Sam Shepard, Alison Laufer Halpin, Dave Wentworth, Vivien Dugan, Suxiang Tong, Justin Lee
hCoV-19/USA/AK-CDC-2-4043831/2021	EPI_ISL_1446655	3/8/2021	AK State Public Health Lab, State Health Department	Centers for Disease Control and Prevention Division of Viral Diseases, Pathogen Discovery	Mili Sheth, Sarah Nobles, Jasmine Padilla, Mark Burroughs, Shoshona Le, Katie Dillon, Peter Cook, Clinton R. Paden, Dhwani Batra, Krista Queen, Kristen Knipe, Dakota Howard, Yvette Unoarumhi, Darlene Wagner, Matthew Schmerer, Ben L. Rambo-Martin, Kristine Lacek, Sam Shepard, Alison Laufer Halpin, Dave Wentworth, Vivien Dugan, Suxiang Tong, Justin Lee
hCoV-19/USA/AK-CDC-2-4043725/2021	EPI_ISL_1446656	3/7/2021	AK State Public Health Lab, State Health Department	Centers for Disease Control and Prevention Division of Viral Diseases, Pathogen Discovery	Mili Sheth, Sarah Nobles, Jasmine Padilla, Mark Burroughs, Shoshona Le, Katie Dillon, Peter Cook, Clinton R. Paden, Dhwani Batra, Krista Queen, Kristen Knipe, Dakota Howard, Yvette Unoarumhi, Darlene Wagner, Matthew Schmerer, Ben L. Rambo-Martin, Kristine Lacek, Sam Shepard, Alison Laufer Halpin, Dave Wentworth, Vivien Dugan, Suxiang Tong, Justin Lee
hCoV-19/USA/AK-CDC-2-4043830/2021	EPI_ISL_1446657	3/7/2021	AK State Public Health Lab, State Health Department	Centers for Disease Control and Prevention Division of Viral Diseases, Pathogen Discovery	Mili Sheth, Sarah Nobles, Jasmine Padilla, Mark Burroughs, Shoshona Le, Katie Dillon, Peter Cook, Clinton R. Paden, Dhwani Batra, Krista Queen, Kristen Knipe, Dakota Howard, Yvette Unoarumhi, Darlene Wagner, Matthew Schmerer, Ben L. Rambo-Martin, Kristine Lacek, Sam Shepard, Alison Laufer Halpin, Dave Wentworth, Vivien Dugan, Suxiang Tong, Justin Lee
hCoV-19/USA/AK-CDC-2-4043822/2021	EPI_ISL_1446658	3/8/2021	AK State Public Health Lab, State Health Department	Centers for Disease Control and Prevention Division of Viral Diseases, Pathogen Discovery	Mili Sheth, Sarah Nobles, Jasmine Padilla, Mark Burroughs, Shoshona Le, Katie Dillon, Peter Cook, Clinton R. Paden, Dhwani Batra, Krista Queen, Kristen Knipe, Dakota Howard, Yvette Unoarumhi, Darlene Wagner, Matthew Schmerer, Ben L. Rambo-Martin, Kristine Lacek, Sam Shepard, Alison Laufer Halpin, Dave Wentworth, Vivien Dugan, Suxiang Tong, Justin Lee
hCoV-19/USA/AK-CDC-2-4043750/2021	EPI_ISL_1446659	3/1/2021	AK State Public Health Lab, State Health Department	Centers for Disease Control and Prevention Division of Viral Diseases, Pathogen Discovery	Mili Sheth, Sarah Nobles, Jasmine Padilla, Mark Burroughs, Shoshona Le, Katie Dillon, Peter Cook, Clinton R. Paden, Dhwani Batra, Krista Queen, Kristen Knipe, Dakota Howard, Yvette Unoarumhi, Darlene Wagner, Matthew Schmerer, Ben L. Rambo-Martin, Kristine Lacek, Sam Shepard, Alison Laufer Halpin, Dave Wentworth, Vivien Dugan, Suxiang Tong, Justin Lee
hCoV-19/USA/AK-CDC-2-4043726/2021	EPI_ISL_1446660	3/6/2021	AK State Public Health Lab, State Health Department	Centers for Disease Control and Prevention Division of Viral Diseases, Pathogen Discovery	Mili Sheth, Sarah Nobles, Jasmine Padilla, Mark Burroughs, Shoshona Le, Katie Dillon, Peter Cook, Clinton R. Paden, Dhwani Batra, Krista Queen, Kristen Knipe, Dakota Howard, Yvette Unoarumhi, Darlene Wagner, Matthew Schmerer, Ben L. Rambo-Martin, Kristine Lacek, Sam Shepard, Alison Laufer Halpin, Dave Wentworth, Vivien Dugan, Suxiang Tong, Justin Lee

hCoV-19/USA/AK-CDC-2-4043744/2021	EPI_ISL_1446661	3/9/2021	AK State Public Health Lab, State Health Department	Centers for Disease Control and Prevention Division of Viral Diseases, Pathogen Discovery	Mili Sheth, Sarah Nobles, Jasmine Padilla, Mark Burroughs, Shoshona Le, Katie Dillon, Peter Cook, Clinton R. Paden, Dhwani Batra, Krista Queen, Kristen Knipe, Dakota Howard, Yvette Unoarumhi, Darlene Wagner, Matthew Schmerer, Ben L. Rambo-Martin, Kristine Lacek, Sam Shepard, Alison Laufer Halpin, Dave Wentworth, Vivien Dugan, Suxiang Tong, Justin Lee
hCoV-19/USA/AK-CDC-2-4043724/2021	EPI_ISL_1446662	3/7/2021	AK State Public Health Lab, State Health Department	Centers for Disease Control and Prevention Division of Viral Diseases, Pathogen Discovery	Mili Sheth, Sarah Nobles, Jasmine Padilla, Mark Burroughs, Shoshona Le, Katie Dillon, Peter Cook, Clinton R. Paden, Dhwani Batra, Krista Queen, Kristen Knipe, Dakota Howard, Yvette Unoarumhi, Darlene Wagner, Matthew Schmerer, Ben L. Rambo-Martin, Kristine Lacek, Sam Shepard, Alison Laufer Halpin, Dave Wentworth, Vivien Dugan, Suxiang Tong, Justin Lee
hCoV-19/USA/AK-CDC-2-4043844/2021	EPI_ISL_1446664	3/9/2021	AK State Public Health Lab, State Health Department	Centers for Disease Control and Prevention Division of Viral Diseases, Pathogen Discovery	Mili Sheth, Sarah Nobles, Jasmine Padilla, Mark Burroughs, Shoshona Le, Katie Dillon, Peter Cook, Clinton R. Paden, Dhwani Batra, Krista Queen, Kristen Knipe, Dakota Howard, Yvette Unoarumhi, Darlene Wagner, Matthew Schmerer, Ben L. Rambo-Martin, Kristine Lacek, Sam Shepard, Alison Laufer Halpin, Dave Wentworth, Vivien Dugan, Suxiang Tong, Justin Lee
hCoV-19/USA/AK-CDC-2-4043704/2021	EPI_ISL_1446665	3/8/2021	AK State Public Health Lab, State Health Department	Centers for Disease Control and Prevention Division of Viral Diseases, Pathogen Discovery	Mili Sheth, Sarah Nobles, Jasmine Padilla, Mark Burroughs, Shoshona Le, Katie Dillon, Peter Cook, Clinton R. Paden, Dhwani Batra, Krista Queen, Kristen Knipe, Dakota Howard, Yvette Unoarumhi, Darlene Wagner, Matthew Schmerer, Ben L. Rambo-Martin, Kristine Lacek, Sam Shepard, Alison Laufer Halpin, Dave Wentworth, Vivien Dugan, Suxiang Tong, Justin Lee
hCoV-19/USA/AK-CDC-2-4043823/2021	EPI_ISL_1446666	3/6/2021	AK State Public Health Lab, State Health Department	Centers for Disease Control and Prevention Division of Viral Diseases, Pathogen Discovery	Mili Sheth, Sarah Nobles, Jasmine Padilla, Mark Burroughs, Shoshona Le, Katie Dillon, Peter Cook, Clinton R. Paden, Dhwani Batra, Krista Queen, Kristen Knipe, Dakota Howard, Yvette Unoarumhi, Darlene Wagner, Matthew Schmerer, Ben L. Rambo-Martin, Kristine Lacek, Sam Shepard, Alison Laufer Halpin, Dave Wentworth, Vivien Dugan, Suxiang Tong, Justin Lee
hCoV-19/USA/AK-CDC-2-4043734/2021	EPI_ISL_1446671	3/7/2021	AK State Public Health Lab, State Health Department	Centers for Disease Control and Prevention Division of Viral Diseases, Pathogen Discovery	Mili Sheth, Sarah Nobles, Jasmine Padilla, Mark Burroughs, Shoshona Le, Katie Dillon, Peter Cook, Clinton R. Paden, Dhwani Batra, Krista Queen, Kristen Knipe, Dakota Howard, Yvette Unoarumhi, Darlene Wagner, Matthew Schmerer, Ben L. Rambo-Martin, Kristine Lacek, Sam Shepard, Alison Laufer Halpin, Dave Wentworth, Vivien Dugan, Suxiang Tong, Justin Lee
hCoV-19/USA/AK-CDC-ASC210006353/2021	EPI_ISL_1479635	3/14/2021	Aegis Sciences Corporation	Centers for Disease Control and Prevention Division of Viral Diseases, Pathogen Discovery	Dakota Howard, Dhwani Batra, Peter W. Cook, Kara Moser, Adrian Paskey, Jason Caravas, Benjamin Rambo-Martin, Shatavia Morrison, Christopher Gulvick, Scott Sammons, Yvette Unoarumhi, Darlene Wagner, Matthew Schmerer, Cyndi Clark, Patrick Campbell, Rob Case, Vikramsinha Ghorpade, Holly Houdeshell, Ola Kvalvaag, Dillon Nall, Ethan Sanders, Alec Vest, Shaun Westlund, Matthew Hardison, Clinton R. Paden, Duncan MacCannell
hCoV-19/USA/AK-CDC-2-4043735/2021	EPI_ISL_1446663	3/1/2021	AK State Public Health Lab, State Health Department	Centers for Disease Control and Prevention Division of Viral Diseases, Pathogen Discovery	Mili Sheth, Sarah Nobles, Jasmine Padilla, Mark Burroughs, Shoshona Le, Katie Dillon, Peter Cook, Clinton R. Paden, Dhwani Batra, Krista Queen, Kristen Knipe, Dakota Howard, Yvette Unoarumhi, Darlene Wagner, Matthew Schmerer, Ben L. Rambo-Martin, Kristine Lacek, Sam Shepard, Alison Laufer Halpin, Dave Wentworth, Vivien Dugan, Suxiang Tong, Justin Lee
hCoV-19/USA/AK-CDC-2-4043705/2021	EPI_ISL_1446652	3/6/2021	AK State Public Health Lab, State Health Department	Centers for Disease Control and Prevention Division of Viral Diseases, Pathogen Discovery	Mili Sheth, Sarah Nobles, Jasmine Padilla, Mark Burroughs, Shoshona Le, Katie Dillon, Peter Cook, Clinton R. Paden, Dhwani Batra, Krista Queen, Kristen Knipe, Dakota Howard, Yvette Unoarumhi, Darlene Wagner, Matthew Schmerer, Ben L. Rambo-Martin, Kristine Lacek, Sam Shepard, Alison Laufer Halpin, Dave Wentworth, Vivien Dugan, Suxiang Tong, Justin Lee
hCoV-19/USA/AK-CDC-2-4122930/2021	EPI_ISL_1528492	3/17/2021	AK State Public Health Lab, State Health Department	Centers for Disease Control and Prevention Division of Viral Diseases, Pathogen Discovery	Mili Sheth, Sarah Nobles, Jasmine Padilla, Mark Burroughs, Shoshona Le, Katie Dillon, Peter Cook, Clinton R. Paden, Dhwani Batra, Krista Queen, Kristen Knipe, Dakota Howard, Yvette Unoarumhi, Darlene Wagner, Matthew Schmerer, Ben L. Rambo-Martin, Kristine Lacek, Sam Shepard, Alison Laufer Halpin, Dave Wentworth, Vivien Dugan, Suxiang Tong, Justin Lee
hCoV-19/USA/AK-CDC-2-4122911/2021	EPI_ISL_1528479	3/22/2021	AK State Public Health Lab, State Health Department	Centers for Disease Control and Prevention Division of Viral Diseases, Pathogen Discovery	Mili Sheth, Sarah Nobles, Jasmine Padilla, Mark Burroughs, Shoshona Le, Katie Dillon, Peter Cook, Clinton R. Paden, Dhwani Batra, Krista Queen, Kristen Knipe, Dakota Howard, Yvette Unoarumhi, Darlene Wagner, Matthew Schmerer, Ben L. Rambo-Martin, Kristine Lacek, Sam Shepard, Alison Laufer Halpin, Dave Wentworth, Vivien Dugan, Suxiang Tong, Justin Lee

hCoV-19/USA/AK-CDC-2-4122917/2021	EPI_ISL_1528480	3/18/2021	AK State Public Health Lab, State Health Department	Centers for Disease Control and Prevention Division of Viral Diseases, Pathogen Discovery	Mili Sheth, Sarah Nobles, Jasmine Padilla, Mark Burroughs, Shoshona Le, Katie Dillon, Peter Cook, Clinton R. Paden, Dhwani Batra, Krista Queen, Kristen Knipe, Dakota Howard, Yvette Unoarumhi, Darlene Wagner, Matthew Schmerer, Ben L. Rambo-Martin, Kristine Lacek, Sam Shepard, Alison Laufer Halpin, Dave Wentworth, Vivien Dugan, Suxiang Tong, Justin Lee
hCoV-19/USA/AK-CDC-2-4123009/2021	EPI_ISL_1528481	3/23/2021	AK State Public Health Lab, State Health Department	Centers for Disease Control and Prevention Division of Viral Diseases, Pathogen Discovery	Mili Sheth, Sarah Nobles, Jasmine Padilla, Mark Burroughs, Shoshona Le, Katie Dillon, Peter Cook, Clinton R. Paden, Dhwani Batra, Krista Queen, Kristen Knipe, Dakota Howard, Yvette Unoarumhi, Darlene Wagner, Matthew Schmerer, Ben L. Rambo-Martin, Kristine Lacek, Sam Shepard, Alison Laufer Halpin, Dave Wentworth, Vivien Dugan, Suxiang Tong, Justin Lee
hCoV-19/USA/AK-CDC-2-4122829/2021	EPI_ISL_1528482	3/21/2021	AK State Public Health Lab, State Health Department	Centers for Disease Control and Prevention Division of Viral Diseases, Pathogen Discovery	Mili Sheth, Sarah Nobles, Jasmine Padilla, Mark Burroughs, Shoshona Le, Katie Dillon, Peter Cook, Clinton R. Paden, Dhwani Batra, Krista Queen, Kristen Knipe, Dakota Howard, Yvette Unoarumhi, Darlene Wagner, Matthew Schmerer, Ben L. Rambo-Martin, Kristine Lacek, Sam Shepard, Alison Laufer Halpin, Dave Wentworth, Vivien Dugan, Suxiang Tong, Justin Lee
hCoV-19/USA/AK-CDC-2-4122920/2021	EPI_ISL_1528483	3/22/2021	AK State Public Health Lab, State Health Department	Centers for Disease Control and Prevention Division of Viral Diseases, Pathogen Discovery	Mili Sheth, Sarah Nobles, Jasmine Padilla, Mark Burroughs, Shoshona Le, Katie Dillon, Peter Cook, Clinton R. Paden, Dhwani Batra, Krista Queen, Kristen Knipe, Dakota Howard, Yvette Unoarumhi, Darlene Wagner, Matthew Schmerer, Ben L. Rambo-Martin, Kristine Lacek, Sam Shepard, Alison Laufer Halpin, Dave Wentworth, Vivien Dugan, Suxiang Tong, Justin Lee
hCoV-19/USA/AK-CDC-2-4122909/2021	EPI_ISL_1528484	3/18/2021	AK State Public Health Lab, State Health Department	Centers for Disease Control and Prevention Division of Viral Diseases, Pathogen Discovery	Mili Sheth, Sarah Nobles, Jasmine Padilla, Mark Burroughs, Shoshona Le, Katie Dillon, Peter Cook, Clinton R. Paden, Dhwani Batra, Krista Queen, Kristen Knipe, Dakota Howard, Yvette Unoarumhi, Darlene Wagner, Matthew Schmerer, Ben L. Rambo-Martin, Kristine Lacek, Sam Shepard, Alison Laufer Halpin, Dave Wentworth, Vivien Dugan, Suxiang Tong, Justin Lee
hCoV-19/USA/AK-CDC-2-4122929/2021	EPI_ISL_1528485	3/22/2021	AK State Public Health Lab, State Health Department	Centers for Disease Control and Prevention Division of Viral Diseases, Pathogen Discovery	Mili Sheth, Sarah Nobles, Jasmine Padilla, Mark Burroughs, Shoshona Le, Katie Dillon, Peter Cook, Clinton R. Paden, Dhwani Batra, Krista Queen, Kristen Knipe, Dakota Howard, Yvette Unoarumhi, Darlene Wagner, Matthew Schmerer, Ben L. Rambo-Martin, Kristine Lacek, Sam Shepard, Alison Laufer Halpin, Dave Wentworth, Vivien Dugan, Suxiang Tong, Justin Lee
hCoV-19/USA/AK-CDC-2-4122833/2021	EPI_ISL_1528486	3/16/2021	AK State Public Health Lab, State Health Department	Centers for Disease Control and Prevention Division of Viral Diseases, Pathogen Discovery	Mili Sheth, Sarah Nobles, Jasmine Padilla, Mark Burroughs, Shoshona Le, Katie Dillon, Peter Cook, Clinton R. Paden, Dhwani Batra, Krista Queen, Kristen Knipe, Dakota Howard, Yvette Unoarumhi, Darlene Wagner, Matthew Schmerer, Ben L. Rambo-Martin, Kristine Lacek, Sam Shepard, Alison Laufer Halpin, Dave Wentworth, Vivien Dugan, Suxiang Tong, Justin Lee
hCoV-19/USA/AK-CDC-2-4122914/2021	EPI_ISL_1528487	3/22/2021	AK State Public Health Lab, State Health Department	Centers for Disease Control and Prevention Division of Viral Diseases, Pathogen Discovery	Mili Sheth, Sarah Nobles, Jasmine Padilla, Mark Burroughs, Shoshona Le, Katie Dillon, Peter Cook, Clinton R. Paden, Dhwani Batra, Krista Queen, Kristen Knipe, Dakota Howard, Yvette Unoarumhi, Darlene Wagner, Matthew Schmerer, Ben L. Rambo-Martin, Kristine Lacek, Sam Shepard, Alison Laufer Halpin, Dave Wentworth, Vivien Dugan, Suxiang Tong, Justin Lee
hCoV-19/USA/AK-CDC-2-4122919/2021	EPI_ISL_1528488	3/19/2021	AK State Public Health Lab, State Health Department	Centers for Disease Control and Prevention Division of Viral Diseases, Pathogen Discovery	Mili Sheth, Sarah Nobles, Jasmine Padilla, Mark Burroughs, Shoshona Le, Katie Dillon, Peter Cook, Clinton R. Paden, Dhwani Batra, Krista Queen, Kristen Knipe, Dakota Howard, Yvette Unoarumhi, Darlene Wagner, Matthew Schmerer, Ben L. Rambo-Martin, Kristine Lacek, Sam Shepard, Alison Laufer Halpin, Dave Wentworth, Vivien Dugan, Suxiang Tong, Justin Lee
hCoV-19/USA/AK-CDC-2-4122915/2021	EPI_ISL_1528489	3/23/2021	AK State Public Health Lab, State Health Department	Centers for Disease Control and Prevention Division of Viral Diseases, Pathogen Discovery	Mili Sheth, Sarah Nobles, Jasmine Padilla, Mark Burroughs, Shoshona Le, Katie Dillon, Peter Cook, Clinton R. Paden, Dhwani Batra, Krista Queen, Kristen Knipe, Dakota Howard, Yvette Unoarumhi, Darlene Wagner, Matthew Schmerer, Ben L. Rambo-Martin, Kristine Lacek, Sam Shepard, Alison Laufer Halpin, Dave Wentworth, Vivien Dugan, Suxiang Tong, Justin Lee
hCoV-19/USA/AK-CDC-2-4122830/2021	EPI_ISL_1528490	3/19/2021	AK State Public Health Lab, State Health Department	Centers for Disease Control and Prevention Division of Viral Diseases, Pathogen Discovery	Mili Sheth, Sarah Nobles, Jasmine Padilla, Mark Burroughs, Shoshona Le, Katie Dillon, Peter Cook, Clinton R. Paden, Dhwani Batra, Krista Queen, Kristen Knipe, Dakota Howard, Yvette Unoarumhi, Darlene Wagner, Matthew Schmerer, Ben L. Rambo-Martin, Kristine Lacek, Sam Shepard, Alison Laufer Halpin, Dave Wentworth, Vivien Dugan, Suxiang Tong, Justin Lee

hCoV-19/USA/AK-CDC-2-4123007/2021	EPI_ISL_1528491	3/22/2021	AK State Public Health Lab, State Health Department	Centers for Disease Control and Prevention Division of Viral Diseases, Pathogen Discovery	Mili Sheth, Sarah Nobles, Jasmine Padilla, Mark Burroughs, Shoshona Le, Katie Dillon, Peter Cook, Clinton R. Paden, Dhwani Batra, Krista Queen, Kristen Knipe, Dakota Howard, Yvette Unoarumhi, Darlene Wagner, Matthew Schmerer, Ben L. Rambo-Martin, Kristine Lacek, Sam Shepard, Alison Laufer Halpin, Dave Wentworth, Vivien Dugan, Suxiang Tong, Justin Lee
hCoV-19/USA/AK-CDC-ASC210003733/2021	EPI_ISL_1550635	3/11/2021	Aegis Sciences Corporation	Centers for Disease Control and Prevention Division of Viral Diseases, Pathogen Discovery	Dakota Howard, Dhwani Batra, Peter W. Cook, Kara Moser, Adrian Paskey, Jason Caravas, Benjamin Rambo-Martin, Shatavia Morrison, Christopher Gulvick, Scott Sammons, Yvette Unoarumhi, Darlene Wagner, Matthew Schmerer, Cyndi Clark, Patrick Campbell, Rob Case, Vikramsinha Ghorpade, Holly Houdeshell, Ola Kvalvaag, Dillon Nall, Ethan Sanders, Alec Vest, Shaun Westlund, Matthew Hardison, Clinton R. Paden, Duncan MacCannell
hCoV-19/USA/AK-CDC-ASC210005628/2021	EPI_ISL_1560222	3/13/2021	Aegis Sciences Corporation	Centers for Disease Control and Prevention Division of Viral Diseases, Pathogen Discovery	Dakota Howard, Dhwani Batra, Peter W. Cook, Kara Moser, Adrian Paskey, Jason Caravas, Benjamin Rambo-Martin, Shatavia Morrison, Christopher Gulvick, Scott Sammons, Yvette Unoarumhi, Darlene Wagner, Matthew Schmerer, Cyndi Clark, Patrick Campbell, Rob Case, Vikramsinha Ghorpade, Holly Houdeshell, Ola Kvalvaag, Dillon Nall, Ethan Sanders, Alec Vest, Shaun Westlund, Matthew Hardison, Clinton R. Paden, Duncan MacCannell
hCoV-19/USA/AK-PHL7768/2021	EPI_ISL_1575082	3/25/2021	Alaska State Virology Laboratory	Alaska State Virology Laboratory	Stephanie DeRonde, Elva House, Lisa Smith, Ph.D., Jack Chen, Ph.D.
hCoV-19/USA/AK-PHL7772/2021	EPI_ISL_1575084	3/27/2021	Alaska State Virology Laboratory	Alaska State Virology Laboratory	Stephanie DeRonde, Elva House, Lisa Smith, Ph.D., Jack Chen, Ph.D.
hCoV-19/USA/AK-PHL7773/2021	EPI_ISL_1575085	3/26/2021	Alaska State Virology Laboratory	Alaska State Virology Laboratory	Stephanie DeRonde, Elva House, Lisa Smith, Ph.D., Jack Chen, Ph.D.
hCoV-19/USA/AK-PHL7776/2021	EPI_ISL_1575087	3/26/2021	Alaska State Virology Laboratory	Alaska State Virology Laboratory	Stephanie DeRonde, Elva House, Lisa Smith, Ph.D., Jack Chen, Ph.D.
hCoV-19/USA/AK-PHL7778/2021	EPI_ISL_1575088	3/26/2021	Alaska State Virology Laboratory	Alaska State Virology Laboratory	Stephanie DeRonde, Elva House, Lisa Smith, Ph.D., Jack Chen, Ph.D.
hCoV-19/USA/AK-PHL7779/2021	EPI_ISL_1575089	3/26/2021	Alaska State Virology Laboratory	Alaska State Virology Laboratory	Stephanie DeRonde, Elva House, Lisa Smith, Ph.D., Jack Chen, Ph.D.
hCoV-19/USA/AK-PHL7780/2021	EPI_ISL_1575090	3/26/2021	Alaska State Virology Laboratory	Alaska State Virology Laboratory	Stephanie DeRonde, Elva House, Lisa Smith, Ph.D., Jack Chen, Ph.D.
hCoV-19/USA/AK-PHL7785/2021	EPI_ISL_1575093	3/26/2021	Alaska State Virology Laboratory	Alaska State Virology Laboratory	Stephanie DeRonde, Elva House, Lisa Smith, Ph.D., Jack Chen, Ph.D.
hCoV-19/USA/AK-PHL7787/2021	EPI_ISL_1575095	3/25/2021	Alaska State Virology Laboratory	Alaska State Virology Laboratory	Stephanie DeRonde, Elva House, Lisa Smith, Ph.D., Jack Chen, Ph.D.
hCoV-19/USA/AK-PHL7793/2021	EPI_ISL_1575098	3/26/2021	Alaska State Virology Laboratory	Alaska State Virology Laboratory	Stephanie DeRonde, Elva House, Lisa Smith, Ph.D., Jack Chen, Ph.D.
hCoV-19/USA/AK-PHL7807/2021	EPI_ISL_1575106	3/25/2021	Alaska State Virology Laboratory	Alaska State Virology Laboratory	Stephanie DeRonde, Elva House, Lisa Smith, Ph.D., Jack Chen, Ph.D.
hCoV-19/USA/AK-PHL7808/2021	EPI_ISL_1575107	3/25/2021	Alaska State Virology Laboratory	Alaska State Virology Laboratory	Stephanie DeRonde, Elva House, Lisa Smith, Ph.D., Jack Chen, Ph.D.
hCoV-19/USA/AK-PHL7809/2021	EPI_ISL_1575108	3/30/2021	Alaska State Virology Laboratory	Alaska State Virology Laboratory	Stephanie DeRonde, Elva House, Lisa Smith, Ph.D., Jack Chen, Ph.D.
hCoV-19/USA/AK-PHL7810/2021	EPI_ISL_1575109	3/30/2021	Alaska State Virology Laboratory	Alaska State Virology Laboratory	Stephanie DeRonde, Elva House, Lisa Smith, Ph.D., Jack Chen, Ph.D.
hCoV-19/USA/AK-PHL7811/2021	EPI_ISL_1575110	3/30/2021	Alaska State Virology Laboratory	Alaska State Virology Laboratory	Stephanie DeRonde, Elva House, Lisa Smith, Ph.D., Jack Chen, Ph.D.
hCoV-19/USA/AK-PHL7812/2021	EPI_ISL_1575111	3/30/2021	Alaska State Virology Laboratory	Alaska State Virology Laboratory	Stephanie DeRonde, Elva House, Lisa Smith, Ph.D., Jack Chen, Ph.D.
hCoV-19/USA/AK-CDC-FG-011939/2021	EPI_ISL_1555564	3/7/2021	Fulgent Genetics	Centers for Disease Control and Prevention Division of Viral Diseases, Pathogen Discovery	Dakota Howard, Dhwani Batra, Peter W. Cook, Kara Moser, Adrian Paskey, Jason Caravas, Benjamin Rambo-Martin, Shatavia Morrison, Christopher Gulvick, Scott Sammons, Yvette Unoarumhi, Darlene Wagner, Matthew Schmerer, Harry Gao, Mickey Li, John Gao, Joseph Fierro, Benafsh Sapra, Becky Tsai, Yan Meng, Doreen Ng, James Xie, Clinton R. Paden, Duncan MacCannell
hCoV-19/USA/AK-PHL6651/2020	EPI_ISL_806829	11/29/2020	Alaska State Virology Laboratory	Alaska State Virology Laboratory	Stephanie DeRonde, Lisa Smith, Ph.D., Devin M. Drown, Ph.D., Jack Chen, Ph.D.
hCoV-19/USA/AK-PHL6652/2020	EPI_ISL_806830	11/30/2020	Alaska State Virology Laboratory	Alaska State Virology Laboratory	Stephanie DeRonde, Lisa Smith, Ph.D., Devin M. Drown, Ph.D., Jack Chen, Ph.D.
hCoV-19/USA/AK-PHL6653/2020	EPI_ISL_806831	11/30/2020	Alaska State Virology Laboratory	Alaska State Virology Laboratory	Stephanie DeRonde, Lisa Smith, Ph.D., Devin M. Drown, Ph.D., Jack Chen, Ph.D.
hCoV-19/USA/AK-PHL6656/2020	EPI_ISL_806834	12/1/2020	Alaska State Virology Laboratory	Alaska State Virology Laboratory	Stephanie DeRonde, Lisa Smith, Ph.D., Devin M. Drown, Ph.D., Jack Chen, Ph.D.
hCoV-19/USA/AK-PHL6657/2020	EPI_ISL_806835	12/1/2020	Alaska State Virology Laboratory	Alaska State Virology Laboratory	Stephanie DeRonde, Lisa Smith, Ph.D., Devin M. Drown, Ph.D., Jack Chen, Ph.D.
hCoV-19/USA/AK-PHL6658/2020	EPI_ISL_806836	12/2/2020	Alaska State Virology Laboratory	Alaska State Virology Laboratory	Stephanie DeRonde, Lisa Smith, Ph.D., Devin M. Drown, Ph.D., Jack Chen, Ph.D.
hCoV-19/USA/AK-PHL6660/2020	EPI_ISL_806838	12/3/2020	Alaska State Virology Laboratory	Alaska State Virology Laboratory	Stephanie DeRonde, Lisa Smith, Ph.D., Devin M. Drown, Ph.D., Jack Chen, Ph.D.
hCoV-19/USA/AK-PHL6661/2020	EPI_ISL_806839	12/3/2020	Alaska State Virology Laboratory	Alaska State Virology Laboratory	Stephanie DeRonde, Lisa Smith, Ph.D., Devin M. Drown, Ph.D., Jack Chen, Ph.D.
hCoV-19/USA/AK-PHL6662/2020	EPI_ISL_806840	11/30/2020	Alaska State Virology Laboratory	Alaska State Virology Laboratory	Stephanie DeRonde, Lisa Smith, Ph.D., Devin M. Drown, Ph.D., Jack Chen, Ph.D.
hCoV-19/USA/AK-PHL6663/2020	EPI_ISL_806841	12/6/2020	Alaska State Virology Laboratory	Alaska State Virology Laboratory	Stephanie DeRonde, Lisa Smith, Ph.D., Devin M. Drown, Ph.D., Jack Chen, Ph.D.
hCoV-19/USA/AK-PHL6665/2020	EPI_ISL_806843	12/8/2020	Alaska State Virology Laboratory	Alaska State Virology Laboratory	Stephanie DeRonde, Lisa Smith, Ph.D., Devin M. Drown, Ph.D., Jack Chen, Ph.D.
hCoV-19/USA/AK-PHL6666/2020	EPI_ISL_806844	12/8/2020	Alaska State Virology Laboratory	Alaska State Virology Laboratory	Stephanie DeRonde, Lisa Smith, Ph.D., Devin M. Drown, Ph.D., Jack Chen, Ph.D.
hCoV-19/USA/AK-PHL6669/2020	EPI_ISL_806847	12/2/2020	Alaska State Virology Laboratory	Alaska State Virology Laboratory	Stephanie DeRonde, Lisa Smith, Ph.D., Devin M. Drown, Ph.D., Jack Chen, Ph.D.
hCoV-19/USA/AK-PHL6670/2020	EPI_ISL_806848	12/12/2020	Alaska State Virology Laboratory	Alaska State Virology Laboratory	Stephanie DeRonde, Lisa Smith, Ph.D., Devin M. Drown, Ph.D., Jack Chen, Ph.D.

hCoV-19/USA/AK-PHL6671/2020	EPI_ISL_806849	12/13/2020	Alaska State Virology Laboratory	Alaska State Virology Laboratory	Stephanie DeRonde, Lisa Smith, Ph.D., Devin M. Drown, Ph.D., Jack Chen, Ph.D.
hCoV-19/USA/AK-PHL6673/2020	EPI_ISL_806851	12/15/2020	Alaska State Virology Laboratory	Alaska State Virology Laboratory	Stephanie DeRonde, Lisa Smith, Ph.D., Devin M. Drown, Ph.D., Jack Chen, Ph.D.
hCoV-19/USA/AK-PHL6675/2020	EPI_ISL_806852	12/17/2020	Alaska State Virology Laboratory	Alaska State Virology Laboratory	Stephanie DeRonde, Lisa Smith, Ph.D., Devin M. Drown, Ph.D., Jack Chen, Ph.D.
hCoV-19/USA/AK-QDX-3198/2021	EPI_ISL_855014	1/8/2021	Quest Diagnostics	Quest Diagnostics	Rosenthal,S.H., Gerasimova,A., Kagan,R.M., Anderson, B., Hua, M., Liu Y., Bernstein, L.E., Livingston, K.E., Perez, A., Shalhout, D.F., Shlyakhter, I.A., Owen, R., Tanpaiboon, P., Lacbawan, F.
hCoV-19/USA/AK-PHL6606/2020	EPI_ISL_872349	12/20/2020	Alaska State Virology Lab	Alaska State Virology Lab	Stephanie DeRonde, Lisa Smith, Ph.D., Jack Chen, Ph.D.
hCoV-19/USA/AK-PHL6568/2020	EPI_ISL_884214	12/10/2020	Alaska State Virology Laboratory	Alaska State Virology Laboratory	Stephanie DeRonde, Lisa Smith, PhD. Jack Chen, PhD.
hCoV-19/USA/AK-PHL6567/2020	EPI_ISL_884215	12/5/2020	Alaska State Virology Laboratory	Alaska State Virology Laboratory	Stephanie DeRonde, Lisa Smith, PhD. Jack Chen, PhD.
hCoV-19/USA/AK-PHL6542/2020	EPI_ISL_884216	12/1/2020	Alaska State Virology Laboratory	Alaska State Virology Laboratory	Stephanie DeRonde, Lisa Smith, PhD. Jack Chen, PhD.
hCoV-19/USA/AK-PHL6562/2020	EPI_ISL_884217	12/7/2020	Alaska State Virology Laboratory	Alaska State Virology Laboratory	Stephanie DeRonde, Lisa Smith, PhD. Jack Chen, PhD.
hCoV-19/USA/AK-PHL6565/2020	EPI_ISL_884218	12/7/2020	Alaska State Virology Laboratory	Alaska State Virology Laboratory	Stephanie DeRonde, Lisa Smith, PhD. Jack Chen, PhD.
hCoV-19/USA/AK-PHL6560/2020	EPI_ISL_884219	12/2/2020	Alaska State Virology Laboratory	Alaska State Virology Laboratory	Stephanie DeRonde, Lisa Smith, PhD. Jack Chen, PhD.
hCoV-19/USA/AK-PHL6549/2020	EPI_ISL_884220	11/30/2020	Alaska State Virology Laboratory	Alaska State Virology Laboratory	Stephanie DeRonde, Lisa Smith, PhD. Jack Chen, PhD.
hCoV-19/USA/AK-PHL6584/2020	EPI_ISL_911677	11/30/2020	Alaska State Virology Laboratory	Alaska State Virology Laboratory	Stephanie DeRonde, Lisa Smith, Ph.D., Jack Chen, Ph.D.
hCoV-19/USA/AK-PHL6587/2020	EPI_ISL_911680	11/30/2020	Alaska State Virology Laboratory	Alaska State Virology Laboratory	Stephanie DeRonde, Lisa Smith, Ph.D., Jack Chen, Ph.D.
hCoV-19/USA/AK-PHL6588/2020	EPI_ISL_911681	12/8/2020	Alaska State Virology Laboratory	Alaska State Virology Laboratory	Stephanie DeRonde, Lisa Smith, Ph.D., Jack Chen, Ph.D.
hCoV-19/USA/AK-PHL6589/2020	EPI_ISL_911682	12/2/2020	Alaska State Virology Laboratory	Alaska State Virology Laboratory	Stephanie DeRonde, Lisa Smith, Ph.D., Jack Chen, Ph.D.
hCoV-19/USA/AK-PHL6591/2020	EPI_ISL_911684	12/14/2020	Alaska State Virology Laboratory	Alaska State Virology Laboratory	Stephanie DeRonde, Lisa Smith, Ph.D., Jack Chen, Ph.D.
hCoV-19/USA/AK-PHL6602/2020	EPI_ISL_911686	12/3/2020	Alaska State Virology Laboratory	Alaska State Virology Laboratory	Stephanie DeRonde, Lisa Smith, Ph.D., Jack Chen, Ph.D.
hCoV-19/USA/AK-PHL6608/2020	EPI_ISL_911688	12/7/2020	Alaska State Virology Laboratory	Alaska State Virology Laboratory	Stephanie DeRonde, Lisa Smith, Ph.D., Jack Chen, Ph.D.
hCoV-19/USA/AK-PHL6609/2020	EPI_ISL_911689	12/21/2020	Alaska State Virology Laboratory	Alaska State Virology Laboratory	Stephanie DeRonde, Lisa Smith, Ph.D., Jack Chen, Ph.D.
hCoV-19/USA/AK-PHL6610/2020	EPI_ISL_911690	12/3/2020	Alaska State Virology Laboratory	Alaska State Virology Laboratory	Stephanie DeRonde, Lisa Smith, Ph.D., Jack Chen, Ph.D.
hCoV-19/USA/AK-PHL6611/2020	EPI_ISL_911691	12/7/2020	Alaska State Virology Laboratory	Alaska State Virology Laboratory	Stephanie DeRonde, Lisa Smith, Ph.D., Jack Chen, Ph.D.
hCoV-19/USA/AK-PHL6614/2020	EPI_ISL_911692	12/8/2020	Alaska State Virology Laboratory	Alaska State Virology Laboratory	Stephanie DeRonde, Lisa Smith, Ph.D., Jack Chen, Ph.D.
hCoV-19/USA/AK-PHL6615/2020	EPI_ISL_911693	12/15/2020	Alaska State Virology Laboratory	Alaska State Virology Laboratory	Stephanie DeRonde, Lisa Smith, Ph.D., Jack Chen, Ph.D.
hCoV-19/USA/AK-PHL6616/2020	EPI_ISL_911694	12/13/2020	Alaska State Virology Laboratory	Alaska State Virology Laboratory	Stephanie DeRonde, Lisa Smith, Ph.D., Jack Chen, Ph.D.
hCoV-19/USA/AK-PHL6620/2020	EPI_ISL_911695	12/14/2020	Alaska State Virology Laboratory	Alaska State Virology Laboratory	Stephanie DeRonde, Lisa Smith, Ph.D., Jack Chen, Ph.D.
hCoV-19/USA/AK-PHL6592/2020	EPI_ISL_911698	12/12/2020	Alaska State Virology Laboratory	Alaska State Virology Laboratory	Stephanie DeRonde, Lisa Smith, Ph.D., Jack Chen, Ph.D.
hCoV-19/USA/AK-PHL6596/2020	EPI_ISL_911699	12/14/2020	Alaska State Virology Laboratory	Alaska State Virology Laboratory	Stephanie DeRonde, Lisa Smith, Ph.D., Jack Chen, Ph.D.
hCoV-19/USA/AK-PHL6598/2020	EPI_ISL_911700	12/2/2020	Alaska State Virology Laboratory	Alaska State Virology Laboratory	Stephanie DeRonde, Lisa Smith, Ph.D., Jack Chen, Ph.D.
hCoV-19/USA/AK-PHL6617/2020	EPI_ISL_911702	12/22/2020	Alaska State Virology Laboratory	Alaska State Virology Laboratory	Stephanie DeRonde, Lisa Smith, Ph.D., Jack Chen, Ph.D.
hCoV-19/USA/AK-PHL6622/2020	EPI_ISL_911703	11/30/2020	Alaska State Virology Laboratory	Alaska State Virology Laboratory	Stephanie DeRonde, Lisa Smith, Ph.D., Jack Chen, Ph.D.
hCoV-19/USA/AK-PHL7994/2021	EPI_ISL_1653901	4/7/2021	Alaska State Virology Laboratory	Alaska State Virology Laboratory	Stephanie DeRonde, Elva House, Lisa Smith, Ph.D., Jack Chen, Ph.D.
hCoV-19/USA/AK-PHL7995/2021	EPI_ISL_1653902	4/7/2021	Alaska State Virology Laboratory	Alaska State Virology Laboratory	Stephanie DeRonde, Elva House, Lisa Smith, Ph.D., Jack Chen, Ph.D.
hCoV-19/USA/AK-PHL7999/2021	EPI_ISL_1653905	4/7/2021	Alaska State Virology Laboratory	Alaska State Virology Laboratory	Stephanie DeRonde, Elva House, Lisa Smith, Ph.D., Jack Chen, Ph.D.
hCoV-19/USA/AK-RD-RA03204/2021	EPI_ISL_1032951	1/29/2021	Fulgent Genetics	Fulgent Genetics	Harry Gao, Mickey Li, John Gao, Joseph Fierro, Benafsh Sapra, Becky Tsai, Yan Meng, Doreen Ng, James Xie
hCoV-19/USA/AK-PHL6819/2021	EPI_ISL_1039712	1/25/2021	Alaska State Virology Laboratory	Alaska State Virology Laboratory	Stephanie DeRonde, Lisa Smith, Ph.D., Jack Chen, Ph.D.
hCoV-19/USA/AK-PHL6828/2021	EPI_ISL_1039713	1/26/2021	Alaska State Virology Laboratory	Alaska State Virology Laboratory	Stephanie DeRonde, Lisa Smith, Ph.D., Jack Chen, Ph.D.
hCoV-19/USA/AK-PHL6829/2021	EPI_ISL_1039714	1/27/2021	Alaska State Virology Laboratory	Alaska State Virology Laboratory	Stephanie DeRonde, Lisa Smith, Ph.D., Jack Chen, Ph.D.
hCoV-19/USA/AK-PHL6814/2021	EPI_ISL_1039715	2/6/2021	Alaska State Virology Laboratory	Alaska State Virology Laboratory	Stephanie DeRonde, Lisa Smith, Ph.D., Jack Chen, Ph.D.
hCoV-19/USA/AK-PHL6813/2021	EPI_ISL_1039716	2/3/2021	Alaska State Virology Laboratory	Alaska State Virology Laboratory	Stephanie DeRonde, Lisa Smith, Ph.D., Jack Chen, Ph.D.
hCoV-19/USA/AK-PHL6867/2021	EPI_ISL_1039717	1/6/2021	Alaska State Virology Laboratory	Alaska State Virology Laboratory	Stephanie DeRonde, Lisa Smith, Ph.D., Jack Chen, Ph.D.
hCoV-19/USA/AK-PHL6801/2021	EPI_ISL_1039718	1/29/2021	Alaska State Virology Laboratory	Alaska State Virology Laboratory	Stephanie DeRonde, Lisa Smith, Ph.D., Jack Chen, Ph.D.
hCoV-19/USA/AK-PHL6802/2021	EPI_ISL_1039719	1/29/2021	Alaska State Virology Laboratory	Alaska State Virology Laboratory	Stephanie DeRonde, Lisa Smith, Ph.D., Jack Chen, Ph.D.
hCoV-19/USA/AK-PHL6800/2021	EPI_ISL_1039720	1/30/2021	Alaska State Virology Laboratory	Alaska State Virology Laboratory	Stephanie DeRonde, Lisa Smith, Ph.D., Jack Chen, Ph.D.
hCoV-19/USA/AK-PHL6799/2021	EPI_ISL_1039721	1/31/2021	Alaska State Virology Laboratory	Alaska State Virology Laboratory	Stephanie DeRonde, Lisa Smith, Ph.D., Jack Chen, Ph.D.
hCoV-19/USA/AK-PHL6812/2021	EPI_ISL_1039722	2/4/2021	Alaska State Virology Laboratory	Alaska State Virology Laboratory	Stephanie DeRonde, Lisa Smith, Ph.D., Jack Chen, Ph.D.
hCoV-19/USA/AK-PHL6815/2021	EPI_ISL_1039723	2/6/2021	Alaska State Virology Laboratory	Alaska State Virology Laboratory	Stephanie DeRonde, Lisa Smith, Ph.D., Jack Chen, Ph.D.
hCoV-19/USA/AK-PHL6869/2020	EPI_ISL_1039724	12/31/2020	Alaska State Virology Laboratory	Alaska State Virology Laboratory	Stephanie DeRonde, Lisa Smith, Ph.D., Jack Chen, Ph.D.
hCoV-19/USA/AK-PHL6868/2021	EPI_ISL_1039725	1/13/2021	Alaska State Virology Laboratory	Alaska State Virology Laboratory	Stephanie DeRonde, Lisa Smith, Ph.D., Jack Chen, Ph.D.

hCoV-19/USA/AK-PHL6866/2021	EPI_ISL_1039726	1/23/2021	Alaska State Virology Laboratory	Alaska State Virology Laboratory	Stephanie DeRonde, Lisa Smith, Ph.D., Jack Chen, Ph.D.
hCoV-19/USA/AK-PHL6836/2021	EPI_ISL_1039727	1/8/2021	Alaska State Virology Laboratory	Alaska State Virology Laboratory	Stephanie DeRonde, Lisa Smith, Ph.D., Jack Chen, Ph.D.
hCoV-19/USA/AK-PHL6837/2021	EPI_ISL_1039728	1/8/2021	Alaska State Virology Laboratory	Alaska State Virology Laboratory	Stephanie DeRonde, Lisa Smith, Ph.D., Jack Chen, Ph.D.
hCoV-19/USA/AK-PHL6832/2021	EPI_ISL_1039729	1/19/2021	Alaska State Virology Laboratory	Alaska State Virology Laboratory	Stephanie DeRonde, Lisa Smith, Ph.D., Jack Chen, Ph.D.
hCoV-19/USA/AK-PHL6833/2021	EPI_ISL_1039730	1/19/2021	Alaska State Virology Laboratory	Alaska State Virology Laboratory	Stephanie DeRonde, Lisa Smith, Ph.D., Jack Chen, Ph.D.
hCoV-19/USA/AK-PHL6834/2021	EPI_ISL_1039731	1/19/2021	Alaska State Virology Laboratory	Alaska State Virology Laboratory	Stephanie DeRonde, Lisa Smith, Ph.D., Jack Chen, Ph.D.
hCoV-19/USA/AK-PHL6817/2021	EPI_ISL_1039732	1/27/2021	Alaska State Virology Laboratory	Alaska State Virology Laboratory	Stephanie DeRonde, Lisa Smith, Ph.D., Jack Chen, Ph.D.
hCoV-19/USA/AK-PHL6818/2021	EPI_ISL_1039733	1/27/2021	Alaska State Virology Laboratory	Alaska State Virology Laboratory	Stephanie DeRonde, Lisa Smith, Ph.D., Jack Chen, Ph.D.
hCoV-19/USA/AK-PHL6825/2021	EPI_ISL_1039735	1/27/2021	Alaska State Virology Laboratory	Alaska State Virology Laboratory	Stephanie DeRonde, Lisa Smith, Ph.D., Jack Chen, Ph.D.
hCoV-19/USA/AK-PHL6768/2021	EPI_ISL_1049483	1/23/2021	Alaska State Virology Laboratory	Alaska State Virology Laboratory	Stephanie DeRonde, Lisa Smith, Ph.D., Jack Chen, Ph.D.
hCoV-19/USA/AK-PHL6720/2021	EPI_ISL_1049484	1/17/2021	Alaska State Virology Laboratory	Alaska State Virology Laboratory	Stephanie DeRonde, Lisa Smith, Ph.D., Jack Chen, Ph.D.
hCoV-19/USA/AK-PHL6771/2021	EPI_ISL_1049485	1/29/2021	Alaska State Virology Laboratory	Alaska State Virology Laboratory	Stephanie DeRonde, Lisa Smith, Ph.D., Jack Chen, Ph.D.
hCoV-19/USA/AK-PHL6794/2021	EPI_ISL_1049486	1/5/2021	Alaska State Virology Laboratory	Alaska State Virology Laboratory	Stephanie DeRonde, Lisa Smith, Ph.D., Jack Chen, Ph.D.
hCoV-19/USA/AK-PHL6779/2021	EPI_ISL_1049487	2/1/2021	Alaska State Virology Laboratory	Alaska State Virology Laboratory	Stephanie DeRonde, Lisa Smith, Ph.D., Jack Chen, Ph.D.
hCoV-19/USA/AK-PHL6780/2021	EPI_ISL_1049488	1/31/2021	Alaska State Virology Laboratory	Alaska State Virology Laboratory	Stephanie DeRonde, Lisa Smith, Ph.D., Jack Chen, Ph.D.
hCoV-19/USA/AK-PHL6781/2021	EPI_ISL_1049489	1/31/2021	Alaska State Virology Laboratory	Alaska State Virology Laboratory	Stephanie DeRonde, Lisa Smith, Ph.D., Jack Chen, Ph.D.
hCoV-19/USA/AK-PHL6769/2021	EPI_ISL_1049490	1/29/2021	Alaska State Virology Laboratory	Alaska State Virology Laboratory	Stephanie DeRonde, Lisa Smith, Ph.D., Jack Chen, Ph.D.
hCoV-19/USA/AK-PHL6770/2021	EPI_ISL_1049491	1/29/2021	Alaska State Virology Laboratory	Alaska State Virology Laboratory	Stephanie DeRonde, Lisa Smith, Ph.D., Jack Chen, Ph.D.
hCoV-19/USA/AK-PHL6776/2021	EPI_ISL_1049492	1/29/2021	Alaska State Virology Laboratory	Alaska State Virology Laboratory	Stephanie DeRonde, Lisa Smith, Ph.D., Jack Chen, Ph.D.
hCoV-19/USA/AK-PHL6767/2021	EPI_ISL_1049493	1/28/2021	Alaska State Virology Laboratory	Alaska State Virology Laboratory	Stephanie DeRonde, Lisa Smith, Ph.D., Jack Chen, Ph.D.
hCoV-19/USA/AK-PHL6783/2021	EPI_ISL_1049494	1/28/2021	Alaska State Virology Laboratory	Alaska State Virology Laboratory	Stephanie DeRonde, Lisa Smith, Ph.D., Jack Chen, Ph.D.
hCoV-19/USA/AK-PHL6773/2021	EPI_ISL_1049495	1/27/2021	Alaska State Virology Laboratory	Alaska State Virology Laboratory	Stephanie DeRonde, Lisa Smith, Ph.D., Jack Chen, Ph.D.
hCoV-19/USA/AK-PHL6763/2021	EPI_ISL_1049496	1/26/2021	Alaska State Virology Laboratory	Alaska State Virology Laboratory	Stephanie DeRonde, Lisa Smith, Ph.D., Jack Chen, Ph.D.
hCoV-19/USA/AK-PHL6765/2021	EPI_ISL_1049497	1/26/2021	Alaska State Virology Laboratory	Alaska State Virology Laboratory	Stephanie DeRonde, Lisa Smith, Ph.D., Jack Chen, Ph.D.
hCoV-19/USA/AK-PHL6774/2021	EPI_ISL_1049498	1/26/2021	Alaska State Virology Laboratory	Alaska State Virology Laboratory	Stephanie DeRonde, Lisa Smith, Ph.D., Jack Chen, Ph.D.
hCoV-19/USA/AK-PHL6782/2021	EPI_ISL_1049499	1/26/2021	Alaska State Virology Laboratory	Alaska State Virology Laboratory	Stephanie DeRonde, Lisa Smith, Ph.D., Jack Chen, Ph.D.
hCoV-19/USA/AK-PHL6775/2021	EPI_ISL_1049500	1/25/2021	Alaska State Virology Laboratory	Alaska State Virology Laboratory	Stephanie DeRonde, Lisa Smith, Ph.D., Jack Chen, Ph.D.
hCoV-19/USA/AK-PHL6777/2021	EPI_ISL_1049501	1/25/2021	Alaska State Virology Laboratory	Alaska State Virology Laboratory	Stephanie DeRonde, Lisa Smith, Ph.D., Jack Chen, Ph.D.
hCoV-19/USA/AK-PHL6778/2021	EPI_ISL_1049502	1/25/2021	Alaska State Virology Laboratory	Alaska State Virology Laboratory	Stephanie DeRonde, Lisa Smith, Ph.D., Jack Chen, Ph.D.
hCoV-19/USA/AK-PHL6784/2021	EPI_ISL_1049503	1/22/2021	Alaska State Virology Laboratory	Alaska State Virology Laboratory	Stephanie DeRonde, Lisa Smith, Ph.D., Jack Chen, Ph.D.
hCoV-19/USA/AK-PHL6791/2021	EPI_ISL_1049504	1/8/2021	Alaska State Virology Laboratory	Alaska State Virology Laboratory	Stephanie DeRonde, Lisa Smith, Ph.D., Jack Chen, Ph.D.
hCoV-19/USA/AK-PHL6793/2021	EPI_ISL_1049505	1/6/2021	Alaska State Virology Laboratory	Alaska State Virology Laboratory	Stephanie DeRonde, Lisa Smith, Ph.D., Jack Chen, Ph.D.
hCoV-19/USA/AK-PHL6797/2021	EPI_ISL_1049506	1/6/2021	Alaska State Virology Laboratory	Alaska State Virology Laboratory	Stephanie DeRonde, Lisa Smith, Ph.D., Jack Chen, Ph.D.
hCoV-19/USA/AK-PHL6788/2021	EPI_ISL_1049507	1/5/2021	Alaska State Virology Laboratory	Alaska State Virology Laboratory	Stephanie DeRonde, Lisa Smith, Ph.D., Jack Chen, Ph.D.
hCoV-19/USA/AK-PHL6789/2020	EPI_ISL_1049508	12/30/2020	Alaska State Virology Laboratory	Alaska State Virology Laboratory	Stephanie DeRonde, Lisa Smith, Ph.D., Jack Chen, Ph.D.
hCoV-19/USA/AK-PHL6786/2020	EPI_ISL_1049509	12/29/2020	Alaska State Virology Laboratory	Alaska State Virology Laboratory	Stephanie DeRonde, Lisa Smith, Ph.D., Jack Chen, Ph.D.
hCoV-19/USA/AK-PHL6581/2020	EPI_ISL_1049510	12/11/2020	Alaska State Virology Laboratory	Alaska State Virology Laboratory	Stephanie DeRonde, Lisa Smith, Ph.D., Jack Chen, Ph.D.
hCoV-19/USA/AK-PHL6888/2021	EPI_ISL_1061279	2/6/2021	Alaska State Virology Laboratory	Alaska State Virology Laboratory	Stephanie DeRonde, Lisa Smith, Ph.D., Jack Chen, Ph.D.
hCoV-19/USA/AK-PHL6889/2021	EPI_ISL_1061280	2/6/2021	Alaska State Virology Laboratory	Alaska State Virology Laboratory	Stephanie DeRonde, Lisa Smith, Ph.D., Jack Chen, Ph.D.
hCoV-19/USA/AK-PHL6890/2021	EPI_ISL_1061281	2/7/2021	Alaska State Virology Laboratory	Alaska State Virology Laboratory	Stephanie DeRonde, Lisa Smith, Ph.D., Jack Chen, Ph.D.
hCoV-19/USA/AK-PHL6891/2021	EPI_ISL_1061282	2/8/2021	Alaska State Virology Laboratory	Alaska State Virology Laboratory	Stephanie DeRonde, Lisa Smith, Ph.D., Jack Chen, Ph.D.
hCoV-19/USA/AK-PHL6892/2021	EPI_ISL_1061283	2/7/2021	Alaska State Virology Laboratory	Alaska State Virology Laboratory	Stephanie DeRonde, Lisa Smith, Ph.D., Jack Chen, Ph.D.
hCoV-19/USA/AK-PHL6893/2021	EPI_ISL_1061284	2/8/2021	Alaska State Virology Laboratory	Alaska State Virology Laboratory	Stephanie DeRonde, Lisa Smith, Ph.D., Jack Chen, Ph.D.
hCoV-19/USA/AK-PHL6894/2021	EPI_ISL_1061285	2/9/2021	Alaska State Virology Laboratory	Alaska State Virology Laboratory	Stephanie DeRonde, Lisa Smith, Ph.D., Jack Chen, Ph.D.
hCoV-19/USA/AK-PHL6897/2021	EPI_ISL_1061286	2/8/2021	Alaska State Virology Laboratory	Alaska State Virology Laboratory	Stephanie DeRonde, Lisa Smith, Ph.D., Jack Chen, Ph.D.
hCoV-19/USA/AK-PHL6899/2021	EPI_ISL_1061287	2/8/2021	Alaska State Virology Laboratory	Alaska State Virology Laboratory	Stephanie DeRonde, Lisa Smith, Ph.D., Jack Chen, Ph.D.
hCoV-19/USA/AK-PHL6900/2021	EPI_ISL_1061288	2/4/2021	Alaska State Virology Laboratory	Alaska State Virology Laboratory	Stephanie DeRonde, Lisa Smith, Ph.D., Jack Chen, Ph.D.
hCoV-19/USA/AK-PHL6901/2021	EPI_ISL_1061289	2/8/2021	Alaska State Virology Laboratory	Alaska State Virology Laboratory	Stephanie DeRonde, Lisa Smith, Ph.D., Jack Chen, Ph.D.
hCoV-19/USA/AK-PHL6902/2021	EPI_ISL_1061290	2/6/2021	Alaska State Virology Laboratory	Alaska State Virology Laboratory	Stephanie DeRonde, Lisa Smith, Ph.D., Jack Chen, Ph.D.
hCoV-19/USA/AK-PHL6903/2021	EPI_ISL_1061291	2/8/2021	Alaska State Virology Laboratory	Alaska State Virology Laboratory	Stephanie DeRonde, Lisa Smith, Ph.D., Jack Chen, Ph.D.
hCoV-19/USA/AK-PHL6904/2021	EPI_ISL_1061292	2/8/2021	Alaska State Virology Laboratory	Alaska State Virology Laboratory	Stephanie DeRonde, Lisa Smith, Ph.D., Jack Chen, Ph.D.

hCoV-19/USA/AK-PHL6905/2021	EPI_ISL_1061293	2/8/2021	Alaska State Virology Laboratory	Alaska State Virology Laboratory	Stephanie DeRonde, Lisa Smith, Ph.D., Jack Chen, Ph.D.
hCoV-19/USA/AK-PHL6907/2021	EPI_ISL_1061294	2/8/2021	Alaska State Virology Laboratory	Alaska State Virology Laboratory	Stephanie DeRonde, Lisa Smith, Ph.D., Jack Chen, Ph.D.
hCoV-19/USA/AK-PHL6908/2021	EPI_ISL_1061295	2/9/2021	Alaska State Virology Laboratory	Alaska State Virology Laboratory	Stephanie DeRonde, Lisa Smith, Ph.D., Jack Chen, Ph.D.
hCoV-19/USA/AK-PHL6909/2021	EPI_ISL_1061296	2/9/2021	Alaska State Virology Laboratory	Alaska State Virology Laboratory	Stephanie DeRonde, Lisa Smith, Ph.D., Jack Chen, Ph.D.
hCoV-19/USA/AK-PHL6910/2021	EPI_ISL_1061297	2/8/2021	Alaska State Virology Laboratory	Alaska State Virology Laboratory	Stephanie DeRonde, Lisa Smith, Ph.D., Jack Chen, Ph.D.
hCoV-19/USA/AK-PHL6911/2021	EPI_ISL_1061298	2/8/2021	Alaska State Virology Laboratory	Alaska State Virology Laboratory	Stephanie DeRonde, Lisa Smith, Ph.D., Jack Chen, Ph.D.
hCoV-19/USA/AK-PHL6912/2021	EPI_ISL_1061299	2/10/2021	Alaska State Virology Laboratory	Alaska State Virology Laboratory	Stephanie DeRonde, Lisa Smith, Ph.D., Jack Chen, Ph.D.
hCoV-19/USA/AK-PHL6913/2021	EPI_ISL_1061300	2/8/2021	Alaska State Virology Laboratory	Alaska State Virology Laboratory	Stephanie DeRonde, Lisa Smith, Ph.D., Jack Chen, Ph.D.
hCoV-19/USA/AK-PHL6914/2021	EPI_ISL_1061301	2/8/2021	Alaska State Virology Laboratory	Alaska State Virology Laboratory	Stephanie DeRonde, Lisa Smith, Ph.D., Jack Chen, Ph.D.
hCoV-19/USA/AK-PHL6915/2020	EPI_ISL_1061302	12/16/2020	Alaska State Virology Laboratory	Alaska State Virology Laboratory	Stephanie DeRonde, Lisa Smith, Ph.D., Jack Chen, Ph.D.
hCoV-19/USA/AK-PHL6916/2020	EPI_ISL_1061303	12/23/2020	Alaska State Virology Laboratory	Alaska State Virology Laboratory	Stephanie DeRonde, Lisa Smith, Ph.D., Jack Chen, Ph.D.
hCoV-19/USA/AK-PHL6917/2020	EPI_ISL_1061304	12/23/2020	Alaska State Virology Laboratory	Alaska State Virology Laboratory	Stephanie DeRonde, Lisa Smith, Ph.D., Jack Chen, Ph.D.
hCoV-19/USA/AK-PHL6922/2021	EPI_ISL_1061305	2/3/2021	Alaska State Virology Laboratory	Alaska State Virology Laboratory	Stephanie DeRonde, Lisa Smith, Ph.D., Jack Chen, Ph.D.
hCoV-19/USA/AK-PHL6925/2021	EPI_ISL_1061306	2/4/2021	Alaska State Virology Laboratory	Alaska State Virology Laboratory	Stephanie DeRonde, Lisa Smith, Ph.D., Jack Chen, Ph.D.
hCoV-19/USA/AK-PHL6926/2021	EPI_ISL_1061307	2/8/2021	Alaska State Virology Laboratory	Alaska State Virology Laboratory	Stephanie DeRonde, Lisa Smith, Ph.D., Jack Chen, Ph.D.
hCoV-19/USA/AK-PHL6928/2021	EPI_ISL_1061308	1/28/2021	Alaska State Virology Laboratory	Alaska State Virology Laboratory	Stephanie DeRonde, Lisa Smith, Ph.D., Jack Chen, Ph.D.
hCoV-19/USA/AK-PHL6847/2021	EPI_ISL_1068293	1/30/2021	Alaska State Virology Laboratory	Alaska State Virology Laboratory	Stephanie DeRonde, Lisa Smith, Ph.D., Jack Chen, Ph.D.
hCoV-19/USA/AK-PHL6848/2021	EPI_ISL_1068294	1/30/2021	Alaska State Virology Laboratory	Alaska State Virology Laboratory	Stephanie DeRonde, Lisa Smith, Ph.D., Jack Chen, Ph.D.
hCoV-19/USA/AK-PHL6849/2021	EPI_ISL_1068295	1/31/2021	Alaska State Virology Laboratory	Alaska State Virology Laboratory	Stephanie DeRonde, Lisa Smith, Ph.D., Jack Chen, Ph.D.
hCoV-19/USA/AK-PHL6850/2021	EPI_ISL_1068296	2/1/2021	Alaska State Virology Laboratory	Alaska State Virology Laboratory	Stephanie DeRonde, Lisa Smith, Ph.D., Jack Chen, Ph.D.
hCoV-19/USA/AK-PHL6851/2021	EPI_ISL_1068297	1/29/2021	Alaska State Virology Laboratory	Alaska State Virology Laboratory	Stephanie DeRonde, Lisa Smith, Ph.D., Jack Chen, Ph.D.
hCoV-19/USA/AK-PHL6852/2021	EPI_ISL_1068298	2/2/2021	Alaska State Virology Laboratory	Alaska State Virology Laboratory	Stephanie DeRonde, Lisa Smith, Ph.D., Jack Chen, Ph.D.
hCoV-19/USA/AK-PHL6855/2021	EPI_ISL_1068299	2/4/2021	Alaska State Virology Laboratory	Alaska State Virology Laboratory	Stephanie DeRonde, Lisa Smith, Ph.D., Jack Chen, Ph.D.
hCoV-19/USA/AK-PHL6856/2021	EPI_ISL_1068300	2/4/2021	Alaska State Virology Laboratory	Alaska State Virology Laboratory	Stephanie DeRonde, Lisa Smith, Ph.D., Jack Chen, Ph.D.
hCoV-19/USA/AK-PHL6857/2021	EPI_ISL_1068301	2/5/2021	Alaska State Virology Laboratory	Alaska State Virology Laboratory	Stephanie DeRonde, Lisa Smith, Ph.D., Jack Chen, Ph.D.
hCoV-19/USA/AK-PHL6870/2021	EPI_ISL_1068302	1/11/2021	Alaska State Virology Laboratory	Alaska State Virology Laboratory	Stephanie DeRonde, Lisa Smith, Ph.D., Jack Chen, Ph.D.
hCoV-19/USA/AK-PHL6871/2021	EPI_ISL_1068303	1/15/2021	Alaska State Virology Laboratory	Alaska State Virology Laboratory	Stephanie DeRonde, Lisa Smith, Ph.D., Jack Chen, Ph.D.
hCoV-19/USA/AK-PHL6872/2021	EPI_ISL_1068304	1/12/2021	Alaska State Virology Laboratory	Alaska State Virology Laboratory	Stephanie DeRonde, Lisa Smith, Ph.D., Jack Chen, Ph.D.
hCoV-19/USA/AK-PHL6876/2021	EPI_ISL_1068305	2/5/2021	Alaska State Virology Laboratory	Alaska State Virology Laboratory	Stephanie DeRonde, Lisa Smith, Ph.D., Jack Chen, Ph.D.
hCoV-19/USA/AK-PHL6877/2021	EPI_ISL_1068306	1/28/2021	Alaska State Virology Laboratory	Alaska State Virology Laboratory	Stephanie DeRonde, Lisa Smith, Ph.D., Jack Chen, Ph.D.
hCoV-19/USA/AK-PHL6878/2021	EPI_ISL_1068307	1/28/2021	Alaska State Virology Laboratory	Alaska State Virology Laboratory	Stephanie DeRonde, Lisa Smith, Ph.D., Jack Chen, Ph.D.
hCoV-19/USA/AK-PHL6879/2021	EPI_ISL_1068308	1/27/2021	Alaska State Virology Laboratory	Alaska State Virology Laboratory	Stephanie DeRonde, Lisa Smith, Ph.D., Jack Chen, Ph.D.
hCoV-19/USA/AK-PHL6880/2021	EPI_ISL_1068309	1/28/2021	Alaska State Virology Laboratory	Alaska State Virology Laboratory	Stephanie DeRonde, Lisa Smith, Ph.D., Jack Chen, Ph.D.
hCoV-19/USA/AK-PHL6881/2021	EPI_ISL_1068310	1/28/2021	Alaska State Virology Laboratory	Alaska State Virology Laboratory	Stephanie DeRonde, Lisa Smith, Ph.D., Jack Chen, Ph.D.
hCoV-19/USA/AK-PHL6882/2021	EPI_ISL_1068311	2/6/2021	Alaska State Virology Laboratory	Alaska State Virology Laboratory	Stephanie DeRonde, Lisa Smith, Ph.D., Jack Chen, Ph.D.
hCoV-19/USA/AK-PHL6883/2021	EPI_ISL_1068312	2/6/2021	Alaska State Virology Laboratory	Alaska State Virology Laboratory	Stephanie DeRonde, Lisa Smith, Ph.D., Jack Chen, Ph.D.
hCoV-19/USA/AK-PHL6884/2021	EPI_ISL_1068313	2/6/2021	Alaska State Virology Laboratory	Alaska State Virology Laboratory	Stephanie DeRonde, Lisa Smith, Ph.D., Jack Chen, Ph.D.
hCoV-19/USA/AK-PHL6885/2021	EPI_ISL_1068314	2/7/2021	Alaska State Virology Laboratory	Alaska State Virology Laboratory	Stephanie DeRonde, Lisa Smith, Ph.D., Jack Chen, Ph.D.
hCoV-19/USA/AK-CDC-ASC210042064/2021	EPI_ISL_1737216	4/1/2021	Aegis Sciences Corporation	Centers for Disease Control and Prevention Division of Viral Diseases, Pathogen Discovery	Dakota Howard, Dhwani Batra, Peter W. Cook, Kara Moser, Adrian Paskey, Jason Caravas, Benjamin Rambo-Martin, Shatavia Morrison, Christopher Gulvick, Scott Sammons, Yvette Unoarumhi, Darlene Wagner, Matthew Schmerer, Cyndi Clark, Patrick Campbell, Rob Case, Vikramsinha Ghorpade, Holly Houdeshell, Ola Kvalvaag, Dillon Nall, Ethan Sanders, Alec Vest, Shaun Westlund, Matthew Hardison, Clinton R. Paden, Duncan MacCannell
hCoV-19/USA/AK-PHL7231/2021	EPI_ISL_1182693	2/16/2021	Alaska State Virology Laboratory	Alaska State Virology Laboratory	Stephanie DeRonde, Lisa Smith, Ph.D., Jack Chen, Ph.D.
hCoV-19/USA/AK-PHL7267/2021	EPI_ISL_1182694	2/19/2021	Alaska State Virology Laboratory	Alaska State Virology Laboratory	Stephanie DeRonde, Lisa Smith, Ph.D., Jack Chen, Ph.D.
hCoV-19/USA/AK-PHL7253/2021	EPI_ISL_1182695	2/17/2021	Alaska State Virology Laboratory	Alaska State Virology Laboratory	Stephanie DeRonde, Lisa Smith, Ph.D., Jack Chen, Ph.D.
hCoV-19/USA/AK-PHL7239/2021	EPI_ISL_1182696	2/16/2021	Alaska State Virology Laboratory	Alaska State Virology Laboratory	Stephanie DeRonde, Lisa Smith, Ph.D., Jack Chen, Ph.D.
hCoV-19/USA/AK-PHL7246/2021	EPI_ISL_1182697	2/17/2021	Alaska State Virology Laboratory	Alaska State Virology Laboratory	Stephanie DeRonde, Lisa Smith, Ph.D., Jack Chen, Ph.D.
hCoV-19/USA/AK-PHL7247/2021	EPI_ISL_1182698	2/17/2021	Alaska State Virology Laboratory	Alaska State Virology Laboratory	Stephanie DeRonde, Lisa Smith, Ph.D., Jack Chen, Ph.D.
hCoV-19/USA/AK-PHL7234/2021	EPI_ISL_1182699	2/16/2021	Alaska State Virology Laboratory	Alaska State Virology Laboratory	Stephanie DeRonde, Lisa Smith, Ph.D., Jack Chen, Ph.D.

hCoV-19/USA/AK-PHL7236/2021	EPI_ISL_1182700	2/16/2021	Alaska State Virology Laboratory	Alaska State Virology Laboratory	Stephanie DeRonde, Lisa Smith, Ph.D., Jack Chen, Ph.D.
hCoV-19/USA/AK-PHL7263/2021	EPI_ISL_1182701	2/19/2021	Alaska State Virology Laboratory	Alaska State Virology Laboratory	Stephanie DeRonde, Lisa Smith, Ph.D., Jack Chen, Ph.D.
hCoV-19/USA/AK-PHL7248/2021	EPI_ISL_1182702	2/17/2021	Alaska State Virology Laboratory	Alaska State Virology Laboratory	Stephanie DeRonde, Lisa Smith, Ph.D., Jack Chen, Ph.D.
hCoV-19/USA/AK-PHL7249/2021	EPI_ISL_1182703	2/17/2021	Alaska State Virology Laboratory	Alaska State Virology Laboratory	Stephanie DeRonde, Lisa Smith, Ph.D., Jack Chen, Ph.D.
hCoV-19/USA/AK-PHL7266/2021	EPI_ISL_1182704	2/19/2021	Alaska State Virology Laboratory	Alaska State Virology Laboratory	Stephanie DeRonde, Lisa Smith, Ph.D., Jack Chen, Ph.D.
hCoV-19/USA/AK-PHL7255/2021	EPI_ISL_1182705	2/18/2021	Alaska State Virology Laboratory	Alaska State Virology Laboratory	Stephanie DeRonde, Lisa Smith, Ph.D., Jack Chen, Ph.D.
hCoV-19/USA/AK-PHL7262/2021	EPI_ISL_1182707	2/18/2021	Alaska State Virology Laboratory	Alaska State Virology Laboratory	Stephanie DeRonde, Lisa Smith, Ph.D., Jack Chen, Ph.D.
hCoV-19/USA/AK-PHL7240/2021	EPI_ISL_1182708	2/17/2021	Alaska State Virology Laboratory	Alaska State Virology Laboratory	Stephanie DeRonde, Lisa Smith, Ph.D., Jack Chen, Ph.D.
hCoV-19/USA/AK-PHL7244/2021	EPI_ISL_1182709	2/17/2021	Alaska State Virology Laboratory	Alaska State Virology Laboratory	Stephanie DeRonde, Lisa Smith, Ph.D., Jack Chen, Ph.D.
hCoV-19/USA/AK-PHL7245/2021	EPI_ISL_1182710	2/17/2021	Alaska State Virology Laboratory	Alaska State Virology Laboratory	Stephanie DeRonde, Lisa Smith, Ph.D., Jack Chen, Ph.D.
hCoV-19/USA/AK-PHL7258/2021	EPI_ISL_1182713	2/17/2021	Alaska State Virology Laboratory	Alaska State Virology Laboratory	Stephanie DeRonde, Lisa Smith, Ph.D., Jack Chen, Ph.D.
hCoV-19/USA/AK-PHL7260/2021	EPI_ISL_1182714	2/17/2021	Alaska State Virology Laboratory	Alaska State Virology Laboratory	Stephanie DeRonde, Lisa Smith, Ph.D., Jack Chen, Ph.D.
hCoV-19/USA/AK-PHL7222/2021	EPI_ISL_1182715	2/16/2021	Alaska State Virology Laboratory	Alaska State Virology Laboratory	Stephanie DeRonde, Lisa Smith, Ph.D., Jack Chen, Ph.D.
hCoV-19/USA/AK-PHL7223/2021	EPI_ISL_1182716	2/16/2021	Alaska State Virology Laboratory	Alaska State Virology Laboratory	Stephanie DeRonde, Lisa Smith, Ph.D., Jack Chen, Ph.D.
hCoV-19/USA/AK-PHL7224/2021	EPI_ISL_1182717	2/16/2021	Alaska State Virology Laboratory	Alaska State Virology Laboratory	Stephanie DeRonde, Lisa Smith, Ph.D., Jack Chen, Ph.D.
hCoV-19/USA/AK-PHL7225/2021	EPI_ISL_1182718	2/16/2021	Alaska State Virology Laboratory	Alaska State Virology Laboratory	Stephanie DeRonde, Lisa Smith, Ph.D., Jack Chen, Ph.D.
hCoV-19/USA/AK-PHL7226/2021	EPI_ISL_1182719	2/16/2021	Alaska State Virology Laboratory	Alaska State Virology Laboratory	Stephanie DeRonde, Lisa Smith, Ph.D., Jack Chen, Ph.D.
hCoV-19/USA/AK-PHL7227/2021	EPI_ISL_1182720	2/16/2021	Alaska State Virology Laboratory	Alaska State Virology Laboratory	Stephanie DeRonde, Lisa Smith, Ph.D., Jack Chen, Ph.D.
hCoV-19/USA/AK-PHL7228/2021	EPI_ISL_1182721	2/16/2021	Alaska State Virology Laboratory	Alaska State Virology Laboratory	Stephanie DeRonde, Lisa Smith, Ph.D., Jack Chen, Ph.D.
hCoV-19/USA/AK-PHL7229/2021	EPI_ISL_1182722	2/16/2021	Alaska State Virology Laboratory	Alaska State Virology Laboratory	Stephanie DeRonde, Lisa Smith, Ph.D., Jack Chen, Ph.D.
hCoV-19/USA/AK-PHL7230/2021	EPI_ISL_1182723	2/16/2021	Alaska State Virology Laboratory	Alaska State Virology Laboratory	Stephanie DeRonde, Lisa Smith, Ph.D., Jack Chen, Ph.D.
hCoV-19/USA/AK-PHL7232/2021	EPI_ISL_1182724	2/16/2021	Alaska State Virology Laboratory	Alaska State Virology Laboratory	Stephanie DeRonde, Lisa Smith, Ph.D., Jack Chen, Ph.D.
hCoV-19/USA/AK-PHL7233/2021	EPI_ISL_1182725	2/16/2021	Alaska State Virology Laboratory	Alaska State Virology Laboratory	Stephanie DeRonde, Lisa Smith, Ph.D., Jack Chen, Ph.D.
hCoV-19/USA/AK-PHL7235/2021	EPI_ISL_1182726	2/16/2021	Alaska State Virology Laboratory	Alaska State Virology Laboratory	Stephanie DeRonde, Lisa Smith, Ph.D., Jack Chen, Ph.D.
hCoV-19/USA/AK-PHL7237/2021	EPI_ISL_1182727	2/16/2021	Alaska State Virology Laboratory	Alaska State Virology Laboratory	Stephanie DeRonde, Lisa Smith, Ph.D., Jack Chen, Ph.D.
hCoV-19/USA/AK-PHL7238/2021	EPI_ISL_1182728	2/16/2021	Alaska State Virology Laboratory	Alaska State Virology Laboratory	Stephanie DeRonde, Lisa Smith, Ph.D., Jack Chen, Ph.D.
hCoV-19/USA/AK-PHL7241/2021	EPI_ISL_1182729	2/16/2021	Alaska State Virology Laboratory	Alaska State Virology Laboratory	Stephanie DeRonde, Lisa Smith, Ph.D., Jack Chen, Ph.D.
hCoV-19/USA/AK-PHL7251/2021	EPI_ISL_1182730	2/16/2021	Alaska State Virology Laboratory	Alaska State Virology Laboratory	Stephanie DeRonde, Lisa Smith, Ph.D., Jack Chen, Ph.D.
hCoV-19/USA/AK-PHL7264/2021	EPI_ISL_1182732	1/29/2021	Alaska State Virology Laboratory	Alaska State Virology Laboratory	Stephanie DeRonde, Lisa Smith, Ph.D., Jack Chen, Ph.D.
hCoV-19/USA/AK-PHL7295/2021	EPI_ISL_1182733	2/21/2021	Alaska State Virology Laboratory	Alaska State Virology Laboratory	Stephanie DeRonde, Lisa Smith, Ph.D., Jack Chen, Ph.D.
hCoV-19/USA/AK-PHL7306/2021	EPI_ISL_1182734	2/8/2021	Alaska State Virology Laboratory	Alaska State Virology Laboratory	Stephanie DeRonde, Lisa Smith, Ph.D., Jack Chen, Ph.D.
hCoV-19/USA/AK-PHL7313/2021	EPI_ISL_1182735	2/17/2021	Alaska State Virology Laboratory	Alaska State Virology Laboratory	Stephanie DeRonde, Lisa Smith, Ph.D., Jack Chen, Ph.D.
hCoV-19/USA/AK-PHL7283/2021	EPI_ISL_1182736	2/16/2021	Alaska State Virology Laboratory	Alaska State Virology Laboratory	Stephanie DeRonde, Lisa Smith, Ph.D., Jack Chen, Ph.D.
hCoV-19/USA/AK-PHL7307/2021	EPI_ISL_1182737	2/13/2021	Alaska State Virology Laboratory	Alaska State Virology Laboratory	Stephanie DeRonde, Lisa Smith, Ph.D., Jack Chen, Ph.D.
hCoV-19/USA/AK-PHL7274/2021	EPI_ISL_1182738	2/23/2021	Alaska State Virology Laboratory	Alaska State Virology Laboratory	Stephanie DeRonde, Lisa Smith, Ph.D., Jack Chen, Ph.D.
hCoV-19/USA/AK-PHL7276/2021	EPI_ISL_1182739	2/23/2021	Alaska State Virology Laboratory	Alaska State Virology Laboratory	Stephanie DeRonde, Lisa Smith, Ph.D., Jack Chen, Ph.D.
hCoV-19/USA/AK-PHL7277/2021	EPI_ISL_1182740	2/23/2021	Alaska State Virology Laboratory	Alaska State Virology Laboratory	Stephanie DeRonde, Lisa Smith, Ph.D., Jack Chen, Ph.D.
hCoV-19/USA/AK-PHL7271/2021	EPI_ISL_1182741	2/22/2021	Alaska State Virology Laboratory	Alaska State Virology Laboratory	Stephanie DeRonde, Lisa Smith, Ph.D., Jack Chen, Ph.D.
hCoV-19/USA/AK-PHL7273/2021	EPI_ISL_1182742	2/22/2021	Alaska State Virology Laboratory	Alaska State Virology Laboratory	Stephanie DeRonde, Lisa Smith, Ph.D., Jack Chen, Ph.D.
hCoV-19/USA/AK-PHL7275/2021	EPI_ISL_1182743	2/22/2021	Alaska State Virology Laboratory	Alaska State Virology Laboratory	Stephanie DeRonde, Lisa Smith, Ph.D., Jack Chen, Ph.D.
hCoV-19/USA/AK-PHL7285/2021	EPI_ISL_1182744	2/22/2021	Alaska State Virology Laboratory	Alaska State Virology Laboratory	Stephanie DeRonde, Lisa Smith, Ph.D., Jack Chen, Ph.D.
hCoV-19/USA/AK-PHL7288/2021	EPI_ISL_1182745	2/22/2021	Alaska State Virology Laboratory	Alaska State Virology Laboratory	Stephanie DeRonde, Lisa Smith, Ph.D., Jack Chen, Ph.D.
hCoV-19/USA/AK-PHL7292/2021	EPI_ISL_1182746	2/22/2021	Alaska State Virology Laboratory	Alaska State Virology Laboratory	Stephanie DeRonde, Lisa Smith, Ph.D., Jack Chen, Ph.D.
hCoV-19/USA/AK-PHL7299/2021	EPI_ISL_1182747	2/22/2021	Alaska State Virology Laboratory	Alaska State Virology Laboratory	Stephanie DeRonde, Lisa Smith, Ph.D., Jack Chen, Ph.D.
hCoV-19/USA/AK-PHL7300/2021	EPI_ISL_1182748	2/22/2021	Alaska State Virology Laboratory	Alaska State Virology Laboratory	Stephanie DeRonde, Lisa Smith, Ph.D., Jack Chen, Ph.D.
hCoV-19/USA/AK-PHL7301/2021	EPI_ISL_1182749	2/22/2021	Alaska State Virology Laboratory	Alaska State Virology Laboratory	Stephanie DeRonde, Lisa Smith, Ph.D., Jack Chen, Ph.D.
hCoV-19/USA/AK-PHL7281/2021	EPI_ISL_1182750	2/21/2021	Alaska State Virology Laboratory	Alaska State Virology Laboratory	Stephanie DeRonde, Lisa Smith, Ph.D., Jack Chen, Ph.D.
hCoV-19/USA/AK-PHL7293/2021	EPI_ISL_1182752	2/21/2021	Alaska State Virology Laboratory	Alaska State Virology Laboratory	Stephanie DeRonde, Lisa Smith, Ph.D., Jack Chen, Ph.D.
hCoV-19/USA/AK-PHL7294/2021	EPI_ISL_1182753	2/21/2021	Alaska State Virology Laboratory	Alaska State Virology Laboratory	Stephanie DeRonde, Lisa Smith, Ph.D., Jack Chen, Ph.D.
hCoV-19/USA/AK-PHL7297/2021	EPI_ISL_1182755	2/21/2021	Alaska State Virology Laboratory	Alaska State Virology Laboratory	Stephanie DeRonde, Lisa Smith, Ph.D., Jack Chen, Ph.D.
hCoV-19/USA/AK-PHL7270/2021	EPI_ISL_1182756	2/20/2021	Alaska State Virology Laboratory	Alaska State Virology Laboratory	Stephanie DeRonde, Lisa Smith, Ph.D., Jack Chen, Ph.D.

hCoV-19/USA/AK-PHL7290/2021	EPI_ISL_1182757	2/20/2021	Alaska State Virology Laboratory	Alaska State Virology Laboratory	Stephanie DeRonde, Lisa Smith, Ph.D., Jack Chen, Ph.D.
hCoV-19/USA/AK-PHL7291/2021	EPI_ISL_1182758	2/20/2021	Alaska State Virology Laboratory	Alaska State Virology Laboratory	Stephanie DeRonde, Lisa Smith, Ph.D., Jack Chen, Ph.D.
hCoV-19/USA/AK-PHL7311/2021	EPI_ISL_1182759	2/17/2021	Alaska State Virology Laboratory	Alaska State Virology Laboratory	Stephanie DeRonde, Lisa Smith, Ph.D., Jack Chen, Ph.D.
hCoV-19/USA/AK-PHL7284/2021	EPI_ISL_1182760	2/16/2021	Alaska State Virology Laboratory	Alaska State Virology Laboratory	Stephanie DeRonde, Lisa Smith, Ph.D., Jack Chen, Ph.D.
hCoV-19/USA/AK-PHL7304/2021	EPI_ISL_1182761	2/15/2021	Alaska State Virology Laboratory	Alaska State Virology Laboratory	Stephanie DeRonde, Lisa Smith, Ph.D., Jack Chen, Ph.D.
hCoV-19/USA/AK-PHL7305/2021	EPI_ISL_1182762	2/15/2021	Alaska State Virology Laboratory	Alaska State Virology Laboratory	Stephanie DeRonde, Lisa Smith, Ph.D., Jack Chen, Ph.D.
hCoV-19/USA/AK-PHL7309/2021	EPI_ISL_1182763	2/15/2021	Alaska State Virology Laboratory	Alaska State Virology Laboratory	Stephanie DeRonde, Lisa Smith, Ph.D., Jack Chen, Ph.D.
hCoV-19/USA/AK-PHL7310/2021	EPI_ISL_1182764	2/14/2021	Alaska State Virology Laboratory	Alaska State Virology Laboratory	Stephanie DeRonde, Lisa Smith, Ph.D., Jack Chen, Ph.D.
hCoV-19/USA/AK-PHL7308/2021	EPI_ISL_1182765	2/13/2021	Alaska State Virology Laboratory	Alaska State Virology Laboratory	Stephanie DeRonde, Lisa Smith, Ph.D., Jack Chen, Ph.D.
hCoV-19/USA/AK-PHL7280/2021	EPI_ISL_1182766	2/5/2021	Alaska State Virology Laboratory	Alaska State Virology Laboratory	Stephanie DeRonde, Lisa Smith, Ph.D., Jack Chen, Ph.D.
hCoV-19/USA/AK-PHL7134/2021	EPI_ISL_1182767	2/15/2021	Alaska State Virology Laboratory	Alaska State Virology Laboratory	Stephanie DeRonde, Lisa Smith, Ph.D., Jack Chen, Ph.D.
hCoV-19/USA/AK-PHL7135/2021	EPI_ISL_1182768	2/15/2021	Alaska State Virology Laboratory	Alaska State Virology Laboratory	Stephanie DeRonde, Lisa Smith, Ph.D., Jack Chen, Ph.D.
hCoV-19/USA/AK-PHL7166/2021	EPI_ISL_1182769	2/15/2021	Alaska State Virology Laboratory	Alaska State Virology Laboratory	Stephanie DeRonde, Lisa Smith, Ph.D., Jack Chen, Ph.D.
hCoV-19/USA/AK-PHL7145/2021	EPI_ISL_1182770	2/15/2021	Alaska State Virology Laboratory	Alaska State Virology Laboratory	Stephanie DeRonde, Lisa Smith, Ph.D., Jack Chen, Ph.D.
hCoV-19/USA/AK-PHL7152/2021	EPI_ISL_1182771	2/15/2021	Alaska State Virology Laboratory	Alaska State Virology Laboratory	Stephanie DeRonde, Lisa Smith, Ph.D., Jack Chen, Ph.D.
hCoV-19/USA/AK-PHL7173/2021	EPI_ISL_1182772	2/15/2021	Alaska State Virology Laboratory	Alaska State Virology Laboratory	Stephanie DeRonde, Lisa Smith, Ph.D., Jack Chen, Ph.D.
hCoV-19/USA/AK-PHL7130/2021	EPI_ISL_1182773	2/15/2021	Alaska State Virology Laboratory	Alaska State Virology Laboratory	Stephanie DeRonde, Lisa Smith, Ph.D., Jack Chen, Ph.D.
hCoV-19/USA/AK-PHL7131/2021	EPI_ISL_1182774	2/15/2021	Alaska State Virology Laboratory	Alaska State Virology Laboratory	Stephanie DeRonde, Lisa Smith, Ph.D., Jack Chen, Ph.D.
hCoV-19/USA/AK-PHL7132/2021	EPI_ISL_1182775	2/15/2021	Alaska State Virology Laboratory	Alaska State Virology Laboratory	Stephanie DeRonde, Lisa Smith, Ph.D., Jack Chen, Ph.D.
hCoV-19/USA/AK-PHL7133/2021	EPI_ISL_1182776	2/15/2021	Alaska State Virology Laboratory	Alaska State Virology Laboratory	Stephanie DeRonde, Lisa Smith, Ph.D., Jack Chen, Ph.D.
hCoV-19/USA/AK-PHL7136/2021	EPI_ISL_1182777	2/15/2021	Alaska State Virology Laboratory	Alaska State Virology Laboratory	Stephanie DeRonde, Lisa Smith, Ph.D., Jack Chen, Ph.D.
hCoV-19/USA/AK-PHL7137/2021	EPI_ISL_1182778	2/15/2021	Alaska State Virology Laboratory	Alaska State Virology Laboratory	Stephanie DeRonde, Lisa Smith, Ph.D., Jack Chen, Ph.D.
hCoV-19/USA/AK-PHL7138/2021	EPI_ISL_1182779	2/15/2021	Alaska State Virology Laboratory	Alaska State Virology Laboratory	Stephanie DeRonde, Lisa Smith, Ph.D., Jack Chen, Ph.D.
hCoV-19/USA/AK-PHL7139/2021	EPI_ISL_1182780	2/15/2021	Alaska State Virology Laboratory	Alaska State Virology Laboratory	Stephanie DeRonde, Lisa Smith, Ph.D., Jack Chen, Ph.D.
hCoV-19/USA/AK-PHL7140/2021	EPI_ISL_1182781	2/15/2021	Alaska State Virology Laboratory	Alaska State Virology Laboratory	Stephanie DeRonde, Lisa Smith, Ph.D., Jack Chen, Ph.D.
hCoV-19/USA/AK-PHL7141/2021	EPI_ISL_1182782	2/15/2021	Alaska State Virology Laboratory	Alaska State Virology Laboratory	Stephanie DeRonde, Lisa Smith, Ph.D., Jack Chen, Ph.D.
hCoV-19/USA/AK-PHL7142/2021	EPI_ISL_1182783	2/15/2021	Alaska State Virology Laboratory	Alaska State Virology Laboratory	Stephanie DeRonde, Lisa Smith, Ph.D., Jack Chen, Ph.D.
hCoV-19/USA/AK-PHL7143/2021	EPI_ISL_1182784	2/15/2021	Alaska State Virology Laboratory	Alaska State Virology Laboratory	Stephanie DeRonde, Lisa Smith, Ph.D., Jack Chen, Ph.D.
hCoV-19/USA/AK-PHL7144/2021	EPI_ISL_1182785	2/15/2021	Alaska State Virology Laboratory	Alaska State Virology Laboratory	Stephanie DeRonde, Lisa Smith, Ph.D., Jack Chen, Ph.D.
hCoV-19/USA/AK-PHL7147/2021	EPI_ISL_1182787	2/15/2021	Alaska State Virology Laboratory	Alaska State Virology Laboratory	Stephanie DeRonde, Lisa Smith, Ph.D., Jack Chen, Ph.D.
hCoV-19/USA/AK-PHL7149/2021	EPI_ISL_1182789	2/15/2021	Alaska State Virology Laboratory	Alaska State Virology Laboratory	Stephanie DeRonde, Lisa Smith, Ph.D., Jack Chen, Ph.D.
hCoV-19/USA/AK-PHL7150/2021	EPI_ISL_1182790	2/15/2021	Alaska State Virology Laboratory	Alaska State Virology Laboratory	Stephanie DeRonde, Lisa Smith, Ph.D., Jack Chen, Ph.D.
hCoV-19/USA/AK-PHL7151/2021	EPI_ISL_1182791	2/15/2021	Alaska State Virology Laboratory	Alaska State Virology Laboratory	Stephanie DeRonde, Lisa Smith, Ph.D., Jack Chen, Ph.D.
hCoV-19/USA/AK-PHL7153/2021	EPI_ISL_1182792	2/15/2021	Alaska State Virology Laboratory	Alaska State Virology Laboratory	Stephanie DeRonde, Lisa Smith, Ph.D., Jack Chen, Ph.D.
hCoV-19/USA/AK-PHL7154/2021	EPI_ISL_1182793	2/15/2021	Alaska State Virology Laboratory	Alaska State Virology Laboratory	Stephanie DeRonde, Lisa Smith, Ph.D., Jack Chen, Ph.D.
hCoV-19/USA/AK-PHL7155/2021	EPI_ISL_1182794	2/15/2021	Alaska State Virology Laboratory	Alaska State Virology Laboratory	Stephanie DeRonde, Lisa Smith, Ph.D., Jack Chen, Ph.D.
hCoV-19/USA/AK-PHL7156/2021	EPI_ISL_1182795	2/15/2021	Alaska State Virology Laboratory	Alaska State Virology Laboratory	Stephanie DeRonde, Lisa Smith, Ph.D., Jack Chen, Ph.D.
hCoV-19/USA/AK-PHL7158/2021	EPI_ISL_1182796	2/15/2021	Alaska State Virology Laboratory	Alaska State Virology Laboratory	Stephanie DeRonde, Lisa Smith, Ph.D., Jack Chen, Ph.D.
hCoV-19/USA/AK-PHL7159/2021	EPI_ISL_1182797	2/15/2021	Alaska State Virology Laboratory	Alaska State Virology Laboratory	Stephanie DeRonde, Lisa Smith, Ph.D., Jack Chen, Ph.D.
hCoV-19/USA/AK-PHL7160/2021	EPI_ISL_1182798	2/15/2021	Alaska State Virology Laboratory	Alaska State Virology Laboratory	Stephanie DeRonde, Lisa Smith, Ph.D., Jack Chen, Ph.D.
hCoV-19/USA/AK-PHL7161/2021	EPI_ISL_1182799	2/15/2021	Alaska State Virology Laboratory	Alaska State Virology Laboratory	Stephanie DeRonde, Lisa Smith, Ph.D., Jack Chen, Ph.D.
hCoV-19/USA/AK-PHL7162/2021	EPI_ISL_1182800	2/15/2021	Alaska State Virology Laboratory	Alaska State Virology Laboratory	Stephanie DeRonde, Lisa Smith, Ph.D., Jack Chen, Ph.D.
hCoV-19/USA/AK-PHL7163/2021	EPI_ISL_1182801	2/15/2021	Alaska State Virology Laboratory	Alaska State Virology Laboratory	Stephanie DeRonde, Lisa Smith, Ph.D., Jack Chen, Ph.D.
hCoV-19/USA/AK-PHL7164/2021	EPI_ISL_1182802	2/15/2021	Alaska State Virology Laboratory	Alaska State Virology Laboratory	Stephanie DeRonde, Lisa Smith, Ph.D., Jack Chen, Ph.D.
hCoV-19/USA/AK-PHL7165/2021	EPI_ISL_1182803	2/15/2021	Alaska State Virology Laboratory	Alaska State Virology Laboratory	Stephanie DeRonde, Lisa Smith, Ph.D., Jack Chen, Ph.D.
hCoV-19/USA/AK-PHL7167/2021	EPI_ISL_1182804	2/15/2021	Alaska State Virology Laboratory	Alaska State Virology Laboratory	Stephanie DeRonde, Lisa Smith, Ph.D., Jack Chen, Ph.D.
hCoV-19/USA/AK-PHL7168/2021	EPI_ISL_1182805	2/15/2021	Alaska State Virology Laboratory	Alaska State Virology Laboratory	Stephanie DeRonde, Lisa Smith, Ph.D., Jack Chen, Ph.D.
hCoV-19/USA/AK-PHL7170/2021	EPI_ISL_1182807	2/15/2021	Alaska State Virology Laboratory	Alaska State Virology Laboratory	Stephanie DeRonde, Lisa Smith, Ph.D., Jack Chen, Ph.D.
hCoV-19/USA/AK-PHL7171/2021	EPI_ISL_1182808	2/15/2021	Alaska State Virology Laboratory	Alaska State Virology Laboratory	Stephanie DeRonde, Lisa Smith, Ph.D., Jack Chen, Ph.D.
hCoV-19/USA/AK-PHL7172/2021	EPI_ISL_1182809	2/15/2021	Alaska State Virology Laboratory	Alaska State Virology Laboratory	Stephanie DeRonde, Lisa Smith, Ph.D., Jack Chen, Ph.D.
hCoV-19/USA/AK-PHL7174/2021	EPI_ISL_1182810	2/15/2021	Alaska State Virology Laboratory	Alaska State Virology Laboratory	Stephanie DeRonde, Lisa Smith, Ph.D., Jack Chen, Ph.D.

hCoV-19/USA/AK-PHL7175/2021	EPI_ISL_1182811	2/15/2021	Alaska State Virology Laboratory	Alaska State Virology Laboratory	Stephanie DeRonde, Lisa Smith, Ph.D., Jack Chen, Ph.D.
hCoV-19/USA/AK-PHL7176/2021	EPI_ISL_1195910	2/13/2021	Alaska State Virology Laboratory	Alaska State Virology Laboratory	Stephanie DeRonde, Elva House, Lisa Smith, Ph.D., Jack Chen, Ph.D.
hCoV-19/USA/AK-PHL7177/2021	EPI_ISL_1195911	2/15/2021	Alaska State Virology Laboratory	Alaska State Virology Laboratory	Stephanie DeRonde, Elva House, Lisa Smith, Ph.D., Jack Chen, Ph.D.
hCoV-19/USA/AK-PHL7178/2021	EPI_ISL_1195912	2/15/2021	Alaska State Virology Laboratory	Alaska State Virology Laboratory	Stephanie DeRonde, Elva House, Lisa Smith, Ph.D., Jack Chen, Ph.D.
hCoV-19/USA/AK-PHL7181/2021	EPI_ISL_1195914	2/15/2021	Alaska State Virology Laboratory	Alaska State Virology Laboratory	Stephanie DeRonde, Elva House, Lisa Smith, Ph.D., Jack Chen, Ph.D.
hCoV-19/USA/AK-PHL7182/2021	EPI_ISL_1195915	2/15/2021	Alaska State Virology Laboratory	Alaska State Virology Laboratory	Stephanie DeRonde, Elva House, Lisa Smith, Ph.D., Jack Chen, Ph.D.
hCoV-19/USA/AK-PHL7184/2021	EPI_ISL_1195917	2/15/2021	Alaska State Virology Laboratory	Alaska State Virology Laboratory	Stephanie DeRonde, Elva House, Lisa Smith, Ph.D., Jack Chen, Ph.D.
hCoV-19/USA/AK-PHL7185/2021	EPI_ISL_1195918	2/15/2021	Alaska State Virology Laboratory	Alaska State Virology Laboratory	Stephanie DeRonde, Elva House, Lisa Smith, Ph.D., Jack Chen, Ph.D.
hCoV-19/USA/AK-PHL7186/2021	EPI_ISL_1195919	2/15/2021	Alaska State Virology Laboratory	Alaska State Virology Laboratory	Stephanie DeRonde, Elva House, Lisa Smith, Ph.D., Jack Chen, Ph.D.
hCoV-19/USA/AK-PHL7187/2021	EPI_ISL_1195920	2/15/2021	Alaska State Virology Laboratory	Alaska State Virology Laboratory	Stephanie DeRonde, Elva House, Lisa Smith, Ph.D., Jack Chen, Ph.D.
hCoV-19/USA/AK-PHL7188/2021	EPI_ISL_1195921	2/15/2021	Alaska State Virology Laboratory	Alaska State Virology Laboratory	Stephanie DeRonde, Elva House, Lisa Smith, Ph.D., Jack Chen, Ph.D.
hCoV-19/USA/AK-PHL7189/2021	EPI_ISL_1195922	2/15/2021	Alaska State Virology Laboratory	Alaska State Virology Laboratory	Stephanie DeRonde, Elva House, Lisa Smith, Ph.D., Jack Chen, Ph.D.
hCoV-19/USA/AK-PHL7190/2021	EPI_ISL_1195923	2/14/2021	Alaska State Virology Laboratory	Alaska State Virology Laboratory	Stephanie DeRonde, Elva House, Lisa Smith, Ph.D., Jack Chen, Ph.D.
hCoV-19/USA/AK-PHL7201/2021	EPI_ISL_1195926	2/16/2021	Alaska State Virology Laboratory	Alaska State Virology Laboratory	Stephanie DeRonde, Elva House, Lisa Smith, Ph.D., Jack Chen, Ph.D.
hCoV-19/USA/AK-PHL7202/2021	EPI_ISL_1195927	2/16/2021	Alaska State Virology Laboratory	Alaska State Virology Laboratory	Stephanie DeRonde, Elva House, Lisa Smith, Ph.D., Jack Chen, Ph.D.
hCoV-19/USA/AK-PHL7204/2021	EPI_ISL_1195929	2/16/2021	Alaska State Virology Laboratory	Alaska State Virology Laboratory	Stephanie DeRonde, Elva House, Lisa Smith, Ph.D., Jack Chen, Ph.D.
hCoV-19/USA/AK-PHL7206/2021	EPI_ISL_1195931	2/16/2021	Alaska State Virology Laboratory	Alaska State Virology Laboratory	Stephanie DeRonde, Elva House, Lisa Smith, Ph.D., Jack Chen, Ph.D.
hCoV-19/USA/AK-PHL7207/2021	EPI_ISL_1195932	2/16/2021	Alaska State Virology Laboratory	Alaska State Virology Laboratory	Stephanie DeRonde, Elva House, Lisa Smith, Ph.D., Jack Chen, Ph.D.
hCoV-19/USA/AK-PHL7208/2021	EPI_ISL_1195933	2/16/2021	Alaska State Virology Laboratory	Alaska State Virology Laboratory	Stephanie DeRonde, Elva House, Lisa Smith, Ph.D., Jack Chen, Ph.D.
hCoV-19/USA/AK-PHL7209/2021	EPI_ISL_1195934	2/16/2021	Alaska State Virology Laboratory	Alaska State Virology Laboratory	Stephanie DeRonde, Elva House, Lisa Smith, Ph.D., Jack Chen, Ph.D.
hCoV-19/USA/AK-PHL7210/2021	EPI_ISL_1195935	2/16/2021	Alaska State Virology Laboratory	Alaska State Virology Laboratory	Stephanie DeRonde, Elva House, Lisa Smith, Ph.D., Jack Chen, Ph.D.
hCoV-19/USA/AK-PHL7211/2021	EPI_ISL_1195936	2/15/2021	Alaska State Virology Laboratory	Alaska State Virology Laboratory	Stephanie DeRonde, Elva House, Lisa Smith, Ph.D., Jack Chen, Ph.D.
hCoV-19/USA/AK-PHL7213/2021	EPI_ISL_1195937	2/16/2021	Alaska State Virology Laboratory	Alaska State Virology Laboratory	Stephanie DeRonde, Elva House, Lisa Smith, Ph.D., Jack Chen, Ph.D.
hCoV-19/USA/AK-PHL7214/2021	EPI_ISL_1195938	2/16/2021	Alaska State Virology Laboratory	Alaska State Virology Laboratory	Stephanie DeRonde, Elva House, Lisa Smith, Ph.D., Jack Chen, Ph.D.
hCoV-19/USA/AK-PHL7215/2021	EPI_ISL_1195939	2/16/2021	Alaska State Virology Laboratory	Alaska State Virology Laboratory	Stephanie DeRonde, Elva House, Lisa Smith, Ph.D., Jack Chen, Ph.D.
hCoV-19/USA/AK-PHL7216/2021	EPI_ISL_1195940	2/16/2021	Alaska State Virology Laboratory	Alaska State Virology Laboratory	Stephanie DeRonde, Elva House, Lisa Smith, Ph.D., Jack Chen, Ph.D.
hCoV-19/USA/AK-PHL7217/2021	EPI_ISL_1195941	2/16/2021	Alaska State Virology Laboratory	Alaska State Virology Laboratory	Stephanie DeRonde, Elva House, Lisa Smith, Ph.D., Jack Chen, Ph.D.
hCoV-19/USA/AK-PHL7218/2021	EPI_ISL_1195942	2/16/2021	Alaska State Virology Laboratory	Alaska State Virology Laboratory	Stephanie DeRonde, Elva House, Lisa Smith, Ph.D., Jack Chen, Ph.D.
hCoV-19/USA/AK-PHL7219/2021	EPI_ISL_1195943	2/16/2021	Alaska State Virology Laboratory	Alaska State Virology Laboratory	Stephanie DeRonde, Elva House, Lisa Smith, Ph.D., Jack Chen, Ph.D.
hCoV-19/USA/AK-PHL7221/2021	EPI_ISL_1195945	2/16/2021	Alaska State Virology Laboratory	Alaska State Virology Laboratory	Stephanie DeRonde, Elva House, Lisa Smith, Ph.D., Jack Chen, Ph.D.
hCoV-19/USA/AK-PHL7314/2021	EPI_ISL_1195964	2/17/2021	Alaska State Virology Laboratory	Alaska State Virology Laboratory	Stephanie DeRonde, Elva House, Lisa Smith, Ph.D., Jack Chen, Ph.D.
hCoV-19/USA/AK-PHL7315/2021	EPI_ISL_1195965	2/7/2021	Alaska State Virology Laboratory	Alaska State Virology Laboratory	Stephanie DeRonde, Elva House, Lisa Smith, Ph.D., Jack Chen, Ph.D.
hCoV-19/USA/AK-PHL7321/2021	EPI_ISL_1195968	2/17/2021	Alaska State Virology Laboratory	Alaska State Virology Laboratory	Stephanie DeRonde, Elva House, Lisa Smith, Ph.D., Jack Chen, Ph.D.
hCoV-19/USA/AK-PHL7322/2021	EPI_ISL_1195969	2/17/2021	Alaska State Virology Laboratory	Alaska State Virology Laboratory	Stephanie DeRonde, Elva House, Lisa Smith, Ph.D., Jack Chen, Ph.D.
hCoV-19/USA/AK-PHL7324/2021	EPI_ISL_1195971	2/17/2021	Alaska State Virology Laboratory	Alaska State Virology Laboratory	Stephanie DeRonde, Elva House, Lisa Smith, Ph.D., Jack Chen, Ph.D.
hCoV-19/USA/AK-PHL7325/2021	EPI_ISL_1195972	2/17/2021	Alaska State Virology Laboratory	Alaska State Virology Laboratory	Stephanie DeRonde, Elva House, Lisa Smith, Ph.D., Jack Chen, Ph.D.
hCoV-19/USA/AK-PHL7326/2021	EPI_ISL_1195973	2/17/2021	Alaska State Virology Laboratory	Alaska State Virology Laboratory	Stephanie DeRonde, Elva House, Lisa Smith, Ph.D., Jack Chen, Ph.D.
hCoV-19/USA/AK-PHL7327/2021	EPI_ISL_1195974	2/18/2021	Alaska State Virology Laboratory	Alaska State Virology Laboratory	Stephanie DeRonde, Elva House, Lisa Smith, Ph.D., Jack Chen, Ph.D.
hCoV-19/USA/AK-PHL7328/2021	EPI_ISL_1195975	2/18/2021	Alaska State Virology Laboratory	Alaska State Virology Laboratory	Stephanie DeRonde, Elva House, Lisa Smith, Ph.D., Jack Chen, Ph.D.
hCoV-19/USA/AK-PHL7329/2021	EPI_ISL_1195976	2/18/2021	Alaska State Virology Laboratory	Alaska State Virology Laboratory	Stephanie DeRonde, Elva House, Lisa Smith, Ph.D., Jack Chen, Ph.D.
hCoV-19/USA/AK-PHL7330/2021	EPI_ISL_1195977	2/18/2021	Alaska State Virology Laboratory	Alaska State Virology Laboratory	Stephanie DeRonde, Elva House, Lisa Smith, Ph.D., Jack Chen, Ph.D.
hCoV-19/USA/AK-PHL7331/2021	EPI_ISL_1195978	2/18/2021	Alaska State Virology Laboratory	Alaska State Virology Laboratory	Stephanie DeRonde, Elva House, Lisa Smith, Ph.D., Jack Chen, Ph.D.
hCoV-19/USA/AK-PHL7332/2021	EPI_ISL_1195979	2/17/2021	Alaska State Virology Laboratory	Alaska State Virology Laboratory	Stephanie DeRonde, Elva House, Lisa Smith, Ph.D., Jack Chen, Ph.D.
hCoV-19/USA/AK-PHL7336/2021	EPI_ISL_1195980	2/17/2021	Alaska State Virology Laboratory	Alaska State Virology Laboratory	Stephanie DeRonde, Elva House, Lisa Smith, Ph.D., Jack Chen, Ph.D.
hCoV-19/USA/AK-PHL7337/2021	EPI_ISL_1195981	2/19/2021	Alaska State Virology Laboratory	Alaska State Virology Laboratory	Stephanie DeRonde, Elva House, Lisa Smith, Ph.D., Jack Chen, Ph.D.
hCoV-19/USA/AK-PHL7338/2021	EPI_ISL_1195982	2/19/2021	Alaska State Virology Laboratory	Alaska State Virology Laboratory	Stephanie DeRonde, Elva House, Lisa Smith, Ph.D., Jack Chen, Ph.D.
hCoV-19/USA/AK-PHL7339/2021	EPI_ISL_1195983	2/18/2021	Alaska State Virology Laboratory	Alaska State Virology Laboratory	Stephanie DeRonde, Elva House, Lisa Smith, Ph.D., Jack Chen, Ph.D.
hCoV-19/USA/AK-PHL7340/2021	EPI_ISL_1195984	2/18/2021	Alaska State Virology Laboratory	Alaska State Virology Laboratory	Stephanie DeRonde, Elva House, Lisa Smith, Ph.D., Jack Chen, Ph.D.
hCoV-19/USA/AK-PHL7343/2021	EPI_ISL_1195985	2/19/2021	Alaska State Virology Laboratory	Alaska State Virology Laboratory	Stephanie DeRonde, Elva House, Lisa Smith, Ph.D., Jack Chen, Ph.D.
hCoV-19/USA/AK-PHL7344/2021	EPI_ISL_1195986	2/19/2021	Alaska State Virology Laboratory	Alaska State Virology Laboratory	Stephanie DeRonde, Elva House, Lisa Smith, Ph.D., Jack Chen, Ph.D.
hCoV-19/USA/AK-PHL7345/2021	EPI_ISL_1195987	2/19/2021	Alaska State Virology Laboratory	Alaska State Virology Laboratory	Stephanie DeRonde, Elva House, Lisa Smith, Ph.D., Jack Chen, Ph.D.

hCoV-19/USA/AK-PHL7346/2021	EPI_ISL_1195988	2/19/2021	Alaska State Virology Laboratory	Alaska State Virology Laboratory	Stephanie DeRonde, Elva House, Lisa Smith, Ph.D., Jack Chen, Ph.D.
hCoV-19/USA/AK-PHL7349/2021	EPI_ISL_1195990	2/20/2021	Alaska State Virology Laboratory	Alaska State Virology Laboratory	Stephanie DeRonde, Elva House, Lisa Smith, Ph.D., Jack Chen, Ph.D.
hCoV-19/USA/AK-PHL7350/2021	EPI_ISL_1195991	2/18/2021	Alaska State Virology Laboratory	Alaska State Virology Laboratory	Stephanie DeRonde, Elva House, Lisa Smith, Ph.D., Jack Chen, Ph.D.
hCoV-19/USA/AK-PHL7351/2021	EPI_ISL_1195992	2/19/2021	Alaska State Virology Laboratory	Alaska State Virology Laboratory	Stephanie DeRonde, Elva House, Lisa Smith, Ph.D., Jack Chen, Ph.D.
hCoV-19/USA/AK-PHL7352/2021	EPI_ISL_1195993	2/20/2021	Alaska State Virology Laboratory	Alaska State Virology Laboratory	Stephanie DeRonde, Elva House, Lisa Smith, Ph.D., Jack Chen, Ph.D.
hCoV-19/USA/AK-PHL7353/2021	EPI_ISL_1195994	2/20/2021	Alaska State Virology Laboratory	Alaska State Virology Laboratory	Stephanie DeRonde, Elva House, Lisa Smith, Ph.D., Jack Chen, Ph.D.
hCoV-19/USA/AK-PHL7354/2021	EPI_ISL_1195995	2/20/2021	Alaska State Virology Laboratory	Alaska State Virology Laboratory	Stephanie DeRonde, Elva House, Lisa Smith, Ph.D., Jack Chen, Ph.D.
hCoV-19/USA/AK-PHL7356/2021	EPI_ISL_1195996	2/21/2021	Alaska State Virology Laboratory	Alaska State Virology Laboratory	Stephanie DeRonde, Elva House, Lisa Smith, Ph.D., Jack Chen, Ph.D.
hCoV-19/USA/AK-PHL7357/2021	EPI_ISL_1195997	2/21/2021	Alaska State Virology Laboratory	Alaska State Virology Laboratory	Stephanie DeRonde, Elva House, Lisa Smith, Ph.D., Jack Chen, Ph.D.
hCoV-19/USA/AK-PHL7359/2021	EPI_ISL_1195999	2/20/2021	Alaska State Virology Laboratory	Alaska State Virology Laboratory	Stephanie DeRonde, Elva House, Lisa Smith, Ph.D., Jack Chen, Ph.D.
hCoV-19/USA/AK-PHL7813/2021	EPI_ISL_1575036	3/30/2021	Alaska State Virology Laboratory	Alaska State Virology Laboratory	Stephanie DeRonde, Elva House, Lisa Smith, Ph.D., Jack Chen, Ph.D.
hCoV-19/USA/AK-PHL7817/2021	EPI_ISL_1575040	3/30/2021	Alaska State Virology Laboratory	Alaska State Virology Laboratory	Stephanie DeRonde, Elva House, Lisa Smith, Ph.D., Jack Chen, Ph.D.
hCoV-19/USA/AK-PHL7825/2021	EPI_ISL_1575046	3/29/2021	Alaska State Virology Laboratory	Alaska State Virology Laboratory	Stephanie DeRonde, Elva House, Lisa Smith, Ph.D., Jack Chen, Ph.D.
hCoV-19/USA/AK-PHL7827/2021	EPI_ISL_1575048	3/29/2021	Alaska State Virology Laboratory	Alaska State Virology Laboratory	Stephanie DeRonde, Elva House, Lisa Smith, Ph.D., Jack Chen, Ph.D.
hCoV-19/USA/AK-PHL7828/2021	EPI_ISL_1575049	3/29/2021	Alaska State Virology Laboratory	Alaska State Virology Laboratory	Stephanie DeRonde, Elva House, Lisa Smith, Ph.D., Jack Chen, Ph.D.
hCoV-19/USA/AK-PHL7835/2021	EPI_ISL_1575056	3/30/2021	Alaska State Virology Laboratory	Alaska State Virology Laboratory	Stephanie DeRonde, Elva House, Lisa Smith, Ph.D., Jack Chen, Ph.D.
hCoV-19/USA/AK-PHL7842/2021	EPI_ISL_1575063	3/29/2021	Alaska State Virology Laboratory	Alaska State Virology Laboratory	Stephanie DeRonde, Elva House, Lisa Smith, Ph.D., Jack Chen, Ph.D.
hCoV-19/USA/AK-PHL7843/2021	EPI_ISL_1575064	3/31/2021	Alaska State Virology Laboratory	Alaska State Virology Laboratory	Stephanie DeRonde, Elva House, Lisa Smith, Ph.D., Jack Chen, Ph.D.
hCoV-19/USA/AK-PHL7850/2021	EPI_ISL_1575071	3/30/2021	Alaska State Virology Laboratory	Alaska State Virology Laboratory	Stephanie DeRonde, Elva House, Lisa Smith, Ph.D., Jack Chen, Ph.D.
hCoV-19/USA/AK-PHL7854/2021	EPI_ISL_1575075	3/30/2021	Alaska State Virology Laboratory	Alaska State Virology Laboratory	Stephanie DeRonde, Elva House, Lisa Smith, Ph.D., Jack Chen, Ph.D.
hCoV-19/USA/AK-PHL7857/2021	EPI_ISL_1575078	3/30/2021	Alaska State Virology Laboratory	Alaska State Virology Laboratory	Stephanie DeRonde, Elva House, Lisa Smith, Ph.D., Jack Chen, Ph.D.
hCoV-19/USA/AK-PHL7858/2021	EPI_ISL_1575079	3/30/2021	Alaska State Virology Laboratory	Alaska State Virology Laboratory	Stephanie DeRonde, Elva House, Lisa Smith, Ph.D., Jack Chen, Ph.D.
hCoV-19/USA/AK-PHL7788/2021	EPI_ISL_1575096	3/23/2021	Alaska State Virology Laboratory	Alaska State Virology Laboratory	Stephanie DeRonde, Elva House, Lisa Smith, Ph.D., Jack Chen, Ph.D.
hCoV-19/USA/AK-PHL7806/2021	EPI_ISL_1575105	3/29/2021	Alaska State Virology Laboratory	Alaska State Virology Laboratory	Stephanie DeRonde, Elva House, Lisa Smith, Ph.D., Jack Chen, Ph.D.
hCoV-19/USA/AK-PHL7392/2021	EPI_ISL_1743698	2/8/2021	Alaska State Virology Laboratory	Alaska State Virology Laboratory	Stephanie DeRonde, Elva House, Lisa Smith, Ph.D., Jack Chen, Ph.D.
hCoV-19/USA/AK-PHL7393/2021	EPI_ISL_1743699	2/19/2021	Alaska State Virology Laboratory	Alaska State Virology Laboratory	Stephanie DeRonde, Elva House, Lisa Smith, Ph.D., Jack Chen, Ph.D.
hCoV-19/USA/AK-PHL7400/2021	EPI_ISL_1743703	2/25/2021	Alaska State Virology Laboratory	Alaska State Virology Laboratory	Stephanie DeRonde, Elva House, Lisa Smith, Ph.D., Jack Chen, Ph.D.
hCoV-19/USA/AK-PHL7402/2021	EPI_ISL_1743704	2/11/2021	Alaska State Virology Laboratory	Alaska State Virology Laboratory	Stephanie DeRonde, Elva House, Lisa Smith, Ph.D., Jack Chen, Ph.D.
hCoV-19/USA/AK-PHL7414/2021	EPI_ISL_1743712	3/6/2021	Alaska State Virology Laboratory	Alaska State Virology Laboratory	Stephanie DeRonde, Elva House, Lisa Smith, Ph.D., Jack Chen, Ph.D.
hCoV-19/USA/AK-PHL7411/2021	EPI_ISL_1743711	2/22/2021	Alaska State Virology Laboratory	Alaska State Virology Laboratory	Stephanie DeRonde, Elva House, Lisa Smith, Ph.D., Jack Chen, Ph.D.
hCoV-19/USA/AK-PHL9110/2021	EPI_ISL_2421684	5/23/2021	Alaska State Virology Laboratory	Alaska State Virology Laboratory	Stephanie DeRonde, Elva House, Jacob Zidek, Lisa Smith, Ph.D., Jack Chen, Ph.D.
hCoV-19/USA/AK-PHL9112/2021	EPI_ISL_2421687	5/23/2021	Alaska State Virology Laboratory	Alaska State Virology Laboratory	Stephanie DeRonde, Elva House, Jacob Zidek, Lisa Smith, Ph.D., Jack Chen, Ph.D.
hCoV-19/USA/AK-PHL9114/2021	EPI_ISL_2421690	5/20/2021	Alaska State Virology Laboratory	Alaska State Virology Laboratory	Stephanie DeRonde, Elva House, Jacob Zidek, Lisa Smith, Ph.D., Jack Chen, Ph.D.
hCoV-19/USA/AK-PHL9115/2021	EPI_ISL_2421691	5/24/2021	Alaska State Virology Laboratory	Alaska State Virology Laboratory	Stephanie DeRonde, Elva House, Jacob Zidek, Lisa Smith, Ph.D., Jack Chen, Ph.D.
hCoV-19/USA/AK-PHL9116/2021	EPI_ISL_2421693	5/24/2021	Alaska State Virology Laboratory	Alaska State Virology Laboratory	Stephanie DeRonde, Elva House, Jacob Zidek, Lisa Smith, Ph.D., Jack Chen, Ph.D.
hCoV-19/USA/AK-PHL9119/2021	EPI_ISL_2421697	5/24/2021	Alaska State Virology Laboratory	Alaska State Virology Laboratory	Stephanie DeRonde, Elva House, Jacob Zidek, Lisa Smith, Ph.D., Jack Chen, Ph.D.
hCoV-19/USA/AK-PHL9121/2021	EPI_ISL_2421700	5/19/2021	Alaska State Virology Laboratory	Alaska State Virology Laboratory	Stephanie DeRonde, Elva House, Jacob Zidek, Lisa Smith, Ph.D., Jack Chen, Ph.D.
hCoV-19/USA/AK-PHL9126/2021	EPI_ISL_2421705	5/25/2021	Alaska State Virology Laboratory	Alaska State Virology Laboratory	Stephanie DeRonde, Elva House, Jacob Zidek, Lisa Smith, Ph.D., Jack Chen, Ph.D.
hCoV-19/USA/AK-PHL9127/2021	EPI_ISL_2421706	5/25/2021	Alaska State Virology Laboratory	Alaska State Virology Laboratory	Stephanie DeRonde, Elva House, Jacob Zidek, Lisa Smith, Ph.D., Jack Chen, Ph.D.
hCoV-19/USA/AK-PHL9128/2021	EPI_ISL_2421707	5/25/2021	Alaska State Virology Laboratory	Alaska State Virology Laboratory	Stephanie DeRonde, Elva House, Jacob Zidek, Lisa Smith, Ph.D., Jack Chen, Ph.D.
hCoV-19/USA/AK-PHL9129/2021	EPI_ISL_2421709	5/25/2021	Alaska State Virology Laboratory	Alaska State Virology Laboratory	Stephanie DeRonde, Elva House, Jacob Zidek, Lisa Smith, Ph.D., Jack Chen, Ph.D.
hCoV-19/USA/AK-PHL9130/2021	EPI_ISL_2421710	5/24/2021	Alaska State Virology Laboratory	Alaska State Virology Laboratory	Stephanie DeRonde, Elva House, Jacob Zidek, Lisa Smith, Ph.D., Jack Chen, Ph.D.
hCoV-19/USA/AK-PHL9141/2021	EPI_ISL_2421717	5/19/2021	Alaska State Virology Laboratory	Alaska State Virology Laboratory	Stephanie DeRonde, Elva House, Jacob Zidek, Lisa Smith, Ph.D., Jack Chen, Ph.D.
hCoV-19/USA/AK-PHL9142/2021	EPI_ISL_2421718	5/24/2021	Alaska State Virology Laboratory	Alaska State Virology Laboratory	Stephanie DeRonde, Elva House, Jacob Zidek, Lisa Smith, Ph.D., Jack Chen, Ph.D.
hCoV-19/USA/AK-PHL9145/2021	EPI_ISL_2421720	4/19/2021	Alaska State Virology Laboratory	Alaska State Virology Laboratory	Stephanie DeRonde, Elva House, Jacob Zidek, Lisa Smith, Ph.D., Jack Chen, Ph.D.
hCoV-19/USA/AK-PHL9152/2021	EPI_ISL_2421726	5/23/2021	Alaska State Virology Laboratory	Alaska State Virology Laboratory	Stephanie DeRonde, Elva House, Jacob Zidek, Lisa Smith, Ph.D., Jack Chen, Ph.D.
hCoV-19/USA/AK-PHL8913/2021	EPI_ISL_2422047	5/16/2021	Alaska State Virology Laboratory	Alaska State Virology Laboratory	Stephanie DeRonde, Elva House, Jacob Zidek, Lisa Smith, Ph.D., Jack Chen, Ph.D.
hCoV-19/USA/AK-PHL8917/2021	EPI_ISL_2422051	5/15/2021	Alaska State Virology Laboratory	Alaska State Virology Laboratory	Stephanie DeRonde, Elva House, Jacob Zidek, Lisa Smith, Ph.D., Jack Chen, Ph.D.
hCoV-19/USA/AK-PHL8918/2021	EPI_ISL_2422053	5/16/2021	Alaska State Virology Laboratory	Alaska State Virology Laboratory	Stephanie DeRonde, Elva House, Jacob Zidek, Lisa Smith, Ph.D., Jack Chen, Ph.D.
hCoV-19/USA/AK-PHL8919/2021	EPI_ISL_2422054	5/16/2021	Alaska State Virology Laboratory	Alaska State Virology Laboratory	Stephanie DeRonde, Elva House, Jacob Zidek, Lisa Smith, Ph.D., Jack Chen, Ph.D.
hCoV-19/USA/AK-PHL8920/2021	EPI_ISL_2422056	5/16/2021	Alaska State Virology Laboratory	Alaska State Virology Laboratory	Stephanie DeRonde, Elva House, Jacob Zidek, Lisa Smith, Ph.D., Jack Chen, Ph.D.

hCoV-19/USA/AK-PHL8921/2021	EPI_ISL_2422057	5/15/2021	Alaska State Virology Laboratory	Alaska State Virology Laboratory	Stephanie DeRonde, Elva House, Jacob Zidek, Lisa Smith, Ph.D., Jack Chen, Ph.D.
hCoV-19/USA/AK-PHL8922/2021	EPI_ISL_2422059	5/15/2021	Alaska State Virology Laboratory	Alaska State Virology Laboratory	Stephanie DeRonde, Elva House, Jacob Zidek, Lisa Smith, Ph.D., Jack Chen, Ph.D.
hCoV-19/USA/AK-PHL8924/2021	EPI_ISL_2422062	5/16/2021	Alaska State Virology Laboratory	Alaska State Virology Laboratory	Stephanie DeRonde, Elva House, Jacob Zidek, Lisa Smith, Ph.D., Jack Chen, Ph.D.
hCoV-19/USA/AK-PHL8925/2021	EPI_ISL_2422063	5/14/2021	Alaska State Virology Laboratory	Alaska State Virology Laboratory	Stephanie DeRonde, Elva House, Jacob Zidek, Lisa Smith, Ph.D., Jack Chen, Ph.D.
hCoV-19/USA/AK-PHL8926/2021	EPI_ISL_2422065	5/17/2021	Alaska State Virology Laboratory	Alaska State Virology Laboratory	Stephanie DeRonde, Elva House, Jacob Zidek, Lisa Smith, Ph.D., Jack Chen, Ph.D.
hCoV-19/USA/AK-PHL8927/2021	EPI_ISL_2422066	5/17/2021	Alaska State Virology Laboratory	Alaska State Virology Laboratory	Stephanie DeRonde, Elva House, Jacob Zidek, Lisa Smith, Ph.D., Jack Chen, Ph.D.
hCoV-19/USA/AK-PHL8928/2021	EPI_ISL_2422068	5/17/2021	Alaska State Virology Laboratory	Alaska State Virology Laboratory	Stephanie DeRonde, Elva House, Jacob Zidek, Lisa Smith, Ph.D., Jack Chen, Ph.D.
hCoV-19/USA/AK-PHL8929/2021	EPI_ISL_2422069	5/17/2021	Alaska State Virology Laboratory	Alaska State Virology Laboratory	Stephanie DeRonde, Elva House, Jacob Zidek, Lisa Smith, Ph.D., Jack Chen, Ph.D.
hCoV-19/USA/AK-PHL8930/2021	EPI_ISL_2422071	5/17/2021	Alaska State Virology Laboratory	Alaska State Virology Laboratory	Stephanie DeRonde, Elva House, Jacob Zidek, Lisa Smith, Ph.D., Jack Chen, Ph.D.
hCoV-19/USA/AK-PHL8931/2021	EPI_ISL_2422072	5/17/2021	Alaska State Virology Laboratory	Alaska State Virology Laboratory	Stephanie DeRonde, Elva House, Jacob Zidek, Lisa Smith, Ph.D., Jack Chen, Ph.D.
hCoV-19/USA/AK-PHL8932/2021	EPI_ISL_2422074	5/17/2021	Alaska State Virology Laboratory	Alaska State Virology Laboratory	Stephanie DeRonde, Elva House, Jacob Zidek, Lisa Smith, Ph.D., Jack Chen, Ph.D.
hCoV-19/USA/AK-PHL8934/2021	EPI_ISL_2422077	5/17/2021	Alaska State Virology Laboratory	Alaska State Virology Laboratory	Stephanie DeRonde, Elva House, Jacob Zidek, Lisa Smith, Ph.D., Jack Chen, Ph.D.
hCoV-19/USA/AK-PHL8935/2021	EPI_ISL_2422078	5/17/2021	Alaska State Virology Laboratory	Alaska State Virology Laboratory	Stephanie DeRonde, Elva House, Jacob Zidek, Lisa Smith, Ph.D., Jack Chen, Ph.D.
hCoV-19/USA/AK-PHL8936/2021	EPI_ISL_2422080	5/17/2021	Alaska State Virology Laboratory	Alaska State Virology Laboratory	Stephanie DeRonde, Elva House, Jacob Zidek, Lisa Smith, Ph.D., Jack Chen, Ph.D.
hCoV-19/USA/AK-PHL8937/2021	EPI_ISL_2422082	5/17/2021	Alaska State Virology Laboratory	Alaska State Virology Laboratory	Stephanie DeRonde, Elva House, Jacob Zidek, Lisa Smith, Ph.D., Jack Chen, Ph.D.
hCoV-19/USA/AK-PHL8940/2021	EPI_ISL_2422086	5/17/2021	Alaska State Virology Laboratory	Alaska State Virology Laboratory	Stephanie DeRonde, Elva House, Jacob Zidek, Lisa Smith, Ph.D., Jack Chen, Ph.D.
hCoV-19/USA/AK-PHL8941/2021	EPI_ISL_2422088	5/17/2021	Alaska State Virology Laboratory	Alaska State Virology Laboratory	Stephanie DeRonde, Elva House, Jacob Zidek, Lisa Smith, Ph.D., Jack Chen, Ph.D.
hCoV-19/USA/AK-PHL8943/2021	EPI_ISL_2422091	5/16/2021	Alaska State Virology Laboratory	Alaska State Virology Laboratory	Stephanie DeRonde, Elva House, Jacob Zidek, Lisa Smith, Ph.D., Jack Chen, Ph.D.
hCoV-19/USA/AK-PHL8944/2021	EPI_ISL_2422092	5/16/2021	Alaska State Virology Laboratory	Alaska State Virology Laboratory	Stephanie DeRonde, Elva House, Jacob Zidek, Lisa Smith, Ph.D., Jack Chen, Ph.D.
hCoV-19/USA/AK-PHL8948/2021	EPI_ISL_2422095	5/17/2021	Alaska State Virology Laboratory	Alaska State Virology Laboratory	Stephanie DeRonde, Elva House, Jacob Zidek, Lisa Smith, Ph.D., Jack Chen, Ph.D.
hCoV-19/USA/AK-PHL8950/2021	EPI_ISL_2422098	5/17/2021	Alaska State Virology Laboratory	Alaska State Virology Laboratory	Stephanie DeRonde, Elva House, Jacob Zidek, Lisa Smith, Ph.D., Jack Chen, Ph.D.
hCoV-19/USA/AK-PHL8952/2021	EPI_ISL_2422101	5/17/2021	Alaska State Virology Laboratory	Alaska State Virology Laboratory	Stephanie DeRonde, Elva House, Jacob Zidek, Lisa Smith, Ph.D., Jack Chen, Ph.D.
hCoV-19/USA/AK-PHL8956/2021	EPI_ISL_2422104	5/16/2021	Alaska State Virology Laboratory	Alaska State Virology Laboratory	Stephanie DeRonde, Elva House, Jacob Zidek, Lisa Smith, Ph.D., Jack Chen, Ph.D.
hCoV-19/USA/AK-PHL8958/2021	EPI_ISL_2422108	5/16/2021	Alaska State Virology Laboratory	Alaska State Virology Laboratory	Stephanie DeRonde, Elva House, Jacob Zidek, Lisa Smith, Ph.D., Jack Chen, Ph.D.
hCoV-19/USA/AK-PHL9122/2021	EPI_ISL_2423412	5/19/2021	Alaska State Virology Laboratory	Alaska State Virology Laboratory	Stephanie DeRonde, Elva House, Jacob Zidek, Lisa Smith, Ph.D., Jack Chen, Ph.D.
hCoV-19/USA/AK-PHL9131/2021	EPI_ISL_2423413	5/24/2021	Alaska State Virology Laboratory	Alaska State Virology Laboratory	Stephanie DeRonde, Elva House, Jacob Zidek, Lisa Smith, Ph.D., Jack Chen, Ph.D.
hCoV-19/USA/AK-PHL9137/2021	EPI_ISL_2423414	5/24/2021	Alaska State Virology Laboratory	Alaska State Virology Laboratory	Stephanie DeRonde, Elva House, Jacob Zidek, Lisa Smith, Ph.D., Jack Chen, Ph.D.
hCoV-19/USA/AK-PHL9140/2021	EPI_ISL_2423415	4/27/2021	Alaska State Virology Laboratory	Alaska State Virology Laboratory	Stephanie DeRonde, Elva House, Jacob Zidek, Lisa Smith, Ph.D., Jack Chen, Ph.D.
hCoV-19/USA/AK-PHL8443/2021	EPI_ISL_2429086	4/27/2021	Alaska State Virology Laboratory	Alaska State Virology Laboratory	Stephanie DeRonde, Elva House, Jacob Zidek, Lisa Smith, Ph.D., Jack Chen, Ph.D.
hCoV-19/USA/AK-PHL8444/2021	EPI_ISL_2429087	4/27/2021	Alaska State Virology Laboratory	Alaska State Virology Laboratory	Stephanie DeRonde, Elva House, Jacob Zidek, Lisa Smith, Ph.D., Jack Chen, Ph.D.
hCoV-19/USA/AK-PHL8447/2021	EPI_ISL_2429090	4/26/2021	Alaska State Virology Laboratory	Alaska State Virology Laboratory	Stephanie DeRonde, Elva House, Jacob Zidek, Lisa Smith, Ph.D., Jack Chen, Ph.D.
hCoV-19/USA/AK-PHL8448/2021	EPI_ISL_2429091	4/27/2021	Alaska State Virology Laboratory	Alaska State Virology Laboratory	Stephanie DeRonde, Elva House, Jacob Zidek, Lisa Smith, Ph.D., Jack Chen, Ph.D.
hCoV-19/USA/AK-PHL8449/2021	EPI_ISL_2429092	4/27/2021	Alaska State Virology Laboratory	Alaska State Virology Laboratory	Stephanie DeRonde, Elva House, Jacob Zidek, Lisa Smith, Ph.D., Jack Chen, Ph.D.
hCoV-19/USA/AK-PHL8451/2021	EPI_ISL_2429094	4/26/2021	Alaska State Virology Laboratory	Alaska State Virology Laboratory	Stephanie DeRonde, Elva House, Jacob Zidek, Lisa Smith, Ph.D., Jack Chen, Ph.D.
hCoV-19/USA/AK-PHL8453/2021	EPI_ISL_2429096	4/27/2021	Alaska State Virology Laboratory	Alaska State Virology Laboratory	Stephanie DeRonde, Elva House, Jacob Zidek, Lisa Smith, Ph.D., Jack Chen, Ph.D.
hCoV-19/USA/AK-PHL8454/2021	EPI_ISL_2429097	4/27/2021	Alaska State Virology Laboratory	Alaska State Virology Laboratory	Stephanie DeRonde, Elva House, Jacob Zidek, Lisa Smith, Ph.D., Jack Chen, Ph.D.
hCoV-19/USA/AK-PHL8455/2021	EPI_ISL_2429098	4/26/2021	Alaska State Virology Laboratory	Alaska State Virology Laboratory	Stephanie DeRonde, Elva House, Jacob Zidek, Lisa Smith, Ph.D., Jack Chen, Ph.D.
hCoV-19/USA/AK-PHL8456/2021	EPI_ISL_2429099	4/26/2021	Alaska State Virology Laboratory	Alaska State Virology Laboratory	Stephanie DeRonde, Elva House, Jacob Zidek, Lisa Smith, Ph.D., Jack Chen, Ph.D.
hCoV-19/USA/AK-PHL8457/2021	EPI_ISL_2429100	4/26/2021	Alaska State Virology Laboratory	Alaska State Virology Laboratory	Stephanie DeRonde, Elva House, Jacob Zidek, Lisa Smith, Ph.D., Jack Chen, Ph.D.
hCoV-19/USA/AK-PHL8458/2021	EPI_ISL_2429101	4/26/2021	Alaska State Virology Laboratory	Alaska State Virology Laboratory	Stephanie DeRonde, Elva House, Jacob Zidek, Lisa Smith, Ph.D., Jack Chen, Ph.D.
hCoV-19/USA/AK-PHL8459/2021	EPI_ISL_2429102	4/26/2021	Alaska State Virology Laboratory	Alaska State Virology Laboratory	Stephanie DeRonde, Elva House, Jacob Zidek, Lisa Smith, Ph.D., Jack Chen, Ph.D.
hCoV-19/USA/AK-PHL8460/2021	EPI_ISL_2429103	4/26/2021	Alaska State Virology Laboratory	Alaska State Virology Laboratory	Stephanie DeRonde, Elva House, Jacob Zidek, Lisa Smith, Ph.D., Jack Chen, Ph.D.
hCoV-19/USA/AK-PHL8463/2021	EPI_ISL_2429106	4/26/2021	Alaska State Virology Laboratory	Alaska State Virology Laboratory	Stephanie DeRonde, Elva House, Jacob Zidek, Lisa Smith, Ph.D., Jack Chen, Ph.D.
hCoV-19/USA/AK-PHL8464/2021	EPI_ISL_2429107	4/27/2021	Alaska State Virology Laboratory	Alaska State Virology Laboratory	Stephanie DeRonde, Elva House, Jacob Zidek, Lisa Smith, Ph.D., Jack Chen, Ph.D.
hCoV-19/USA/AK-PHL8466/2021	EPI_ISL_2429109	4/18/2021	Alaska State Virology Laboratory	Alaska State Virology Laboratory	Stephanie DeRonde, Elva House, Jacob Zidek, Lisa Smith, Ph.D., Jack Chen, Ph.D.
hCoV-19/USA/AK-PHL8467/2021	EPI_ISL_2429110	4/21/2021	Alaska State Virology Laboratory	Alaska State Virology Laboratory	Stephanie DeRonde, Elva House, Jacob Zidek, Lisa Smith, Ph.D., Jack Chen, Ph.D.
hCoV-19/USA/AK-PHL8469/2021	EPI_ISL_2429112	4/18/2021	Alaska State Virology Laboratory	Alaska State Virology Laboratory	Stephanie DeRonde, Elva House, Jacob Zidek, Lisa Smith, Ph.D., Jack Chen, Ph.D.
hCoV-19/USA/AK-PHL8470/2021	EPI_ISL_2429113	4/18/2021	Alaska State Virology Laboratory	Alaska State Virology Laboratory	Stephanie DeRonde, Elva House, Jacob Zidek, Lisa Smith, Ph.D., Jack Chen, Ph.D.
hCoV-19/USA/AK-PHL8473/2021	EPI_ISL_2429116	4/19/2021	Alaska State Virology Laboratory	Alaska State Virology Laboratory	Stephanie DeRonde, Elva House, Jacob Zidek, Lisa Smith, Ph.D., Jack Chen, Ph.D.
hCoV-19/USA/AK-PHL8475/2021	EPI_ISL_2429118	4/27/2021	Alaska State Virology Laboratory	Alaska State Virology Laboratory	Stephanie DeRonde, Elva House, Jacob Zidek, Lisa Smith, Ph.D., Jack Chen, Ph.D.
hCoV-19/USA/AK-PHL8478/2021	EPI_ISL_2429119	4/26/2021	Alaska State Virology Laboratory	Alaska State Virology Laboratory	Stephanie DeRonde, Elva House, Jacob Zidek, Lisa Smith, Ph.D., Jack Chen, Ph.D.

hCoV-19/USA/AK-PHL8479/2021	EPI_ISL_2429120	4/26/2021	Alaska State Virology Laboratory	Alaska State Virology Laboratory	Stephanie DeRonde, Elva House, Jacob Zidek, Lisa Smith, Ph.D., Jack Chen, Ph.D.
hCoV-19/USA/AK-PHL7373/2021	EPI_ISL_2429121	2/13/2021	Alaska State Virology Laboratory	Alaska State Virology Laboratory	Stephanie DeRonde, Elva House, Jacob Zidek, Lisa Smith, Ph.D., Jack Chen, Ph.D.
hCoV-19/USA/AK-PHL8482/2021	EPI_ISL_2429122	3/25/2021	Alaska State Virology Laboratory	Alaska State Virology Laboratory	Stephanie DeRonde, Elva House, Jacob Zidek, Lisa Smith, Ph.D., Jack Chen, Ph.D.
hCoV-19/USA/AK-CDC-2-4449593/2021	EPI_ISL_2451937	5/17/2021	AK State Public Health Lab, State Health Department	Centers for Disease Control and Prevention Division of Viral Diseases, Pathogen Discovery	Mili Sheth, Sarah Nobles, Jasmine Padilla, Mark Burroughs, Shoshona Le, Katie Dillon, Peter Cook, Clinton R. Paden, Dhwani Batra, Krista Queen, Kristen Knipe, Dakota Howard, Yvette Unoarumhi, Darlene Wagner, Matthew Schmerer, Ben L. Rambo-Martin, Kristine Lacek, Sam Shepard, Alison Laufer Halpin, Dave Wentworth, Vivien Dugan, Suxiang Tong, Justin Lee
hCoV-19/USA/AK-CDC-2-4449455/2021	EPI_ISL_2451938	5/17/2021	AK State Public Health Lab, State Health Department	Centers for Disease Control and Prevention Division of Viral Diseases, Pathogen Discovery	Mili Sheth, Sarah Nobles, Jasmine Padilla, Mark Burroughs, Shoshona Le, Katie Dillon, Peter Cook, Clinton R. Paden, Dhwani Batra, Krista Queen, Kristen Knipe, Dakota Howard, Yvette Unoarumhi, Darlene Wagner, Matthew Schmerer, Ben L. Rambo-Martin, Kristine Lacek, Sam Shepard, Alison Laufer Halpin, Dave Wentworth, Vivien Dugan, Suxiang Tong, Justin Lee
hCoV-19/USA/AK-CDC-2-4449673/2021	EPI_ISL_2451940	5/19/2021	AK State Public Health Lab, State Health Department	Centers for Disease Control and Prevention Division of Viral Diseases, Pathogen Discovery	Mili Sheth, Sarah Nobles, Jasmine Padilla, Mark Burroughs, Shoshona Le, Katie Dillon, Peter Cook, Clinton R. Paden, Dhwani Batra, Krista Queen, Kristen Knipe, Dakota Howard, Yvette Unoarumhi, Darlene Wagner, Matthew Schmerer, Ben L. Rambo-Martin, Kristine Lacek, Sam Shepard, Alison Laufer Halpin, Dave Wentworth, Vivien Dugan, Suxiang Tong, Justin Lee
hCoV-19/USA/AK-CDC-2-4449277/2021	EPI_ISL_2451943	5/17/2021	AK State Public Health Lab, State Health Department	Centers for Disease Control and Prevention Division of Viral Diseases, Pathogen Discovery	Mili Sheth, Sarah Nobles, Jasmine Padilla, Mark Burroughs, Shoshona Le, Katie Dillon, Peter Cook, Clinton R. Paden, Dhwani Batra, Krista Queen, Kristen Knipe, Dakota Howard, Yvette Unoarumhi, Darlene Wagner, Matthew Schmerer, Ben L. Rambo-Martin, Kristine Lacek, Sam Shepard, Alison Laufer Halpin, Dave Wentworth, Vivien Dugan, Suxiang Tong, Justin Lee
hCoV-19/USA/AK-CDC-2-4449459/2021	EPI_ISL_2451945	5/16/2021	AK State Public Health Lab, State Health Department	Centers for Disease Control and Prevention Division of Viral Diseases, Pathogen Discovery	Mili Sheth, Sarah Nobles, Jasmine Padilla, Mark Burroughs, Shoshona Le, Katie Dillon, Peter Cook, Clinton R. Paden, Dhwani Batra, Krista Queen, Kristen Knipe, Dakota Howard, Yvette Unoarumhi, Darlene Wagner, Matthew Schmerer, Ben L. Rambo-Martin, Kristine Lacek, Sam Shepard, Alison Laufer Halpin, Dave Wentworth, Vivien Dugan, Suxiang Tong, Justin Lee
hCoV-19/USA/AK-CDC-2-4449298/2021	EPI_ISL_2451947	5/16/2021	AK State Public Health Lab, State Health Department	Centers for Disease Control and Prevention Division of Viral Diseases, Pathogen Discovery	Mili Sheth, Sarah Nobles, Jasmine Padilla, Mark Burroughs, Shoshona Le, Katie Dillon, Peter Cook, Clinton R. Paden, Dhwani Batra, Krista Queen, Kristen Knipe, Dakota Howard, Yvette Unoarumhi, Darlene Wagner, Matthew Schmerer, Ben L. Rambo-Martin, Kristine Lacek, Sam Shepard, Alison Laufer Halpin, Dave Wentworth, Vivien Dugan, Suxiang Tong, Justin Lee
hCoV-19/USA/AK-CDC-2-4449591/2021	EPI_ISL_2451948	5/20/2021	AK State Public Health Lab, State Health Department	Centers for Disease Control and Prevention Division of Viral Diseases, Pathogen Discovery	Mili Sheth, Sarah Nobles, Jasmine Padilla, Mark Burroughs, Shoshona Le, Katie Dillon, Peter Cook, Clinton R. Paden, Dhwani Batra, Krista Queen, Kristen Knipe, Dakota Howard, Yvette Unoarumhi, Darlene Wagner, Matthew Schmerer, Ben L. Rambo-Martin, Kristine Lacek, Sam Shepard, Alison Laufer Halpin, Dave Wentworth, Vivien Dugan, Suxiang Tong, Justin Lee
hCoV-19/USA/AK-PHL8419/2021	EPI_ISL_1967922	4/25/2021	Alaska State Virology Laboratory	Alaska State Virology Laboratory	Stephanie DeRonde, Elva House, Jacob Zidek, Lisa Smith, Ph.D., Jack Chen, Ph.D.
hCoV-19/USA/AK-PHL8441/2021	EPI_ISL_1967941	4/27/2021	Alaska State Virology Laboratory	Alaska State Virology Laboratory	Stephanie DeRonde, Elva House, Jacob Zidek, Lisa Smith, Ph.D., Jack Chen, Ph.D.
hCoV-19/USA/AK-PHL9202/2021	EPI_ISL_2454551	5/26/2021	Alaska State Virology Laboratory	Alaska State Virology Laboratory	Stephanie DeRonde, Elva House, Jacob Zidek, Lisa Smith, Ph.D., Jack Chen, Ph.D.
hCoV-19/USA/AK-PHL9203/2021	EPI_ISL_2454552	5/26/2021	Alaska State Virology Laboratory	Alaska State Virology Laboratory	Stephanie DeRonde, Elva House, Jacob Zidek, Lisa Smith, Ph.D., Jack Chen, Ph.D.
hCoV-19/USA/AK-PHL9204/2021	EPI_ISL_2454553	5/26/2021	Alaska State Virology Laboratory	Alaska State Virology Laboratory	Stephanie DeRonde, Elva House, Jacob Zidek, Lisa Smith, Ph.D., Jack Chen, Ph.D.
hCoV-19/USA/AK-PHL9205/2021	EPI_ISL_2454554	5/26/2021	Alaska State Virology Laboratory	Alaska State Virology Laboratory	Stephanie DeRonde, Elva House, Jacob Zidek, Lisa Smith, Ph.D., Jack Chen, Ph.D.
hCoV-19/USA/AK-PHL9206/2021	EPI_ISL_2454555	5/26/2021	Alaska State Virology Laboratory	Alaska State Virology Laboratory	Stephanie DeRonde, Elva House, Jacob Zidek, Lisa Smith, Ph.D., Jack Chen, Ph.D.
hCoV-19/USA/AK-PHL9209/2021	EPI_ISL_2454557	5/26/2021	Alaska State Virology Laboratory	Alaska State Virology Laboratory	Stephanie DeRonde, Elva House, Jacob Zidek, Lisa Smith, Ph.D., Jack Chen, Ph.D.
hCoV-19/USA/AK-PHL9210/2021	EPI_ISL_2454558	5/26/2021	Alaska State Virology Laboratory	Alaska State Virology Laboratory	Stephanie DeRonde, Elva House, Jacob Zidek, Lisa Smith, Ph.D., Jack Chen, Ph.D.
hCoV-19/USA/AK-PHL9212/2021	EPI_ISL_2454559	5/25/2021	Alaska State Virology Laboratory	Alaska State Virology Laboratory	Stephanie DeRonde, Elva House, Jacob Zidek, Lisa Smith, Ph.D., Jack Chen, Ph.D.
hCoV-19/USA/AK-PHL9215/2021	EPI_ISL_2454560	5/26/2021	Alaska State Virology Laboratory	Alaska State Virology Laboratory	Stephanie DeRonde, Elva House, Jacob Zidek, Lisa Smith, Ph.D., Jack Chen, Ph.D.
hCoV-19/USA/AK-PHL9216/2021	EPI_ISL_2454561	5/27/2021	Alaska State Virology Laboratory	Alaska State Virology Laboratory	Stephanie DeRonde, Elva House, Jacob Zidek, Lisa Smith, Ph.D., Jack Chen, Ph.D.
hCoV-19/USA/AK-PHL9218/2021	EPI_ISL_2454563	5/27/2021	Alaska State Virology Laboratory	Alaska State Virology Laboratory	Stephanie DeRonde, Elva House, Jacob Zidek, Lisa Smith, Ph.D., Jack Chen, Ph.D.
hCoV-19/USA/AK-PHL9219/2021	EPI_ISL_2454564	5/27/2021	Alaska State Virology Laboratory	Alaska State Virology Laboratory	Stephanie DeRonde, Elva House, Jacob Zidek, Lisa Smith, Ph.D., Jack Chen, Ph.D.
hCoV-19/USA/AK-PHL9220/2021	EPI_ISL_2454565	5/27/2021	Alaska State Virology Laboratory	Alaska State Virology Laboratory	Stephanie DeRonde, Elva House, Jacob Zidek, Lisa Smith, Ph.D., Jack Chen, Ph.D.
hCoV-19/USA/AK-PHL9221/2021	EPI_ISL_2454566	5/27/2021	Alaska State Virology Laboratory	Alaska State Virology Laboratory	Stephanie DeRonde, Elva House, Jacob Zidek, Lisa Smith, Ph.D., Jack Chen, Ph.D.

hCoV-19/USA/AK-PHL9224/2021	EPI_ISL_2454567	5/27/2021	Alaska State Virology Laboratory	Alaska State Virology Laboratory	Stephanie DeRonde, Elva House, Jacob Zidek, Lisa Smith, Ph.D., Jack Chen, Ph.D.
hCoV-19/USA/AK-PHL9225/2021	EPI_ISL_2454568	5/27/2021	Alaska State Virology Laboratory	Alaska State Virology Laboratory	Stephanie DeRonde, Elva House, Jacob Zidek, Lisa Smith, Ph.D., Jack Chen, Ph.D.
hCoV-19/USA/AK-PHL9227/2021	EPI_ISL_2454569	5/21/2021	Alaska State Virology Laboratory	Alaska State Virology Laboratory	Stephanie DeRonde, Elva House, Jacob Zidek, Lisa Smith, Ph.D., Jack Chen, Ph.D.
hCoV-19/USA/AK-PHL9229/2021	EPI_ISL_2454570	5/26/2021	Alaska State Virology Laboratory	Alaska State Virology Laboratory	Stephanie DeRonde, Elva House, Jacob Zidek, Lisa Smith, Ph.D., Jack Chen, Ph.D.
hCoV-19/USA/AK-PHL9231/2021	EPI_ISL_2454571	4/16/2021	Alaska State Virology Laboratory	Alaska State Virology Laboratory	Stephanie DeRonde, Elva House, Jacob Zidek, Lisa Smith, Ph.D., Jack Chen, Ph.D.
hCoV-19/USA/AK-PHL9232/2021	EPI_ISL_2454572	5/24/2021	Alaska State Virology Laboratory	Alaska State Virology Laboratory	Stephanie DeRonde, Elva House, Jacob Zidek, Lisa Smith, Ph.D., Jack Chen, Ph.D.
hCoV-19/USA/AK-PHL9233/2021	EPI_ISL_2454573	5/27/2021	Alaska State Virology Laboratory	Alaska State Virology Laboratory	Stephanie DeRonde, Elva House, Jacob Zidek, Lisa Smith, Ph.D., Jack Chen, Ph.D.
hCoV-19/USA/AK-PHL9235/2021	EPI_ISL_2454575	5/28/2021	Alaska State Virology Laboratory	Alaska State Virology Laboratory	Stephanie DeRonde, Elva House, Jacob Zidek, Lisa Smith, Ph.D., Jack Chen, Ph.D.
hCoV-19/USA/AK-PHL9239/2021	EPI_ISL_2454578	5/28/2021	Alaska State Virology Laboratory	Alaska State Virology Laboratory	Stephanie DeRonde, Elva House, Jacob Zidek, Lisa Smith, Ph.D., Jack Chen, Ph.D.
hCoV-19/USA/AK-PHL9240/2021	EPI_ISL_2454579	5/28/2021	Alaska State Virology Laboratory	Alaska State Virology Laboratory	Stephanie DeRonde, Elva House, Jacob Zidek, Lisa Smith, Ph.D., Jack Chen, Ph.D.
hCoV-19/USA/AK-PHL9243/2021	EPI_ISL_2454580	5/29/2021	Alaska State Virology Laboratory	Alaska State Virology Laboratory	Stephanie DeRonde, Elva House, Jacob Zidek, Lisa Smith, Ph.D., Jack Chen, Ph.D.
hCoV-19/USA/AK-PHL7371/2021	EPI_ISL_2455328	2/25/2021	Alaska State Virology Laboratory	Alaska State Virology Laboratory	Stephanie DeRonde, Elva House, Jacob Zidek, Lisa Smith, Ph.D., Jack Chen, Ph.D.
hCoV-19/USA/AK-PHL9248/2021	EPI_ISL_2455417	5/29/2021	Alaska State Virology Laboratory	Alaska State Virology Laboratory	Stephanie DeRonde, Elva House, Jacob Zidek, Lisa Smith, Ph.D., Jack Chen, Ph.D.
hCoV-19/USA/AK-PHL9249/2021	EPI_ISL_2455418	5/29/2021	Alaska State Virology Laboratory	Alaska State Virology Laboratory	Stephanie DeRonde, Elva House, Jacob Zidek, Lisa Smith, Ph.D., Jack Chen, Ph.D.
hCoV-19/USA/AK-PHL9251/2021	EPI_ISL_2455420	5/30/2021	Alaska State Virology Laboratory	Alaska State Virology Laboratory	Stephanie DeRonde, Elva House, Jacob Zidek, Lisa Smith, Ph.D., Jack Chen, Ph.D.
hCoV-19/USA/AK-PHL9252/2021	EPI_ISL_2455421	5/27/2021	Alaska State Virology Laboratory	Alaska State Virology Laboratory	Stephanie DeRonde, Elva House, Jacob Zidek, Lisa Smith, Ph.D., Jack Chen, Ph.D.
hCoV-19/USA/AK-PHL9262/2021	EPI_ISL_2455427	5/18/2021	Alaska State Virology Laboratory	Alaska State Virology Laboratory	Stephanie DeRonde, Elva House, Jacob Zidek, Lisa Smith, Ph.D., Jack Chen, Ph.D.
hCoV-19/USA/AK-PHL9263/2021	EPI_ISL_2455428	5/28/2021	Alaska State Virology Laboratory	Alaska State Virology Laboratory	Stephanie DeRonde, Elva House, Jacob Zidek, Lisa Smith, Ph.D., Jack Chen, Ph.D.
hCoV-19/USA/AK-PHL9264/2021	EPI_ISL_2455429	5/27/2021	Alaska State Virology Laboratory	Alaska State Virology Laboratory	Stephanie DeRonde, Elva House, Jacob Zidek, Lisa Smith, Ph.D., Jack Chen, Ph.D.
hCoV-19/USA/AK-PHL9265/2021	EPI_ISL_2455430	5/27/2021	Alaska State Virology Laboratory	Alaska State Virology Laboratory	Stephanie DeRonde, Elva House, Jacob Zidek, Lisa Smith, Ph.D., Jack Chen, Ph.D.
hCoV-19/USA/AK-PHL9266/2021	EPI_ISL_2455431	5/26/2021	Alaska State Virology Laboratory	Alaska State Virology Laboratory	Stephanie DeRonde, Elva House, Jacob Zidek, Lisa Smith, Ph.D., Jack Chen, Ph.D.
hCoV-19/USA/AK-PHL9267/2021	EPI_ISL_2455432	5/29/2021	Alaska State Virology Laboratory	Alaska State Virology Laboratory	Stephanie DeRonde, Elva House, Jacob Zidek, Lisa Smith, Ph.D., Jack Chen, Ph.D.
hCoV-19/USA/AK-PHL9268/2021	EPI_ISL_2455433	5/28/2021	Alaska State Virology Laboratory	Alaska State Virology Laboratory	Stephanie DeRonde, Elva House, Jacob Zidek, Lisa Smith, Ph.D., Jack Chen, Ph.D.
hCoV-19/USA/AK-PHL9270/2021	EPI_ISL_2455434	5/28/2021	Alaska State Virology Laboratory	Alaska State Virology Laboratory	Stephanie DeRonde, Elva House, Jacob Zidek, Lisa Smith, Ph.D., Jack Chen, Ph.D.
hCoV-19/USA/AK-PHL9273/2021	EPI_ISL_2455436	5/6/2021	Alaska State Virology Laboratory	Alaska State Virology Laboratory	Stephanie DeRonde, Elva House, Jacob Zidek, Lisa Smith, Ph.D., Jack Chen, Ph.D.
hCoV-19/USA/AK-PHL9274/2021	EPI_ISL_2455437	5/5/2021	Alaska State Virology Laboratory	Alaska State Virology Laboratory	Stephanie DeRonde, Elva House, Jacob Zidek, Lisa Smith, Ph.D., Jack Chen, Ph.D.
hCoV-19/USA/AK-PHL9282/2021	EPI_ISL_2455442	5/8/2021	Alaska State Virology Laboratory	Alaska State Virology Laboratory	Stephanie DeRonde, Elva House, Jacob Zidek, Lisa Smith, Ph.D., Jack Chen, Ph.D.
hCoV-19/USA/AK-PHL9283/2021	EPI_ISL_2455443	5/8/2021	Alaska State Virology Laboratory	Alaska State Virology Laboratory	Stephanie DeRonde, Elva House, Jacob Zidek, Lisa Smith, Ph.D., Jack Chen, Ph.D.
hCoV-19/USA/AK-PHL9284/2021	EPI_ISL_2455444	5/8/2021	Alaska State Virology Laboratory	Alaska State Virology Laboratory	Stephanie DeRonde, Elva House, Jacob Zidek, Lisa Smith, Ph.D., Jack Chen, Ph.D.
hCoV-19/USA/AK-PHL9285/2021	EPI_ISL_2455445	5/7/2021	Alaska State Virology Laboratory	Alaska State Virology Laboratory	Stephanie DeRonde, Elva House, Jacob Zidek, Lisa Smith, Ph.D., Jack Chen, Ph.D.
hCoV-19/USA/AK-PHL9286/2021	EPI_ISL_2455446	5/5/2021	Alaska State Virology Laboratory	Alaska State Virology Laboratory	Stephanie DeRonde, Elva House, Jacob Zidek, Lisa Smith, Ph.D., Jack Chen, Ph.D.
hCoV-19/USA/AK-PHL9288/2021	EPI_ISL_2455447	5/5/2021	Alaska State Virology Laboratory	Alaska State Virology Laboratory	Stephanie DeRonde, Elva House, Jacob Zidek, Lisa Smith, Ph.D., Jack Chen, Ph.D.
hCoV-19/USA/AK-PHL9289/2021	EPI_ISL_2455448	5/2/2021	Alaska State Virology Laboratory	Alaska State Virology Laboratory	Stephanie DeRonde, Elva House, Jacob Zidek, Lisa Smith, Ph.D., Jack Chen, Ph.D.
hCoV-19/USA/AK-PHL9291/2021	EPI_ISL_2455450	5/6/2021	Alaska State Virology Laboratory	Alaska State Virology Laboratory	Stephanie DeRonde, Elva House, Jacob Zidek, Lisa Smith, Ph.D., Jack Chen, Ph.D.
hCoV-19/USA/AK-PHL9242/2021	EPI_ISL_2458225	5/28/2021	Alaska State Virology Laboratory	Alaska State Virology Laboratory	Stephanie DeRonde, Elva House, Jacob Zidek, Lisa Smith, Ph.D., Jack Chen, Ph.D.
hCoV-19/USA/AK-PHL9245/2021	EPI_ISL_2458226	5/29/2021	Alaska State Virology Laboratory	Alaska State Virology Laboratory	Stephanie DeRonde, Elva House, Jacob Zidek, Lisa Smith, Ph.D., Jack Chen, Ph.D.
hCoV-19/USA/AK-PHL9246/2021	EPI_ISL_2458227	5/29/2021	Alaska State Virology Laboratory	Alaska State Virology Laboratory	Stephanie DeRonde, Elva House, Jacob Zidek, Lisa Smith, Ph.D., Jack Chen, Ph.D.
hCoV-19/USA/AK-PHL8787/2021	EPI_ISL_2187656	5/10/2021	Alaska State Virology Laboratory	Alaska State Virology Laboratory	Stephanie DeRonde, Elva House, Jacob Zidek, Lisa Smith, Ph.D., Jack Chen, Ph.D.
hCoV-19/USA/AK-PHL8775/2021	EPI_ISL_2187645	5/5/2021	Alaska State Virology Laboratory	Alaska State Virology Laboratory	Stephanie DeRonde, Elva House, Jacob Zidek, Lisa Smith, Ph.D., Jack Chen, Ph.D.
hCoV-19/USA/AK-PHL8810/2021	EPI_ISL_2187674	5/8/2021	Alaska State Virology Laboratory	Alaska State Virology Laboratory	Stephanie DeRonde, Elva House, Jacob Zidek, Lisa Smith, Ph.D., Jack Chen, Ph.D.
hCoV-19/USA/AK-PHL8816/2021	EPI_ISL_2187679	5/5/2021	Alaska State Virology Laboratory	Alaska State Virology Laboratory	Stephanie DeRonde, Elva House, Jacob Zidek, Lisa Smith, Ph.D., Jack Chen, Ph.D.
hCoV-19/USA/AK-PHL8817/2021	EPI_ISL_2187680	5/5/2021	Alaska State Virology Laboratory	Alaska State Virology Laboratory	Stephanie DeRonde, Elva House, Jacob Zidek, Lisa Smith, Ph.D., Jack Chen, Ph.D.
hCoV-19/USA/AK-PHL8988/2021	EPI_ISL_2466047	5/17/2021	Alaska State Virology Lab, Alaska State Public Health Labs	Alaska State Virology Lab, Alaska State Public Health Labs	DeRonde,S., House,E., Zidek,J., Redlinger,M., Smith,L., Chen,J.
hCoV-19/USA/AK-PHL9059/2021	EPI_ISL_2466048	5/19/2021	Alaska State Virology Lab, Alaska State Public Health Labs	Alaska State Virology Lab, Alaska State Public Health Labs	DeRonde,S., House,E., Zidek,J., Redlinger,M., Smith,L., Chen,J.
hCoV-19/USA/AK-PHL9060/2021	EPI_ISL_2466049	5/19/2021	Alaska State Virology Lab, Alaska State Public Health Labs	Alaska State Virology Lab, Alaska State Public Health Labs	DeRonde,S., House,E., Zidek,J., Redlinger,M., Smith,L., Chen,J.
hCoV-19/USA/AK-PHL9295/2021	EPI_ISL_2467798	5/31/2021	Alaska State Virology Laboratory	Alaska State Virology Laboratory	Stephanie DeRonde, Elva House, Jacob Zidek, Lisa Smith, Ph.D., Jack Chen, Ph.D.
hCoV-19/USA/AK-PHL9296/2021	EPI_ISL_2467799	5/31/2021	Alaska State Virology Laboratory	Alaska State Virology Laboratory	Stephanie DeRonde, Elva House, Jacob Zidek, Lisa Smith, Ph.D., Jack Chen, Ph.D.
hCoV-19/USA/AK-PHL9297/2021	EPI_ISL_2467800	5/31/2021	Alaska State Virology Laboratory	Alaska State Virology Laboratory	Stephanie DeRonde, Elva House, Jacob Zidek, Lisa Smith, Ph.D., Jack Chen, Ph.D.
hCoV-19/USA/AK-PHL9298/2021	EPI_ISL_2467801	5/31/2021	Alaska State Virology Laboratory	Alaska State Virology Laboratory	Stephanie DeRonde, Elva House, Jacob Zidek, Lisa Smith, Ph.D., Jack Chen, Ph.D.
hCoV-19/USA/AK-PHL9299/2021	EPI_ISL_2467802	5/31/2021	Alaska State Virology Laboratory	Alaska State Virology Laboratory	Stephanie DeRonde, Elva House, Jacob Zidek, Lisa Smith, Ph.D., Jack Chen, Ph.D.
hCoV-19/USA/AK-PHL9300/2021	EPI_ISL_2467803	5/31/2021	Alaska State Virology Laboratory	Alaska State Virology Laboratory	Stephanie DeRonde, Elva House, Jacob Zidek, Lisa Smith, Ph.D., Jack Chen, Ph.D.

hCoV-19/USA/AK-PHL9301/2021	EPI_ISL_2467804	5/31/2021	Alaska State Virology Laboratory	Alaska State Virology Laboratory	Stephanie DeRonde, Elva House, Jacob Zidek, Lisa Smith, Ph.D., Jack Chen, Ph.D.
hCoV-19/USA/AK-PHL9309/2021	EPI_ISL_2467805	6/1/2021	Alaska State Virology Laboratory	Alaska State Virology Laboratory	Stephanie DeRonde, Elva House, Jacob Zidek, Lisa Smith, Ph.D., Jack Chen, Ph.D.
hCoV-19/USA/AK-PHL9310/2021	EPI_ISL_2467806	6/1/2021	Alaska State Virology Laboratory	Alaska State Virology Laboratory	Stephanie DeRonde, Elva House, Jacob Zidek, Lisa Smith, Ph.D., Jack Chen, Ph.D.
hCoV-19/USA/AK-PHL9311/2021	EPI_ISL_2467807	6/1/2021	Alaska State Virology Laboratory	Alaska State Virology Laboratory	Stephanie DeRonde, Elva House, Jacob Zidek, Lisa Smith, Ph.D., Jack Chen, Ph.D.
hCoV-19/USA/AK-PHL9312/2021	EPI_ISL_2467808	6/1/2021	Alaska State Virology Laboratory	Alaska State Virology Laboratory	Stephanie DeRonde, Elva House, Jacob Zidek, Lisa Smith, Ph.D., Jack Chen, Ph.D.
hCoV-19/USA/AK-PHL9313/2021	EPI_ISL_2467809	6/1/2021	Alaska State Virology Laboratory	Alaska State Virology Laboratory	Stephanie DeRonde, Elva House, Jacob Zidek, Lisa Smith, Ph.D., Jack Chen, Ph.D.
hCoV-19/USA/AK-PHL9314/2021	EPI_ISL_2467810	6/1/2021	Alaska State Virology Laboratory	Alaska State Virology Laboratory	Stephanie DeRonde, Elva House, Jacob Zidek, Lisa Smith, Ph.D., Jack Chen, Ph.D.
hCoV-19/USA/AK-PHL9315/2021	EPI_ISL_2467811	6/1/2021	Alaska State Virology Laboratory	Alaska State Virology Laboratory	Stephanie DeRonde, Elva House, Jacob Zidek, Lisa Smith, Ph.D., Jack Chen, Ph.D.
hCoV-19/USA/AK-PHL9316/2021	EPI_ISL_2467812	6/1/2021	Alaska State Virology Laboratory	Alaska State Virology Laboratory	Stephanie DeRonde, Elva House, Jacob Zidek, Lisa Smith, Ph.D., Jack Chen, Ph.D.
hCoV-19/USA/AK-PHL9317/2021	EPI_ISL_2467813	6/1/2021	Alaska State Virology Laboratory	Alaska State Virology Laboratory	Stephanie DeRonde, Elva House, Jacob Zidek, Lisa Smith, Ph.D., Jack Chen, Ph.D.
hCoV-19/USA/AK-PHL9318/2021	EPI_ISL_2467814	6/1/2021	Alaska State Virology Laboratory	Alaska State Virology Laboratory	Stephanie DeRonde, Elva House, Jacob Zidek, Lisa Smith, Ph.D., Jack Chen, Ph.D.
hCoV-19/USA/AK-PHL9319/2021	EPI_ISL_2467815	6/1/2021	Alaska State Virology Laboratory	Alaska State Virology Laboratory	Stephanie DeRonde, Elva House, Jacob Zidek, Lisa Smith, Ph.D., Jack Chen, Ph.D.
hCoV-19/USA/AK-PHL9320/2021	EPI_ISL_2467816	6/1/2021	Alaska State Virology Laboratory	Alaska State Virology Laboratory	Stephanie DeRonde, Elva House, Jacob Zidek, Lisa Smith, Ph.D., Jack Chen, Ph.D.
hCoV-19/USA/AK-PHL9322/2021	EPI_ISL_2467817	5/7/2021	Alaska State Virology Laboratory	Alaska State Virology Laboratory	Stephanie DeRonde, Elva House, Jacob Zidek, Lisa Smith, Ph.D., Jack Chen, Ph.D.
hCoV-19/USA/AK-PHL9323/2021	EPI_ISL_2467818	5/7/2021	Alaska State Virology Laboratory	Alaska State Virology Laboratory	Stephanie DeRonde, Elva House, Jacob Zidek, Lisa Smith, Ph.D., Jack Chen, Ph.D.
hCoV-19/USA/AK-PHL9324/2021	EPI_ISL_2467819	5/7/2021	Alaska State Virology Laboratory	Alaska State Virology Laboratory	Stephanie DeRonde, Elva House, Jacob Zidek, Lisa Smith, Ph.D., Jack Chen, Ph.D.
hCoV-19/USA/AK-PHL9325/2021	EPI_ISL_2467820	5/7/2021	Alaska State Virology Laboratory	Alaska State Virology Laboratory	Stephanie DeRonde, Elva House, Jacob Zidek, Lisa Smith, Ph.D., Jack Chen, Ph.D.
hCoV-19/USA/AK-PHL9326/2021	EPI_ISL_2467821	5/7/2021	Alaska State Virology Laboratory	Alaska State Virology Laboratory	Stephanie DeRonde, Elva House, Jacob Zidek, Lisa Smith, Ph.D., Jack Chen, Ph.D.
hCoV-19/USA/AK-PHL9327/2021	EPI_ISL_2467822	5/7/2021	Alaska State Virology Laboratory	Alaska State Virology Laboratory	Stephanie DeRonde, Elva House, Jacob Zidek, Lisa Smith, Ph.D., Jack Chen, Ph.D.
hCoV-19/USA/AK-PHL9328/2021	EPI_ISL_2467823	5/7/2021	Alaska State Virology Laboratory	Alaska State Virology Laboratory	Stephanie DeRonde, Elva House, Jacob Zidek, Lisa Smith, Ph.D., Jack Chen, Ph.D.
hCoV-19/USA/AK-PHL9329/2021	EPI_ISL_2467824	5/7/2021	Alaska State Virology Laboratory	Alaska State Virology Laboratory	Stephanie DeRonde, Elva House, Jacob Zidek, Lisa Smith, Ph.D., Jack Chen, Ph.D.
hCoV-19/USA/AK-PHL9330/2021	EPI_ISL_2467825	5/7/2021	Alaska State Virology Laboratory	Alaska State Virology Laboratory	Stephanie DeRonde, Elva House, Jacob Zidek, Lisa Smith, Ph.D., Jack Chen, Ph.D.
hCoV-19/USA/AK-PHL9331/2021	EPI_ISL_2467826	5/7/2021	Alaska State Virology Laboratory	Alaska State Virology Laboratory	Stephanie DeRonde, Elva House, Jacob Zidek, Lisa Smith, Ph.D., Jack Chen, Ph.D.
hCoV-19/USA/AK-PHL9332/2021	EPI_ISL_2467827	5/7/2021	Alaska State Virology Laboratory	Alaska State Virology Laboratory	Stephanie DeRonde, Elva House, Jacob Zidek, Lisa Smith, Ph.D., Jack Chen, Ph.D.
hCoV-19/USA/AK-PHL9333/2021	EPI_ISL_2467828	5/7/2021	Alaska State Virology Laboratory	Alaska State Virology Laboratory	Stephanie DeRonde, Elva House, Jacob Zidek, Lisa Smith, Ph.D., Jack Chen, Ph.D.
hCoV-19/USA/AK-PHL9335/2021	EPI_ISL_2467829	5/7/2021	Alaska State Virology Laboratory	Alaska State Virology Laboratory	Stephanie DeRonde, Elva House, Jacob Zidek, Lisa Smith, Ph.D., Jack Chen, Ph.D.
hCoV-19/USA/AK-PHL9336/2021	EPI_ISL_2467830	5/8/2021	Alaska State Virology Laboratory	Alaska State Virology Laboratory	Stephanie DeRonde, Elva House, Jacob Zidek, Lisa Smith, Ph.D., Jack Chen, Ph.D.
hCoV-19/USA/AK-PHL9337/2021	EPI_ISL_2467831	5/8/2021	Alaska State Virology Laboratory	Alaska State Virology Laboratory	Stephanie DeRonde, Elva House, Jacob Zidek, Lisa Smith, Ph.D., Jack Chen, Ph.D.
hCoV-19/USA/AK-PHL9338/2021	EPI_ISL_2467832	5/8/2021	Alaska State Virology Laboratory	Alaska State Virology Laboratory	Stephanie DeRonde, Elva House, Jacob Zidek, Lisa Smith, Ph.D., Jack Chen, Ph.D.
hCoV-19/USA/AK-PHL9339/2021	EPI_ISL_2467833	5/8/2021	Alaska State Virology Laboratory	Alaska State Virology Laboratory	Stephanie DeRonde, Elva House, Jacob Zidek, Lisa Smith, Ph.D., Jack Chen, Ph.D.
hCoV-19/USA/AK-PHL9389/2021	EPI_ISL_2626689	5/22/2021	Alaska State Virology Laboratory	Alaska State Virology Laboratory	Stephanie DeRonde, Elva House, Jacob Zidek, Lisa Smith, Ph.D., Jack Chen, Ph.D.
hCoV-19/USA/AK-PHL9392/2021	EPI_ISL_2626690	5/18/2021	Alaska State Virology Laboratory	Alaska State Virology Laboratory	Stephanie DeRonde, Elva House, Jacob Zidek, Lisa Smith, Ph.D., Jack Chen, Ph.D.
hCoV-19/USA/AK-PHL9394/2021	EPI_ISL_2626691	6/2/2021	Alaska State Virology Laboratory	Alaska State Virology Laboratory	Stephanie DeRonde, Elva House, Jacob Zidek, Lisa Smith, Ph.D., Jack Chen, Ph.D.
hCoV-19/USA/AK-PHL9395/2021	EPI_ISL_2626692	6/5/2021	Alaska State Virology Laboratory	Alaska State Virology Laboratory	Stephanie DeRonde, Elva House, Jacob Zidek, Lisa Smith, Ph.D., Jack Chen, Ph.D.
hCoV-19/USA/AK-PHL9399/2021	EPI_ISL_2626693	6/3/2021	Alaska State Virology Laboratory	Alaska State Virology Laboratory	Stephanie DeRonde, Elva House, Jacob Zidek, Lisa Smith, Ph.D., Jack Chen, Ph.D.
hCoV-19/USA/AK-PHL9400/2021	EPI_ISL_2626694	6/3/2021	Alaska State Virology Laboratory	Alaska State Virology Laboratory	Stephanie DeRonde, Elva House, Jacob Zidek, Lisa Smith, Ph.D., Jack Chen, Ph.D.
hCoV-19/USA/AK-PHL9401/2021	EPI_ISL_2626695	6/3/2021	Alaska State Virology Laboratory	Alaska State Virology Laboratory	Stephanie DeRonde, Elva House, Jacob Zidek, Lisa Smith, Ph.D., Jack Chen, Ph.D.
hCoV-19/USA/AK-PHL9403/2021	EPI_ISL_2626696	5/21/2021	Alaska State Virology Laboratory	Alaska State Virology Laboratory	Stephanie DeRonde, Elva House, Jacob Zidek, Lisa Smith, Ph.D., Jack Chen, Ph.D.
hCoV-19/USA/AK-PHL9404/2021	EPI_ISL_2626697	5/23/2021	Alaska State Virology Laboratory	Alaska State Virology Laboratory	Stephanie DeRonde, Elva House, Jacob Zidek, Lisa Smith, Ph.D., Jack Chen, Ph.D.
hCoV-19/USA/AK-PHL9405/2021	EPI_ISL_2626698	5/22/2021	Alaska State Virology Laboratory	Alaska State Virology Laboratory	Stephanie DeRonde, Elva House, Jacob Zidek, Lisa Smith, Ph.D., Jack Chen, Ph.D.
hCoV-19/USA/AK-PHL9406/2021	EPI_ISL_2626699	5/24/2021	Alaska State Virology Laboratory	Alaska State Virology Laboratory	Stephanie DeRonde, Elva House, Jacob Zidek, Lisa Smith, Ph.D., Jack Chen, Ph.D.
hCoV-19/USA/AK-PHL9422/2021	EPI_ISL_2626703	5/31/2021	Alaska State Virology Laboratory	Alaska State Virology Laboratory	Stephanie DeRonde, Elva House, Jacob Zidek, Lisa Smith, Ph.D., Jack Chen, Ph.D.
hCoV-19/USA/AK-PHL9423/2021	EPI_ISL_2626704	6/6/2021	Alaska State Virology Laboratory	Alaska State Virology Laboratory	Stephanie DeRonde, Elva House, Jacob Zidek, Lisa Smith, Ph.D., Jack Chen, Ph.D.
hCoV-19/USA/AK-PHL9424/2021	EPI_ISL_2626705	6/5/2021	Alaska State Virology Laboratory	Alaska State Virology Laboratory	Stephanie DeRonde, Elva House, Jacob Zidek, Lisa Smith, Ph.D., Jack Chen, Ph.D.
hCoV-19/USA/AK-PHL9429/2021	EPI_ISL_2626709	6/4/2021	Alaska State Virology Laboratory	Alaska State Virology Laboratory	Stephanie DeRonde, Elva House, Jacob Zidek, Lisa Smith, Ph.D., Jack Chen, Ph.D.
hCoV-19/USA/AK-PHL9430/2021	EPI_ISL_2626710	6/4/2021	Alaska State Virology Laboratory	Alaska State Virology Laboratory	Stephanie DeRonde, Elva House, Jacob Zidek, Lisa Smith, Ph.D., Jack Chen, Ph.D.
hCoV-19/USA/AK-PHL9431/2021	EPI_ISL_2626711	6/4/2021	Alaska State Virology Laboratory	Alaska State Virology Laboratory	Stephanie DeRonde, Elva House, Jacob Zidek, Lisa Smith, Ph.D., Jack Chen, Ph.D.
hCoV-19/USA/AK-PHL9619/2021	EPI_ISL_2626715	6/7/2021	Alaska State Virology Laboratory	Alaska State Virology Laboratory	Stephanie DeRonde, Elva House, Jacob Zidek, Lisa Smith, Ph.D., Jack Chen, Ph.D.
hCoV-19/USA/AK-PHL9620/2021	EPI_ISL_2626716	6/7/2021	Alaska State Virology Laboratory	Alaska State Virology Laboratory	Stephanie DeRonde, Elva House, Jacob Zidek, Lisa Smith, Ph.D., Jack Chen, Ph.D.
hCoV-19/USA/AK-PHL9623/2021	EPI_ISL_2626719	6/7/2021	Alaska State Virology Laboratory	Alaska State Virology Laboratory	Stephanie DeRonde, Elva House, Jacob Zidek, Lisa Smith, Ph.D., Jack Chen, Ph.D.
hCoV-19/USA/AK-PHL9625/2021	EPI_ISL_2626721	6/7/2021	Alaska State Virology Laboratory	Alaska State Virology Laboratory	Stephanie DeRonde, Elva House, Jacob Zidek, Lisa Smith, Ph.D., Jack Chen, Ph.D.

hCoV-19/USA/AK-PHL9626/2021	EPI_ISL_2626722	6/7/2021	Alaska State Virology Laboratory	Alaska State Virology Laboratory	Stephanie DeRonde, Elva House, Jacob Zidek, Lisa Smith, Ph.D., Jack Chen, Ph.D.
hCoV-19/USA/AK-PHL9627/2021	EPI_ISL_2626723	6/8/2021	Alaska State Virology Laboratory	Alaska State Virology Laboratory	Stephanie DeRonde, Elva House, Jacob Zidek, Lisa Smith, Ph.D., Jack Chen, Ph.D.
hCoV-19/USA/AK-PHL9628/2021	EPI_ISL_2626724	6/8/2021	Alaska State Virology Laboratory	Alaska State Virology Laboratory	Stephanie DeRonde, Elva House, Jacob Zidek, Lisa Smith, Ph.D., Jack Chen, Ph.D.
hCoV-19/USA/AK-PHL9630/2021	EPI_ISL_2626726	6/8/2021	Alaska State Virology Laboratory	Alaska State Virology Laboratory	Stephanie DeRonde, Elva House, Jacob Zidek, Lisa Smith, Ph.D., Jack Chen, Ph.D.
hCoV-19/USA/AK-PHL9631/2021	EPI_ISL_2626727	6/8/2021	Alaska State Virology Laboratory	Alaska State Virology Laboratory	Stephanie DeRonde, Elva House, Jacob Zidek, Lisa Smith, Ph.D., Jack Chen, Ph.D.
hCoV-19/USA/AK-PHL9632/2021	EPI_ISL_2626728	6/8/2021	Alaska State Virology Laboratory	Alaska State Virology Laboratory	Stephanie DeRonde, Elva House, Jacob Zidek, Lisa Smith, Ph.D., Jack Chen, Ph.D.
hCoV-19/USA/AK-PHL9633/2021	EPI_ISL_2626729	6/7/2021	Alaska State Virology Laboratory	Alaska State Virology Laboratory	Stephanie DeRonde, Elva House, Jacob Zidek, Lisa Smith, Ph.D., Jack Chen, Ph.D.
hCoV-19/USA/AK-PHL9634/2021	EPI_ISL_2626730	5/31/2021	Alaska State Virology Laboratory	Alaska State Virology Laboratory	Stephanie DeRonde, Elva House, Jacob Zidek, Lisa Smith, Ph.D., Jack Chen, Ph.D.
hCoV-19/USA/AK-PHL9635/2021	EPI_ISL_2626731	6/12/2021	Alaska State Virology Laboratory	Alaska State Virology Laboratory	Stephanie DeRonde, Elva House, Jacob Zidek, Lisa Smith, Ph.D., Jack Chen, Ph.D.
hCoV-19/USA/AK-PHL9636/2021	EPI_ISL_2626732	6/12/2021	Alaska State Virology Laboratory	Alaska State Virology Laboratory	Stephanie DeRonde, Elva House, Jacob Zidek, Lisa Smith, Ph.D., Jack Chen, Ph.D.
hCoV-19/USA/AK-PHL9637/2021	EPI_ISL_2626733	6/12/2021	Alaska State Virology Laboratory	Alaska State Virology Laboratory	Stephanie DeRonde, Elva House, Jacob Zidek, Lisa Smith, Ph.D., Jack Chen, Ph.D.
hCoV-19/USA/AK-PHL9638/2021	EPI_ISL_2626734	6/9/2021	Alaska State Virology Laboratory	Alaska State Virology Laboratory	Stephanie DeRonde, Elva House, Jacob Zidek, Lisa Smith, Ph.D., Jack Chen, Ph.D.
hCoV-19/USA/AK-PHL9639/2021	EPI_ISL_2626735	6/12/2021	Alaska State Virology Laboratory	Alaska State Virology Laboratory	Stephanie DeRonde, Elva House, Jacob Zidek, Lisa Smith, Ph.D., Jack Chen, Ph.D.
hCoV-19/USA/AK-PHL9640/2021	EPI_ISL_2626736	6/11/2021	Alaska State Virology Laboratory	Alaska State Virology Laboratory	Stephanie DeRonde, Elva House, Jacob Zidek, Lisa Smith, Ph.D., Jack Chen, Ph.D.
hCoV-19/USA/AK-PHL9641/2021	EPI_ISL_2626737	6/12/2021	Alaska State Virology Laboratory	Alaska State Virology Laboratory	Stephanie DeRonde, Elva House, Jacob Zidek, Lisa Smith, Ph.D., Jack Chen, Ph.D.
hCoV-19/USA/AK-PHL9645/2021	EPI_ISL_2626741	6/11/2021	Alaska State Virology Laboratory	Alaska State Virology Laboratory	Stephanie DeRonde, Elva House, Jacob Zidek, Lisa Smith, Ph.D., Jack Chen, Ph.D.
hCoV-19/USA/AK-PHL9646/2021	EPI_ISL_2626742	6/11/2021	Alaska State Virology Laboratory	Alaska State Virology Laboratory	Stephanie DeRonde, Elva House, Jacob Zidek, Lisa Smith, Ph.D., Jack Chen, Ph.D.
hCoV-19/USA/AK-PHL9647/2021	EPI_ISL_2626743	6/11/2021	Alaska State Virology Laboratory	Alaska State Virology Laboratory	Stephanie DeRonde, Elva House, Jacob Zidek, Lisa Smith, Ph.D., Jack Chen, Ph.D.
hCoV-19/USA/AK-PHL9648/2021	EPI_ISL_2626744	6/10/2021	Alaska State Virology Laboratory	Alaska State Virology Laboratory	Stephanie DeRonde, Elva House, Jacob Zidek, Lisa Smith, Ph.D., Jack Chen, Ph.D.
hCoV-19/USA/AK-PHL9649/2021	EPI_ISL_2626745	6/9/2021	Alaska State Virology Laboratory	Alaska State Virology Laboratory	Stephanie DeRonde, Elva House, Jacob Zidek, Lisa Smith, Ph.D., Jack Chen, Ph.D.
hCoV-19/USA/AK-PHL9650/2021	EPI_ISL_2626746	6/10/2021	Alaska State Virology Laboratory	Alaska State Virology Laboratory	Stephanie DeRonde, Elva House, Jacob Zidek, Lisa Smith, Ph.D., Jack Chen, Ph.D.
hCoV-19/USA/AK-PHL9651/2021	EPI_ISL_2626747	6/10/2021	Alaska State Virology Laboratory	Alaska State Virology Laboratory	Stephanie DeRonde, Elva House, Jacob Zidek, Lisa Smith, Ph.D., Jack Chen, Ph.D.
hCoV-19/USA/AK-PHL9652/2021	EPI_ISL_2626748	6/7/2021	Alaska State Virology Laboratory	Alaska State Virology Laboratory	Stephanie DeRonde, Elva House, Jacob Zidek, Lisa Smith, Ph.D., Jack Chen, Ph.D.
hCoV-19/USA/AK-PHL9655/2021	EPI_ISL_2626750	6/13/2021	Alaska State Virology Laboratory	Alaska State Virology Laboratory	Stephanie DeRonde, Elva House, Jacob Zidek, Lisa Smith, Ph.D., Jack Chen, Ph.D.
hCoV-19/USA/AK-PHL9656/2021	EPI_ISL_2626751	6/13/2021	Alaska State Virology Laboratory	Alaska State Virology Laboratory	Stephanie DeRonde, Elva House, Jacob Zidek, Lisa Smith, Ph.D., Jack Chen, Ph.D.
hCoV-19/USA/AK-PHL9657/2021	EPI_ISL_2626752	6/13/2021	Alaska State Virology Laboratory	Alaska State Virology Laboratory	Stephanie DeRonde, Elva House, Jacob Zidek, Lisa Smith, Ph.D., Jack Chen, Ph.D.
hCoV-19/USA/AK-PHL9660/2021	EPI_ISL_2626754	6/13/2021	Alaska State Virology Laboratory	Alaska State Virology Laboratory	Stephanie DeRonde, Elva House, Jacob Zidek, Lisa Smith, Ph.D., Jack Chen, Ph.D.
hCoV-19/USA/AK-PHL9398/2021	EPI_ISL_2628193	6/1/2021	Alaska State Virology Laboratory	Alaska State Virology Laboratory	Stephanie DeRonde, Elva House, Jacob Zidek, Lisa Smith, Ph.D., Jack Chen, Ph.D.
hCoV-19/USA/AK-PHL8835/2021	EPI_ISL_2473680	5/12/2021	Alaska State Virology Laboratory	Alaska State Virology Laboratory	Stephanie DeRonde, Elva House, Jacob Zidek, Lisa Smith, Ph.D., Jack Chen, Ph.D.
hCoV-19/USA/AK-PHL8860/2021	EPI_ISL_2473681	5/15/2021	Alaska State Virology Laboratory	Alaska State Virology Laboratory	Stephanie DeRonde, Elva House, Jacob Zidek, Lisa Smith, Ph.D., Jack Chen, Ph.D.
hCoV-19/USA/AK-PHL8993/2021	EPI_ISL_2473682	5/14/2021	Alaska State Virology Laboratory	Alaska State Virology Laboratory	Stephanie DeRonde, Elva House, Jacob Zidek, Lisa Smith, Ph.D., Jack Chen, Ph.D.
hCoV-19/USA/AK-PHL9056/2021	EPI_ISL_2473683	5/19/2021	Alaska State Virology Laboratory	Alaska State Virology Laboratory	Stephanie DeRonde, Elva House, Jacob Zidek, Lisa Smith, Ph.D., Jack Chen, Ph.D.
hCoV-19/USA/AK-PHL9061/2021	EPI_ISL_2473684	5/19/2021	Alaska State Virology Laboratory	Alaska State Virology Laboratory	Stephanie DeRonde, Elva House, Jacob Zidek, Lisa Smith, Ph.D., Jack Chen, Ph.D.
hCoV-19/USA/AK-PHL9066/2021	EPI_ISL_2473685	5/20/2021	Alaska State Virology Laboratory	Alaska State Virology Laboratory	Stephanie DeRonde, Elva House, Jacob Zidek, Lisa Smith, Ph.D., Jack Chen, Ph.D.
hCoV-19/USA/AK-PHL9068/2021	EPI_ISL_2473686	5/20/2021	Alaska State Virology Laboratory	Alaska State Virology Laboratory	Stephanie DeRonde, Elva House, Jacob Zidek, Lisa Smith, Ph.D., Jack Chen, Ph.D.
hCoV-19/USA/AK-PHL9072/2021	EPI_ISL_2473687	5/20/2021	Alaska State Virology Laboratory	Alaska State Virology Laboratory	Stephanie DeRonde, Elva House, Jacob Zidek, Lisa Smith, Ph.D., Jack Chen, Ph.D.
hCoV-19/USA/AK-PHL9080/2021	EPI_ISL_2473688	5/21/2021	Alaska State Virology Laboratory	Alaska State Virology Laboratory	Stephanie DeRonde, Elva House, Jacob Zidek, Lisa Smith, Ph.D., Jack Chen, Ph.D.
hCoV-19/USA/AK-PHL9011/2021	EPI_ISL_2473689	5/18/2021	Alaska State Virology Laboratory	Alaska State Virology Laboratory	Stephanie DeRonde, Elva House, Jacob Zidek, Lisa Smith, Ph.D., Jack Chen, Ph.D.
hCoV-19/USA/AK-CDC-LC0066441/2021	EPI_ISL_2480855	5/24/2021	Laboratory Corporation of America	Centers for Disease Control and Prevention Division of Viral Diseases, Pathogen Discovery	Dakota Howard, Dhwani Batra, Peter W. Cook, Kara Moser, Adrian Paskey, Jason Caravas, Benjamin Rambo-Martin, Shatavia Morrison, Christopher Gulvick, Scott Sammons, Yvette Unoarumhi, Darlene Wagner, Matthew Schmerer, Minoo Agarwal, Eyad Almasri, Debbie Boles, Ayla Burns, Nuthawin Charoensri, Oren Cohen, Susan Countryman, Mary Ann Cristobal, Bobbi Croy, Suzanne Dale, Hrushikesh Deshmukh, Amanda Douglas, Vincent Drouillon, Marcia Eisenberg, Howard Engler, Rama Ghatti, Prashant Gupta, Susan Hicks, Jake Humphrey, Lax Iyer, Lisa Pfefferle, Manoj Jain, Matthew Robinson, Mohan Kolli, Brian Krueger, Tim Kuphal, Stanley Letovsky, Michael Levandoski, Craig Lukasik, Jonathan Meltzer, Brian Norvell, Mindy Nye, Scott Parker, Christos Petropoulos, John Pruitt, Steven Ragan, Scott Ryan, Mike Sapeta, Jana Schroth, Suresh Babu Selvaraju, Goran Stevovic, Amanda Suchanek, Andrea Throop, Lyndon Tilson, Thomas Urban, Joe Voshell, Kimberly Wagner, Jonathan Williams, Mary Williamson, Qian Zeng, Tricia Zwiefelhofer, Clinton R. Paden, Duncan MacCannell

hCoV-19/USA/AK-PHL8745/2021	EPI_ISL_2499753	5/9/2021	Alaska State Virology Laboratory	Alaska State Virology Laboratory	Stephanie DeRonde, Elva House, Jacob Zidek, Lisa Smith, Ph.D., Jack Chen, Ph.D.
hCoV-19/USA/AK-PHL8776/2021	EPI_ISL_2499754	5/6/2021	Alaska State Virology Laboratory	Alaska State Virology Laboratory	Stephanie DeRonde, Elva House, Jacob Zidek, Lisa Smith, Ph.D., Jack Chen, Ph.D.
hCoV-19/USA/AK-PHL8795/2021	EPI_ISL_2499755	5/6/2021	Alaska State Virology Laboratory	Alaska State Virology Laboratory	Stephanie DeRonde, Elva House, Jacob Zidek, Lisa Smith, Ph.D., Jack Chen, Ph.D.
hCoV-19/USA/AK-PHL8799/2021	EPI_ISL_2499756	4/23/2021	Alaska State Virology Laboratory	Alaska State Virology Laboratory	Stephanie DeRonde, Elva House, Jacob Zidek, Lisa Smith, Ph.D., Jack Chen, Ph.D.
hCoV-19/USA/AK-PHL8381/2021	EPI_ISL_2499919	4/26/2021	Alaska State Virology Laboratory	Alaska State Virology Laboratory	Stephanie DeRonde, Elva House, Jacob Zidek, Lisa Smith, Ph.D., Jack Chen, Ph.D.
hCoV-19/USA/AK-PHL8741/2021	EPI_ISL_2499920	5/7/2021	Alaska State Virology Laboratory	Alaska State Virology Laboratory	Stephanie DeRonde, Elva House, Jacob Zidek, Lisa Smith, Ph.D., Jack Chen, Ph.D.
hCoV-19/USA/AK-PHL9015/2021	EPI_ISL_2499921	5/20/2021	Alaska State Virology Laboratory	Alaska State Virology Laboratory	Stephanie DeRonde, Elva House, Jacob Zidek, Lisa Smith, Ph.D., Jack Chen, Ph.D.
hCoV-19/USA/AK-PHL9100/2021	EPI_ISL_2499922	5/17/2021	Alaska State Virology Laboratory	Alaska State Virology Laboratory	Stephanie DeRonde, Elva House, Jacob Zidek, Lisa Smith, Ph.D., Jack Chen, Ph.D.
hCoV-19/USA/AK-PHL8979/2021	EPI_ISL_2499925	5/10/2021	Alaska State Virology Laboratory	Alaska State Virology Laboratory	Stephanie DeRonde, Elva House, Jacob Zidek, Lisa Smith, Ph.D., Jack Chen, Ph.D.
hCoV-19/USA/AK-PHL8992/2021	EPI_ISL_2499926	5/14/2021	Alaska State Virology Laboratory	Alaska State Virology Laboratory	Stephanie DeRonde, Elva House, Jacob Zidek, Lisa Smith, Ph.D., Jack Chen, Ph.D.
hCoV-19/USA/AK-PHL8847/2021	EPI_ISL_2499929	5/12/2021	Alaska State Virology Laboratory	Alaska State Virology Laboratory	Stephanie DeRonde, Elva House, Jacob Zidek, Lisa Smith, Ph.D., Jack Chen, Ph.D.
hCoV-19/USA/AK-PHL8114/2021	EPI_ISL_2499930	4/12/2021	Alaska State Virology Laboratory	Alaska State Virology Laboratory	Stephanie DeRonde, Elva House, Jacob Zidek, Lisa Smith, Ph.D., Jack Chen, Ph.D.
hCoV-19/USA/AK-PHL8142/2021	EPI_ISL_2499932	4/12/2021	Alaska State Virology Laboratory	Alaska State Virology Laboratory	Stephanie DeRonde, Elva House, Jacob Zidek, Lisa Smith, Ph.D., Jack Chen, Ph.D.
hCoV-19/USA/AK-PHL8145/2021	EPI_ISL_2499933	4/12/2021	Alaska State Virology Laboratory	Alaska State Virology Laboratory	Stephanie DeRonde, Elva House, Jacob Zidek, Lisa Smith, Ph.D., Jack Chen, Ph.D.
hCoV-19/USA/AK-PHL8216/2021	EPI_ISL_2499934	4/19/2021	Alaska State Virology Laboratory	Alaska State Virology Laboratory	Stephanie DeRonde, Elva House, Lisa Smith, Ph.D., Jack Chen, Ph.D.
hCoV-19/USA/AK-PHL8240/2021	EPI_ISL_2499935	4/19/2021	Alaska State Virology Laboratory	Alaska State Virology Laboratory	Stephanie DeRonde, Elva House, Lisa Smith, Ph.D., Jack Chen, Ph.D.
hCoV-19/USA/AK-PHL8870/2021	EPI_ISL_2499937	5/12/2021	Alaska State Virology Laboratory	Alaska State Virology Laboratory	Stephanie DeRonde, Elva House, Jacob Zidek, Lisa Smith, Ph.D., Jack Chen, Ph.D.
hCoV-19/USA/AK-PHL8881/2021	EPI_ISL_2499938	5/11/2021	Alaska State Virology Laboratory	Alaska State Virology Laboratory	Stephanie DeRonde, Elva House, Jacob Zidek, Lisa Smith, Ph.D., Jack Chen, Ph.D.
hCoV-19/USA/AK-PHL8885/2021	EPI_ISL_2499939	5/11/2021	Alaska State Virology Laboratory	Alaska State Virology Laboratory	Stephanie DeRonde, Elva House, Jacob Zidek, Lisa Smith, Ph.D., Jack Chen, Ph.D.
hCoV-19/USA/AK-PHL8890/2021	EPI_ISL_2499940	5/7/2021	Alaska State Virology Laboratory	Alaska State Virology Laboratory	Stephanie DeRonde, Elva House, Jacob Zidek, Lisa Smith, Ph.D., Jack Chen, Ph.D.
hCoV-19/USA/AK-PHL8907/2021	EPI_ISL_2499941	5/14/2021	Alaska State Virology Laboratory	Alaska State Virology Laboratory	Stephanie DeRonde, Elva House, Jacob Zidek, Lisa Smith, Ph.D., Jack Chen, Ph.D.
hCoV-19/USA/AK-PHL7458/2021	EPI_ISL_2499956	2/1/2021	Alaska State Virology Laboratory	Alaska State Virology Laboratory	Stephanie DeRonde, Elva House, Lisa Smith, Ph.D., Jack Chen, Ph.D.
hCoV-19/USA/AK-PHL8308/2021	EPI_ISL_2499957	4/16/2021	Alaska State Virology Laboratory	Alaska State Virology Laboratory	Stephanie DeRonde, Elva House, Jacob Zidek, Lisa Smith, Ph.D., Jack Chen, Ph.D.
hCoV-19/USA/AK-PHL8331/2021	EPI_ISL_2499958	4/21/2021	Alaska State Virology Laboratory	Alaska State Virology Laboratory	Stephanie DeRonde, Elva House, Jacob Zidek, Lisa Smith, Ph.D., Jack Chen, Ph.D.
hCoV-19/USA/AK-PHL8332/2021	EPI_ISL_2499959	4/19/2021	Alaska State Virology Laboratory	Alaska State Virology Laboratory	Stephanie DeRonde, Elva House, Jacob Zidek, Lisa Smith, Ph.D., Jack Chen, Ph.D.
hCoV-19/USA/AK-PHL7791/2021	EPI_ISL_2502005	3/30/2021	Alaska State Virology Laboratory	Alaska State Virology Laboratory	Stephanie DeRonde, Elva House, Jacob Zidek, Lisa Smith, Ph.D., Jack Chen, Ph.D.
hCoV-19/USA/AK-PHL8052/2021	EPI_ISL_2502006	4/11/2021	Alaska State Virology Laboratory	Alaska State Virology Laboratory	Stephanie DeRonde, Elva House, Jacob Zidek, Lisa Smith, Ph.D., Jack Chen, Ph.D.
hCoV-19/USA/AK-PHL7501/2021	EPI_ISL_2502007	3/6/2021	Alaska State Virology Laboratory	Alaska State Virology Laboratory	Stephanie DeRonde, Elva House, Jacob Zidek, Lisa Smith, Ph.D., Jack Chen, Ph.D.
hCoV-19/USA/AK-PHL7618/2021	EPI_ISL_2502008	3/16/2021	Alaska State Virology Laboratory	Alaska State Virology Laboratory	Stephanie DeRonde, Elva House, Jacob Zidek, Lisa Smith, Ph.D., Jack Chen, Ph.D.
hCoV-19/USA/AK-PHL7950/2021	EPI_ISL_2502009	3/10/2021	Alaska State Virology Laboratory	Alaska State Virology Laboratory	Stephanie DeRonde, Elva House, Jacob Zidek, Lisa Smith, Ph.D., Jack Chen, Ph.D.
hCoV-19/USA/AK-PHL7860/2021	EPI_ISL_2502010	4/1/2021	Alaska State Virology Laboratory	Alaska State Virology Laboratory	Stephanie DeRonde, Elva House, Lisa Smith, Ph.D., Jack Chen, Ph.D.
hCoV-19/USA/AK-PHL8072/2021	EPI_ISL_2502011	4/6/2021	Alaska State Virology Laboratory	Alaska State Virology Laboratory	Stephanie DeRonde, Elva House, Jacob Zidek, Lisa Smith, Ph.D., Jack Chen, Ph.D.
hCoV-19/USA/AK-PHL7801/2021	EPI_ISL_2502012	3/26/2021	Alaska State Virology Laboratory	Alaska State Virology Laboratory	Stephanie DeRonde, Elva House, Jacob Zidek, Lisa Smith, Ph.D., Jack Chen, Ph.D.
hCoV-19/USA/AK-PHL7611/2021	EPI_ISL_2502013	2/1/2021	Alaska State Virology Laboratory	Alaska State Virology Laboratory	Stephanie DeRonde, Elva House, Jacob Zidek, Lisa Smith, Ph.D., Jack Chen, Ph.D.
hCoV-19/USA/AK-PHL7867/2021	EPI_ISL_2502014	3/30/2021	Alaska State Virology Laboratory	Alaska State Virology Laboratory	Stephanie DeRonde, Elva House, Jacob Zidek, Lisa Smith, Ph.D., Jack Chen, Ph.D.
hCoV-19/USA/AK-PHL9281/2021	EPI_ISL_2502579	5/5/2021	Alaska State Virology Lab, Alaska State Public Health Labs	Alaska State Virology Lab, Alaska State Public Health Labs	DeRonde,S., House,E., Zidek,J., Redlinger,M., Smith,L. and Chen,J.
hCoV-19/USA/AK-PHL9341/2021	EPI_ISL_2503977	6/1/2021	Alaska State Virology Laboratory	Alaska State Virology Laboratory	Stephanie DeRonde, Elva House, Jacob Zidek, Lisa Smith, Ph.D., Jack Chen, Ph.D.
hCoV-19/USA/AK-PHL9344/2021	EPI_ISL_2503980	6/1/2021	Alaska State Virology Laboratory	Alaska State Virology Laboratory	Stephanie DeRonde, Elva House, Jacob Zidek, Lisa Smith, Ph.D., Jack Chen, Ph.D.
hCoV-19/USA/AK-PHL9345/2021	EPI_ISL_2503981	6/1/2021	Alaska State Virology Laboratory	Alaska State Virology Laboratory	Stephanie DeRonde, Elva House, Jacob Zidek, Lisa Smith, Ph.D., Jack Chen, Ph.D.
hCoV-19/USA/AK-PHL9349/2021	EPI_ISL_2503983	6/2/2021	Alaska State Virology Laboratory	Alaska State Virology Laboratory	Stephanie DeRonde, Elva House, Jacob Zidek, Lisa Smith, Ph.D., Jack Chen, Ph.D.
hCoV-19/USA/AK-PHL9351/2021	EPI_ISL_2503985	6/2/2021	Alaska State Virology Laboratory	Alaska State Virology Laboratory	Stephanie DeRonde, Elva House, Jacob Zidek, Lisa Smith, Ph.D., Jack Chen, Ph.D.
hCoV-19/USA/AK-PHL9353/2021	EPI_ISL_2503987	6/2/2021	Alaska State Virology Laboratory	Alaska State Virology Laboratory	Stephanie DeRonde, Elva House, Jacob Zidek, Lisa Smith, Ph.D., Jack Chen, Ph.D.
hCoV-19/USA/AK-PHL9355/2021	EPI_ISL_2503989	5/31/2021	Alaska State Virology Laboratory	Alaska State Virology Laboratory	Stephanie DeRonde, Elva House, Jacob Zidek, Lisa Smith, Ph.D., Jack Chen, Ph.D.
hCoV-19/USA/AK-PHL9356/2021	EPI_ISL_2503990	6/1/2021	Alaska State Virology Laboratory	Alaska State Virology Laboratory	Stephanie DeRonde, Elva House, Jacob Zidek, Lisa Smith, Ph.D., Jack Chen, Ph.D.
hCoV-19/USA/AK-PHL9360/2021	EPI_ISL_2503992	6/1/2021	Alaska State Virology Laboratory	Alaska State Virology Laboratory	Stephanie DeRonde, Elva House, Jacob Zidek, Lisa Smith, Ph.D., Jack Chen, Ph.D.
hCoV-19/USA/AK-PHL9362/2021	EPI_ISL_2503993	5/30/2021	Alaska State Virology Laboratory	Alaska State Virology Laboratory	Stephanie DeRonde, Elva House, Jacob Zidek, Lisa Smith, Ph.D., Jack Chen, Ph.D.
hCoV-19/USA/AK-PHL9364/2021	EPI_ISL_2503995	5/30/2021	Alaska State Virology Laboratory	Alaska State Virology Laboratory	Stephanie DeRonde, Elva House, Jacob Zidek, Lisa Smith, Ph.D., Jack Chen, Ph.D.
hCoV-19/USA/AK-PHL9366/2021	EPI_ISL_2503996	5/31/2021	Alaska State Virology Laboratory	Alaska State Virology Laboratory	Stephanie DeRonde, Elva House, Jacob Zidek, Lisa Smith, Ph.D., Jack Chen, Ph.D.
hCoV-19/USA/AK-PHL9367/2021	EPI_ISL_2503997	6/4/2021	Alaska State Virology Laboratory	Alaska State Virology Laboratory	Stephanie DeRonde, Elva House, Jacob Zidek, Lisa Smith, Ph.D., Jack Chen, Ph.D.
hCoV-19/USA/AK-PHL9368/2021	EPI_ISL_2503998	6/2/2021	Alaska State Virology Laboratory	Alaska State Virology Laboratory	Stephanie DeRonde, Elva House, Jacob Zidek, Lisa Smith, Ph.D., Jack Chen, Ph.D.
hCoV-19/USA/AK-PHL9369/2021	EPI_ISL_2503999	6/3/2021	Alaska State Virology Laboratory	Alaska State Virology Laboratory	Stephanie DeRonde, Elva House, Jacob Zidek, Lisa Smith, Ph.D., Jack Chen, Ph.D.

hCoV-19/USA/AK-PHL9370/2021	EPI_ISL_2504000	6/4/2021	Alaska State Virology Laboratory	Alaska State Virology Laboratory	Stephanie DeRonde, Elva House, Jacob Zidek, Lisa Smith, Ph.D., Jack Chen, Ph.D.
hCoV-19/USA/AK-PHL9371/2021	EPI_ISL_2504001	6/4/2021	Alaska State Virology Laboratory	Alaska State Virology Laboratory	Stephanie DeRonde, Elva House, Jacob Zidek, Lisa Smith, Ph.D., Jack Chen, Ph.D.
hCoV-19/USA/AK-PHL9374/2021	EPI_ISL_2504004	6/4/2021	Alaska State Virology Laboratory	Alaska State Virology Laboratory	Stephanie DeRonde, Elva House, Jacob Zidek, Lisa Smith, Ph.D., Jack Chen, Ph.D.
hCoV-19/USA/AK-PHL9377/2021	EPI_ISL_2504006	6/2/2021	Alaska State Virology Laboratory	Alaska State Virology Laboratory	Stephanie DeRonde, Elva House, Jacob Zidek, Lisa Smith, Ph.D., Jack Chen, Ph.D.
hCoV-19/USA/AK-PHL9379/2021	EPI_ISL_2504008	6/4/2021	Alaska State Virology Laboratory	Alaska State Virology Laboratory	Stephanie DeRonde, Elva House, Jacob Zidek, Lisa Smith, Ph.D., Jack Chen, Ph.D.
hCoV-19/USA/AK-PHL9380/2021	EPI_ISL_2504009	5/3/2021	Alaska State Virology Laboratory	Alaska State Virology Laboratory	Stephanie DeRonde, Elva House, Jacob Zidek, Lisa Smith, Ph.D., Jack Chen, Ph.D.
hCoV-19/USA/AK-PHL9381/2021	EPI_ISL_2504010	4/21/2021	Alaska State Virology Laboratory	Alaska State Virology Laboratory	Stephanie DeRonde, Elva House, Jacob Zidek, Lisa Smith, Ph.D., Jack Chen, Ph.D.
hCoV-19/USA/AK-PHL9382/2021	EPI_ISL_2504011	5/2/2021	Alaska State Virology Laboratory	Alaska State Virology Laboratory	Stephanie DeRonde, Elva House, Jacob Zidek, Lisa Smith, Ph.D., Jack Chen, Ph.D.
hCoV-19/USA/AK-PHL9383/2021	EPI_ISL_2504012	5/4/2021	Alaska State Virology Laboratory	Alaska State Virology Laboratory	Stephanie DeRonde, Elva House, Jacob Zidek, Lisa Smith, Ph.D., Jack Chen, Ph.D.
hCoV-19/USA/AK-PHL7974/2021	EPI_ISL_2502004	4/7/2021	Alaska State Virology Laboratory	Alaska State Virology Laboratory	Stephanie DeRonde, Elva House, Jacob Zidek, Lisa Smith, Ph.D., Jack Chen, Ph.D.
hCoV-19/USA/AK-CDC-ASC210106954/2021	EPI_ISL_2528030	6/2/2021	Aegis Sciences Corporation	Centers for Disease Control and Prevention Division of Viral Diseases, Pathogen Discovery	Dakota Howard, Dhwani Batra, Peter W. Cook, Kara Moser, Adrian Paskey, Jason Caravas, Benjamin Rambo-Martin, Shatavia Morrison, Christopher Gulvick, Scott Sammons, Yvette Unoarumhi, Darlene Wagner, Matthew Schmerer, Cyndi Clark, Patrick Campbell, Rob Case, Vikramsinha Ghorpade, Holly Houdeshell, Ola Kvalvaag, Dillon Nall, Ethan Sanders, Alec Vest, Shaun Westlund, Matthew Hardison, Clinton R. Paden, Duncan MacCannell
hCoV-19/USA/AK-PHL8358/2021	EPI_ISL_1920970	4/23/2021	Alaska State Virology Laboratory	Alaska State Virology Laboratory	Stephanie DeRonde, Elva House, Jacob Zidek, Lisa Smith, Ph.D., Jack Chen, Ph.D.
hCoV-19/USA/AK-PHL8770/2021	EPI_ISL_2154083	5/10/2021	Alaska State Virology Laboratory	Alaska State Virology Laboratory	Stephanie DeRonde, Elva House, Jacob Zidek, Lisa Smith, Ph.D., Jack Chen, Ph.D.
hCoV-19/USA/AK-CDC-ASC210105751/2021	EPI_ISL_2531059	5/26/2021	Aegis Sciences Corporation	Centers for Disease Control and Prevention Division of Viral Diseases, Pathogen Discovery	Dakota Howard, Dhwani Batra, Peter W. Cook, Kara Moser, Adrian Paskey, Jason Caravas, Benjamin Rambo-Martin, Shatavia Morrison, Christopher Gulvick, Scott Sammons, Yvette Unoarumhi, Darlene Wagner, Matthew Schmerer, Cyndi Clark, Patrick Campbell, Rob Case, Vikramsinha Ghorpade, Holly Houdeshell, Ola Kvalvaag, Dillon Nall, Ethan Sanders, Alec Vest, Shaun Westlund, Matthew Hardison, Clinton R. Paden, Duncan MacCannell
hCoV-19/USA/AK-PHL8144/2021	EPI_ISL_1893894	4/12/2021	Alaska State Virology Laboratory	Alaska State Virology Laboratory	Stephanie DeRonde, Elva House, Jacob Zidek, Lisa Smith, Ph.D., Jack Chen, Ph.D.
hCoV-19/USA/AK-PHL8146/2021	EPI_ISL_1893897	4/12/2021	Alaska State Virology Laboratory	Alaska State Virology Laboratory	Stephanie DeRonde, Elva House, Jacob Zidek, Lisa Smith, Ph.D., Jack Chen, Ph.D.
hCoV-19/USA/AK-PHL8147/2021	EPI_ISL_1893904	4/12/2021	Alaska State Virology Laboratory	Alaska State Virology Laboratory	Stephanie DeRonde, Elva House, Jacob Zidek, Lisa Smith, Ph.D., Jack Chen, Ph.D.
hCoV-19/USA/AK-PHL8148/2021	EPI_ISL_1893906	4/12/2021	Alaska State Virology Laboratory	Alaska State Virology Laboratory	Stephanie DeRonde, Elva House, Jacob Zidek, Lisa Smith, Ph.D., Jack Chen, Ph.D.
hCoV-19/USA/AK-PHL8149/2021	EPI_ISL_1893908	4/16/2021	Alaska State Virology Laboratory	Alaska State Virology Laboratory	Stephanie DeRonde, Elva House, Jacob Zidek, Lisa Smith, Ph.D., Jack Chen, Ph.D.
hCoV-19/USA/AK-PHL8150/2021	EPI_ISL_1893910	4/17/2021	Alaska State Virology Laboratory	Alaska State Virology Laboratory	Stephanie DeRonde, Elva House, Jacob Zidek, Lisa Smith, Ph.D., Jack Chen, Ph.D.
hCoV-19/USA/AK-PHL8305/2021	EPI_ISL_1895852	4/21/2021	Alaska State Virology Laboratory	Alaska State Virology Laboratory	Stephanie DeRonde, Elva House, Jacob Zidek, Lisa Smith, Ph.D., Jack Chen, Ph.D.
hCoV-19/USA/AK-PHL8306/2021	EPI_ISL_1895857	4/22/2021	Alaska State Virology Laboratory	Alaska State Virology Laboratory	Stephanie DeRonde, Elva House, Jacob Zidek, Lisa Smith, Ph.D., Jack Chen, Ph.D.
hCoV-19/USA/AK-PHL8309/2021	EPI_ISL_1895868	4/17/2021	Alaska State Virology Laboratory	Alaska State Virology Laboratory	Stephanie DeRonde, Elva House, Jacob Zidek, Lisa Smith, Ph.D., Jack Chen, Ph.D.
hCoV-19/USA/AK-PHL8311/2021	EPI_ISL_1895878	4/20/2021	Alaska State Virology Laboratory	Alaska State Virology Laboratory	Stephanie DeRonde, Elva House, Jacob Zidek, Lisa Smith, Ph.D., Jack Chen, Ph.D.
hCoV-19/USA/AK-PHL8313/2021	EPI_ISL_1895889	4/18/2021	Alaska State Virology Laboratory	Alaska State Virology Laboratory	Stephanie DeRonde, Elva House, Jacob Zidek, Lisa Smith, Ph.D., Jack Chen, Ph.D.
hCoV-19/USA/AK-PHL8314/2021	EPI_ISL_1895893	4/20/2021	Alaska State Virology Laboratory	Alaska State Virology Laboratory	Stephanie DeRonde, Elva House, Jacob Zidek, Lisa Smith, Ph.D., Jack Chen, Ph.D.
hCoV-19/USA/AK-PHL8319/2021	EPI_ISL_1895915	4/23/2021	Alaska State Virology Laboratory	Alaska State Virology Laboratory	Stephanie DeRonde, Elva House, Jacob Zidek, Lisa Smith, Ph.D., Jack Chen, Ph.D.
hCoV-19/USA/AK-PHL8320/2021	EPI_ISL_1895921	4/25/2021	Alaska State Virology Laboratory	Alaska State Virology Laboratory	Stephanie DeRonde, Elva House, Jacob Zidek, Lisa Smith, Ph.D., Jack Chen, Ph.D.
hCoV-19/USA/AK-PHL8321/2021	EPI_ISL_1895926	4/25/2021	Alaska State Virology Laboratory	Alaska State Virology Laboratory	Stephanie DeRonde, Elva House, Jacob Zidek, Lisa Smith, Ph.D., Jack Chen, Ph.D.
hCoV-19/USA/AK-PHL8324/2021	EPI_ISL_1895938	4/24/2021	Alaska State Virology Laboratory	Alaska State Virology Laboratory	Stephanie DeRonde, Elva House, Jacob Zidek, Lisa Smith, Ph.D., Jack Chen, Ph.D.
hCoV-19/USA/AK-PHL8326/2021	EPI_ISL_1895949	4/25/2021	Alaska State Virology Laboratory	Alaska State Virology Laboratory	Stephanie DeRonde, Elva House, Jacob Zidek, Lisa Smith, Ph.D., Jack Chen, Ph.D.
hCoV-19/USA/AK-PHL8327/2021	EPI_ISL_1895954	4/25/2021	Alaska State Virology Laboratory	Alaska State Virology Laboratory	Stephanie DeRonde, Elva House, Jacob Zidek, Lisa Smith, Ph.D., Jack Chen, Ph.D.
hCoV-19/USA/AK-PHL8329/2021	EPI_ISL_1895964	4/25/2021	Alaska State Virology Laboratory	Alaska State Virology Laboratory	Stephanie DeRonde, Elva House, Jacob Zidek, Lisa Smith, Ph.D., Jack Chen, Ph.D.
hCoV-19/USA/AK-PHL8333/2021	EPI_ISL_1895969	4/19/2021	Alaska State Virology Laboratory	Alaska State Virology Laboratory	Stephanie DeRonde, Elva House, Jacob Zidek, Lisa Smith, Ph.D., Jack Chen, Ph.D.
hCoV-19/USA/AK-PHL8335/2021	EPI_ISL_1895980	4/14/2021	Alaska State Virology Laboratory	Alaska State Virology Laboratory	Stephanie DeRonde, Elva House, Jacob Zidek, Lisa Smith, Ph.D., Jack Chen, Ph.D.
hCoV-19/USA/AK-PHL8336/2021	EPI_ISL_1895984	4/17/2021	Alaska State Virology Laboratory	Alaska State Virology Laboratory	Stephanie DeRonde, Elva House, Jacob Zidek, Lisa Smith, Ph.D., Jack Chen, Ph.D.
hCoV-19/USA/AK-PHL8338/2021	EPI_ISL_1895995	4/22/2021	Alaska State Virology Laboratory	Alaska State Virology Laboratory	Stephanie DeRonde, Elva House, Jacob Zidek, Lisa Smith, Ph.D., Jack Chen, Ph.D.
hCoV-19/USA/AK-PHL8339/2021	EPI_ISL_1896000	4/20/2021	Alaska State Virology Laboratory	Alaska State Virology Laboratory	Stephanie DeRonde, Elva House, Jacob Zidek, Lisa Smith, Ph.D., Jack Chen, Ph.D.
hCoV-19/USA/AK-PHL8340/2021	EPI_ISL_1896005	4/19/2021	Alaska State Virology Laboratory	Alaska State Virology Laboratory	Stephanie DeRonde, Elva House, Jacob Zidek, Lisa Smith, Ph.D., Jack Chen, Ph.D.
hCoV-19/USA/AK-PHL8342/2021	EPI_ISL_1896015	4/19/2021	Alaska State Virology Laboratory	Alaska State Virology Laboratory	Stephanie DeRonde, Elva House, Jacob Zidek, Lisa Smith, Ph.D., Jack Chen, Ph.D.
hCoV-19/USA/AK-PHL8345/2021	EPI_ISL_1896025	4/21/2021	Alaska State Virology Laboratory	Alaska State Virology Laboratory	Stephanie DeRonde, Elva House, Jacob Zidek, Lisa Smith, Ph.D., Jack Chen, Ph.D.
hCoV-19/USA/AK-PHL8347/2021	EPI_ISL_1896035	4/22/2021	Alaska State Virology Laboratory	Alaska State Virology Laboratory	Stephanie DeRonde, Elva House, Jacob Zidek, Lisa Smith, Ph.D., Jack Chen, Ph.D.

hCoV-19/USA/AK-PHL8349/2021	EPI_ISL_1896041	4/22/2021	Alaska State Virology Laboratory	Alaska State Virology Laboratory	Stephanie DeRonde, Elva House, Jacob Zidek, Lisa Smith, Ph.D., Jack Chen, Ph.D.
hCoV-19/USA/AK-PHL6544/2020	EPI_ISL_2557913	11/30/2020	Alaska State Virology Laboratory	Alaska State Virology Laboratory	Stephanie DeRonde, Elva House, Jacob Zidek, Lisa Smith, Ph.D., Jack Chen, Ph.D.
hCoV-19/USA/AK-CDC-2-4504187/2021	EPI_ISL_2689868	5/18/2021	AK State Public Health Lab, State Health Department	Centers for Disease Control and Prevention Division of Viral Diseases, Pathogen Discovery	Mili Sheth, Sarah Nobles, Jasmine Padilla, Mark Burroughs, Shoshona Le, Katie Dillon, Peter Cook, Clinton R. Paden, Dhwani Batra, Krista Queen, Kristen Knipe, Dakota Howard, Yvette Unoarumhi, Darlene Wagner, Matthew Schmerer, Ben L. Rambo-Martin, Kristine Lacek, Sam Shepard, Alison Laufer Halpin, Dave Wentworth, Vivien Dugan, Suxiang Tong, Justin Lee
hCoV-19/USA/AK-CDC-2-4504158/2021	EPI_ISL_2689869	5/16/2021	AK State Public Health Lab, State Health Department	Centers for Disease Control and Prevention Division of Viral Diseases, Pathogen Discovery	Mili Sheth, Sarah Nobles, Jasmine Padilla, Mark Burroughs, Shoshona Le, Katie Dillon, Peter Cook, Clinton R. Paden, Dhwani Batra, Krista Queen, Kristen Knipe, Dakota Howard, Yvette Unoarumhi, Darlene Wagner, Matthew Schmerer, Ben L. Rambo-Martin, Kristine Lacek, Sam Shepard, Alison Laufer Halpin, Dave Wentworth, Vivien Dugan, Suxiang Tong, Justin Lee
hCoV-19/USA/AK-CDC-2-4504091/2021	EPI_ISL_2689872	5/14/2021	AK State Public Health Lab, State Health Department	Centers for Disease Control and Prevention Division of Viral Diseases, Pathogen Discovery	Mili Sheth, Sarah Nobles, Jasmine Padilla, Mark Burroughs, Shoshona Le, Katie Dillon, Peter Cook, Clinton R. Paden, Dhwani Batra, Krista Queen, Kristen Knipe, Dakota Howard, Yvette Unoarumhi, Darlene Wagner, Matthew Schmerer, Ben L. Rambo-Martin, Kristine Lacek, Sam Shepard, Alison Laufer Halpin, Dave Wentworth, Vivien Dugan, Suxiang Tong, Justin Lee
hCoV-19/USA/AK-CDC-2-4504062/2021	EPI_ISL_2689873	5/16/2021	AK State Public Health Lab, State Health Department	Centers for Disease Control and Prevention Division of Viral Diseases, Pathogen Discovery	Mili Sheth, Sarah Nobles, Jasmine Padilla, Mark Burroughs, Shoshona Le, Katie Dillon, Peter Cook, Clinton R. Paden, Dhwani Batra, Krista Queen, Kristen Knipe, Dakota Howard, Yvette Unoarumhi, Darlene Wagner, Matthew Schmerer, Ben L. Rambo-Martin, Kristine Lacek, Sam Shepard, Alison Laufer Halpin, Dave Wentworth, Vivien Dugan, Suxiang Tong, Justin Lee
hCoV-19/USA/AK-PHL6677/2021	EPI_ISL_2716840	1/5/2021	Alaska State Virology Laboratory	Alaska State Virology Laboratory	Stephanie DeRonde, Elva House, Jacob Zidek, Lisa Smith, Ph.D., Jack Chen, Ph.D.
hCoV-19/USA/AK-PHL6678/2021	EPI_ISL_2716841	1/7/2021	Alaska State Virology Laboratory	Alaska State Virology Laboratory	Stephanie DeRonde, Elva House, Jacob Zidek, Lisa Smith, Ph.D., Jack Chen, Ph.D.
hCoV-19/USA/AK-PHL6679/2021	EPI_ISL_2716842	1/8/2021	Alaska State Virology Laboratory	Alaska State Virology Laboratory	Stephanie DeRonde, Elva House, Jacob Zidek, Lisa Smith, Ph.D., Jack Chen, Ph.D.
hCoV-19/USA/AK-PHL6683/2021	EPI_ISL_2716843	1/8/2021	Alaska State Virology Laboratory	Alaska State Virology Laboratory	Stephanie DeRonde, Elva House, Jacob Zidek, Lisa Smith, Ph.D., Jack Chen, Ph.D.
hCoV-19/USA/AK-PHL8307/2021	EPI_ISL_1895862	4/16/2021	Alaska State Virology Laboratory	Alaska State Virology Laboratory	Stephanie DeRonde, Elva House, Jacob Zidek, Lisa Smith, Ph.D., Jack Chen, Ph.D.
hCoV-19/USA/AK-PHL8310/2021	EPI_ISL_1895873	4/20/2021	Alaska State Virology Laboratory	Alaska State Virology Laboratory	Stephanie DeRonde, Elva House, Jacob Zidek, Lisa Smith, Ph.D., Jack Chen, Ph.D.
hCoV-19/USA/AK-PHL8312/2021	EPI_ISL_1895884	4/22/2021	Alaska State Virology Laboratory	Alaska State Virology Laboratory	Stephanie DeRonde, Elva House, Jacob Zidek, Lisa Smith, Ph.D., Jack Chen, Ph.D.
hCoV-19/USA/AK-PHL8315/2021	EPI_ISL_1895899	4/20/2021	Alaska State Virology Laboratory	Alaska State Virology Laboratory	Stephanie DeRonde, Elva House, Jacob Zidek, Lisa Smith, Ph.D., Jack Chen, Ph.D.
hCoV-19/USA/AK-PHL8316/2021	EPI_ISL_1895904	4/20/2021	Alaska State Virology Laboratory	Alaska State Virology Laboratory	Stephanie DeRonde, Elva House, Jacob Zidek, Lisa Smith, Ph.D., Jack Chen, Ph.D.
hCoV-19/USA/AK-PHL8317/2021	EPI_ISL_1895909	4/20/2021	Alaska State Virology Laboratory	Alaska State Virology Laboratory	Stephanie DeRonde, Elva House, Jacob Zidek, Lisa Smith, Ph.D., Jack Chen, Ph.D.
hCoV-19/USA/AK-PHL8323/2021	EPI_ISL_1895932	4/24/2021	Alaska State Virology Laboratory	Alaska State Virology Laboratory	Stephanie DeRonde, Elva House, Jacob Zidek, Lisa Smith, Ph.D., Jack Chen, Ph.D.
hCoV-19/USA/AK-PHL8325/2021	EPI_ISL_1895943	4/25/2021	Alaska State Virology Laboratory	Alaska State Virology Laboratory	Stephanie DeRonde, Elva House, Jacob Zidek, Lisa Smith, Ph.D., Jack Chen, Ph.D.
hCoV-19/USA/AK-PHL8328/2021	EPI_ISL_1895959	4/25/2021	Alaska State Virology Laboratory	Alaska State Virology Laboratory	Stephanie DeRonde, Elva House, Jacob Zidek, Lisa Smith, Ph.D., Jack Chen, Ph.D.
hCoV-19/USA/AK-PHL8334/2021	EPI_ISL_1895974	4/18/2021	Alaska State Virology Laboratory	Alaska State Virology Laboratory	Stephanie DeRonde, Elva House, Jacob Zidek, Lisa Smith, Ph.D., Jack Chen, Ph.D.
hCoV-19/USA/AK-PHL8337/2021	EPI_ISL_1895990	4/19/2021	Alaska State Virology Laboratory	Alaska State Virology Laboratory	Stephanie DeRonde, Elva House, Jacob Zidek, Lisa Smith, Ph.D., Jack Chen, Ph.D.
hCoV-19/USA/AK-PHL8341/2021	EPI_ISL_1896010	4/19/2021	Alaska State Virology Laboratory	Alaska State Virology Laboratory	Stephanie DeRonde, Elva House, Jacob Zidek, Lisa Smith, Ph.D., Jack Chen, Ph.D.
hCoV-19/USA/AK-PHL8344/2021	EPI_ISL_1896020	4/20/2021	Alaska State Virology Laboratory	Alaska State Virology Laboratory	Stephanie DeRonde, Elva House, Jacob Zidek, Lisa Smith, Ph.D., Jack Chen, Ph.D.
hCoV-19/USA/AK-PHL8346/2021	EPI_ISL_1896030	4/21/2021	Alaska State Virology Laboratory	Alaska State Virology Laboratory	Stephanie DeRonde, Elva House, Jacob Zidek, Lisa Smith, Ph.D., Jack Chen, Ph.D.
hCoV-19/USA/AK-CDC-ASC210053159/2021	EPI_ISL_1900421	4/7/2021	Aegis Sciences Corporation	Centers for Disease Control and Prevention Division of Viral Diseases, Pathogen Discovery	Dakota Howard, Dhwani Batra, Peter W. Cook, Kara Moser, Adrian Paskey, Jason Caravas, Benjamin Rambo-Martin, Shatavia Morrison, Christopher Gulvick, Scott Sammons, Yvette Unoarumhi, Darlene Wagner, Matthew Schmerer, Cyndi Clark, Patrick Campbell, Rob Case, Vikramsinha Ghorpade, Holly Houdeshell, Ola Kvalvaag, Dillon Nall, Ethan Sanders, Alec Vest, Shaun Westlund, Matthew Hardison, Clinton R. Paden, Duncan MacCannell
hCoV-19/USA/AK-PHL8350/2021	EPI_ISL_1920963	4/19/2021	Alaska State Virology Laboratory	Alaska State Virology Laboratory	Stephanie DeRonde, Elva House, Jacob Zidek, Lisa Smith, Ph.D., Jack Chen, Ph.D.
hCoV-19/USA/AK-PHL8351/2021	EPI_ISL_1920964	4/23/2021	Alaska State Virology Laboratory	Alaska State Virology Laboratory	Stephanie DeRonde, Elva House, Jacob Zidek, Lisa Smith, Ph.D., Jack Chen, Ph.D.
hCoV-19/USA/AK-PHL8352/2021	EPI_ISL_1920965	4/23/2021	Alaska State Virology Laboratory	Alaska State Virology Laboratory	Stephanie DeRonde, Elva House, Jacob Zidek, Lisa Smith, Ph.D., Jack Chen, Ph.D.
hCoV-19/USA/AK-PHL8354/2021	EPI_ISL_1920966	4/23/2021	Alaska State Virology Laboratory	Alaska State Virology Laboratory	Stephanie DeRonde, Elva House, Jacob Zidek, Lisa Smith, Ph.D., Jack Chen, Ph.D.
hCoV-19/USA/AK-PHL8355/2021	EPI_ISL_1920967	4/19/2021	Alaska State Virology Laboratory	Alaska State Virology Laboratory	Stephanie DeRonde, Elva House, Jacob Zidek, Lisa Smith, Ph.D., Jack Chen, Ph.D.
hCoV-19/USA/AK-PHL8356/2021	EPI_ISL_1920968	4/19/2021	Alaska State Virology Laboratory	Alaska State Virology Laboratory	Stephanie DeRonde, Elva House, Jacob Zidek, Lisa Smith, Ph.D., Jack Chen, Ph.D.
hCoV-19/USA/AK-PHL8357/2021	EPI_ISL_1920969	4/24/2021	Alaska State Virology Laboratory	Alaska State Virology Laboratory	Stephanie DeRonde, Elva House, Jacob Zidek, Lisa Smith, Ph.D., Jack Chen, Ph.D.

hCoV-19/USA/AK-PHL8359/2021	EPI_ISL_1920971	4/23/2021	Alaska State Virology Laboratory	Alaska State Virology Laboratory	Stephanie DeRonde, Elva House, Jacob Zidek, Lisa Smith, Ph.D., Jack Chen, Ph.D.
hCoV-19/USA/AK-PHL8360/2021	EPI_ISL_1920972	4/23/2021	Alaska State Virology Laboratory	Alaska State Virology Laboratory	Stephanie DeRonde, Elva House, Jacob Zidek, Lisa Smith, Ph.D., Jack Chen, Ph.D.
hCoV-19/USA/AK-PHL8361/2021	EPI_ISL_1920973	4/23/2021	Alaska State Virology Laboratory	Alaska State Virology Laboratory	Stephanie DeRonde, Elva House, Jacob Zidek, Lisa Smith, Ph.D., Jack Chen, Ph.D.
hCoV-19/USA/AK-PHL8362/2021	EPI_ISL_1920974	4/23/2021	Alaska State Virology Laboratory	Alaska State Virology Laboratory	Stephanie DeRonde, Elva House, Jacob Zidek, Lisa Smith, Ph.D., Jack Chen, Ph.D.
hCoV-19/USA/AK-PHL8363/2021	EPI_ISL_1920975	4/23/2021	Alaska State Virology Laboratory	Alaska State Virology Laboratory	Stephanie DeRonde, Elva House, Jacob Zidek, Lisa Smith, Ph.D., Jack Chen, Ph.D.
hCoV-19/USA/AK-PHL8364/2021	EPI_ISL_1920976	4/22/2021	Alaska State Virology Laboratory	Alaska State Virology Laboratory	Stephanie DeRonde, Elva House, Jacob Zidek, Lisa Smith, Ph.D., Jack Chen, Ph.D.
hCoV-19/USA/AK-PHL8366/2021	EPI_ISL_1920977	4/22/2021	Alaska State Virology Laboratory	Alaska State Virology Laboratory	Stephanie DeRonde, Elva House, Jacob Zidek, Lisa Smith, Ph.D., Jack Chen, Ph.D.
hCoV-19/USA/AK-PHL8367/2021	EPI_ISL_1920978	4/22/2021	Alaska State Virology Laboratory	Alaska State Virology Laboratory	Stephanie DeRonde, Elva House, Jacob Zidek, Lisa Smith, Ph.D., Jack Chen, Ph.D.
hCoV-19/USA/AK-PHL8369/2021	EPI_ISL_1920979	4/22/2021	Alaska State Virology Laboratory	Alaska State Virology Laboratory	Stephanie DeRonde, Elva House, Jacob Zidek, Lisa Smith, Ph.D., Jack Chen, Ph.D.
hCoV-19/USA/AK-PHL8370/2021	EPI_ISL_1920980	4/22/2021	Alaska State Virology Laboratory	Alaska State Virology Laboratory	Stephanie DeRonde, Elva House, Jacob Zidek, Lisa Smith, Ph.D., Jack Chen, Ph.D.
hCoV-19/USA/AK-PHL8371/2021	EPI_ISL_1920981	4/22/2021	Alaska State Virology Laboratory	Alaska State Virology Laboratory	Stephanie DeRonde, Elva House, Jacob Zidek, Lisa Smith, Ph.D., Jack Chen, Ph.D.
hCoV-19/USA/AK-PHL8372/2021	EPI_ISL_1920982	4/22/2021	Alaska State Virology Laboratory	Alaska State Virology Laboratory	Stephanie DeRonde, Elva House, Jacob Zidek, Lisa Smith, Ph.D., Jack Chen, Ph.D.
hCoV-19/USA/AK-PHL8373/2021	EPI_ISL_1920983	4/22/2021	Alaska State Virology Laboratory	Alaska State Virology Laboratory	Stephanie DeRonde, Elva House, Jacob Zidek, Lisa Smith, Ph.D., Jack Chen, Ph.D.
hCoV-19/USA/AK-PHL8374/2021	EPI_ISL_1920984	4/22/2021	Alaska State Virology Laboratory	Alaska State Virology Laboratory	Stephanie DeRonde, Elva House, Jacob Zidek, Lisa Smith, Ph.D., Jack Chen, Ph.D.
hCoV-19/USA/AK-PHL8375/2021	EPI_ISL_1920985	4/22/2021	Alaska State Virology Laboratory	Alaska State Virology Laboratory	Stephanie DeRonde, Elva House, Jacob Zidek, Lisa Smith, Ph.D., Jack Chen, Ph.D.
hCoV-19/USA/AK-PHL8376/2021	EPI_ISL_1920986	4/24/2021	Alaska State Virology Laboratory	Alaska State Virology Laboratory	Stephanie DeRonde, Elva House, Jacob Zidek, Lisa Smith, Ph.D., Jack Chen, Ph.D.
hCoV-19/USA/AK-PHL8378/2021	EPI_ISL_1920987	4/24/2021	Alaska State Virology Laboratory	Alaska State Virology Laboratory	Stephanie DeRonde, Elva House, Jacob Zidek, Lisa Smith, Ph.D., Jack Chen, Ph.D.
hCoV-19/USA/AK-PHL8379/2021	EPI_ISL_1920988	4/24/2021	Alaska State Virology Laboratory	Alaska State Virology Laboratory	Stephanie DeRonde, Elva House, Jacob Zidek, Lisa Smith, Ph.D., Jack Chen, Ph.D.
hCoV-19/USA/AK-PHL8380/2021	EPI_ISL_1920989	4/24/2021	Alaska State Virology Laboratory	Alaska State Virology Laboratory	Stephanie DeRonde, Elva House, Jacob Zidek, Lisa Smith, Ph.D., Jack Chen, Ph.D.
hCoV-19/USA/AK-PHL8382/2021	EPI_ISL_1920990	4/23/2021	Alaska State Virology Laboratory	Alaska State Virology Laboratory	Stephanie DeRonde, Elva House, Jacob Zidek, Lisa Smith, Ph.D., Jack Chen, Ph.D.
hCoV-19/USA/AK-PHL8383/2021	EPI_ISL_1920991	4/25/2021	Alaska State Virology Laboratory	Alaska State Virology Laboratory	Stephanie DeRonde, Elva House, Jacob Zidek, Lisa Smith, Ph.D., Jack Chen, Ph.D.
hCoV-19/USA/AK-PHL8384/2021	EPI_ISL_1920992	4/23/2021	Alaska State Virology Laboratory	Alaska State Virology Laboratory	Stephanie DeRonde, Elva House, Jacob Zidek, Lisa Smith, Ph.D., Jack Chen, Ph.D.
hCoV-19/USA/AK-PHL8386/2021	EPI_ISL_1920993	4/25/2021	Alaska State Virology Laboratory	Alaska State Virology Laboratory	Stephanie DeRonde, Elva House, Jacob Zidek, Lisa Smith, Ph.D., Jack Chen, Ph.D.
hCoV-19/USA/AK-PHL8388/2021	EPI_ISL_1920994	4/22/2021	Alaska State Virology Laboratory	Alaska State Virology Laboratory	Stephanie DeRonde, Elva House, Jacob Zidek, Lisa Smith, Ph.D., Jack Chen, Ph.D.
hCoV-19/USA/AK-PHL8390/2021	EPI_ISL_1920995	4/22/2021	Alaska State Virology Laboratory	Alaska State Virology Laboratory	Stephanie DeRonde, Elva House, Jacob Zidek, Lisa Smith, Ph.D., Jack Chen, Ph.D.
hCoV-19/USA/AK-PHL8391/2021	EPI_ISL_1920996	4/23/2021	Alaska State Virology Laboratory	Alaska State Virology Laboratory	Stephanie DeRonde, Elva House, Jacob Zidek, Lisa Smith, Ph.D., Jack Chen, Ph.D.
hCoV-19/USA/AK-CDC-ASC210069283/2021	EPI_ISL_1922915	4/12/2021	Aegis Sciences Corporation	Centers for Disease Control and Prevention Division of Viral Diseases, Pathogen Discovery	Dakota Howard, Dhwani Batra, Peter W. Cook, Kara Moser, Adrian Paskey, Jason Caravas, Benjamin Rambo-Martin, Shatavia Morrison, Christopher Gulvick, Scott Sammons, Yvette Unoarumhi, Darlene Wagner, Matthew Schmerer, Cyndi Clark, Patrick Campbell, Rob Case, Vikramsinha Ghorpade, Holly Houdeshell, Ola Kvalvaag, Dillon Nall, Ethan Sanders, Alec Vest, Shaun Westlund, Matthew Hardison, Clinton R. Paden, Duncan MacCannell
hCoV-19/USA/AK-PHL8396/2021	EPI_ISL_1967903	4/12/2021	Alaska State Virology Laboratory	Alaska State Virology Laboratory	Stephanie DeRonde, Elva House, Jacob Zidek, Lisa Smith, Ph.D., Jack Chen, Ph.D.
hCoV-19/USA/AK-PHL8397/2021	EPI_ISL_1967904	4/16/2021	Alaska State Virology Laboratory	Alaska State Virology Laboratory	Stephanie DeRonde, Elva House, Jacob Zidek, Lisa Smith, Ph.D., Jack Chen, Ph.D.
hCoV-19/USA/AK-PHL8398/2021	EPI_ISL_1967905	4/20/2021	Alaska State Virology Laboratory	Alaska State Virology Laboratory	Stephanie DeRonde, Elva House, Jacob Zidek, Lisa Smith, Ph.D., Jack Chen, Ph.D.
hCoV-19/USA/AK-PHL8399/2021	EPI_ISL_1967906	4/25/2021	Alaska State Virology Laboratory	Alaska State Virology Laboratory	Stephanie DeRonde, Elva House, Jacob Zidek, Lisa Smith, Ph.D., Jack Chen, Ph.D.
hCoV-19/USA/AK-PHL8400/2021	EPI_ISL_1967907	4/24/2021	Alaska State Virology Laboratory	Alaska State Virology Laboratory	Stephanie DeRonde, Elva House, Jacob Zidek, Lisa Smith, Ph.D., Jack Chen, Ph.D.
hCoV-19/USA/AK-PHL8401/2021	EPI_ISL_1967908	4/24/2021	Alaska State Virology Laboratory	Alaska State Virology Laboratory	Stephanie DeRonde, Elva House, Jacob Zidek, Lisa Smith, Ph.D., Jack Chen, Ph.D.
hCoV-19/USA/AK-PHL8402/2021	EPI_ISL_1967909	4/25/2021	Alaska State Virology Laboratory	Alaska State Virology Laboratory	Stephanie DeRonde, Elva House, Jacob Zidek, Lisa Smith, Ph.D., Jack Chen, Ph.D.
hCoV-19/USA/AK-PHL8404/2021	EPI_ISL_1967910	4/25/2021	Alaska State Virology Laboratory	Alaska State Virology Laboratory	Stephanie DeRonde, Elva House, Jacob Zidek, Lisa Smith, Ph.D., Jack Chen, Ph.D.
hCoV-19/USA/AK-PHL8407/2021	EPI_ISL_1967911	4/24/2021	Alaska State Virology Laboratory	Alaska State Virology Laboratory	Stephanie DeRonde, Elva House, Jacob Zidek, Lisa Smith, Ph.D., Jack Chen, Ph.D.
hCoV-19/USA/AK-PHL8408/2021	EPI_ISL_1967912	4/25/2021	Alaska State Virology Laboratory	Alaska State Virology Laboratory	Stephanie DeRonde, Elva House, Jacob Zidek, Lisa Smith, Ph.D., Jack Chen, Ph.D.
hCoV-19/USA/AK-PHL8409/2021	EPI_ISL_1967913	4/26/2021	Alaska State Virology Laboratory	Alaska State Virology Laboratory	Stephanie DeRonde, Elva House, Jacob Zidek, Lisa Smith, Ph.D., Jack Chen, Ph.D.
hCoV-19/USA/AK-PHL8410/2021	EPI_ISL_1967914	4/26/2021	Alaska State Virology Laboratory	Alaska State Virology Laboratory	Stephanie DeRonde, Elva House, Jacob Zidek, Lisa Smith, Ph.D., Jack Chen, Ph.D.
hCoV-19/USA/AK-PHL8411/2021	EPI_ISL_1967915	4/26/2021	Alaska State Virology Laboratory	Alaska State Virology Laboratory	Stephanie DeRonde, Elva House, Jacob Zidek, Lisa Smith, Ph.D., Jack Chen, Ph.D.
hCoV-19/USA/AK-PHL8413/2021	EPI_ISL_1967916	4/26/2021	Alaska State Virology Laboratory	Alaska State Virology Laboratory	Stephanie DeRonde, Elva House, Jacob Zidek, Lisa Smith, Ph.D., Jack Chen, Ph.D.
hCoV-19/USA/AK-PHL8414/2021	EPI_ISL_1967917	4/26/2021	Alaska State Virology Laboratory	Alaska State Virology Laboratory	Stephanie DeRonde, Elva House, Jacob Zidek, Lisa Smith, Ph.D., Jack Chen, Ph.D.
hCoV-19/USA/AK-PHL8415/2021	EPI_ISL_1967918	4/26/2021	Alaska State Virology Laboratory	Alaska State Virology Laboratory	Stephanie DeRonde, Elva House, Jacob Zidek, Lisa Smith, Ph.D., Jack Chen, Ph.D.
hCoV-19/USA/AK-PHL8416/2021	EPI_ISL_1967919	4/24/2021	Alaska State Virology Laboratory	Alaska State Virology Laboratory	Stephanie DeRonde, Elva House, Jacob Zidek, Lisa Smith, Ph.D., Jack Chen, Ph.D.
hCoV-19/USA/AK-PHL8417/2021	EPI_ISL_1967920	4/24/2021	Alaska State Virology Laboratory	Alaska State Virology Laboratory	Stephanie DeRonde, Elva House, Jacob Zidek, Lisa Smith, Ph.D., Jack Chen, Ph.D.
hCoV-19/USA/AK-PHL8418/2021	EPI_ISL_1967921	4/25/2021	Alaska State Virology Laboratory	Alaska State Virology Laboratory	Stephanie DeRonde, Elva House, Jacob Zidek, Lisa Smith, Ph.D., Jack Chen, Ph.D.

hCoV-19/USA/AK-PHL8420/2021	EPI_ISL_1967923	4/25/2021	Alaska State Virology Laboratory	Alaska State Virology Laboratory	Stephanie DeRonde, Elva House, Jacob Zidek, Lisa Smith, Ph.D., Jack Chen, Ph.D.
hCoV-19/USA/AK-PHL8421/2021	EPI_ISL_1967924	4/24/2021	Alaska State Virology Laboratory	Alaska State Virology Laboratory	Stephanie DeRonde, Elva House, Jacob Zidek, Lisa Smith, Ph.D., Jack Chen, Ph.D.
hCoV-19/USA/AK-PHL8422/2021	EPI_ISL_1967925	4/26/2021	Alaska State Virology Laboratory	Alaska State Virology Laboratory	Stephanie DeRonde, Elva House, Jacob Zidek, Lisa Smith, Ph.D., Jack Chen, Ph.D.
hCoV-19/USA/AK-PHL8423/2021	EPI_ISL_1967926	4/24/2021	Alaska State Virology Laboratory	Alaska State Virology Laboratory	Stephanie DeRonde, Elva House, Jacob Zidek, Lisa Smith, Ph.D., Jack Chen, Ph.D.
hCoV-19/USA/AK-PHL8426/2021	EPI_ISL_1967927	4/25/2021	Alaska State Virology Laboratory	Alaska State Virology Laboratory	Stephanie DeRonde, Elva House, Jacob Zidek, Lisa Smith, Ph.D., Jack Chen, Ph.D.
hCoV-19/USA/AK-PHL8427/2021	EPI_ISL_1967928	4/27/2021	Alaska State Virology Laboratory	Alaska State Virology Laboratory	Stephanie DeRonde, Elva House, Jacob Zidek, Lisa Smith, Ph.D., Jack Chen, Ph.D.
hCoV-19/USA/AK-PHL8428/2021	EPI_ISL_1967929	4/26/2021	Alaska State Virology Laboratory	Alaska State Virology Laboratory	Stephanie DeRonde, Elva House, Jacob Zidek, Lisa Smith, Ph.D., Jack Chen, Ph.D.
hCoV-19/USA/AK-PHL8429/2021	EPI_ISL_1967930	4/24/2021	Alaska State Virology Laboratory	Alaska State Virology Laboratory	Stephanie DeRonde, Elva House, Jacob Zidek, Lisa Smith, Ph.D., Jack Chen, Ph.D.
hCoV-19/USA/AK-PHL8430/2021	EPI_ISL_1967931	4/27/2021	Alaska State Virology Laboratory	Alaska State Virology Laboratory	Stephanie DeRonde, Elva House, Jacob Zidek, Lisa Smith, Ph.D., Jack Chen, Ph.D.
hCoV-19/USA/AK-PHL8431/2021	EPI_ISL_1967932	4/27/2021	Alaska State Virology Laboratory	Alaska State Virology Laboratory	Stephanie DeRonde, Elva House, Jacob Zidek, Lisa Smith, Ph.D., Jack Chen, Ph.D.
hCoV-19/USA/AK-PHL8432/2021	EPI_ISL_1967933	4/26/2021	Alaska State Virology Laboratory	Alaska State Virology Laboratory	Stephanie DeRonde, Elva House, Jacob Zidek, Lisa Smith, Ph.D., Jack Chen, Ph.D.
hCoV-19/USA/AK-PHL8433/2021	EPI_ISL_1967934	4/24/2021	Alaska State Virology Laboratory	Alaska State Virology Laboratory	Stephanie DeRonde, Elva House, Jacob Zidek, Lisa Smith, Ph.D., Jack Chen, Ph.D.
hCoV-19/USA/AK-PHL8435/2021	EPI_ISL_1967935	4/26/2021	Alaska State Virology Laboratory	Alaska State Virology Laboratory	Stephanie DeRonde, Elva House, Jacob Zidek, Lisa Smith, Ph.D., Jack Chen, Ph.D.
hCoV-19/USA/AK-PHL8436/2021	EPI_ISL_1967936	4/29/2021	Alaska State Virology Laboratory	Alaska State Virology Laboratory	Stephanie DeRonde, Elva House, Jacob Zidek, Lisa Smith, Ph.D., Jack Chen, Ph.D.
hCoV-19/USA/AK-PHL8437/2021	EPI_ISL_1967937	4/28/2021	Alaska State Virology Laboratory	Alaska State Virology Laboratory	Stephanie DeRonde, Elva House, Jacob Zidek, Lisa Smith, Ph.D., Jack Chen, Ph.D.
hCoV-19/USA/AK-PHL8438/2021	EPI_ISL_1967938	4/28/2021	Alaska State Virology Laboratory	Alaska State Virology Laboratory	Stephanie DeRonde, Elva House, Jacob Zidek, Lisa Smith, Ph.D., Jack Chen, Ph.D.
hCoV-19/USA/AK-PHL8439/2021	EPI_ISL_1967939	4/28/2021	Alaska State Virology Laboratory	Alaska State Virology Laboratory	Stephanie DeRonde, Elva House, Jacob Zidek, Lisa Smith, Ph.D., Jack Chen, Ph.D.
hCoV-19/USA/AK-PHL8440/2021	EPI_ISL_1967940	4/27/2021	Alaska State Virology Laboratory	Alaska State Virology Laboratory	Stephanie DeRonde, Elva House, Jacob Zidek, Lisa Smith, Ph.D., Jack Chen, Ph.D.
hCoV-19/USA/AK-CDC-ASC210069231/2021	EPI_ISL_1999454	4/12/2021	Aegis Sciences Corporation	Centers for Disease Control and Prevention Division of Viral Diseases, Pathogen Discovery	Dakota Howard, Dhwani Batra, Peter W. Cook, Kara Moser, Adrian Paskey, Jason Caravas, Benjamin Rambo-Martin, Shatavia Morrison, Christopher Gulvick, Scott Sammons, Yvette Unoarumhi, Darlene Wagner, Matthew Schmerer, Cyndi Clark, Patrick Campbell, Rob Case, Vikramsinha Ghorpade, Holly Houdeshell, Ola Kvalvaag, Dillon Nall, Ethan Sanders, Alec Vest, Shaun Westlund, Matthew Hardison, Clinton R. Paden, Duncan MacCannell
hCoV-19/USA/AK-CDC-ASC210069249/2021	EPI_ISL_1999469	4/12/2021	Aegis Sciences Corporation	Centers for Disease Control and Prevention Division of Viral Diseases, Pathogen Discovery	Dakota Howard, Dhwani Batra, Peter W. Cook, Kara Moser, Adrian Paskey, Jason Caravas, Benjamin Rambo-Martin, Shatavia Morrison, Christopher Gulvick, Scott Sammons, Yvette Unoarumhi, Darlene Wagner, Matthew Schmerer, Cyndi Clark, Patrick Campbell, Rob Case, Vikramsinha Ghorpade, Holly Houdeshell, Ola Kvalvaag, Dillon Nall, Ethan Sanders, Alec Vest, Shaun Westlund, Matthew Hardison, Clinton R. Paden, Duncan MacCannell
hCoV-19/USA/AK-CDC-ASC210069251/2021	EPI_ISL_1999471	4/12/2021	Aegis Sciences Corporation	Centers for Disease Control and Prevention Division of Viral Diseases, Pathogen Discovery	Dakota Howard, Dhwani Batra, Peter W. Cook, Kara Moser, Adrian Paskey, Jason Caravas, Benjamin Rambo-Martin, Shatavia Morrison, Christopher Gulvick, Scott Sammons, Yvette Unoarumhi, Darlene Wagner, Matthew Schmerer, Cyndi Clark, Patrick Campbell, Rob Case, Vikramsinha Ghorpade, Holly Houdeshell, Ola Kvalvaag, Dillon Nall, Ethan Sanders, Alec Vest, Shaun Westlund, Matthew Hardison, Clinton R. Paden, Duncan MacCannell
hCoV-19/USA/AK-PHL8526/2021	EPI_ISL_2008978	4/30/2021	Alaska State Virology Laboratory	Alaska State Virology Laboratory	Stephanie DeRonde, Elva House, Jacob Zidek, Lisa Smith, Ph.D., Jack Chen, Ph.D.
hCoV-19/USA/AK-PHL8483/2021	EPI_ISL_2008979	5/1/2021	Alaska State Virology Laboratory	Alaska State Virology Laboratory	Stephanie DeRonde, Elva House, Jacob Zidek, Lisa Smith, Ph.D., Jack Chen, Ph.D.
hCoV-19/USA/AK-PHL8485/2021	EPI_ISL_2008980	4/30/2021	Alaska State Virology Laboratory	Alaska State Virology Laboratory	Stephanie DeRonde, Elva House, Jacob Zidek, Lisa Smith, Ph.D., Jack Chen, Ph.D.
hCoV-19/USA/AK-PHL8488/2021	EPI_ISL_2008981	5/1/2021	Alaska State Virology Laboratory	Alaska State Virology Laboratory	Stephanie DeRonde, Elva House, Jacob Zidek, Lisa Smith, Ph.D., Jack Chen, Ph.D.
hCoV-19/USA/AK-PHL8489/2021	EPI_ISL_2008982	5/1/2021	Alaska State Virology Laboratory	Alaska State Virology Laboratory	Stephanie DeRonde, Elva House, Jacob Zidek, Lisa Smith, Ph.D., Jack Chen, Ph.D.
hCoV-19/USA/AK-PHL8519/2021	EPI_ISL_2008983	4/25/2021	Alaska State Virology Laboratory	Alaska State Virology Laboratory	Stephanie DeRonde, Elva House, Jacob Zidek, Lisa Smith, Ph.D., Jack Chen, Ph.D.
hCoV-19/USA/AK-PHL8524/2021	EPI_ISL_2008984	4/29/2021	Alaska State Virology Laboratory	Alaska State Virology Laboratory	Stephanie DeRonde, Elva House, Jacob Zidek, Lisa Smith, Ph.D., Jack Chen, Ph.D.
hCoV-19/USA/AK-PHL8487/2021	EPI_ISL_2008985	5/1/2021	Alaska State Virology Laboratory	Alaska State Virology Laboratory	Stephanie DeRonde, Elva House, Jacob Zidek, Lisa Smith, Ph.D., Jack Chen, Ph.D.
hCoV-19/USA/AK-PHL8496/2021	EPI_ISL_2008986	4/21/2021	Alaska State Virology Laboratory	Alaska State Virology Laboratory	Stephanie DeRonde, Elva House, Jacob Zidek, Lisa Smith, Ph.D., Jack Chen, Ph.D.
hCoV-19/USA/AK-PHL8497/2021	EPI_ISL_2008987	4/20/2021	Alaska State Virology Laboratory	Alaska State Virology Laboratory	Stephanie DeRonde, Elva House, Jacob Zidek, Lisa Smith, Ph.D., Jack Chen, Ph.D.
hCoV-19/USA/AK-PHL8499/2021	EPI_ISL_2008988	4/22/2021	Alaska State Virology Laboratory	Alaska State Virology Laboratory	Stephanie DeRonde, Elva House, Jacob Zidek, Lisa Smith, Ph.D., Jack Chen, Ph.D.
hCoV-19/USA/AK-PHL8512/2021	EPI_ISL_2008989	5/1/2021	Alaska State Virology Laboratory	Alaska State Virology Laboratory	Stephanie DeRonde, Elva House, Jacob Zidek, Lisa Smith, Ph.D., Jack Chen, Ph.D.
hCoV-19/USA/AK-PHL8486/2021	EPI_ISL_2008991	4/29/2021	Alaska State Virology Laboratory	Alaska State Virology Laboratory	Stephanie DeRonde, Elva House, Jacob Zidek, Lisa Smith, Ph.D., Jack Chen, Ph.D.
hCoV-19/USA/AK-PHL8492/2021	EPI_ISL_2008992	4/21/2021	Alaska State Virology Laboratory	Alaska State Virology Laboratory	Stephanie DeRonde, Elva House, Jacob Zidek, Lisa Smith, Ph.D., Jack Chen, Ph.D.
hCoV-19/USA/AK-PHL8505/2021	EPI_ISL_2008993	4/1/2021	Alaska State Virology Laboratory	Alaska State Virology Laboratory	Stephanie DeRonde, Elva House, Jacob Zidek, Lisa Smith, Ph.D., Jack Chen, Ph.D.
hCoV-19/USA/AK-PHL8517/2021	EPI_ISL_2008994	4/27/2021	Alaska State Virology Laboratory	Alaska State Virology Laboratory	Stephanie DeRonde, Elva House, Jacob Zidek, Lisa Smith, Ph.D., Jack Chen, Ph.D.

hCoV-19/USA/AK-PHL8501/2021	EPI_ISL_2008996	4/29/2021	Alaska State Virology Laboratory	Alaska State Virology Laboratory	Stephanie DeRonde, Elva House, Jacob Zidek, Lisa Smith, Ph.D., Jack Chen, Ph.D.
hCoV-19/USA/AK-PHL8484/2021	EPI_ISL_2008997	4/28/2021	Alaska State Virology Laboratory	Alaska State Virology Laboratory	Stephanie DeRonde, Elva House, Jacob Zidek, Lisa Smith, Ph.D., Jack Chen, Ph.D.
hCoV-19/USA/AK-PHL8493/2021	EPI_ISL_2008999	4/19/2021	Alaska State Virology Laboratory	Alaska State Virology Laboratory	Stephanie DeRonde, Elva House, Jacob Zidek, Lisa Smith, Ph.D., Jack Chen, Ph.D.
hCoV-19/USA/AK-PHL8494/2021	EPI_ISL_2009000	4/16/2021	Alaska State Virology Laboratory	Alaska State Virology Laboratory	Stephanie DeRonde, Elva House, Jacob Zidek, Lisa Smith, Ph.D., Jack Chen, Ph.D.
hCoV-19/USA/AK-PHL8495/2021	EPI_ISL_2009001	4/19/2021	Alaska State Virology Laboratory	Alaska State Virology Laboratory	Stephanie DeRonde, Elva House, Jacob Zidek, Lisa Smith, Ph.D., Jack Chen, Ph.D.
hCoV-19/USA/AK-PHL8510/2021	EPI_ISL_2009005	5/1/2021	Alaska State Virology Laboratory	Alaska State Virology Laboratory	Stephanie DeRonde, Elva House, Jacob Zidek, Lisa Smith, Ph.D., Jack Chen, Ph.D.
hCoV-19/USA/AK-PHL8511/2021	EPI_ISL_2009006	4/30/2021	Alaska State Virology Laboratory	Alaska State Virology Laboratory	Stephanie DeRonde, Elva House, Jacob Zidek, Lisa Smith, Ph.D., Jack Chen, Ph.D.
hCoV-19/USA/AK-PHL8514/2021	EPI_ISL_2009007	5/1/2021	Alaska State Virology Laboratory	Alaska State Virology Laboratory	Stephanie DeRonde, Elva House, Jacob Zidek, Lisa Smith, Ph.D., Jack Chen, Ph.D.
hCoV-19/USA/AK-PHL8515/2021	EPI_ISL_2009008	5/1/2021	Alaska State Virology Laboratory	Alaska State Virology Laboratory	Stephanie DeRonde, Elva House, Jacob Zidek, Lisa Smith, Ph.D., Jack Chen, Ph.D.
hCoV-19/USA/AK-PHL8516/2021	EPI_ISL_2009009	4/27/2021	Alaska State Virology Laboratory	Alaska State Virology Laboratory	Stephanie DeRonde, Elva House, Jacob Zidek, Lisa Smith, Ph.D., Jack Chen, Ph.D.
hCoV-19/USA/AK-PHL8525/2021	EPI_ISL_2009013	4/30/2021	Alaska State Virology Laboratory	Alaska State Virology Laboratory	Stephanie DeRonde, Elva House, Jacob Zidek, Lisa Smith, Ph.D., Jack Chen, Ph.D.
hCoV-19/USA/AK-PHL8527/2021	EPI_ISL_2009014	4/30/2021	Alaska State Virology Laboratory	Alaska State Virology Laboratory	Stephanie DeRonde, Elva House, Jacob Zidek, Lisa Smith, Ph.D., Jack Chen, Ph.D.
hCoV-19/USA/AK-CDC-2-4306914/2021	EPI_ISL_2018247	4/16/2021	AK State Public Health Lab, State Health Department	Centers for Disease Control and Prevention Division of Viral Diseases, Pathogen Discovery	Mili Sheth, Sarah Nobles, Jasmine Padilla, Mark Burroughs, Shoshona Le, Katie Dillon, Peter Cook, Clinton R. Paden, Dhwani Batra, Krista Queen, Kristen Knipe, Dakota Howard, Yvette Unoarumhi, Darlene Wagner, Matthew Schmerer, Ben L. Rambo-Martin, Kristine Lacek, Sam Shepard, Alison Laufer Halpin, Dave Wentworth, Vivien Dugan, Suxiang Tong, Justin Lee
hCoV-19/USA/AK-CDC-2-4306913/2021	EPI_ISL_2018248	4/18/2021	AK State Public Health Lab, State Health Department	Centers for Disease Control and Prevention Division of Viral Diseases, Pathogen Discovery	Mili Sheth, Sarah Nobles, Jasmine Padilla, Mark Burroughs, Shoshona Le, Katie Dillon, Peter Cook, Clinton R. Paden, Dhwani Batra, Krista Queen, Kristen Knipe, Dakota Howard, Yvette Unoarumhi, Darlene Wagner, Matthew Schmerer, Ben L. Rambo-Martin, Kristine Lacek, Sam Shepard, Alison Laufer Halpin, Dave Wentworth, Vivien Dugan, Suxiang Tong, Justin Lee
hCoV-19/USA/AK-CDC-2-4307102/2021	EPI_ISL_2018249	4/12/2021	AK State Public Health Lab, State Health Department	Centers for Disease Control and Prevention Division of Viral Diseases, Pathogen Discovery	Mili Sheth, Sarah Nobles, Jasmine Padilla, Mark Burroughs, Shoshona Le, Katie Dillon, Peter Cook, Clinton R. Paden, Dhwani Batra, Krista Queen, Kristen Knipe, Dakota Howard, Yvette Unoarumhi, Darlene Wagner, Matthew Schmerer, Ben L. Rambo-Martin, Kristine Lacek, Sam Shepard, Alison Laufer Halpin, Dave Wentworth, Vivien Dugan, Suxiang Tong, Justin Lee
hCoV-19/USA/AK-CDC-2-4307033/2021	EPI_ISL_2018250	4/15/2021	AK State Public Health Lab, State Health Department	Centers for Disease Control and Prevention Division of Viral Diseases, Pathogen Discovery	Mili Sheth, Sarah Nobles, Jasmine Padilla, Mark Burroughs, Shoshona Le, Katie Dillon, Peter Cook, Clinton R. Paden, Dhwani Batra, Krista Queen, Kristen Knipe, Dakota Howard, Yvette Unoarumhi, Darlene Wagner, Matthew Schmerer, Ben L. Rambo-Martin, Kristine Lacek, Sam Shepard, Alison Laufer Halpin, Dave Wentworth, Vivien Dugan, Suxiang Tong, Justin Lee
hCoV-19/USA/AK-CDC-2-4306933/2021	EPI_ISL_2018251	4/12/2021	AK State Public Health Lab, State Health Department	Centers for Disease Control and Prevention Division of Viral Diseases, Pathogen Discovery	Mili Sheth, Sarah Nobles, Jasmine Padilla, Mark Burroughs, Shoshona Le, Katie Dillon, Peter Cook, Clinton R. Paden, Dhwani Batra, Krista Queen, Kristen Knipe, Dakota Howard, Yvette Unoarumhi, Darlene Wagner, Matthew Schmerer, Ben L. Rambo-Martin, Kristine Lacek, Sam Shepard, Alison Laufer Halpin, Dave Wentworth, Vivien Dugan, Suxiang Tong, Justin Lee
hCoV-19/USA/AK-CDC-2-4307032/2021	EPI_ISL_2018252	4/18/2021	AK State Public Health Lab, State Health Department	Centers for Disease Control and Prevention Division of Viral Diseases, Pathogen Discovery	Mili Sheth, Sarah Nobles, Jasmine Padilla, Mark Burroughs, Shoshona Le, Katie Dillon, Peter Cook, Clinton R. Paden, Dhwani Batra, Krista Queen, Kristen Knipe, Dakota Howard, Yvette Unoarumhi, Darlene Wagner, Matthew Schmerer, Ben L. Rambo-Martin, Kristine Lacek, Sam Shepard, Alison Laufer Halpin, Dave Wentworth, Vivien Dugan, Suxiang Tong, Justin Lee
hCoV-19/USA/AK-CDC-2-4307041/2021	EPI_ISL_2018253	4/18/2021	AK State Public Health Lab, State Health Department	Centers for Disease Control and Prevention Division of Viral Diseases, Pathogen Discovery	Mili Sheth, Sarah Nobles, Jasmine Padilla, Mark Burroughs, Shoshona Le, Katie Dillon, Peter Cook, Clinton R. Paden, Dhwani Batra, Krista Queen, Kristen Knipe, Dakota Howard, Yvette Unoarumhi, Darlene Wagner, Matthew Schmerer, Ben L. Rambo-Martin, Kristine Lacek, Sam Shepard, Alison Laufer Halpin, Dave Wentworth, Vivien Dugan, Suxiang Tong, Justin Lee
hCoV-19/USA/AK-CDC-2-4306935/2021	EPI_ISL_2018254	4/18/2021	AK State Public Health Lab, State Health Department	Centers for Disease Control and Prevention Division of Viral Diseases, Pathogen Discovery	Mili Sheth, Sarah Nobles, Jasmine Padilla, Mark Burroughs, Shoshona Le, Katie Dillon, Peter Cook, Clinton R. Paden, Dhwani Batra, Krista Queen, Kristen Knipe, Dakota Howard, Yvette Unoarumhi, Darlene Wagner, Matthew Schmerer, Ben L. Rambo-Martin, Kristine Lacek, Sam Shepard, Alison Laufer Halpin, Dave Wentworth, Vivien Dugan, Suxiang Tong, Justin Lee

hCoV-19/USA/AK-CDC-2-4307009/2021	EPI_ISL_2018255	4/21/2021	AK State Public Health Lab, State Health Department	Centers for Disease Control and Prevention Division of Viral Diseases, Pathogen Discovery	Mili Sheth, Sarah Nobles, Jasmine Padilla, Mark Burroughs, Shoshona Le, Katie Dillon, Peter Cook, Clinton R. Paden, Dhwani Batra, Krista Queen, Kristen Knipe, Dakota Howard, Yvette Unoarumhi, Darlene Wagner, Matthew Schmerer, Ben L. Rambo-Martin, Kristine Lacek, Sam Shepard, Alison Laufer Halpin, Dave Wentworth, Vivien Dugan, Suxiang Tong, Justin Lee
hCoV-19/USA/AK-CDC-2-4307021/2021	EPI_ISL_2018256	4/20/2021	AK State Public Health Lab, State Health Department	Centers for Disease Control and Prevention Division of Viral Diseases, Pathogen Discovery	Mili Sheth, Sarah Nobles, Jasmine Padilla, Mark Burroughs, Shoshona Le, Katie Dillon, Peter Cook, Clinton R. Paden, Dhwani Batra, Krista Queen, Kristen Knipe, Dakota Howard, Yvette Unoarumhi, Darlene Wagner, Matthew Schmerer, Ben L. Rambo-Martin, Kristine Lacek, Sam Shepard, Alison Laufer Halpin, Dave Wentworth, Vivien Dugan, Suxiang Tong, Justin Lee
hCoV-19/USA/AK-CDC-2-4306932/2021	EPI_ISL_2018257	4/18/2021	AK State Public Health Lab, State Health Department	Centers for Disease Control and Prevention Division of Viral Diseases, Pathogen Discovery	Mili Sheth, Sarah Nobles, Jasmine Padilla, Mark Burroughs, Shoshona Le, Katie Dillon, Peter Cook, Clinton R. Paden, Dhwani Batra, Krista Queen, Kristen Knipe, Dakota Howard, Yvette Unoarumhi, Darlene Wagner, Matthew Schmerer, Ben L. Rambo-Martin, Kristine Lacek, Sam Shepard, Alison Laufer Halpin, Dave Wentworth, Vivien Dugan, Suxiang Tong, Justin Lee
hCoV-19/USA/AK-CDC-2-4307016/2021	EPI_ISL_2018258	4/12/2021	AK State Public Health Lab, State Health Department	Centers for Disease Control and Prevention Division of Viral Diseases, Pathogen Discovery	Mili Sheth, Sarah Nobles, Jasmine Padilla, Mark Burroughs, Shoshona Le, Katie Dillon, Peter Cook, Clinton R. Paden, Dhwani Batra, Krista Queen, Kristen Knipe, Dakota Howard, Yvette Unoarumhi, Darlene Wagner, Matthew Schmerer, Ben L. Rambo-Martin, Kristine Lacek, Sam Shepard, Alison Laufer Halpin, Dave Wentworth, Vivien Dugan, Suxiang Tong, Justin Lee
hCoV-19/USA/AK-CDC-2-4306946/2021	EPI_ISL_2018260	4/11/2021	AK State Public Health Lab, State Health Department	Centers for Disease Control and Prevention Division of Viral Diseases, Pathogen Discovery	Mili Sheth, Sarah Nobles, Jasmine Padilla, Mark Burroughs, Shoshona Le, Katie Dillon, Peter Cook, Clinton R. Paden, Dhwani Batra, Krista Queen, Kristen Knipe, Dakota Howard, Yvette Unoarumhi, Darlene Wagner, Matthew Schmerer, Ben L. Rambo-Martin, Kristine Lacek, Sam Shepard, Alison Laufer Halpin, Dave Wentworth, Vivien Dugan, Suxiang Tong, Justin Lee
hCoV-19/USA/AK-CDC-2-4306943/2021	EPI_ISL_2018261	4/16/2021	AK State Public Health Lab, State Health Department	Centers for Disease Control and Prevention Division of Viral Diseases, Pathogen Discovery	Mili Sheth, Sarah Nobles, Jasmine Padilla, Mark Burroughs, Shoshona Le, Katie Dillon, Peter Cook, Clinton R. Paden, Dhwani Batra, Krista Queen, Kristen Knipe, Dakota Howard, Yvette Unoarumhi, Darlene Wagner, Matthew Schmerer, Ben L. Rambo-Martin, Kristine Lacek, Sam Shepard, Alison Laufer Halpin, Dave Wentworth, Vivien Dugan, Suxiang Tong, Justin Lee
hCoV-19/USA/AK-CDC-2-4307040/2021	EPI_ISL_2018262	4/16/2021	AK State Public Health Lab, State Health Department	Centers for Disease Control and Prevention Division of Viral Diseases, Pathogen Discovery	Mili Sheth, Sarah Nobles, Jasmine Padilla, Mark Burroughs, Shoshona Le, Katie Dillon, Peter Cook, Clinton R. Paden, Dhwani Batra, Krista Queen, Kristen Knipe, Dakota Howard, Yvette Unoarumhi, Darlene Wagner, Matthew Schmerer, Ben L. Rambo-Martin, Kristine Lacek, Sam Shepard, Alison Laufer Halpin, Dave Wentworth, Vivien Dugan, Suxiang Tong, Justin Lee
hCoV-19/USA/AK-CDC-2-4306944/2021	EPI_ISL_2018263	4/19/2021	AK State Public Health Lab, State Health Department	Centers for Disease Control and Prevention Division of Viral Diseases, Pathogen Discovery	Mili Sheth, Sarah Nobles, Jasmine Padilla, Mark Burroughs, Shoshona Le, Katie Dillon, Peter Cook, Clinton R. Paden, Dhwani Batra, Krista Queen, Kristen Knipe, Dakota Howard, Yvette Unoarumhi, Darlene Wagner, Matthew Schmerer, Ben L. Rambo-Martin, Kristine Lacek, Sam Shepard, Alison Laufer Halpin, Dave Wentworth, Vivien Dugan, Suxiang Tong, Justin Lee
hCoV-19/USA/AK-PHL8674/2021	EPI_ISL_2213219	4/29/2021	Alaska State Virology Laboratory	Alaska State Virology Laboratory	Stephanie DeRonde, Elva House, Jacob Zidek, Lisa Smith, Ph.D., Jack Chen, Ph.D.
hCoV-19/USA/AK-CDC-ASC210074931/2021	EPI_ISL_2042407	4/18/2021	Aegis Sciences Corporation	Centers for Disease Control and Prevention Division of Viral Diseases, Pathogen Discovery	Dakota Howard, Dhwani Batra, Peter W. Cook, Kara Moser, Adrian Paskey, Jason Caravas, Benjamin Rambo-Martin, Shatavia Morrison, Christopher Gulvick, Scott Sammons, Yvette Unoarumhi, Darlene Wagner, Matthew Schmerer, Cyndi Clark, Patrick Campbell, Rob Case, Vikramsinha Ghorpade, Holly Houdeshell, Ola Kvalvaag, Dillon Nall, Ethan Sanders, Alec Vest, Shaun Westlund, Matthew Hardison, Clinton R. Paden, Duncan MacCannell

hCoV-19/USA/AK-CDC-LC0056023/2021	EPI_ISL_2046163	4/28/2021	Laboratory Corporation of America	Centers for Disease Control and Prevention Division of Viral Diseases, Pathogen Discovery	Dakota Howard, Dhwani Batra, Peter W. Cook, Kara Moser, Adrian Paskey, Jason Caravas, Benjamin Rambo-Martin, Shatavia Morrison, Christopher Gulvick, Scott Sammons, Yvette Unoarumhi, Darlene Wagner, Matthew Schmerer, Minoo Agarwal, Eyad Almasri, Debbie Boles, Ayla Burns, Nuthawin Charoensri, Oren Cohen, Susan Countryman, Mary Ann Cristobal, Bobbi Croy, Suzanne Dale, Hrushikesh Deshmukh, Amanda Douglas, Vincent Drouillon, Marcia Eisenberg, Howard Engler, Rama Ghatti, Prashant Gupta, Susan Hicks, Jake Humphrey, Lax Iyer, Manoj Jain, Mohan Kolli, Brian Krueger, Tim Kuphal, Stanley Letovsky, Michael Levandoski, Craig Lukasik, Jonathan Meltzer, Brian Norvell, Mindy Nye, Scott Parker, Christos Petropoulos, John Pruitt, Steven Ragan, Scott Ryan, Mike Sapeta, Jana Schroth, Suresh Babu Selvaraju, Goran Stevovic, Amanda Suchanek, Andrea Throop, Lyndon Tilson, Thomas Urban, Joe Voshell, Kimberly Wagner, Jonathan Williams, Mary Williamson, Qian Zeng, Tricia Zwiefelhofer, Clinton R. Paden, Duncan MacCannell
hCoV-19/USA/AK-PHL8530/2021	EPI_ISL_2101749	5/3/2021	Alaska State Virology Laboratory	Alaska State Virology Laboratory	Stephanie DeRonde, Elva House, Jacob Zidek, Lisa Smith, Ph.D., Jack Chen, Ph.D.
hCoV-19/USA/AK-PHL8535/2021	EPI_ISL_2101750	5/3/2021	Alaska State Virology Laboratory	Alaska State Virology Laboratory	Stephanie DeRonde, Elva House, Jacob Zidek, Lisa Smith, Ph.D., Jack Chen, Ph.D.
hCoV-19/USA/AK-PHL8571/2021	EPI_ISL_2101752	5/2/2021	Alaska State Virology Laboratory	Alaska State Virology Laboratory	Stephanie DeRonde, Elva House, Jacob Zidek, Lisa Smith, Ph.D., Jack Chen, Ph.D.
hCoV-19/USA/AK-PHL8531/2021	EPI_ISL_2101753	5/4/2021	Alaska State Virology Laboratory	Alaska State Virology Laboratory	Stephanie DeRonde, Elva House, Jacob Zidek, Lisa Smith, Ph.D., Jack Chen, Ph.D.
hCoV-19/USA/AK-PHL8532/2021	EPI_ISL_2101754	5/1/2021	Alaska State Virology Laboratory	Alaska State Virology Laboratory	Stephanie DeRonde, Elva House, Jacob Zidek, Lisa Smith, Ph.D., Jack Chen, Ph.D.
hCoV-19/USA/AK-PHL8537/2021	EPI_ISL_2101755	5/2/2021	Alaska State Virology Laboratory	Alaska State Virology Laboratory	Stephanie DeRonde, Elva House, Jacob Zidek, Lisa Smith, Ph.D., Jack Chen, Ph.D.
hCoV-19/USA/AK-PHL8540/2021	EPI_ISL_2101756	4/17/2021	Alaska State Virology Laboratory	Alaska State Virology Laboratory	Stephanie DeRonde, Elva House, Jacob Zidek, Lisa Smith, Ph.D., Jack Chen, Ph.D.
hCoV-19/USA/AK-PHL8538/2021	EPI_ISL_2101758	5/2/2021	Alaska State Virology Laboratory	Alaska State Virology Laboratory	Stephanie DeRonde, Elva House, Jacob Zidek, Lisa Smith, Ph.D., Jack Chen, Ph.D.
hCoV-19/USA/AK-PHL8564/2021	EPI_ISL_2101759	5/3/2021	Alaska State Virology Laboratory	Alaska State Virology Laboratory	Stephanie DeRonde, Elva House, Jacob Zidek, Lisa Smith, Ph.D., Jack Chen, Ph.D.
hCoV-19/USA/AK-PHL8536/2021	EPI_ISL_2101760	5/3/2021	Alaska State Virology Laboratory	Alaska State Virology Laboratory	Stephanie DeRonde, Elva House, Jacob Zidek, Lisa Smith, Ph.D., Jack Chen, Ph.D.
hCoV-19/USA/AK-PHL8552/2021	EPI_ISL_2101761	4/29/2021	Alaska State Virology Laboratory	Alaska State Virology Laboratory	Stephanie DeRonde, Elva House, Jacob Zidek, Lisa Smith, Ph.D., Jack Chen, Ph.D.
hCoV-19/USA/AK-PHL8553/2021	EPI_ISL_2101762	4/29/2021	Alaska State Virology Laboratory	Alaska State Virology Laboratory	Stephanie DeRonde, Elva House, Jacob Zidek, Lisa Smith, Ph.D., Jack Chen, Ph.D.
hCoV-19/USA/AK-PHL8555/2021	EPI_ISL_2101763	4/30/2021	Alaska State Virology Laboratory	Alaska State Virology Laboratory	Stephanie DeRonde, Elva House, Jacob Zidek, Lisa Smith, Ph.D., Jack Chen, Ph.D.
hCoV-19/USA/AK-PHL8539/2021	EPI_ISL_2101764	5/2/2021	Alaska State Virology Laboratory	Alaska State Virology Laboratory	Stephanie DeRonde, Elva House, Jacob Zidek, Lisa Smith, Ph.D., Jack Chen, Ph.D.
hCoV-19/USA/AK-PHL8542/2021	EPI_ISL_2101766	4/12/2021	Alaska State Virology Laboratory	Alaska State Virology Laboratory	Stephanie DeRonde, Elva House, Jacob Zidek, Lisa Smith, Ph.D., Jack Chen, Ph.D.
hCoV-19/USA/AK-PHL8548/2021	EPI_ISL_2101767	4/30/2021	Alaska State Virology Laboratory	Alaska State Virology Laboratory	Stephanie DeRonde, Elva House, Jacob Zidek, Lisa Smith, Ph.D., Jack Chen, Ph.D.
hCoV-19/USA/AK-PHL8551/2021	EPI_ISL_2101768	4/29/2021	Alaska State Virology Laboratory	Alaska State Virology Laboratory	Stephanie DeRonde, Elva House, Jacob Zidek, Lisa Smith, Ph.D., Jack Chen, Ph.D.
hCoV-19/USA/AK-PHL8556/2021	EPI_ISL_2101770	4/30/2021	Alaska State Virology Laboratory	Alaska State Virology Laboratory	Stephanie DeRonde, Elva House, Jacob Zidek, Lisa Smith, Ph.D., Jack Chen, Ph.D.
hCoV-19/USA/AK-PHL8558/2021	EPI_ISL_2101771	4/29/2021	Alaska State Virology Laboratory	Alaska State Virology Laboratory	Stephanie DeRonde, Elva House, Jacob Zidek, Lisa Smith, Ph.D., Jack Chen, Ph.D.
hCoV-19/USA/AK-PHL8559/2021	EPI_ISL_2101772	4/29/2021	Alaska State Virology Laboratory	Alaska State Virology Laboratory	Stephanie DeRonde, Elva House, Jacob Zidek, Lisa Smith, Ph.D., Jack Chen, Ph.D.
hCoV-19/USA/AK-PHL8560/2021	EPI_ISL_2101773	5/1/2021	Alaska State Virology Laboratory	Alaska State Virology Laboratory	Stephanie DeRonde, Elva House, Jacob Zidek, Lisa Smith, Ph.D., Jack Chen, Ph.D.
hCoV-19/USA/AK-PHL8563/2021	EPI_ISL_2101774	5/2/2021	Alaska State Virology Laboratory	Alaska State Virology Laboratory	Stephanie DeRonde, Elva House, Jacob Zidek, Lisa Smith, Ph.D., Jack Chen, Ph.D.
hCoV-19/USA/AK-PHL8565/2021	EPI_ISL_2101775	4/30/2021	Alaska State Virology Laboratory	Alaska State Virology Laboratory	Stephanie DeRonde, Elva House, Jacob Zidek, Lisa Smith, Ph.D., Jack Chen, Ph.D.
hCoV-19/USA/AK-PHL8566/2021	EPI_ISL_2101776	5/2/2021	Alaska State Virology Laboratory	Alaska State Virology Laboratory	Stephanie DeRonde, Elva House, Jacob Zidek, Lisa Smith, Ph.D., Jack Chen, Ph.D.
hCoV-19/USA/AK-PHL8572/2021	EPI_ISL_2101777	5/2/2021	Alaska State Virology Laboratory	Alaska State Virology Laboratory	Stephanie DeRonde, Elva House, Jacob Zidek, Lisa Smith, Ph.D., Jack Chen, Ph.D.
hCoV-19/USA/AK-PHL8573/2021	EPI_ISL_2101778	5/1/2021	Alaska State Virology Laboratory	Alaska State Virology Laboratory	Stephanie DeRonde, Elva House, Jacob Zidek, Lisa Smith, Ph.D., Jack Chen, Ph.D.
hCoV-19/USA/AK-PHL8683/2021	EPI_ISL_2136429	5/6/2021	Alaska State Virology Laboratory	Alaska State Virology Laboratory	Stephanie DeRonde, Elva House, Jacob Zidek, Lisa Smith, Ph.D., Jack Chen, Ph.D.
hCoV-19/USA/AK-PHL8684/2021	EPI_ISL_2136430	5/6/2021	Alaska State Virology Laboratory	Alaska State Virology Laboratory	Stephanie DeRonde, Elva House, Jacob Zidek, Lisa Smith, Ph.D., Jack Chen, Ph.D.
hCoV-19/USA/AK-PHL8688/2021	EPI_ISL_2136434	5/6/2021	Alaska State Virology Laboratory	Alaska State Virology Laboratory	Stephanie DeRonde, Elva House, Jacob Zidek, Lisa Smith, Ph.D., Jack Chen, Ph.D.
hCoV-19/USA/AK-PHL8690/2021	EPI_ISL_2136435	5/8/2021	Alaska State Virology Laboratory	Alaska State Virology Laboratory	Stephanie DeRonde, Elva House, Jacob Zidek, Lisa Smith, Ph.D., Jack Chen, Ph.D.
hCoV-19/USA/AK-PHL8691/2021	EPI_ISL_2136436	5/6/2021	Alaska State Virology Laboratory	Alaska State Virology Laboratory	Stephanie DeRonde, Elva House, Jacob Zidek, Lisa Smith, Ph.D., Jack Chen, Ph.D.
hCoV-19/USA/AK-PHL8695/2021	EPI_ISL_2136440	5/6/2021	Alaska State Virology Laboratory	Alaska State Virology Laboratory	Stephanie DeRonde, Elva House, Jacob Zidek, Lisa Smith, Ph.D., Jack Chen, Ph.D.
hCoV-19/USA/AK-PHL8697/2021	EPI_ISL_2136442	5/6/2021	Alaska State Virology Laboratory	Alaska State Virology Laboratory	Stephanie DeRonde, Elva House, Jacob Zidek, Lisa Smith, Ph.D., Jack Chen, Ph.D.
hCoV-19/USA/AK-PHL8698/2021	EPI_ISL_2136443	5/6/2021	Alaska State Virology Laboratory	Alaska State Virology Laboratory	Stephanie DeRonde, Elva House, Jacob Zidek, Lisa Smith, Ph.D., Jack Chen, Ph.D.
hCoV-19/USA/AK-PHL8699/2021	EPI_ISL_2136444	5/6/2021	Alaska State Virology Laboratory	Alaska State Virology Laboratory	Stephanie DeRonde, Elva House, Jacob Zidek, Lisa Smith, Ph.D., Jack Chen, Ph.D.
hCoV-19/USA/AK-PHL8703/2021	EPI_ISL_2136446	5/7/2021	Alaska State Virology Laboratory	Alaska State Virology Laboratory	Stephanie DeRonde, Elva House, Jacob Zidek, Lisa Smith, Ph.D., Jack Chen, Ph.D.
hCoV-19/USA/AK-PHL8706/2021	EPI_ISL_2136448	5/8/2021	Alaska State Virology Laboratory	Alaska State Virology Laboratory	Stephanie DeRonde, Elva House, Jacob Zidek, Lisa Smith, Ph.D., Jack Chen, Ph.D.
hCoV-19/USA/AK-PHL8707/2021	EPI_ISL_2136449	5/8/2021	Alaska State Virology Laboratory	Alaska State Virology Laboratory	Stephanie DeRonde, Elva House, Jacob Zidek, Lisa Smith, Ph.D., Jack Chen, Ph.D.

hCoV-19/USA/AK-PHL8708/2021	EPI_ISL_2136450	5/5/2021	Alaska State Virology Laboratory	Alaska State Virology Laboratory	Stephanie DeRonde, Elva House, Jacob Zidek, Lisa Smith, Ph.D., Jack Chen, Ph.D.
hCoV-19/USA/AK-PHL8709/2021	EPI_ISL_2136451	5/4/2021	Alaska State Virology Laboratory	Alaska State Virology Laboratory	Stephanie DeRonde, Elva House, Jacob Zidek, Lisa Smith, Ph.D., Jack Chen, Ph.D.
hCoV-19/USA/AK-PHL8711/2021	EPI_ISL_2136453	5/4/2021	Alaska State Virology Laboratory	Alaska State Virology Laboratory	Stephanie DeRonde, Elva House, Jacob Zidek, Lisa Smith, Ph.D., Jack Chen, Ph.D.
hCoV-19/USA/AK-PHL8712/2021	EPI_ISL_2136454	5/5/2021	Alaska State Virology Laboratory	Alaska State Virology Laboratory	Stephanie DeRonde, Elva House, Jacob Zidek, Lisa Smith, Ph.D., Jack Chen, Ph.D.
hCoV-19/USA/AK-PHL8713/2021	EPI_ISL_2136455	5/5/2021	Alaska State Virology Laboratory	Alaska State Virology Laboratory	Stephanie DeRonde, Elva House, Jacob Zidek, Lisa Smith, Ph.D., Jack Chen, Ph.D.
hCoV-19/USA/AK-PHL8714/2021	EPI_ISL_2136456	5/4/2021	Alaska State Virology Laboratory	Alaska State Virology Laboratory	Stephanie DeRonde, Elva House, Jacob Zidek, Lisa Smith, Ph.D., Jack Chen, Ph.D.
hCoV-19/USA/AK-PHL8715/2021	EPI_ISL_2136457	5/4/2021	Alaska State Virology Laboratory	Alaska State Virology Laboratory	Stephanie DeRonde, Elva House, Jacob Zidek, Lisa Smith, Ph.D., Jack Chen, Ph.D.
hCoV-19/USA/AK-PHL8717/2021	EPI_ISL_2136458	5/5/2021	Alaska State Virology Laboratory	Alaska State Virology Laboratory	Stephanie DeRonde, Elva House, Jacob Zidek, Lisa Smith, Ph.D., Jack Chen, Ph.D.
hCoV-19/USA/AK-PHL8718/2021	EPI_ISL_2136459	5/5/2021	Alaska State Virology Laboratory	Alaska State Virology Laboratory	Stephanie DeRonde, Elva House, Jacob Zidek, Lisa Smith, Ph.D., Jack Chen, Ph.D.
hCoV-19/USA/AK-PHL8719/2021	EPI_ISL_2136460	5/5/2021	Alaska State Virology Laboratory	Alaska State Virology Laboratory	Stephanie DeRonde, Elva House, Jacob Zidek, Lisa Smith, Ph.D., Jack Chen, Ph.D.
hCoV-19/USA/AK-PHL8721/2021	EPI_ISL_2136462	5/5/2021	Alaska State Virology Laboratory	Alaska State Virology Laboratory	Stephanie DeRonde, Elva House, Jacob Zidek, Lisa Smith, Ph.D., Jack Chen, Ph.D.
hCoV-19/USA/AK-PHL8723/2021	EPI_ISL_2136464	5/5/2021	Alaska State Virology Laboratory	Alaska State Virology Laboratory	Stephanie DeRonde, Elva House, Jacob Zidek, Lisa Smith, Ph.D., Jack Chen, Ph.D.
hCoV-19/USA/AK-PHL8725/2021	EPI_ISL_2136466	5/4/2021	Alaska State Virology Laboratory	Alaska State Virology Laboratory	Stephanie DeRonde, Elva House, Jacob Zidek, Lisa Smith, Ph.D., Jack Chen, Ph.D.
hCoV-19/USA/AK-PHL8726/2021	EPI_ISL_2136467	5/4/2021	Alaska State Virology Laboratory	Alaska State Virology Laboratory	Stephanie DeRonde, Elva House, Jacob Zidek, Lisa Smith, Ph.D., Jack Chen, Ph.D.
hCoV-19/USA/AK-PHL8727/2021	EPI_ISL_2136468	5/4/2021	Alaska State Virology Laboratory	Alaska State Virology Laboratory	Stephanie DeRonde, Elva House, Jacob Zidek, Lisa Smith, Ph.D., Jack Chen, Ph.D.
hCoV-19/USA/AK-PHL8728/2021	EPI_ISL_2136469	5/4/2021	Alaska State Virology Laboratory	Alaska State Virology Laboratory	Stephanie DeRonde, Elva House, Jacob Zidek, Lisa Smith, Ph.D., Jack Chen, Ph.D.
hCoV-19/USA/AK-PHL8575/2021	EPI_ISL_2136748	5/1/2021	Alaska State Virology Laboratory	Alaska State Virology Laboratory	Stephanie DeRonde, Elva House, Jacob Zidek, Lisa Smith, Ph.D., Jack Chen, Ph.D.
hCoV-19/USA/AK-PHL8576/2021	EPI_ISL_2136749	4/30/2021	Alaska State Virology Laboratory	Alaska State Virology Laboratory	Stephanie DeRonde, Elva House, Jacob Zidek, Lisa Smith, Ph.D., Jack Chen, Ph.D.
hCoV-19/USA/AK-PHL8577/2021	EPI_ISL_2136750	4/30/2021	Alaska State Virology Laboratory	Alaska State Virology Laboratory	Stephanie DeRonde, Elva House, Jacob Zidek, Lisa Smith, Ph.D., Jack Chen, Ph.D.
hCoV-19/USA/AK-PHL8579/2021	EPI_ISL_2136751	4/30/2021	Alaska State Virology Laboratory	Alaska State Virology Laboratory	Stephanie DeRonde, Elva House, Jacob Zidek, Lisa Smith, Ph.D., Jack Chen, Ph.D.
hCoV-19/USA/AK-PHL8580/2021	EPI_ISL_2136752	4/30/2021	Alaska State Virology Laboratory	Alaska State Virology Laboratory	Stephanie DeRonde, Elva House, Jacob Zidek, Lisa Smith, Ph.D., Jack Chen, Ph.D.
hCoV-19/USA/AK-PHL8581/2021	EPI_ISL_2136753	4/30/2021	Alaska State Virology Laboratory	Alaska State Virology Laboratory	Stephanie DeRonde, Elva House, Jacob Zidek, Lisa Smith, Ph.D., Jack Chen, Ph.D.
hCoV-19/USA/AK-PHL8582/2021	EPI_ISL_2136754	4/30/2021	Alaska State Virology Laboratory	Alaska State Virology Laboratory	Stephanie DeRonde, Elva House, Jacob Zidek, Lisa Smith, Ph.D., Jack Chen, Ph.D.
hCoV-19/USA/AK-PHL8583/2021	EPI_ISL_2136755	4/30/2021	Alaska State Virology Laboratory	Alaska State Virology Laboratory	Stephanie DeRonde, Elva House, Jacob Zidek, Lisa Smith, Ph.D., Jack Chen, Ph.D.
hCoV-19/USA/AK-PHL8584/2021	EPI_ISL_2136756	5/3/2021	Alaska State Virology Laboratory	Alaska State Virology Laboratory	Stephanie DeRonde, Elva House, Jacob Zidek, Lisa Smith, Ph.D., Jack Chen, Ph.D.
hCoV-19/USA/AK-PHL8585/2021	EPI_ISL_2136757	5/3/2021	Alaska State Virology Laboratory	Alaska State Virology Laboratory	Stephanie DeRonde, Elva House, Jacob Zidek, Lisa Smith, Ph.D., Jack Chen, Ph.D.
hCoV-19/USA/AK-PHL8586/2021	EPI_ISL_2136758	5/3/2021	Alaska State Virology Laboratory	Alaska State Virology Laboratory	Stephanie DeRonde, Elva House, Jacob Zidek, Lisa Smith, Ph.D., Jack Chen, Ph.D.
hCoV-19/USA/AK-PHL8587/2021	EPI_ISL_2136759	5/3/2021	Alaska State Virology Laboratory	Alaska State Virology Laboratory	Stephanie DeRonde, Elva House, Jacob Zidek, Lisa Smith, Ph.D., Jack Chen, Ph.D.
hCoV-19/USA/AK-PHL8588/2021	EPI_ISL_2136760	5/3/2021	Alaska State Virology Laboratory	Alaska State Virology Laboratory	Stephanie DeRonde, Elva House, Jacob Zidek, Lisa Smith, Ph.D., Jack Chen, Ph.D.
hCoV-19/USA/AK-PHL8589/2021	EPI_ISL_2136761	5/3/2021	Alaska State Virology Laboratory	Alaska State Virology Laboratory	Stephanie DeRonde, Elva House, Jacob Zidek, Lisa Smith, Ph.D., Jack Chen, Ph.D.
hCoV-19/USA/AK-PHL8593/2021	EPI_ISL_2136765	5/3/2021	Alaska State Virology Laboratory	Alaska State Virology Laboratory	Stephanie DeRonde, Elva House, Jacob Zidek, Lisa Smith, Ph.D., Jack Chen, Ph.D.
hCoV-19/USA/AK-PHL8595/2021	EPI_ISL_2136767	5/3/2021	Alaska State Virology Laboratory	Alaska State Virology Laboratory	Stephanie DeRonde, Elva House, Jacob Zidek, Lisa Smith, Ph.D., Jack Chen, Ph.D.
hCoV-19/USA/AK-PHL8596/2021	EPI_ISL_2136768	4/29/2021	Alaska State Virology Laboratory	Alaska State Virology Laboratory	Stephanie DeRonde, Elva House, Jacob Zidek, Lisa Smith, Ph.D., Jack Chen, Ph.D.
hCoV-19/USA/AK-PHL8597/2021	EPI_ISL_2136769	4/28/2021	Alaska State Virology Laboratory	Alaska State Virology Laboratory	Stephanie DeRonde, Elva House, Jacob Zidek, Lisa Smith, Ph.D., Jack Chen, Ph.D.
hCoV-19/USA/AK-PHL8598/2021	EPI_ISL_2136770	4/28/2021	Alaska State Virology Laboratory	Alaska State Virology Laboratory	Stephanie DeRonde, Elva House, Jacob Zidek, Lisa Smith, Ph.D., Jack Chen, Ph.D.
hCoV-19/USA/AK-PHL8599/2021	EPI_ISL_2136771	4/28/2021	Alaska State Virology Laboratory	Alaska State Virology Laboratory	Stephanie DeRonde, Elva House, Jacob Zidek, Lisa Smith, Ph.D., Jack Chen, Ph.D.
hCoV-19/USA/AK-PHL8601/2021	EPI_ISL_2136773	4/28/2021	Alaska State Virology Laboratory	Alaska State Virology Laboratory	Stephanie DeRonde, Elva House, Jacob Zidek, Lisa Smith, Ph.D., Jack Chen, Ph.D.
hCoV-19/USA/AK-PHL8602/2021	EPI_ISL_2136774	4/28/2021	Alaska State Virology Laboratory	Alaska State Virology Laboratory	Stephanie DeRonde, Elva House, Jacob Zidek, Lisa Smith, Ph.D., Jack Chen, Ph.D.
hCoV-19/USA/AK-PHL8603/2021	EPI_ISL_2136775	4/27/2021	Alaska State Virology Laboratory	Alaska State Virology Laboratory	Stephanie DeRonde, Elva House, Jacob Zidek, Lisa Smith, Ph.D., Jack Chen, Ph.D.
hCoV-19/USA/AK-PHL8608/2021	EPI_ISL_2136778	4/27/2021	Alaska State Virology Laboratory	Alaska State Virology Laboratory	Stephanie DeRonde, Elva House, Jacob Zidek, Lisa Smith, Ph.D., Jack Chen, Ph.D.
hCoV-19/USA/AK-PHL8609/2021	EPI_ISL_2136779	4/28/2021	Alaska State Virology Laboratory	Alaska State Virology Laboratory	Stephanie DeRonde, Elva House, Jacob Zidek, Lisa Smith, Ph.D., Jack Chen, Ph.D.
hCoV-19/USA/AK-PHL8610/2021	EPI_ISL_2136780	4/28/2021	Alaska State Virology Laboratory	Alaska State Virology Laboratory	Stephanie DeRonde, Elva House, Jacob Zidek, Lisa Smith, Ph.D., Jack Chen, Ph.D.
hCoV-19/USA/AK-PHL8611/2021	EPI_ISL_2136781	5/4/2021	Alaska State Virology Laboratory	Alaska State Virology Laboratory	Stephanie DeRonde, Elva House, Jacob Zidek, Lisa Smith, Ph.D., Jack Chen, Ph.D.
hCoV-19/USA/AK-PHL8612/2021	EPI_ISL_2136782	5/3/2021	Alaska State Virology Laboratory	Alaska State Virology Laboratory	Stephanie DeRonde, Elva House, Jacob Zidek, Lisa Smith, Ph.D., Jack Chen, Ph.D.
hCoV-19/USA/AK-PHL8614/2021	EPI_ISL_2136784	4/30/2021	Alaska State Virology Laboratory	Alaska State Virology Laboratory	Stephanie DeRonde, Elva House, Jacob Zidek, Lisa Smith, Ph.D., Jack Chen, Ph.D.
hCoV-19/USA/AK-PHL8616/2021	EPI_ISL_2136786	4/30/2021	Alaska State Virology Laboratory	Alaska State Virology Laboratory	Stephanie DeRonde, Elva House, Jacob Zidek, Lisa Smith, Ph.D., Jack Chen, Ph.D.
hCoV-19/USA/AK-PHL8617/2021	EPI_ISL_2136787	4/30/2021	Alaska State Virology Laboratory	Alaska State Virology Laboratory	Stephanie DeRonde, Elva House, Jacob Zidek, Lisa Smith, Ph.D., Jack Chen, Ph.D.
hCoV-19/USA/AK-PHL8618/2021	EPI_ISL_2136788	5/1/2021	Alaska State Virology Laboratory	Alaska State Virology Laboratory	Stephanie DeRonde, Elva House, Jacob Zidek, Lisa Smith, Ph.D., Jack Chen, Ph.D.
hCoV-19/USA/AK-PHL8619/2021	EPI_ISL_2136789	5/3/2021	Alaska State Virology Laboratory	Alaska State Virology Laboratory	Stephanie DeRonde, Elva House, Jacob Zidek, Lisa Smith, Ph.D., Jack Chen, Ph.D.

hCoV-19/USA/AK-CDC-ASC210059767/2021	EPI_ISL_2146300	5/6/2021	Aegis Sciences Corporation	Centers for Disease Control and Prevention Division of Viral Diseases, Pathogen Discovery	Dakota Howard, Dhwani Batra, Peter W. Cook, Kara Moser, Adrian Paskey, Jason Caravas, Benjamin Rambo-Martin, Shatavia Morrison, Christopher Gulvick, Scott Sammons, Yvette Unoarumhi, Darlene Wagner, Matthew Schmerer, Cyndi Clark, Patrick Campbell, Rob Case, Vikramsinha Ghorpade, Holly Houdeshell, Ola Kvalvaag, Dillon Nall, Ethan Sanders, Alec Vest, Shaun Westlund, Matthew Hardison, Clinton R. Paden, Duncan MacCannell
hCoV-19/USA/AK-CDC-ASC210037582/2021	EPI_ISL_2148263	4/29/2021	Aegis Sciences Corporation	Centers for Disease Control and Prevention Division of Viral Diseases, Pathogen Discovery	Dakota Howard, Dhwani Batra, Peter W. Cook, Kara Moser, Adrian Paskey, Jason Caravas, Benjamin Rambo-Martin, Shatavia Morrison, Christopher Gulvick, Scott Sammons, Yvette Unoarumhi, Darlene Wagner, Matthew Schmerer, Cyndi Clark, Patrick Campbell, Rob Case, Vikramsinha Ghorpade, Holly Houdeshell, Ola Kvalvaag, Dillon Nall, Ethan Sanders, Alec Vest, Shaun Westlund, Matthew Hardison, Clinton R. Paden, Duncan MacCannell
hCoV-19/USA/AK-CDC-ASC210029183/2021	EPI_ISL_2150613	4/20/2021	Aegis Sciences Corporation	Centers for Disease Control and Prevention Division of Viral Diseases, Pathogen Discovery	Dakota Howard, Dhwani Batra, Peter W. Cook, Kara Moser, Adrian Paskey, Jason Caravas, Benjamin Rambo-Martin, Shatavia Morrison, Christopher Gulvick, Scott Sammons, Yvette Unoarumhi, Darlene Wagner, Matthew Schmerer, Cyndi Clark, Patrick Campbell, Rob Case, Vikramsinha Ghorpade, Holly Houdeshell, Ola Kvalvaag, Dillon Nall, Ethan Sanders, Alec Vest, Shaun Westlund, Matthew Hardison, Clinton R. Paden, Duncan MacCannell
hCoV-19/USA/AK-CDC-ASC210030856/2021	EPI_ISL_2150961	4/21/2021	Aegis Sciences Corporation	Centers for Disease Control and Prevention Division of Viral Diseases, Pathogen Discovery	Dakota Howard, Dhwani Batra, Peter W. Cook, Kara Moser, Adrian Paskey, Jason Caravas, Benjamin Rambo-Martin, Shatavia Morrison, Christopher Gulvick, Scott Sammons, Yvette Unoarumhi, Darlene Wagner, Matthew Schmerer, Cyndi Clark, Patrick Campbell, Rob Case, Vikramsinha Ghorpade, Holly Houdeshell, Ola Kvalvaag, Dillon Nall, Ethan Sanders, Alec Vest, Shaun Westlund, Matthew Hardison, Clinton R. Paden, Duncan MacCannell
hCoV-19/USA/AK-PHL7902/2021	EPI_ISL_2153708	4/3/2021	Alaska State Virology Laboratory	Alaska State Virology Laboratory	Stephanie DeRonde, Elva House, Lisa Smith, Ph.D., Jack Chen, Ph.D.
hCoV-19/USA/AK-PHL8017/2021	EPI_ISL_2153709	4/5/2021	Alaska State Virology Laboratory	Alaska State Virology Laboratory	Stephanie DeRonde, Elva House, Lisa Smith, Ph.D., Jack Chen, Ph.D.
hCoV-19/USA/AK-PHL8211/2021	EPI_ISL_2153710	4/16/2021	Alaska State Virology Laboratory	Alaska State Virology Laboratory	Stephanie DeRonde, Elva House, Lisa Smith, Ph.D., Jack Chen, Ph.D.
hCoV-19/USA/AK-PHL8214/2021	EPI_ISL_2153711	4/15/2021	Alaska State Virology Laboratory	Alaska State Virology Laboratory	Stephanie DeRonde, Elva House, Lisa Smith, Ph.D., Jack Chen, Ph.D.
hCoV-19/USA/AK-PHL8348/2021	EPI_ISL_2153712	4/22/2021	Alaska State Virology Laboratory	Alaska State Virology Laboratory	Stephanie DeRonde, Elva House, Jacob Zidek, Lisa Smith, Ph.D., Jack Chen, Ph.D.
hCoV-19/USA/AK-PHL8729/2021	EPI_ISL_2154052	5/9/2021	Alaska State Virology Laboratory	Alaska State Virology Laboratory	Stephanie DeRonde, Elva House, Jacob Zidek, Lisa Smith, Ph.D., Jack Chen, Ph.D.
hCoV-19/USA/AK-PHL8730/2021	EPI_ISL_2154053	5/9/2021	Alaska State Virology Laboratory	Alaska State Virology Laboratory	Stephanie DeRonde, Elva House, Jacob Zidek, Lisa Smith, Ph.D., Jack Chen, Ph.D.
hCoV-19/USA/AK-PHL8732/2021	EPI_ISL_2154054	5/9/2021	Alaska State Virology Laboratory	Alaska State Virology Laboratory	Stephanie DeRonde, Elva House, Jacob Zidek, Lisa Smith, Ph.D., Jack Chen, Ph.D.
hCoV-19/USA/AK-PHL8733/2021	EPI_ISL_2154055	5/9/2021	Alaska State Virology Laboratory	Alaska State Virology Laboratory	Stephanie DeRonde, Elva House, Jacob Zidek, Lisa Smith, Ph.D., Jack Chen, Ph.D.
hCoV-19/USA/AK-PHL8734/2021	EPI_ISL_2154056	5/9/2021	Alaska State Virology Laboratory	Alaska State Virology Laboratory	Stephanie DeRonde, Elva House, Jacob Zidek, Lisa Smith, Ph.D., Jack Chen, Ph.D.
hCoV-19/USA/AK-PHL8735/2021	EPI_ISL_2154057	5/8/2021	Alaska State Virology Laboratory	Alaska State Virology Laboratory	Stephanie DeRonde, Elva House, Jacob Zidek, Lisa Smith, Ph.D., Jack Chen, Ph.D.
hCoV-19/USA/AK-PHL8736/2021	EPI_ISL_2154058	5/9/2021	Alaska State Virology Laboratory	Alaska State Virology Laboratory	Stephanie DeRonde, Elva House, Jacob Zidek, Lisa Smith, Ph.D., Jack Chen, Ph.D.
hCoV-19/USA/AK-PHL8739/2021	EPI_ISL_2154059	5/9/2021	Alaska State Virology Laboratory	Alaska State Virology Laboratory	Stephanie DeRonde, Elva House, Jacob Zidek, Lisa Smith, Ph.D., Jack Chen, Ph.D.
hCoV-19/USA/AK-PHL8740/2021	EPI_ISL_2154060	5/9/2021	Alaska State Virology Laboratory	Alaska State Virology Laboratory	Stephanie DeRonde, Elva House, Jacob Zidek, Lisa Smith, Ph.D., Jack Chen, Ph.D.
hCoV-19/USA/AK-PHL8742/2021	EPI_ISL_2154061	5/8/2021	Alaska State Virology Laboratory	Alaska State Virology Laboratory	Stephanie DeRonde, Elva House, Jacob Zidek, Lisa Smith, Ph.D., Jack Chen, Ph.D.
hCoV-19/USA/AK-PHL8743/2021	EPI_ISL_2154062	5/7/2021	Alaska State Virology Laboratory	Alaska State Virology Laboratory	Stephanie DeRonde, Elva House, Jacob Zidek, Lisa Smith, Ph.D., Jack Chen, Ph.D.
hCoV-19/USA/AK-PHL8744/2021	EPI_ISL_2154063	5/7/2021	Alaska State Virology Laboratory	Alaska State Virology Laboratory	Stephanie DeRonde, Elva House, Jacob Zidek, Lisa Smith, Ph.D., Jack Chen, Ph.D.
hCoV-19/USA/AK-PHL8748/2021	EPI_ISL_2154064	5/10/2021	Alaska State Virology Laboratory	Alaska State Virology Laboratory	Stephanie DeRonde, Elva House, Jacob Zidek, Lisa Smith, Ph.D., Jack Chen, Ph.D.
hCoV-19/USA/AK-PHL8749/2021	EPI_ISL_2154065	5/10/2021	Alaska State Virology Laboratory	Alaska State Virology Laboratory	Stephanie DeRonde, Elva House, Jacob Zidek, Lisa Smith, Ph.D., Jack Chen, Ph.D.
hCoV-19/USA/AK-PHL8751/2021	EPI_ISL_2154067	5/10/2021	Alaska State Virology Laboratory	Alaska State Virology Laboratory	Stephanie DeRonde, Elva House, Jacob Zidek, Lisa Smith, Ph.D., Jack Chen, Ph.D.
hCoV-19/USA/AK-PHL8752/2021	EPI_ISL_2154068	5/10/2021	Alaska State Virology Laboratory	Alaska State Virology Laboratory	Stephanie DeRonde, Elva House, Jacob Zidek, Lisa Smith, Ph.D., Jack Chen, Ph.D.
hCoV-19/USA/AK-PHL8753/2021	EPI_ISL_2154069	5/10/2021	Alaska State Virology Laboratory	Alaska State Virology Laboratory	Stephanie DeRonde, Elva House, Jacob Zidek, Lisa Smith, Ph.D., Jack Chen, Ph.D.
hCoV-19/USA/AK-PHL8754/2021	EPI_ISL_2154070	5/10/2021	Alaska State Virology Laboratory	Alaska State Virology Laboratory	Stephanie DeRonde, Elva House, Jacob Zidek, Lisa Smith, Ph.D., Jack Chen, Ph.D.
hCoV-19/USA/AK-PHL8755/2021	EPI_ISL_2154071	5/10/2021	Alaska State Virology Laboratory	Alaska State Virology Laboratory	Stephanie DeRonde, Elva House, Jacob Zidek, Lisa Smith, Ph.D., Jack Chen, Ph.D.
hCoV-19/USA/AK-PHL8757/2021	EPI_ISL_2154072	5/10/2021	Alaska State Virology Laboratory	Alaska State Virology Laboratory	Stephanie DeRonde, Elva House, Jacob Zidek, Lisa Smith, Ph.D., Jack Chen, Ph.D.
hCoV-19/USA/AK-PHL8758/2021	EPI_ISL_2154073	5/10/2021	Alaska State Virology Laboratory	Alaska State Virology Laboratory	Stephanie DeRonde, Elva House, Jacob Zidek, Lisa Smith, Ph.D., Jack Chen, Ph.D.
hCoV-19/USA/AK-PHL8760/2021	EPI_ISL_2154074	5/10/2021	Alaska State Virology Laboratory	Alaska State Virology Laboratory	Stephanie DeRonde, Elva House, Jacob Zidek, Lisa Smith, Ph.D., Jack Chen, Ph.D.
hCoV-19/USA/AK-PHL8761/2021	EPI_ISL_2154075	5/10/2021	Alaska State Virology Laboratory	Alaska State Virology Laboratory	Stephanie DeRonde, Elva House, Jacob Zidek, Lisa Smith, Ph.D., Jack Chen, Ph.D.
hCoV-19/USA/AK-PHL8762/2021	EPI_ISL_2154076	5/10/2021	Alaska State Virology Laboratory	Alaska State Virology Laboratory	Stephanie DeRonde, Elva House, Jacob Zidek, Lisa Smith, Ph.D., Jack Chen, Ph.D.

hCoV-19/USA/AK-PHL8763/2021	EPI_ISL_2154077	5/10/2021	Alaska State Virology Laboratory	Alaska State Virology Laboratory	Stephanie DeRonde, Elva House, Jacob Zidek, Lisa Smith, Ph.D., Jack Chen, Ph.D.
hCoV-19/USA/AK-PHL8764/2021	EPI_ISL_2154078	5/10/2021	Alaska State Virology Laboratory	Alaska State Virology Laboratory	Stephanie DeRonde, Elva House, Jacob Zidek, Lisa Smith, Ph.D., Jack Chen, Ph.D.
hCoV-19/USA/AK-PHL8765/2021	EPI_ISL_2154079	5/10/2021	Alaska State Virology Laboratory	Alaska State Virology Laboratory	Stephanie DeRonde, Elva House, Jacob Zidek, Lisa Smith, Ph.D., Jack Chen, Ph.D.
hCoV-19/USA/AK-PHL8766/2021	EPI_ISL_2154080	5/10/2021	Alaska State Virology Laboratory	Alaska State Virology Laboratory	Stephanie DeRonde, Elva House, Jacob Zidek, Lisa Smith, Ph.D., Jack Chen, Ph.D.
hCoV-19/USA/AK-PHL8767/2021	EPI_ISL_2154081	5/9/2021	Alaska State Virology Laboratory	Alaska State Virology Laboratory	Stephanie DeRonde, Elva House, Jacob Zidek, Lisa Smith, Ph.D., Jack Chen, Ph.D.
hCoV-19/USA/AK-PHL8768/2021	EPI_ISL_2154082	5/10/2021	Alaska State Virology Laboratory	Alaska State Virology Laboratory	Stephanie DeRonde, Elva House, Jacob Zidek, Lisa Smith, Ph.D., Jack Chen, Ph.D.
hCoV-19/USA/AK-PHL8771/2021	EPI_ISL_2154084	5/11/2021	Alaska State Virology Laboratory	Alaska State Virology Laboratory	Stephanie DeRonde, Elva House, Jacob Zidek, Lisa Smith, Ph.D., Jack Chen, Ph.D.
hCoV-19/USA/AK-PHL8772/2021	EPI_ISL_2154085	5/10/2021	Alaska State Virology Laboratory	Alaska State Virology Laboratory	Stephanie DeRonde, Elva House, Jacob Zidek, Lisa Smith, Ph.D., Jack Chen, Ph.D.
hCoV-19/USA/AK-PHL8773/2021	EPI_ISL_2154086	5/11/2021	Alaska State Virology Laboratory	Alaska State Virology Laboratory	Stephanie DeRonde, Elva House, Jacob Zidek, Lisa Smith, Ph.D., Jack Chen, Ph.D.
hCoV-19/USA/AK-PHL8774/2021	EPI_ISL_2154087	5/10/2021	Alaska State Virology Laboratory	Alaska State Virology Laboratory	Stephanie DeRonde, Elva House, Jacob Zidek, Lisa Smith, Ph.D., Jack Chen, Ph.D.
hCoV-19/USA/AK-PHL8689/2021	EPI_ISL_2158264	5/6/2021	Alaska State Virology Laboratory	Alaska State Virology Laboratory	Stephanie DeRonde, Elva House, Jacob Zidek, Lisa Smith, Ph.D., Jack Chen, Ph.D.
hCoV-19/USA/AK-PHL8756/2021	EPI_ISL_2158265	5/10/2021	Alaska State Virology Laboratory	Alaska State Virology Laboratory	Stephanie DeRonde, Elva House, Jacob Zidek, Lisa Smith, Ph.D., Jack Chen, Ph.D.
hCoV-19/USA/AK-PHL8737/2021	EPI_ISL_2158266	5/8/2021	Alaska State Virology Laboratory	Alaska State Virology Laboratory	Stephanie DeRonde, Elva House, Jacob Zidek, Lisa Smith, Ph.D., Jack Chen, Ph.D.
hCoV-19/USA/AK-PHL8759/2021	EPI_ISL_2158267	5/10/2021	Alaska State Virology Laboratory	Alaska State Virology Laboratory	Stephanie DeRonde, Elva House, Jacob Zidek, Lisa Smith, Ph.D., Jack Chen, Ph.D.
hCoV-19/USA/AK-PHL8731/2021	EPI_ISL_2158268	5/9/2021	Alaska State Virology Laboratory	Alaska State Virology Laboratory	Stephanie DeRonde, Elva House, Jacob Zidek, Lisa Smith, Ph.D., Jack Chen, Ph.D.
hCoV-19/USA/AK-CDC-LC0059338/2021	EPI_ISL_2185465	5/9/2021	Laboratory Corporation of America	Centers for Disease Control and Prevention Division of Viral Diseases, Pathogen Discovery	Dakota Howard, Dhwani Batra, Peter W. Cook, Kara Moser, Adrian Paskey, Jason Caravas, Benjamin Rambo-Martin, Shatavia Morrison, Christopher Gulvick, Scott Sammons, Yvette Unoarumhi, Darlene Wagner, Matthew Schmerer, Minoo Agarwal, Eyad Almasri, Debbie Boles, Ayla Burns, Nuthawin Charoensri, Oren Cohen, Susan Countryman, Mary Ann Cristobal, Bobbi Croy, Suzanne Dale, Hrushikesh Deshmukh, Amanda Douglas, Vincent Drouillon, Marcia Eisenberg, Howard Engler, Rama Ghatti, Prashant Gupta, Susan Hicks, Jake Humphrey, Lax Iyer, Manoj Jain, Mohan Kolli, Brian Krueger, Tim Kuphal, Stanley Letovsky, Michael Levandoski, Craig Lukasik, Jonathan Meltzer, Brian Norvell, Mindy Nye, Scott Parker, Christos Petropoulos, John Pruitt, Steven Ragan, Scott Ryan, Mike Sapeta, Jana Schroth, Suresh Babu Selvaraju, Goran Stevovic, Amanda Suchanek, Andrea Throop, Lyndon Tilson, Thomas Urban, Joe Voshell, Kimberly Wagner, Jonathan Williams, Mary Williamson, Qian Zeng, Tricia Zwiefelhofer, Clinton R. Paden, Duncan MacCannell
hCoV-19/USA/AK-CDC-ASC210008123/2021	EPI_ISL_2185980	3/16/2021	Aegis Sciences Corporation	Centers for Disease Control and Prevention Division of Viral Diseases, Pathogen Discovery	Dakota Howard, Dhwani Batra, Peter W. Cook, Kara Moser, Adrian Paskey, Jason Caravas, Benjamin Rambo-Martin, Shatavia Morrison, Christopher Gulvick, Scott Sammons, Yvette Unoarumhi, Darlene Wagner, Matthew Schmerer, Cyndi Clark, Patrick Campbell, Rob Case, Vikramsinha Ghorpade, Holly Houdeshell, Ola Kvalvaag, Dillon Nall, Ethan Sanders, Alec Vest, Shaun Westlund, Matthew Hardison, Clinton R. Paden, Duncan MacCannell
hCoV-19/USA/AK-PHL8777/2021	EPI_ISL_2187646	5/6/2021	Alaska State Virology Laboratory	Alaska State Virology Laboratory	Stephanie DeRonde, Elva House, Jacob Zidek, Lisa Smith, Ph.D., Jack Chen, Ph.D.
hCoV-19/USA/AK-PHL8778/2021	EPI_ISL_2187647	5/6/2021	Alaska State Virology Laboratory	Alaska State Virology Laboratory	Stephanie DeRonde, Elva House, Jacob Zidek, Lisa Smith, Ph.D., Jack Chen, Ph.D.
hCoV-19/USA/AK-PHL8779/2021	EPI_ISL_2187648	5/11/2021	Alaska State Virology Laboratory	Alaska State Virology Laboratory	Stephanie DeRonde, Elva House, Jacob Zidek, Lisa Smith, Ph.D., Jack Chen, Ph.D.
hCoV-19/USA/AK-PHL8780/2021	EPI_ISL_2187649	5/10/2021	Alaska State Virology Laboratory	Alaska State Virology Laboratory	Stephanie DeRonde, Elva House, Jacob Zidek, Lisa Smith, Ph.D., Jack Chen, Ph.D.
hCoV-19/USA/AK-PHL8781/2021	EPI_ISL_2187650	5/11/2021	Alaska State Virology Laboratory	Alaska State Virology Laboratory	Stephanie DeRonde, Elva House, Jacob Zidek, Lisa Smith, Ph.D., Jack Chen, Ph.D.
hCoV-19/USA/AK-PHL8782/2021	EPI_ISL_2187651	5/11/2021	Alaska State Virology Laboratory	Alaska State Virology Laboratory	Stephanie DeRonde, Elva House, Jacob Zidek, Lisa Smith, Ph.D., Jack Chen, Ph.D.
hCoV-19/USA/AK-PHL8783/2021	EPI_ISL_2187652	5/11/2021	Alaska State Virology Laboratory	Alaska State Virology Laboratory	Stephanie DeRonde, Elva House, Jacob Zidek, Lisa Smith, Ph.D., Jack Chen, Ph.D.
hCoV-19/USA/AK-PHL8784/2021	EPI_ISL_2187653	5/11/2021	Alaska State Virology Laboratory	Alaska State Virology Laboratory	Stephanie DeRonde, Elva House, Jacob Zidek, Lisa Smith, Ph.D., Jack Chen, Ph.D.
hCoV-19/USA/AK-PHL8785/2021	EPI_ISL_2187654	5/9/2021	Alaska State Virology Laboratory	Alaska State Virology Laboratory	Stephanie DeRonde, Elva House, Jacob Zidek, Lisa Smith, Ph.D., Jack Chen, Ph.D.
hCoV-19/USA/AK-PHL8786/2021	EPI_ISL_2187655	5/10/2021	Alaska State Virology Laboratory	Alaska State Virology Laboratory	Stephanie DeRonde, Elva House, Jacob Zidek, Lisa Smith, Ph.D., Jack Chen, Ph.D.
hCoV-19/USA/AK-PHL8788/2021	EPI_ISL_2187657	5/10/2021	Alaska State Virology Laboratory	Alaska State Virology Laboratory	Stephanie DeRonde, Elva House, Jacob Zidek, Lisa Smith, Ph.D., Jack Chen, Ph.D.
hCoV-19/USA/AK-PHL8789/2021	EPI_ISL_2187658	5/10/2021	Alaska State Virology Laboratory	Alaska State Virology Laboratory	Stephanie DeRonde, Elva House, Jacob Zidek, Lisa Smith, Ph.D., Jack Chen, Ph.D.
hCoV-19/USA/AK-PHL8790/2021	EPI_ISL_2187659	5/5/2021	Alaska State Virology Laboratory	Alaska State Virology Laboratory	Stephanie DeRonde, Elva House, Jacob Zidek, Lisa Smith, Ph.D., Jack Chen, Ph.D.
hCoV-19/USA/AK-PHL8791/2021	EPI_ISL_2187660	5/4/2021	Alaska State Virology Laboratory	Alaska State Virology Laboratory	Stephanie DeRonde, Elva House, Jacob Zidek, Lisa Smith, Ph.D., Jack Chen, Ph.D.
hCoV-19/USA/AK-PHL8792/2021	EPI_ISL_2187661	5/4/2021	Alaska State Virology Laboratory	Alaska State Virology Laboratory	Stephanie DeRonde, Elva House, Jacob Zidek, Lisa Smith, Ph.D., Jack Chen, Ph.D.
hCoV-19/USA/AK-PHL8793/2021	EPI_ISL_2187662	5/4/2021	Alaska State Virology Laboratory	Alaska State Virology Laboratory	Stephanie DeRonde, Elva House, Jacob Zidek, Lisa Smith, Ph.D., Jack Chen, Ph.D.
hCoV-19/USA/AK-PHL8794/2021	EPI_ISL_2187663	5/4/2021	Alaska State Virology Laboratory	Alaska State Virology Laboratory	Stephanie DeRonde, Elva House, Jacob Zidek, Lisa Smith, Ph.D., Jack Chen, Ph.D.
hCoV-19/USA/AK-PHL8796/2021	EPI_ISL_2187664	5/5/2021	Alaska State Virology Laboratory	Alaska State Virology Laboratory	Stephanie DeRonde, Elva House, Jacob Zidek, Lisa Smith, Ph.D., Jack Chen, Ph.D.

hCoV-19/USA/AK-PHL8797/2021	EPI_ISL_2187665	5/11/2021	Alaska State Virology Laboratory	Alaska State Virology Laboratory	Stephanie DeRonde, Elva House, Jacob Zidek, Lisa Smith, Ph.D., Jack Chen, Ph.D.
hCoV-19/USA/AK-PHL8800/2021	EPI_ISL_2187666	4/26/2021	Alaska State Virology Laboratory	Alaska State Virology Laboratory	Stephanie DeRonde, Elva House, Jacob Zidek, Lisa Smith, Ph.D., Jack Chen, Ph.D.
hCoV-19/USA/AK-PHL8803/2021	EPI_ISL_2187667	5/10/2021	Alaska State Virology Laboratory	Alaska State Virology Laboratory	Stephanie DeRonde, Elva House, Jacob Zidek, Lisa Smith, Ph.D., Jack Chen, Ph.D.
hCoV-19/USA/AK-PHL8804/2021	EPI_ISL_2187668	5/10/2021	Alaska State Virology Laboratory	Alaska State Virology Laboratory	Stephanie DeRonde, Elva House, Jacob Zidek, Lisa Smith, Ph.D., Jack Chen, Ph.D.
hCoV-19/USA/AK-PHL8805/2021	EPI_ISL_2187669	5/10/2021	Alaska State Virology Laboratory	Alaska State Virology Laboratory	Stephanie DeRonde, Elva House, Jacob Zidek, Lisa Smith, Ph.D., Jack Chen, Ph.D.
hCoV-19/USA/AK-PHL8806/2021	EPI_ISL_2187670	5/10/2021	Alaska State Virology Laboratory	Alaska State Virology Laboratory	Stephanie DeRonde, Elva House, Jacob Zidek, Lisa Smith, Ph.D., Jack Chen, Ph.D.
hCoV-19/USA/AK-PHL8807/2021	EPI_ISL_2187671	5/6/2021	Alaska State Virology Laboratory	Alaska State Virology Laboratory	Stephanie DeRonde, Elva House, Jacob Zidek, Lisa Smith, Ph.D., Jack Chen, Ph.D.
hCoV-19/USA/AK-PHL8808/2021	EPI_ISL_2187672	5/6/2021	Alaska State Virology Laboratory	Alaska State Virology Laboratory	Stephanie DeRonde, Elva House, Jacob Zidek, Lisa Smith, Ph.D., Jack Chen, Ph.D.
hCoV-19/USA/AK-PHL8809/2021	EPI_ISL_2187673	5/5/2021	Alaska State Virology Laboratory	Alaska State Virology Laboratory	Stephanie DeRonde, Elva House, Jacob Zidek, Lisa Smith, Ph.D., Jack Chen, Ph.D.
hCoV-19/USA/AK-PHL8811/2021	EPI_ISL_2187675	5/5/2021	Alaska State Virology Laboratory	Alaska State Virology Laboratory	Stephanie DeRonde, Elva House, Jacob Zidek, Lisa Smith, Ph.D., Jack Chen, Ph.D.
hCoV-19/USA/AK-PHL8812/2021	EPI_ISL_2187676	5/8/2021	Alaska State Virology Laboratory	Alaska State Virology Laboratory	Stephanie DeRonde, Elva House, Jacob Zidek, Lisa Smith, Ph.D., Jack Chen, Ph.D.
hCoV-19/USA/AK-PHL8814/2021	EPI_ISL_2187677	5/6/2021	Alaska State Virology Laboratory	Alaska State Virology Laboratory	Stephanie DeRonde, Elva House, Jacob Zidek, Lisa Smith, Ph.D., Jack Chen, Ph.D.
hCoV-19/USA/AK-PHL8815/2021	EPI_ISL_2187678	5/4/2021	Alaska State Virology Laboratory	Alaska State Virology Laboratory	Stephanie DeRonde, Elva House, Jacob Zidek, Lisa Smith, Ph.D., Jack Chen, Ph.D.
hCoV-19/USA/AK-PHL8818/2021	EPI_ISL_2187681	5/1/2021	Alaska State Virology Laboratory	Alaska State Virology Laboratory	Stephanie DeRonde, Elva House, Jacob Zidek, Lisa Smith, Ph.D., Jack Chen, Ph.D.
hCoV-19/USA/AK-PHL8819/2021	EPI_ISL_2187682	5/2/2021	Alaska State Virology Laboratory	Alaska State Virology Laboratory	Stephanie DeRonde, Elva House, Jacob Zidek, Lisa Smith, Ph.D., Jack Chen, Ph.D.
hCoV-19/USA/AK-PHL8820/2021	EPI_ISL_2187683	4/29/2021	Alaska State Virology Laboratory	Alaska State Virology Laboratory	Stephanie DeRonde, Elva House, Jacob Zidek, Lisa Smith, Ph.D., Jack Chen, Ph.D.
hCoV-19/USA/AK-CDC-ASC210073436/2021	EPI_ISL_2202659	4/15/2021	Aegis Sciences Corporation	Centers for Disease Control and Prevention Division of Viral Diseases, Pathogen Discovery	Dakota Howard, Dhwani Batra, Peter W. Cook, Kara Moser, Adrian Paskey, Jason Caravas, Benjamin Rambo-Martin, Shatavia Morrison, Christopher Gulvick, Scott Sammons, Yvette Unoarumhi, Darlene Wagner, Matthew Schmerer, Cyndi Clark, Patrick Campbell, Rob Case, Vikramsinha Ghorpade, Holly Houdeshell, Ola Kvalvaag, Dillon Nall, Ethan Sanders, Alec Vest, Shaun Westlund, Matthew Hardison, Clinton R. Paden, Duncan MacCannell
hCoV-19/USA/AK-PHL8637/2021	EPI_ISL_2213152	4/26/2021	Alaska State Virology Laboratory	Alaska State Virology Laboratory	Stephanie DeRonde, Elva House, Jacob Zidek, Lisa Smith, Ph.D., Jack Chen, Ph.D.
hCoV-19/USA/AK-PHL8639/2021	EPI_ISL_2213154	4/29/2021	Alaska State Virology Laboratory	Alaska State Virology Laboratory	Stephanie DeRonde, Elva House, Jacob Zidek, Lisa Smith, Ph.D., Jack Chen, Ph.D.
hCoV-19/USA/AK-PHL8640/2021	EPI_ISL_2213157	5/1/2021	Alaska State Virology Laboratory	Alaska State Virology Laboratory	Stephanie DeRonde, Elva House, Jacob Zidek, Lisa Smith, Ph.D., Jack Chen, Ph.D.
hCoV-19/USA/AK-PHL8641/2021	EPI_ISL_2213159	5/1/2021	Alaska State Virology Laboratory	Alaska State Virology Laboratory	Stephanie DeRonde, Elva House, Jacob Zidek, Lisa Smith, Ph.D., Jack Chen, Ph.D.
hCoV-19/USA/AK-PHL8642/2021	EPI_ISL_2213162	5/3/2021	Alaska State Virology Laboratory	Alaska State Virology Laboratory	Stephanie DeRonde, Elva House, Jacob Zidek, Lisa Smith, Ph.D., Jack Chen, Ph.D.
hCoV-19/USA/AK-PHL8643/2021	EPI_ISL_2213165	5/4/2021	Alaska State Virology Laboratory	Alaska State Virology Laboratory	Stephanie DeRonde, Elva House, Jacob Zidek, Lisa Smith, Ph.D., Jack Chen, Ph.D.
hCoV-19/USA/AK-PHL8644/2021	EPI_ISL_2213167	5/4/2021	Alaska State Virology Laboratory	Alaska State Virology Laboratory	Stephanie DeRonde, Elva House, Jacob Zidek, Lisa Smith, Ph.D., Jack Chen, Ph.D.
hCoV-19/USA/AK-PHL8647/2021	EPI_ISL_2213172	5/4/2021	Alaska State Virology Laboratory	Alaska State Virology Laboratory	Stephanie DeRonde, Elva House, Jacob Zidek, Lisa Smith, Ph.D., Jack Chen, Ph.D.
hCoV-19/USA/AK-PHL8648/2021	EPI_ISL_2213174	5/7/2021	Alaska State Virology Laboratory	Alaska State Virology Laboratory	Stephanie DeRonde, Elva House, Jacob Zidek, Lisa Smith, Ph.D., Jack Chen, Ph.D.
hCoV-19/USA/AK-PHL8650/2021	EPI_ISL_2213176	5/4/2021	Alaska State Virology Laboratory	Alaska State Virology Laboratory	Stephanie DeRonde, Elva House, Jacob Zidek, Lisa Smith, Ph.D., Jack Chen, Ph.D.
hCoV-19/USA/AK-PHL8651/2021	EPI_ISL_2213179	5/4/2021	Alaska State Virology Laboratory	Alaska State Virology Laboratory	Stephanie DeRonde, Elva House, Jacob Zidek, Lisa Smith, Ph.D., Jack Chen, Ph.D.
hCoV-19/USA/AK-PHL8652/2021	EPI_ISL_2213181	5/5/2021	Alaska State Virology Laboratory	Alaska State Virology Laboratory	Stephanie DeRonde, Elva House, Jacob Zidek, Lisa Smith, Ph.D., Jack Chen, Ph.D.
hCoV-19/USA/AK-PHL8653/2021	EPI_ISL_2213184	5/5/2021	Alaska State Virology Laboratory	Alaska State Virology Laboratory	Stephanie DeRonde, Elva House, Jacob Zidek, Lisa Smith, Ph.D., Jack Chen, Ph.D.
hCoV-19/USA/AK-PHL8654/2021	EPI_ISL_2213186	5/7/2021	Alaska State Virology Laboratory	Alaska State Virology Laboratory	Stephanie DeRonde, Elva House, Jacob Zidek, Lisa Smith, Ph.D., Jack Chen, Ph.D.
hCoV-19/USA/AK-PHL8655/2021	EPI_ISL_2213189	5/7/2021	Alaska State Virology Laboratory	Alaska State Virology Laboratory	Stephanie DeRonde, Elva House, Jacob Zidek, Lisa Smith, Ph.D., Jack Chen, Ph.D.
hCoV-19/USA/AK-PHL8656/2021	EPI_ISL_2213191	5/7/2021	Alaska State Virology Laboratory	Alaska State Virology Laboratory	Stephanie DeRonde, Elva House, Jacob Zidek, Lisa Smith, Ph.D., Jack Chen, Ph.D.
hCoV-19/USA/AK-PHL8657/2021	EPI_ISL_2213194	5/6/2021	Alaska State Virology Laboratory	Alaska State Virology Laboratory	Stephanie DeRonde, Elva House, Jacob Zidek, Lisa Smith, Ph.D., Jack Chen, Ph.D.
hCoV-19/USA/AK-PHL8660/2021	EPI_ISL_2213196	5/5/2021	Alaska State Virology Laboratory	Alaska State Virology Laboratory	Stephanie DeRonde, Elva House, Jacob Zidek, Lisa Smith, Ph.D., Jack Chen, Ph.D.
hCoV-19/USA/AK-PHL8663/2021	EPI_ISL_2213199	5/3/2021	Alaska State Virology Laboratory	Alaska State Virology Laboratory	Stephanie DeRonde, Elva House, Jacob Zidek, Lisa Smith, Ph.D., Jack Chen, Ph.D.
hCoV-19/USA/AK-PHL8665/2021	EPI_ISL_2213203	5/3/2021	Alaska State Virology Laboratory	Alaska State Virology Laboratory	Stephanie DeRonde, Elva House, Jacob Zidek, Lisa Smith, Ph.D., Jack Chen, Ph.D.
hCoV-19/USA/AK-PHL8666/2021	EPI_ISL_2213204	4/23/2021	Alaska State Virology Laboratory	Alaska State Virology Laboratory	Stephanie DeRonde, Elva House, Jacob Zidek, Lisa Smith, Ph.D., Jack Chen, Ph.D.
hCoV-19/USA/AK-PHL8667/2021	EPI_ISL_2213207	4/19/2021	Alaska State Virology Laboratory	Alaska State Virology Laboratory	Stephanie DeRonde, Elva House, Jacob Zidek, Lisa Smith, Ph.D., Jack Chen, Ph.D.
hCoV-19/USA/AK-PHL8669/2021	EPI_ISL_2213210	5/4/2021	Alaska State Virology Laboratory	Alaska State Virology Laboratory	Stephanie DeRonde, Elva House, Jacob Zidek, Lisa Smith, Ph.D., Jack Chen, Ph.D.
hCoV-19/USA/AK-PHL8671/2021	EPI_ISL_2213212	5/1/2021	Alaska State Virology Laboratory	Alaska State Virology Laboratory	Stephanie DeRonde, Elva House, Jacob Zidek, Lisa Smith, Ph.D., Jack Chen, Ph.D.
hCoV-19/USA/AK-PHL8672/2021	EPI_ISL_2213215	4/30/2021	Alaska State Virology Laboratory	Alaska State Virology Laboratory	Stephanie DeRonde, Elva House, Jacob Zidek, Lisa Smith, Ph.D., Jack Chen, Ph.D.
hCoV-19/USA/AK-PHL8673/2021	EPI_ISL_2213216	4/29/2021	Alaska State Virology Laboratory	Alaska State Virology Laboratory	Stephanie DeRonde, Elva House, Jacob Zidek, Lisa Smith, Ph.D., Jack Chen, Ph.D.
hCoV-19/USA/AK-PHL8675/2021	EPI_ISL_2213221	5/6/2021	Alaska State Virology Laboratory	Alaska State Virology Laboratory	Stephanie DeRonde, Elva House, Jacob Zidek, Lisa Smith, Ph.D., Jack Chen, Ph.D.
hCoV-19/USA/AK-PHL8677/2021	EPI_ISL_2213224	5/6/2021	Alaska State Virology Laboratory	Alaska State Virology Laboratory	Stephanie DeRonde, Elva House, Jacob Zidek, Lisa Smith, Ph.D., Jack Chen, Ph.D.
hCoV-19/USA/AK-PHL8678/2021	EPI_ISL_2213225	5/6/2021	Alaska State Virology Laboratory	Alaska State Virology Laboratory	Stephanie DeRonde, Elva House, Jacob Zidek, Lisa Smith, Ph.D., Jack Chen, Ph.D.

hCoV-19/USA/AK-PHL8679/2021	EPI_ISL_2213228	5/5/2021	Alaska State Virology Laboratory	Alaska State Virology Laboratory	Stephanie DeRonde, Elva House, Jacob Zidek, Lisa Smith, Ph.D., Jack Chen, Ph.D.
hCoV-19/USA/AK-PHL8680/2021	EPI_ISL_2213231	5/5/2021	Alaska State Virology Laboratory	Alaska State Virology Laboratory	Stephanie DeRonde, Elva House, Jacob Zidek, Lisa Smith, Ph.D., Jack Chen, Ph.D.
hCoV-19/USA/AK-PHL8681/2021	EPI_ISL_2213232	5/5/2021	Alaska State Virology Laboratory	Alaska State Virology Laboratory	Stephanie DeRonde, Elva House, Jacob Zidek, Lisa Smith, Ph.D., Jack Chen, Ph.D.
hCoV-19/USA/AK-PHL8682/2021	EPI_ISL_2213235	5/5/2021	Alaska State Virology Laboratory	Alaska State Virology Laboratory	Stephanie DeRonde, Elva House, Jacob Zidek, Lisa Smith, Ph.D., Jack Chen, Ph.D.
hCoV-19/USA/AK-PHL7919/2021	EPI_ISL_2227457	4/3/2021	Alaska State Virology Laboratory	Alaska State Virology Laboratory	Stephanie DeRonde, Elva House, Lisa Smith, Ph.D., Jack Chen, Ph.D.
hCoV-19/USA/AK-PHL7923/2021	EPI_ISL_2227459	4/3/2021	Alaska State Virology Laboratory	Alaska State Virology Laboratory	Stephanie DeRonde, Elva House, Lisa Smith, Ph.D., Jack Chen, Ph.D.
hCoV-19/USA/AK-PHL8649/2021	EPI_ISL_2227462	5/5/2021	Alaska State Virology Laboratory	Alaska State Virology Laboratory	Stephanie DeRonde, Elva House, Jacob Zidek, Lisa Smith, Ph.D., Jack Chen, Ph.D.
hCoV-19/USA/AK-PHL8661/2021	EPI_ISL_2227463	5/4/2021	Alaska State Virology Laboratory	Alaska State Virology Laboratory	Stephanie DeRonde, Elva House, Jacob Zidek, Lisa Smith, Ph.D., Jack Chen, Ph.D.
hCoV-19/USA/AK-CDC-2-4356403/2021	EPI_ISL_2229014	5/1/2021	AK State Public Health Lab, State Health Department	Centers for Disease Control and Prevention Division of Viral Diseases, Pathogen Discovery	Mili Sheth, Sarah Nobles, Jasmine Padilla, Mark Burroughs, Shoshona Le, Katie Dillon, Peter Cook, Clinton R. Paden, Dhwani Batra, Krista Queen, Kristen Knipe, Dakota Howard, Yvette Unoarumhi, Darlene Wagner, Matthew Schmerer, Ben L. Rambo-Martin, Kristine Lacek, Sam Shepard, Alison Laufer Halpin, Dave Wentworth, Vivien Dugan, Suxiang Tong, Justin Lee
hCoV-19/USA/AK-CDC-2-4356404/2021	EPI_ISL_2229017	4/26/2021	AK State Public Health Lab, State Health Department	Centers for Disease Control and Prevention Division of Viral Diseases, Pathogen Discovery	Mili Sheth, Sarah Nobles, Jasmine Padilla, Mark Burroughs, Shoshona Le, Katie Dillon, Peter Cook, Clinton R. Paden, Dhwani Batra, Krista Queen, Kristen Knipe, Dakota Howard, Yvette Unoarumhi, Darlene Wagner, Matthew Schmerer, Ben L. Rambo-Martin, Kristine Lacek, Sam Shepard, Alison Laufer Halpin, Dave Wentworth, Vivien Dugan, Suxiang Tong, Justin Lee
hCoV-19/USA/AK-CDC-2-4356434/2021	EPI_ISL_2229020	5/3/2021	AK State Public Health Lab, State Health Department	Centers for Disease Control and Prevention Division of Viral Diseases, Pathogen Discovery	Mili Sheth, Sarah Nobles, Jasmine Padilla, Mark Burroughs, Shoshona Le, Katie Dillon, Peter Cook, Clinton R. Paden, Dhwani Batra, Krista Queen, Kristen Knipe, Dakota Howard, Yvette Unoarumhi, Darlene Wagner, Matthew Schmerer, Ben L. Rambo-Martin, Kristine Lacek, Sam Shepard, Alison Laufer Halpin, Dave Wentworth, Vivien Dugan, Suxiang Tong, Justin Lee
hCoV-19/USA/AK-CDC-2-4356446/2021	EPI_ISL_2229022	5/5/2021	AK State Public Health Lab, State Health Department	Centers for Disease Control and Prevention Division of Viral Diseases, Pathogen Discovery	Mili Sheth, Sarah Nobles, Jasmine Padilla, Mark Burroughs, Shoshona Le, Katie Dillon, Peter Cook, Clinton R. Paden, Dhwani Batra, Krista Queen, Kristen Knipe, Dakota Howard, Yvette Unoarumhi, Darlene Wagner, Matthew Schmerer, Ben L. Rambo-Martin, Kristine Lacek, Sam Shepard, Alison Laufer Halpin, Dave Wentworth, Vivien Dugan, Suxiang Tong, Justin Lee
hCoV-19/USA/AK-CDC-2-4356511/2021	EPI_ISL_2229025	5/3/2021	AK State Public Health Lab, State Health Department	Centers for Disease Control and Prevention Division of Viral Diseases, Pathogen Discovery	Mili Sheth, Sarah Nobles, Jasmine Padilla, Mark Burroughs, Shoshona Le, Katie Dillon, Peter Cook, Clinton R. Paden, Dhwani Batra, Krista Queen, Kristen Knipe, Dakota Howard, Yvette Unoarumhi, Darlene Wagner, Matthew Schmerer, Ben L. Rambo-Martin, Kristine Lacek, Sam Shepard, Alison Laufer Halpin, Dave Wentworth, Vivien Dugan, Suxiang Tong, Justin Lee
hCoV-19/USA/AK-CDC-2-4356422/2021	EPI_ISL_2229027	4/26/2021	AK State Public Health Lab, State Health Department	Centers for Disease Control and Prevention Division of Viral Diseases, Pathogen Discovery	Mili Sheth, Sarah Nobles, Jasmine Padilla, Mark Burroughs, Shoshona Le, Katie Dillon, Peter Cook, Clinton R. Paden, Dhwani Batra, Krista Queen, Kristen Knipe, Dakota Howard, Yvette Unoarumhi, Darlene Wagner, Matthew Schmerer, Ben L. Rambo-Martin, Kristine Lacek, Sam Shepard, Alison Laufer Halpin, Dave Wentworth, Vivien Dugan, Suxiang Tong, Justin Lee
hCoV-19/USA/AK-CDC-2-4356530/2021	EPI_ISL_2229030	4/26/2021	AK State Public Health Lab, State Health Department	Centers for Disease Control and Prevention Division of Viral Diseases, Pathogen Discovery	Mili Sheth, Sarah Nobles, Jasmine Padilla, Mark Burroughs, Shoshona Le, Katie Dillon, Peter Cook, Clinton R. Paden, Dhwani Batra, Krista Queen, Kristen Knipe, Dakota Howard, Yvette Unoarumhi, Darlene Wagner, Matthew Schmerer, Ben L. Rambo-Martin, Kristine Lacek, Sam Shepard, Alison Laufer Halpin, Dave Wentworth, Vivien Dugan, Suxiang Tong, Justin Lee
hCoV-19/USA/AK-CDC-2-4356522/2021	EPI_ISL_2229032	4/27/2021	AK State Public Health Lab, State Health Department	Centers for Disease Control and Prevention Division of Viral Diseases, Pathogen Discovery	Mili Sheth, Sarah Nobles, Jasmine Padilla, Mark Burroughs, Shoshona Le, Katie Dillon, Peter Cook, Clinton R. Paden, Dhwani Batra, Krista Queen, Kristen Knipe, Dakota Howard, Yvette Unoarumhi, Darlene Wagner, Matthew Schmerer, Ben L. Rambo-Martin, Kristine Lacek, Sam Shepard, Alison Laufer Halpin, Dave Wentworth, Vivien Dugan, Suxiang Tong, Justin Lee
hCoV-19/USA/AK-CDC-2-4356414/2021	EPI_ISL_2229034	5/3/2021	AK State Public Health Lab, State Health Department	Centers for Disease Control and Prevention Division of Viral Diseases, Pathogen Discovery	Mili Sheth, Sarah Nobles, Jasmine Padilla, Mark Burroughs, Shoshona Le, Katie Dillon, Peter Cook, Clinton R. Paden, Dhwani Batra, Krista Queen, Kristen Knipe, Dakota Howard, Yvette Unoarumhi, Darlene Wagner, Matthew Schmerer, Ben L. Rambo-Martin, Kristine Lacek, Sam Shepard, Alison Laufer Halpin, Dave Wentworth, Vivien Dugan, Suxiang Tong, Justin Lee

hCoV-19/USA/AK-CDC-2-4356544/2021	EPI_ISL_2229036	5/4/2021	AK State Public Health Lab, State Health Department	Centers for Disease Control and Prevention Division of Viral Diseases, Pathogen Discovery	Mili Sheth, Sarah Nobles, Jasmine Padilla, Mark Burroughs, Shoshona Le, Katie Dillon, Peter Cook, Clinton R. Paden, Dhwani Batra, Krista Queen, Kristen Knipe, Dakota Howard, Yvette Unoarumhi, Darlene Wagner, Matthew Schmerer, Ben L. Rambo-Martin, Kristine Lacek, Sam Shepard, Alison Laufer Halpin, Dave Wentworth, Vivien Dugan, Suxiang Tong, Justin Lee
hCoV-19/USA/AK-CDC-2-4356433/2021	EPI_ISL_2229039	4/26/2021	AK State Public Health Lab, State Health Department	Centers for Disease Control and Prevention Division of Viral Diseases, Pathogen Discovery	Mili Sheth, Sarah Nobles, Jasmine Padilla, Mark Burroughs, Shoshona Le, Katie Dillon, Peter Cook, Clinton R. Paden, Dhwani Batra, Krista Queen, Kristen Knipe, Dakota Howard, Yvette Unoarumhi, Darlene Wagner, Matthew Schmerer, Ben L. Rambo-Martin, Kristine Lacek, Sam Shepard, Alison Laufer Halpin, Dave Wentworth, Vivien Dugan, Suxiang Tong, Justin Lee
hCoV-19/USA/AK-CDC-2-4356443/2021	EPI_ISL_2229042	5/4/2021	AK State Public Health Lab, State Health Department	Centers for Disease Control and Prevention Division of Viral Diseases, Pathogen Discovery	Mili Sheth, Sarah Nobles, Jasmine Padilla, Mark Burroughs, Shoshona Le, Katie Dillon, Peter Cook, Clinton R. Paden, Dhwani Batra, Krista Queen, Kristen Knipe, Dakota Howard, Yvette Unoarumhi, Darlene Wagner, Matthew Schmerer, Ben L. Rambo-Martin, Kristine Lacek, Sam Shepard, Alison Laufer Halpin, Dave Wentworth, Vivien Dugan, Suxiang Tong, Justin Lee
hCoV-19/USA/AK-CDC-2-4356501/2021	EPI_ISL_2229047	5/3/2021	AK State Public Health Lab, State Health Department	Centers for Disease Control and Prevention Division of Viral Diseases, Pathogen Discovery	Mili Sheth, Sarah Nobles, Jasmine Padilla, Mark Burroughs, Shoshona Le, Katie Dillon, Peter Cook, Clinton R. Paden, Dhwani Batra, Krista Queen, Kristen Knipe, Dakota Howard, Yvette Unoarumhi, Darlene Wagner, Matthew Schmerer, Ben L. Rambo-Martin, Kristine Lacek, Sam Shepard, Alison Laufer Halpin, Dave Wentworth, Vivien Dugan, Suxiang Tong, Justin Lee
hCoV-19/USA/AK-CDC-2-4356523/2021	EPI_ISL_2229050	4/26/2021	AK State Public Health Lab, State Health Department	Centers for Disease Control and Prevention Division of Viral Diseases, Pathogen Discovery	Mili Sheth, Sarah Nobles, Jasmine Padilla, Mark Burroughs, Shoshona Le, Katie Dillon, Peter Cook, Clinton R. Paden, Dhwani Batra, Krista Queen, Kristen Knipe, Dakota Howard, Yvette Unoarumhi, Darlene Wagner, Matthew Schmerer, Ben L. Rambo-Martin, Kristine Lacek, Sam Shepard, Alison Laufer Halpin, Dave Wentworth, Vivien Dugan, Suxiang Tong, Justin Lee
hCoV-19/USA/AK-CDC-2-4356449/2021	EPI_ISL_2229052	5/3/2021	AK State Public Health Lab, State Health Department	Centers for Disease Control and Prevention Division of Viral Diseases, Pathogen Discovery	Mili Sheth, Sarah Nobles, Jasmine Padilla, Mark Burroughs, Shoshona Le, Katie Dillon, Peter Cook, Clinton R. Paden, Dhwani Batra, Krista Queen, Kristen Knipe, Dakota Howard, Yvette Unoarumhi, Darlene Wagner, Matthew Schmerer, Ben L. Rambo-Martin, Kristine Lacek, Sam Shepard, Alison Laufer Halpin, Dave Wentworth, Vivien Dugan, Suxiang Tong, Justin Lee
hCoV-19/USA/AK-CDC-2-4356425/2021	EPI_ISL_2229054	4/27/2021	AK State Public Health Lab, State Health Department	Centers for Disease Control and Prevention Division of Viral Diseases, Pathogen Discovery	Mili Sheth, Sarah Nobles, Jasmine Padilla, Mark Burroughs, Shoshona Le, Katie Dillon, Peter Cook, Clinton R. Paden, Dhwani Batra, Krista Queen, Kristen Knipe, Dakota Howard, Yvette Unoarumhi, Darlene Wagner, Matthew Schmerer, Ben L. Rambo-Martin, Kristine Lacek, Sam Shepard, Alison Laufer Halpin, Dave Wentworth, Vivien Dugan, Suxiang Tong, Justin Lee
hCoV-19/USA/AK-PHL7917/2021	EPI_ISL_2230701	4/3/2021	Alaska State Virology Laboratory	Alaska State Virology Laboratory	Stephanie DeRonde, Elva House, Lisa Smith, Ph.D., Jack Chen, Ph.D.
hCoV-19/USA/AK-PHL7792/2021	EPI_ISL_2230703	3/26/2021	Alaska State Virology Laboratory	Alaska State Virology Laboratory	Stephanie DeRonde, Elva House, Lisa Smith, Ph.D., Jack Chen, Ph.D.
hCoV-19/USA/AK-PHL7804/2021	EPI_ISL_2230704	3/29/2021	Alaska State Virology Laboratory	Alaska State Virology Laboratory	Stephanie DeRonde, Elva House, Lisa Smith, Ph.D., Jack Chen, Ph.D.
hCoV-19/USA/AK-PHL7764/2021	EPI_ISL_2230705	3/26/2021	Alaska State Virology Laboratory	Alaska State Virology Laboratory	Stephanie DeRonde, Elva House, Lisa Smith, Ph.D., Jack Chen, Ph.D.
hCoV-19/USA/AK-CDC-ASC210073437/2021	EPI_ISL_2268080	4/15/2021	Aegis Sciences Corporation	Centers for Disease Control and Prevention Division of Viral Diseases, Pathogen Discovery	Dakota Howard, Dhwani Batra, Peter W. Cook, Kara Moser, Adrian Paskey, Jason Caravas, Benjamin Rambo-Martin, Shatavia Morrison, Christopher Gulvick, Scott Sammons, Yvette Unoarumhi, Darlene Wagner, Matthew Schmerer, Cyndi Clark, Patrick Campbell, Rob Case, Vikramsinha Ghorpade, Holly Houdeshell, Ola Kvalvaag, Dillon Nall, Ethan Sanders, Alec Vest, Shaun Westlund, Matthew Hardison, Clinton R. Paden, Duncan MacCannell
hCoV-19/USA/AK-CDC-STM-000070167/2021	EPI_ISL_2270401	5/6/2021	Helix/Illumina	Centers for Disease Control and Prevention Division of Viral Diseases, Pathogen Discovery	Dakota Howard, Dhwani Batra, Peter W. Cook, Kara Moser, Adrian Paskey, Jason Caravas, Benjamin Rambo-Martin, Shatavia Morrison, Christopher Gulvick, Scott Sammons, Yvette Unoarumhi, Darlene Wagner, Matthew Schmerer, Eileen de Feo, Jan Antico, Christine Tran, Matthew Tolentino, Shannon Wickline, Kim Gietzen, Brad Sickler, Jingtao Liu, Eric Allen, Phil Febbo, Nicole L. Washington, Simon White, Geraint Levan, Kelly Schiabor Barrett, Elizabeth Cirulli, Alexandre Bolze, Ary Ascencio, Charlotte Rivera-Garcia, Ryan Cho, Jason Nguyen, Sherry Wang, Jimmy Ramirez, Tyler Cassens, Efren Sandoval, Magnus Isaksson, William Lee, David Becker, Marc Laurent, James Lu, Clinton R. Paden, Duncan MacCannell
hCoV-19/USA/AK-PHL8823/2021	EPI_ISL_2272522	5/14/2021	Alaska State Virology Laboratory	Alaska State Virology Laboratory	Stephanie DeRonde, Elva House, Jacob Zidek, Lisa Smith, Ph.D., Jack Chen, Ph.D.

hCoV-19/USA/AK-PHL8824/2021	EPI_ISL_2272523	5/12/2021	Alaska State Virology Laboratory	Alaska State Virology Laboratory	Stephanie DeRonde, Elva House, Jacob Zidek, Lisa Smith, Ph.D., Jack Chen, Ph.D.
hCoV-19/USA/AK-PHL8826/2021	EPI_ISL_2272524	5/10/2021	Alaska State Virology Laboratory	Alaska State Virology Laboratory	Stephanie DeRonde, Elva House, Jacob Zidek, Lisa Smith, Ph.D., Jack Chen, Ph.D.
hCoV-19/USA/AK-PHL8829/2021	EPI_ISL_2272526	5/12/2021	Alaska State Virology Laboratory	Alaska State Virology Laboratory	Stephanie DeRonde, Elva House, Jacob Zidek, Lisa Smith, Ph.D., Jack Chen, Ph.D.
hCoV-19/USA/AK-PHL8831/2021	EPI_ISL_2272527	5/14/2021	Alaska State Virology Laboratory	Alaska State Virology Laboratory	Stephanie DeRonde, Elva House, Jacob Zidek, Lisa Smith, Ph.D., Jack Chen, Ph.D.
hCoV-19/USA/AK-PHL8832/2021	EPI_ISL_2272528	5/14/2021	Alaska State Virology Laboratory	Alaska State Virology Laboratory	Stephanie DeRonde, Elva House, Jacob Zidek, Lisa Smith, Ph.D., Jack Chen, Ph.D.
hCoV-19/USA/AK-PHL8833/2021	EPI_ISL_2272529	5/12/2021	Alaska State Virology Laboratory	Alaska State Virology Laboratory	Stephanie DeRonde, Elva House, Jacob Zidek, Lisa Smith, Ph.D., Jack Chen, Ph.D.
hCoV-19/USA/AK-PHL8834/2021	EPI_ISL_2272530	5/13/2021	Alaska State Virology Laboratory	Alaska State Virology Laboratory	Stephanie DeRonde, Elva House, Jacob Zidek, Lisa Smith, Ph.D., Jack Chen, Ph.D.
hCoV-19/USA/AK-PHL8837/2021	EPI_ISL_2272532	5/12/2021	Alaska State Virology Laboratory	Alaska State Virology Laboratory	Stephanie DeRonde, Elva House, Jacob Zidek, Lisa Smith, Ph.D., Jack Chen, Ph.D.
hCoV-19/USA/AK-PHL8838/2021	EPI_ISL_2272533	5/12/2021	Alaska State Virology Laboratory	Alaska State Virology Laboratory	Stephanie DeRonde, Elva House, Jacob Zidek, Lisa Smith, Ph.D., Jack Chen, Ph.D.
hCoV-19/USA/AK-PHL8839/2021	EPI_ISL_2272534	5/12/2021	Alaska State Virology Laboratory	Alaska State Virology Laboratory	Stephanie DeRonde, Elva House, Jacob Zidek, Lisa Smith, Ph.D., Jack Chen, Ph.D.
hCoV-19/USA/AK-PHL8840/2021	EPI_ISL_2272535	5/11/2021	Alaska State Virology Laboratory	Alaska State Virology Laboratory	Stephanie DeRonde, Elva House, Jacob Zidek, Lisa Smith, Ph.D., Jack Chen, Ph.D.
hCoV-19/USA/AK-PHL8842/2021	EPI_ISL_2272536	5/12/2021	Alaska State Virology Laboratory	Alaska State Virology Laboratory	Stephanie DeRonde, Elva House, Jacob Zidek, Lisa Smith, Ph.D., Jack Chen, Ph.D.
hCoV-19/USA/AK-PHL8843/2021	EPI_ISL_2272537	5/12/2021	Alaska State Virology Laboratory	Alaska State Virology Laboratory	Stephanie DeRonde, Elva House, Jacob Zidek, Lisa Smith, Ph.D., Jack Chen, Ph.D.
hCoV-19/USA/AK-PHL8844/2021	EPI_ISL_2272538	5/12/2021	Alaska State Virology Laboratory	Alaska State Virology Laboratory	Stephanie DeRonde, Elva House, Jacob Zidek, Lisa Smith, Ph.D., Jack Chen, Ph.D.
hCoV-19/USA/AK-PHL8846/2021	EPI_ISL_2272540	5/12/2021	Alaska State Virology Laboratory	Alaska State Virology Laboratory	Stephanie DeRonde, Elva House, Jacob Zidek, Lisa Smith, Ph.D., Jack Chen, Ph.D.
hCoV-19/USA/AK-PHL8848/2021	EPI_ISL_2272541	5/12/2021	Alaska State Virology Laboratory	Alaska State Virology Laboratory	Stephanie DeRonde, Elva House, Jacob Zidek, Lisa Smith, Ph.D., Jack Chen, Ph.D.
hCoV-19/USA/AK-PHL8849/2021	EPI_ISL_2272542	5/12/2021	Alaska State Virology Laboratory	Alaska State Virology Laboratory	Stephanie DeRonde, Elva House, Jacob Zidek, Lisa Smith, Ph.D., Jack Chen, Ph.D.
hCoV-19/USA/AK-PHL8850/2021	EPI_ISL_2272543	5/11/2021	Alaska State Virology Laboratory	Alaska State Virology Laboratory	Stephanie DeRonde, Elva House, Jacob Zidek, Lisa Smith, Ph.D., Jack Chen, Ph.D.
hCoV-19/USA/AK-PHL8854/2021	EPI_ISL_2272546	5/12/2021	Alaska State Virology Laboratory	Alaska State Virology Laboratory	Stephanie DeRonde, Elva House, Jacob Zidek, Lisa Smith, Ph.D., Jack Chen, Ph.D.
hCoV-19/USA/AK-PHL8855/2021	EPI_ISL_2272547	5/15/2021	Alaska State Virology Laboratory	Alaska State Virology Laboratory	Stephanie DeRonde, Elva House, Jacob Zidek, Lisa Smith, Ph.D., Jack Chen, Ph.D.
hCoV-19/USA/AK-PHL8856/2021	EPI_ISL_2272548	5/15/2021	Alaska State Virology Laboratory	Alaska State Virology Laboratory	Stephanie DeRonde, Elva House, Jacob Zidek, Lisa Smith, Ph.D., Jack Chen, Ph.D.
hCoV-19/USA/AK-PHL8857/2021	EPI_ISL_2272549	5/15/2021	Alaska State Virology Laboratory	Alaska State Virology Laboratory	Stephanie DeRonde, Elva House, Jacob Zidek, Lisa Smith, Ph.D., Jack Chen, Ph.D.
hCoV-19/USA/AK-PHL8858/2021	EPI_ISL_2272550	5/14/2021	Alaska State Virology Laboratory	Alaska State Virology Laboratory	Stephanie DeRonde, Elva House, Jacob Zidek, Lisa Smith, Ph.D., Jack Chen, Ph.D.
hCoV-19/USA/AK-PHL8859/2021	EPI_ISL_2272551	5/15/2021	Alaska State Virology Laboratory	Alaska State Virology Laboratory	Stephanie DeRonde, Elva House, Jacob Zidek, Lisa Smith, Ph.D., Jack Chen, Ph.D.
hCoV-19/USA/AK-PHL8861/2021	EPI_ISL_2272552	5/15/2021	Alaska State Virology Laboratory	Alaska State Virology Laboratory	Stephanie DeRonde, Elva House, Jacob Zidek, Lisa Smith, Ph.D., Jack Chen, Ph.D.
hCoV-19/USA/AK-PHL8862/2021	EPI_ISL_2272553	5/15/2021	Alaska State Virology Laboratory	Alaska State Virology Laboratory	Stephanie DeRonde, Elva House, Jacob Zidek, Lisa Smith, Ph.D., Jack Chen, Ph.D.
hCoV-19/USA/AK-PHL8863/2021	EPI_ISL_2272554	5/15/2021	Alaska State Virology Laboratory	Alaska State Virology Laboratory	Stephanie DeRonde, Elva House, Jacob Zidek, Lisa Smith, Ph.D., Jack Chen, Ph.D.
hCoV-19/USA/AK-PHL8865/2021	EPI_ISL_2272556	5/14/2021	Alaska State Virology Laboratory	Alaska State Virology Laboratory	Stephanie DeRonde, Elva House, Jacob Zidek, Lisa Smith, Ph.D., Jack Chen, Ph.D.
hCoV-19/USA/AK-PHL8866/2021	EPI_ISL_2272557	5/13/2021	Alaska State Virology Laboratory	Alaska State Virology Laboratory	Stephanie DeRonde, Elva House, Jacob Zidek, Lisa Smith, Ph.D., Jack Chen, Ph.D.
hCoV-19/USA/AK-PHL8871/2021	EPI_ISL_2308645	5/12/2021	Alaska State Virology Laboratory	Alaska State Virology Laboratory	Stephanie DeRonde, Elva House, Jacob Zidek, Lisa Smith, Ph.D., Jack Chen, Ph.D.
hCoV-19/USA/AK-PHL8872/2021	EPI_ISL_2308646	5/14/2021	Alaska State Virology Laboratory	Alaska State Virology Laboratory	Stephanie DeRonde, Elva House, Jacob Zidek, Lisa Smith, Ph.D., Jack Chen, Ph.D.
hCoV-19/USA/AK-PHL8874/2021	EPI_ISL_2308647	5/12/2021	Alaska State Virology Laboratory	Alaska State Virology Laboratory	Stephanie DeRonde, Elva House, Jacob Zidek, Lisa Smith, Ph.D., Jack Chen, Ph.D.
hCoV-19/USA/AK-PHL8875/2021	EPI_ISL_2308648	5/14/2021	Alaska State Virology Laboratory	Alaska State Virology Laboratory	Stephanie DeRonde, Elva House, Jacob Zidek, Lisa Smith, Ph.D., Jack Chen, Ph.D.
hCoV-19/USA/AK-PHL8876/2021	EPI_ISL_2308649	5/12/2021	Alaska State Virology Laboratory	Alaska State Virology Laboratory	Stephanie DeRonde, Elva House, Jacob Zidek, Lisa Smith, Ph.D., Jack Chen, Ph.D.
hCoV-19/USA/AK-PHL8877/2021	EPI_ISL_2308650	5/12/2021	Alaska State Virology Laboratory	Alaska State Virology Laboratory	Stephanie DeRonde, Elva House, Jacob Zidek, Lisa Smith, Ph.D., Jack Chen, Ph.D.
hCoV-19/USA/AK-PHL8878/2021	EPI_ISL_2308651	5/12/2021	Alaska State Virology Laboratory	Alaska State Virology Laboratory	Stephanie DeRonde, Elva House, Jacob Zidek, Lisa Smith, Ph.D., Jack Chen, Ph.D.
hCoV-19/USA/AK-PHL8879/2021	EPI_ISL_2308652	5/11/2021	Alaska State Virology Laboratory	Alaska State Virology Laboratory	Stephanie DeRonde, Elva House, Jacob Zidek, Lisa Smith, Ph.D., Jack Chen, Ph.D.
hCoV-19/USA/AK-PHL8880/2021	EPI_ISL_2308653	5/11/2021	Alaska State Virology Laboratory	Alaska State Virology Laboratory	Stephanie DeRonde, Elva House, Jacob Zidek, Lisa Smith, Ph.D., Jack Chen, Ph.D.
hCoV-19/USA/AK-PHL8882/2021	EPI_ISL_2308654	5/12/2021	Alaska State Virology Laboratory	Alaska State Virology Laboratory	Stephanie DeRonde, Elva House, Jacob Zidek, Lisa Smith, Ph.D., Jack Chen, Ph.D.
hCoV-19/USA/AK-PHL8883/2021	EPI_ISL_2308655	5/12/2021	Alaska State Virology Laboratory	Alaska State Virology Laboratory	Stephanie DeRonde, Elva House, Jacob Zidek, Lisa Smith, Ph.D., Jack Chen, Ph.D.
hCoV-19/USA/AK-PHL8884/2021	EPI_ISL_2308656	5/12/2021	Alaska State Virology Laboratory	Alaska State Virology Laboratory	Stephanie DeRonde, Elva House, Jacob Zidek, Lisa Smith, Ph.D., Jack Chen, Ph.D.
hCoV-19/USA/AK-PHL8888/2021	EPI_ISL_2308657	5/11/2021	Alaska State Virology Laboratory	Alaska State Virology Laboratory	Stephanie DeRonde, Elva House, Jacob Zidek, Lisa Smith, Ph.D., Jack Chen, Ph.D.
hCoV-19/USA/AK-PHL8889/2021	EPI_ISL_2308658	5/9/2021	Alaska State Virology Laboratory	Alaska State Virology Laboratory	Stephanie DeRonde, Elva House, Jacob Zidek, Lisa Smith, Ph.D., Jack Chen, Ph.D.
hCoV-19/USA/AK-PHL8897/2021	EPI_ISL_2308659	5/13/2021	Alaska State Virology Laboratory	Alaska State Virology Laboratory	Stephanie DeRonde, Elva House, Jacob Zidek, Lisa Smith, Ph.D., Jack Chen, Ph.D.
hCoV-19/USA/AK-PHL8898/2021	EPI_ISL_2308660	5/13/2021	Alaska State Virology Laboratory	Alaska State Virology Laboratory	Stephanie DeRonde, Elva House, Jacob Zidek, Lisa Smith, Ph.D., Jack Chen, Ph.D.
hCoV-19/USA/AK-PHL8899/2021	EPI_ISL_2308661	5/13/2021	Alaska State Virology Laboratory	Alaska State Virology Laboratory	Stephanie DeRonde, Elva House, Jacob Zidek, Lisa Smith, Ph.D., Jack Chen, Ph.D.
hCoV-19/USA/AK-PHL8900/2021	EPI_ISL_2308662	5/13/2021	Alaska State Virology Laboratory	Alaska State Virology Laboratory	Stephanie DeRonde, Elva House, Jacob Zidek, Lisa Smith, Ph.D., Jack Chen, Ph.D.
hCoV-19/USA/AK-PHL8902/2021	EPI_ISL_2308663	5/13/2021	Alaska State Virology Laboratory	Alaska State Virology Laboratory	Stephanie DeRonde, Elva House, Jacob Zidek, Lisa Smith, Ph.D., Jack Chen, Ph.D.
hCoV-19/USA/AK-PHL8903/2021	EPI_ISL_2308664	5/13/2021	Alaska State Virology Laboratory	Alaska State Virology Laboratory	Stephanie DeRonde, Elva House, Jacob Zidek, Lisa Smith, Ph.D., Jack Chen, Ph.D.
hCoV-19/USA/AK-PHL8904/2021	EPI_ISL_2308665	5/13/2021	Alaska State Virology Laboratory	Alaska State Virology Laboratory	Stephanie DeRonde, Elva House, Jacob Zidek, Lisa Smith, Ph.D., Jack Chen, Ph.D.
hCoV-19/USA/AK-PHL8905/2021	EPI_ISL_2308666	5/14/2021	Alaska State Virology Laboratory	Alaska State Virology Laboratory	Stephanie DeRonde, Elva House, Jacob Zidek, Lisa Smith, Ph.D., Jack Chen, Ph.D.

hCoV-19/USA/AK-PHL8906/2021	EPI_ISL_2308667	5/14/2021	Alaska State Virology Laboratory	Alaska State Virology Laboratory	Stephanie DeRonde, Elva House, Jacob Zidek, Lisa Smith, Ph.D., Jack Chen, Ph.D.
hCoV-19/USA/AK-PHL8909/2021	EPI_ISL_2308668	5/14/2021	Alaska State Virology Laboratory	Alaska State Virology Laboratory	Stephanie DeRonde, Elva House, Jacob Zidek, Lisa Smith, Ph.D., Jack Chen, Ph.D.
hCoV-19/USA/AK-PHL8910/2021	EPI_ISL_2308669	5/14/2021	Alaska State Virology Laboratory	Alaska State Virology Laboratory	Stephanie DeRonde, Elva House, Jacob Zidek, Lisa Smith, Ph.D., Jack Chen, Ph.D.
hCoV-19/USA/AK-PHL8911/2021	EPI_ISL_2308670	5/14/2021	Alaska State Virology Laboratory	Alaska State Virology Laboratory	Stephanie DeRonde, Elva House, Jacob Zidek, Lisa Smith, Ph.D., Jack Chen, Ph.D.
hCoV-19/USA/AK-PHL8908/2021	EPI_ISL_2322742	5/14/2021	Alaska State Virology Laboratory	Alaska State Virology Laboratory	Stephanie DeRonde, Elva House, Jacob Zidek, Lisa Smith, Ph.D., Jack Chen, Ph.D.
hCoV-19/USA/AK-PHL8504/2021	EPI_ISL_2009004	4/30/2021	Alaska State Virology Laboratory	Alaska State Virology Laboratory	Stephanie DeRonde, Elva House, Jacob Zidek, Lisa Smith, Ph.D., Jack Chen, Ph.D.
hCoV-19/USA/AK-PHL6772/2021	EPI_ISL_2348954	1/29/2021	Alaska State Virology Lab, Alaska State Public Health Labs	Alaska State Virology Lab, Alaska State Public Health Labs	DeRonde,S., House,E., Zidek,J., Redlinger,M., Smith,L., Chen,J.
hCoV-19/USA/AK-PHL6923/2021	EPI_ISL_2348956	2/8/2021	Alaska State Virology Lab, Alaska State Public Health Labs	Alaska State Virology Lab, Alaska State Public Health Labs	DeRonde,S., House,E., Zidek,J., Redlinger,M., Smith,L., Chen,J.
hCoV-19/USA/AK-PHL6927/2021	EPI_ISL_2348957	2/2/2021	Alaska State Virology Lab, Alaska State Public Health Labs	Alaska State Virology Lab, Alaska State Public Health Labs	DeRonde,S., House,E., Zidek,J., Redlinger,M., Smith,L., Chen,J.
hCoV-19/USA/AK-PHL7243/2021	EPI_ISL_2348958	2/17/2021	Alaska State Virology Lab, Alaska State Public Health Labs	Alaska State Virology Lab, Alaska State Public Health Labs	DeRonde,S., House,E., Zidek,J., Redlinger,M., Smith,L., Chen,J.
hCoV-19/USA/AK-PHL7257/2021	EPI_ISL_2348959	2/17/2021	Alaska State Virology Lab, Alaska State Public Health Labs	Alaska State Virology Lab, Alaska State Public Health Labs	DeRonde,S., House,E., Zidek,J., Redlinger,M., Smith,L., Chen,J.
hCoV-19/USA/AK-CDC-ASC210065763/2021	EPI_ISL_2370048	5/19/2021	Aegis Sciences Corporation	Centers for Disease Control and Prevention Division of Viral Diseases, Pathogen Discovery	Dakota Howard, Dhwani Batra, Peter W. Cook, Kara Moser, Adrian Paskey, Jason Caravas, Benjamin Rambo-Martin, Shatavia Morrison, Christopher Gulvick, Scott Sammons, Yvette Unoarumhi, Darlene Wagner, Matthew Schmerer, Cyndi Clark, Patrick Campbell, Rob Case, Vikramsinha Ghorpade, Holly Houdeshell, Ola Kvalvaag, Dillon Nall, Ethan Sanders, Alec Vest, Shaun Westlund, Matthew Hardison, Clinton R. Paden, Duncan MacCannell
hCoV-19/USA/AK-PHL9019/2021	EPI_ISL_2375803	5/18/2021	Alaska State Virology Laboratory	Alaska State Virology Laboratory	Stephanie DeRonde, Elva House, Jacob Zidek, Lisa Smith, Ph.D., Jack Chen, Ph.D.
hCoV-19/USA/AK-PHL9020/2021	EPI_ISL_2375804	5/18/2021	Alaska State Virology Laboratory	Alaska State Virology Laboratory	Stephanie DeRonde, Elva House, Jacob Zidek, Lisa Smith, Ph.D., Jack Chen, Ph.D.
hCoV-19/USA/AK-PHL9021/2021	EPI_ISL_2375805	5/18/2021	Alaska State Virology Laboratory	Alaska State Virology Laboratory	Stephanie DeRonde, Elva House, Jacob Zidek, Lisa Smith, Ph.D., Jack Chen, Ph.D.
hCoV-19/USA/AK-PHL9022/2021	EPI_ISL_2375806	5/18/2021	Alaska State Virology Laboratory	Alaska State Virology Laboratory	Stephanie DeRonde, Elva House, Jacob Zidek, Lisa Smith, Ph.D., Jack Chen, Ph.D.
hCoV-19/USA/AK-PHL9023/2021	EPI_ISL_2375807	5/18/2021	Alaska State Virology Laboratory	Alaska State Virology Laboratory	Stephanie DeRonde, Elva House, Jacob Zidek, Lisa Smith, Ph.D., Jack Chen, Ph.D.
hCoV-19/USA/AK-PHL9025/2021	EPI_ISL_2375808	5/18/2021	Alaska State Virology Laboratory	Alaska State Virology Laboratory	Stephanie DeRonde, Elva House, Jacob Zidek, Lisa Smith, Ph.D., Jack Chen, Ph.D.
hCoV-19/USA/AK-PHL9026/2021	EPI_ISL_2375809	5/18/2021	Alaska State Virology Laboratory	Alaska State Virology Laboratory	Stephanie DeRonde, Elva House, Jacob Zidek, Lisa Smith, Ph.D., Jack Chen, Ph.D.
hCoV-19/USA/AK-PHL9027/2021	EPI_ISL_2375810	5/18/2021	Alaska State Virology Laboratory	Alaska State Virology Laboratory	Stephanie DeRonde, Elva House, Jacob Zidek, Lisa Smith, Ph.D., Jack Chen, Ph.D.
hCoV-19/USA/AK-PHL9028/2021	EPI_ISL_2375811	5/18/2021	Alaska State Virology Laboratory	Alaska State Virology Laboratory	Stephanie DeRonde, Elva House, Jacob Zidek, Lisa Smith, Ph.D., Jack Chen, Ph.D.
hCoV-19/USA/AK-PHL9029/2021	EPI_ISL_2375812	5/18/2021	Alaska State Virology Laboratory	Alaska State Virology Laboratory	Stephanie DeRonde, Elva House, Jacob Zidek, Lisa Smith, Ph.D., Jack Chen, Ph.D.
hCoV-19/USA/AK-PHL9030/2021	EPI_ISL_2375813	5/18/2021	Alaska State Virology Laboratory	Alaska State Virology Laboratory	Stephanie DeRonde, Elva House, Jacob Zidek, Lisa Smith, Ph.D., Jack Chen, Ph.D.
hCoV-19/USA/AK-PHL9031/2021	EPI_ISL_2375814	5/18/2021	Alaska State Virology Laboratory	Alaska State Virology Laboratory	Stephanie DeRonde, Elva House, Jacob Zidek, Lisa Smith, Ph.D., Jack Chen, Ph.D.
hCoV-19/USA/AK-PHL9032/2021	EPI_ISL_2375815	5/17/2021	Alaska State Virology Laboratory	Alaska State Virology Laboratory	Stephanie DeRonde, Elva House, Jacob Zidek, Lisa Smith, Ph.D., Jack Chen, Ph.D.
hCoV-19/USA/AK-PHL9033/2021	EPI_ISL_2375816	5/17/2021	Alaska State Virology Laboratory	Alaska State Virology Laboratory	Stephanie DeRonde, Elva House, Jacob Zidek, Lisa Smith, Ph.D., Jack Chen, Ph.D.
hCoV-19/USA/AK-PHL9034/2021	EPI_ISL_2375817	5/17/2021	Alaska State Virology Laboratory	Alaska State Virology Laboratory	Stephanie DeRonde, Elva House, Jacob Zidek, Lisa Smith, Ph.D., Jack Chen, Ph.D.
hCoV-19/USA/AK-PHL9036/2021	EPI_ISL_2375818	5/18/2021	Alaska State Virology Laboratory	Alaska State Virology Laboratory	Stephanie DeRonde, Elva House, Jacob Zidek, Lisa Smith, Ph.D., Jack Chen, Ph.D.
hCoV-19/USA/AK-PHL9037/2021	EPI_ISL_2375819	5/18/2021	Alaska State Virology Laboratory	Alaska State Virology Laboratory	Stephanie DeRonde, Elva House, Jacob Zidek, Lisa Smith, Ph.D., Jack Chen, Ph.D.
hCoV-19/USA/AK-PHL9039/2021	EPI_ISL_2375820	5/18/2021	Alaska State Virology Laboratory	Alaska State Virology Laboratory	Stephanie DeRonde, Elva House, Jacob Zidek, Lisa Smith, Ph.D., Jack Chen, Ph.D.
hCoV-19/USA/AK-PHL9040/2021	EPI_ISL_2375821	5/18/2021	Alaska State Virology Laboratory	Alaska State Virology Laboratory	Stephanie DeRonde, Elva House, Jacob Zidek, Lisa Smith, Ph.D., Jack Chen, Ph.D.
hCoV-19/USA/AK-PHL9041/2021	EPI_ISL_2375822	5/13/2021	Alaska State Virology Laboratory	Alaska State Virology Laboratory	Stephanie DeRonde, Elva House, Jacob Zidek, Lisa Smith, Ph.D., Jack Chen, Ph.D.
hCoV-19/USA/AK-PHL9042/2021	EPI_ISL_2375823	4/20/2021	Alaska State Virology Laboratory	Alaska State Virology Laboratory	Stephanie DeRonde, Elva House, Jacob Zidek, Lisa Smith, Ph.D., Jack Chen, Ph.D.
hCoV-19/USA/AK-PHL9043/2021	EPI_ISL_2375824	5/2/2021	Alaska State Virology Laboratory	Alaska State Virology Laboratory	Stephanie DeRonde, Elva House, Jacob Zidek, Lisa Smith, Ph.D., Jack Chen, Ph.D.
hCoV-19/USA/AK-PHL9044/2021	EPI_ISL_2375825	4/14/2021	Alaska State Virology Laboratory	Alaska State Virology Laboratory	Stephanie DeRonde, Elva House, Jacob Zidek, Lisa Smith, Ph.D., Jack Chen, Ph.D.
hCoV-19/USA/AK-PHL9045/2021	EPI_ISL_2375826	5/3/2021	Alaska State Virology Laboratory	Alaska State Virology Laboratory	Stephanie DeRonde, Elva House, Jacob Zidek, Lisa Smith, Ph.D., Jack Chen, Ph.D.
hCoV-19/USA/AK-PHL9046/2021	EPI_ISL_2375827	5/12/2021	Alaska State Virology Laboratory	Alaska State Virology Laboratory	Stephanie DeRonde, Elva House, Jacob Zidek, Lisa Smith, Ph.D., Jack Chen, Ph.D.
hCoV-19/USA/AK-PHL9047/2021	EPI_ISL_2375828	5/14/2021	Alaska State Virology Laboratory	Alaska State Virology Laboratory	Stephanie DeRonde, Elva House, Jacob Zidek, Lisa Smith, Ph.D., Jack Chen, Ph.D.
hCoV-19/USA/AK-PHL9048/2021	EPI_ISL_2375829	5/8/2021	Alaska State Virology Laboratory	Alaska State Virology Laboratory	Stephanie DeRonde, Elva House, Jacob Zidek, Lisa Smith, Ph.D., Jack Chen, Ph.D.
hCoV-19/USA/AK-PHL9049/2021	EPI_ISL_2375830	5/15/2021	Alaska State Virology Laboratory	Alaska State Virology Laboratory	Stephanie DeRonde, Elva House, Jacob Zidek, Lisa Smith, Ph.D., Jack Chen, Ph.D.
hCoV-19/USA/AK-PHL9050/2021	EPI_ISL_2375831	5/16/2021	Alaska State Virology Laboratory	Alaska State Virology Laboratory	Stephanie DeRonde, Elva House, Jacob Zidek, Lisa Smith, Ph.D., Jack Chen, Ph.D.
hCoV-19/USA/AK-PHL9051/2021	EPI_ISL_2375832	5/19/2021	Alaska State Virology Laboratory	Alaska State Virology Laboratory	Stephanie DeRonde, Elva House, Jacob Zidek, Lisa Smith, Ph.D., Jack Chen, Ph.D.
hCoV-19/USA/AK-PHL9052/2021	EPI_ISL_2375833	5/19/2021	Alaska State Virology Laboratory	Alaska State Virology Laboratory	Stephanie DeRonde, Elva House, Jacob Zidek, Lisa Smith, Ph.D., Jack Chen, Ph.D.
hCoV-19/USA/AK-PHL9054/2021	EPI_ISL_2375834	5/19/2021	Alaska State Virology Laboratory	Alaska State Virology Laboratory	Stephanie DeRonde, Elva House, Jacob Zidek, Lisa Smith, Ph.D., Jack Chen, Ph.D.
hCoV-19/USA/AK-PHL9055/2021	EPI_ISL_2375835	5/19/2021	Alaska State Virology Laboratory	Alaska State Virology Laboratory	Stephanie DeRonde, Elva House, Jacob Zidek, Lisa Smith, Ph.D., Jack Chen, Ph.D.
hCoV-19/USA/AK-PHL9057/2021	EPI_ISL_2375836	5/19/2021	Alaska State Virology Laboratory	Alaska State Virology Laboratory	Stephanie DeRonde, Elva House, Jacob Zidek, Lisa Smith, Ph.D., Jack Chen, Ph.D.

hCoV-19/USA/AK-PHL9058/2021	EPI_ISL_2375837	5/19/2021	Alaska State Virology Laboratory	Alaska State Virology Laboratory	Stephanie DeRonde, Elva House, Jacob Zidek, Lisa Smith, Ph.D., Jack Chen, Ph.D.
hCoV-19/USA/AK-PHL9063/2021	EPI_ISL_2375996	5/19/2021	Alaska State Virology Laboratory	Alaska State Virology Laboratory	Stephanie DeRonde, Elva House, Jacob Zidek, Lisa Smith, Ph.D., Jack Chen, Ph.D.
hCoV-19/USA/AK-PHL9064/2021	EPI_ISL_2375997	5/20/2021	Alaska State Virology Laboratory	Alaska State Virology Laboratory	Stephanie DeRonde, Elva House, Jacob Zidek, Lisa Smith, Ph.D., Jack Chen, Ph.D.
hCoV-19/USA/AK-PHL9069/2021	EPI_ISL_2375998	5/20/2021	Alaska State Virology Laboratory	Alaska State Virology Laboratory	Stephanie DeRonde, Elva House, Jacob Zidek, Lisa Smith, Ph.D., Jack Chen, Ph.D.
hCoV-19/USA/AK-PHL9070/2021	EPI_ISL_2375999	5/20/2021	Alaska State Virology Laboratory	Alaska State Virology Laboratory	Stephanie DeRonde, Elva House, Jacob Zidek, Lisa Smith, Ph.D., Jack Chen, Ph.D.
hCoV-19/USA/AK-PHL9073/2021	EPI_ISL_2376000	5/20/2021	Alaska State Virology Laboratory	Alaska State Virology Laboratory	Stephanie DeRonde, Elva House, Jacob Zidek, Lisa Smith, Ph.D., Jack Chen, Ph.D.
hCoV-19/USA/AK-PHL9074/2021	EPI_ISL_2376001	5/22/2021	Alaska State Virology Laboratory	Alaska State Virology Laboratory	Stephanie DeRonde, Elva House, Jacob Zidek, Lisa Smith, Ph.D., Jack Chen, Ph.D.
hCoV-19/USA/AK-PHL9075/2021	EPI_ISL_2376002	5/21/2021	Alaska State Virology Laboratory	Alaska State Virology Laboratory	Stephanie DeRonde, Elva House, Jacob Zidek, Lisa Smith, Ph.D., Jack Chen, Ph.D.
hCoV-19/USA/AK-PHL9077/2021	EPI_ISL_2376003	5/22/2021	Alaska State Virology Laboratory	Alaska State Virology Laboratory	Stephanie DeRonde, Elva House, Jacob Zidek, Lisa Smith, Ph.D., Jack Chen, Ph.D.
hCoV-19/USA/AK-PHL9078/2021	EPI_ISL_2376004	5/21/2021	Alaska State Virology Laboratory	Alaska State Virology Laboratory	Stephanie DeRonde, Elva House, Jacob Zidek, Lisa Smith, Ph.D., Jack Chen, Ph.D.
hCoV-19/USA/AK-PHL9079/2021	EPI_ISL_2376005	5/21/2021	Alaska State Virology Laboratory	Alaska State Virology Laboratory	Stephanie DeRonde, Elva House, Jacob Zidek, Lisa Smith, Ph.D., Jack Chen, Ph.D.
hCoV-19/USA/AK-PHL9081/2021	EPI_ISL_2376006	5/21/2021	Alaska State Virology Laboratory	Alaska State Virology Laboratory	Stephanie DeRonde, Elva House, Jacob Zidek, Lisa Smith, Ph.D., Jack Chen, Ph.D.
hCoV-19/USA/AK-PHL9082/2021	EPI_ISL_2376007	5/21/2021	Alaska State Virology Laboratory	Alaska State Virology Laboratory	Stephanie DeRonde, Elva House, Jacob Zidek, Lisa Smith, Ph.D., Jack Chen, Ph.D.
hCoV-19/USA/AK-PHL9083/2021	EPI_ISL_2376008	5/20/2021	Alaska State Virology Laboratory	Alaska State Virology Laboratory	Stephanie DeRonde, Elva House, Jacob Zidek, Lisa Smith, Ph.D., Jack Chen, Ph.D.
hCoV-19/USA/AK-PHL9013/2021	EPI_ISL_2376009	5/19/2021	Alaska State Virology Laboratory	Alaska State Virology Laboratory	Stephanie DeRonde, Elva House, Jacob Zidek, Lisa Smith, Ph.D., Jack Chen, Ph.D.
hCoV-19/USA/AK-PHL9006/2021	EPI_ISL_2376010	5/17/2021	Alaska State Virology Laboratory	Alaska State Virology Laboratory	Stephanie DeRonde, Elva House, Jacob Zidek, Lisa Smith, Ph.D., Jack Chen, Ph.D.
hCoV-19/USA/AK-PHL9010/2021	EPI_ISL_2376011	5/18/2021	Alaska State Virology Laboratory	Alaska State Virology Laboratory	Stephanie DeRonde, Elva House, Jacob Zidek, Lisa Smith, Ph.D., Jack Chen, Ph.D.
hCoV-19/USA/AK-PHL9007/2021	EPI_ISL_2376012	5/17/2021	Alaska State Virology Laboratory	Alaska State Virology Laboratory	Stephanie DeRonde, Elva House, Jacob Zidek, Lisa Smith, Ph.D., Jack Chen, Ph.D.
hCoV-19/USA/AK-PHL9089/2021	EPI_ISL_2376013	5/18/2021	Alaska State Virology Laboratory	Alaska State Virology Laboratory	Stephanie DeRonde, Elva House, Jacob Zidek, Lisa Smith, Ph.D., Jack Chen, Ph.D.
hCoV-19/USA/AK-PHL9008/2021	EPI_ISL_2376014	5/17/2021	Alaska State Virology Laboratory	Alaska State Virology Laboratory	Stephanie DeRonde, Elva House, Jacob Zidek, Lisa Smith, Ph.D., Jack Chen, Ph.D.
hCoV-19/USA/AK-PHL9093/2021	EPI_ISL_2376015	5/17/2021	Alaska State Virology Laboratory	Alaska State Virology Laboratory	Stephanie DeRonde, Elva House, Jacob Zidek, Lisa Smith, Ph.D., Jack Chen, Ph.D.
hCoV-19/USA/AK-PHL9094/2021	EPI_ISL_2376016	5/17/2021	Alaska State Virology Laboratory	Alaska State Virology Laboratory	Stephanie DeRonde, Elva House, Jacob Zidek, Lisa Smith, Ph.D., Jack Chen, Ph.D.
hCoV-19/USA/AK-PHL9096/2021	EPI_ISL_2376017	5/17/2021	Alaska State Virology Laboratory	Alaska State Virology Laboratory	Stephanie DeRonde, Elva House, Jacob Zidek, Lisa Smith, Ph.D., Jack Chen, Ph.D.
hCoV-19/USA/AK-PHL9097/2021	EPI_ISL_2376018	5/16/2021	Alaska State Virology Laboratory	Alaska State Virology Laboratory	Stephanie DeRonde, Elva House, Jacob Zidek, Lisa Smith, Ph.D., Jack Chen, Ph.D.
hCoV-19/USA/AK-PHL9014/2021	EPI_ISL_2376019	5/20/2021	Alaska State Virology Laboratory	Alaska State Virology Laboratory	Stephanie DeRonde, Elva House, Jacob Zidek, Lisa Smith, Ph.D., Jack Chen, Ph.D.
hCoV-19/USA/AK-PHL9016/2021	EPI_ISL_2376020	5/20/2021	Alaska State Virology Laboratory	Alaska State Virology Laboratory	Stephanie DeRonde, Elva House, Jacob Zidek, Lisa Smith, Ph.D., Jack Chen, Ph.D.
hCoV-19/USA/AK-PHL9103/2021	EPI_ISL_2376021	5/1/2021	Alaska State Virology Laboratory	Alaska State Virology Laboratory	Stephanie DeRonde, Elva House, Jacob Zidek, Lisa Smith, Ph.D., Jack Chen, Ph.D.
hCoV-19/USA/AK-PHL9104/2021	EPI_ISL_2376022	4/29/2021	Alaska State Virology Laboratory	Alaska State Virology Laboratory	Stephanie DeRonde, Elva House, Jacob Zidek, Lisa Smith, Ph.D., Jack Chen, Ph.D.
hCoV-19/USA/AK-PHL9105/2021	EPI_ISL_2376023	4/30/2021	Alaska State Virology Laboratory	Alaska State Virology Laboratory	Stephanie DeRonde, Elva House, Jacob Zidek, Lisa Smith, Ph.D., Jack Chen, Ph.D.
hCoV-19/USA/AK-PHL9106/2021	EPI_ISL_2376024	4/30/2021	Alaska State Virology Laboratory	Alaska State Virology Laboratory	Stephanie DeRonde, Elva House, Jacob Zidek, Lisa Smith, Ph.D., Jack Chen, Ph.D.
hCoV-19/USA/AK-PHL9107/2021	EPI_ISL_2376025	5/5/2021	Alaska State Virology Laboratory	Alaska State Virology Laboratory	Stephanie DeRonde, Elva House, Jacob Zidek, Lisa Smith, Ph.D., Jack Chen, Ph.D.
hCoV-19/USA/AK-PHL9108/2021	EPI_ISL_2376026	5/1/2021	Alaska State Virology Laboratory	Alaska State Virology Laboratory	Stephanie DeRonde, Elva House, Jacob Zidek, Lisa Smith, Ph.D., Jack Chen, Ph.D.
hCoV-19/USA/AK-PHL8959/2021	EPI_ISL_2376068	5/16/2021	Alaska State Virology Laboratory	Alaska State Virology Laboratory	Stephanie DeRonde, Elva House, Jacob Zidek, Lisa Smith, Ph.D., Jack Chen, Ph.D.
hCoV-19/USA/AK-PHL8960/2021	EPI_ISL_2376069	5/3/2021	Alaska State Virology Laboratory	Alaska State Virology Laboratory	Stephanie DeRonde, Elva House, Jacob Zidek, Lisa Smith, Ph.D., Jack Chen, Ph.D.
hCoV-19/USA/AK-PHL8962/2021	EPI_ISL_2376070	5/5/2021	Alaska State Virology Laboratory	Alaska State Virology Laboratory	Stephanie DeRonde, Elva House, Jacob Zidek, Lisa Smith, Ph.D., Jack Chen, Ph.D.
hCoV-19/USA/AK-PHL8964/2021	EPI_ISL_2376071	5/14/2021	Alaska State Virology Laboratory	Alaska State Virology Laboratory	Stephanie DeRonde, Elva House, Jacob Zidek, Lisa Smith, Ph.D., Jack Chen, Ph.D.
hCoV-19/USA/AK-PHL8965/2021	EPI_ISL_2376072	5/15/2021	Alaska State Virology Laboratory	Alaska State Virology Laboratory	Stephanie DeRonde, Elva House, Jacob Zidek, Lisa Smith, Ph.D., Jack Chen, Ph.D.
hCoV-19/USA/AK-PHL8966/2021	EPI_ISL_2376073	5/14/2021	Alaska State Virology Laboratory	Alaska State Virology Laboratory	Stephanie DeRonde, Elva House, Jacob Zidek, Lisa Smith, Ph.D., Jack Chen, Ph.D.
hCoV-19/USA/AK-PHL8967/2021	EPI_ISL_2376074	5/14/2021	Alaska State Virology Laboratory	Alaska State Virology Laboratory	Stephanie DeRonde, Elva House, Jacob Zidek, Lisa Smith, Ph.D., Jack Chen, Ph.D.
hCoV-19/USA/AK-PHL8968/2021	EPI_ISL_2376075	5/13/2021	Alaska State Virology Laboratory	Alaska State Virology Laboratory	Stephanie DeRonde, Elva House, Jacob Zidek, Lisa Smith, Ph.D., Jack Chen, Ph.D.
hCoV-19/USA/AK-PHL8969/2021	EPI_ISL_2376076	5/14/2021	Alaska State Virology Laboratory	Alaska State Virology Laboratory	Stephanie DeRonde, Elva House, Jacob Zidek, Lisa Smith, Ph.D., Jack Chen, Ph.D.
hCoV-19/USA/AK-PHL8970/2021	EPI_ISL_2376077	5/14/2021	Alaska State Virology Laboratory	Alaska State Virology Laboratory	Stephanie DeRonde, Elva House, Jacob Zidek, Lisa Smith, Ph.D., Jack Chen, Ph.D.
hCoV-19/USA/AK-PHL8972/2021	EPI_ISL_2376078	5/7/2021	Alaska State Virology Laboratory	Alaska State Virology Laboratory	Stephanie DeRonde, Elva House, Jacob Zidek, Lisa Smith, Ph.D., Jack Chen, Ph.D.
hCoV-19/USA/AK-PHL8973/2021	EPI_ISL_2376079	5/12/2021	Alaska State Virology Laboratory	Alaska State Virology Laboratory	Stephanie DeRonde, Elva House, Jacob Zidek, Lisa Smith, Ph.D., Jack Chen, Ph.D.
hCoV-19/USA/AK-PHL8974/2021	EPI_ISL_2376080	5/13/2021	Alaska State Virology Laboratory	Alaska State Virology Laboratory	Stephanie DeRonde, Elva House, Jacob Zidek, Lisa Smith, Ph.D., Jack Chen, Ph.D.
hCoV-19/USA/AK-PHL8975/2021	EPI_ISL_2376081	5/14/2021	Alaska State Virology Laboratory	Alaska State Virology Laboratory	Stephanie DeRonde, Elva House, Jacob Zidek, Lisa Smith, Ph.D., Jack Chen, Ph.D.
hCoV-19/USA/AK-PHL8977/2021	EPI_ISL_2376082	5/15/2021	Alaska State Virology Laboratory	Alaska State Virology Laboratory	Stephanie DeRonde, Elva House, Jacob Zidek, Lisa Smith, Ph.D., Jack Chen, Ph.D.
hCoV-19/USA/AK-PHL8978/2021	EPI_ISL_2376083	5/10/2021	Alaska State Virology Laboratory	Alaska State Virology Laboratory	Stephanie DeRonde, Elva House, Jacob Zidek, Lisa Smith, Ph.D., Jack Chen, Ph.D.
hCoV-19/USA/AK-PHL8980/2021	EPI_ISL_2376084	5/9/2021	Alaska State Virology Laboratory	Alaska State Virology Laboratory	Stephanie DeRonde, Elva House, Jacob Zidek, Lisa Smith, Ph.D., Jack Chen, Ph.D.
hCoV-19/USA/AK-PHL8981/2021	EPI_ISL_2376085	5/10/2021	Alaska State Virology Laboratory	Alaska State Virology Laboratory	Stephanie DeRonde, Elva House, Jacob Zidek, Lisa Smith, Ph.D., Jack Chen, Ph.D.
hCoV-19/USA/AK-PHL8982/2021	EPI_ISL_2376086	5/10/2021	Alaska State Virology Laboratory	Alaska State Virology Laboratory	Stephanie DeRonde, Elva House, Jacob Zidek, Lisa Smith, Ph.D., Jack Chen, Ph.D.

hCoV-19/USA/AK-PHL8983/2021	EPI_ISL_2376087	5/18/2021	Alaska State Virology Laboratory	Alaska State Virology Laboratory	Stephanie DeRonde, Elva House, Jacob Zidek, Lisa Smith, Ph.D., Jack Chen, Ph.D.
hCoV-19/USA/AK-PHL8984/2021	EPI_ISL_2376088	5/18/2021	Alaska State Virology Laboratory	Alaska State Virology Laboratory	Stephanie DeRonde, Elva House, Jacob Zidek, Lisa Smith, Ph.D., Jack Chen, Ph.D.
hCoV-19/USA/AK-PHL8986/2021	EPI_ISL_2376089	5/13/2021	Alaska State Virology Laboratory	Alaska State Virology Laboratory	Stephanie DeRonde, Elva House, Jacob Zidek, Lisa Smith, Ph.D., Jack Chen, Ph.D.
hCoV-19/USA/AK-PHL8989/2021	EPI_ISL_2376090	5/17/2021	Alaska State Virology Laboratory	Alaska State Virology Laboratory	Stephanie DeRonde, Elva House, Jacob Zidek, Lisa Smith, Ph.D., Jack Chen, Ph.D.
hCoV-19/USA/AK-PHL8990/2021	EPI_ISL_2376091	5/11/2021	Alaska State Virology Laboratory	Alaska State Virology Laboratory	Stephanie DeRonde, Elva House, Jacob Zidek, Lisa Smith, Ph.D., Jack Chen, Ph.D.
hCoV-19/USA/AK-PHL8991/2021	EPI_ISL_2376092	5/11/2021	Alaska State Virology Laboratory	Alaska State Virology Laboratory	Stephanie DeRonde, Elva House, Jacob Zidek, Lisa Smith, Ph.D., Jack Chen, Ph.D.
hCoV-19/USA/AK-PHL8994/2021	EPI_ISL_2376093	5/13/2021	Alaska State Virology Laboratory	Alaska State Virology Laboratory	Stephanie DeRonde, Elva House, Jacob Zidek, Lisa Smith, Ph.D., Jack Chen, Ph.D.
hCoV-19/USA/AK-PHL8995/2021	EPI_ISL_2376094	5/13/2021	Alaska State Virology Laboratory	Alaska State Virology Laboratory	Stephanie DeRonde, Elva House, Jacob Zidek, Lisa Smith, Ph.D., Jack Chen, Ph.D.
hCoV-19/USA/AK-PHL8996/2021	EPI_ISL_2376095	5/13/2021	Alaska State Virology Laboratory	Alaska State Virology Laboratory	Stephanie DeRonde, Elva House, Jacob Zidek, Lisa Smith, Ph.D., Jack Chen, Ph.D.
hCoV-19/USA/AK-PHL8998/2021	EPI_ISL_2376096	5/13/2021	Alaska State Virology Laboratory	Alaska State Virology Laboratory	Stephanie DeRonde, Elva House, Jacob Zidek, Lisa Smith, Ph.D., Jack Chen, Ph.D.
hCoV-19/USA/AK-PHL8999/2021	EPI_ISL_2376097	5/13/2021	Alaska State Virology Laboratory	Alaska State Virology Laboratory	Stephanie DeRonde, Elva House, Jacob Zidek, Lisa Smith, Ph.D., Jack Chen, Ph.D.
hCoV-19/USA/AK-PHL9000/2021	EPI_ISL_2376098	5/13/2021	Alaska State Virology Laboratory	Alaska State Virology Laboratory	Stephanie DeRonde, Elva House, Jacob Zidek, Lisa Smith, Ph.D., Jack Chen, Ph.D.
hCoV-19/USA/AK-PHL9001/2021	EPI_ISL_2376099	5/13/2021	Alaska State Virology Laboratory	Alaska State Virology Laboratory	Stephanie DeRonde, Elva House, Jacob Zidek, Lisa Smith, Ph.D., Jack Chen, Ph.D.
hCoV-19/USA/AK-PHL8491/2021	EPI_ISL_2008998	4/19/2021	Alaska State Virology Laboratory	Alaska State Virology Laboratory	Stephanie DeRonde, Elva House, Jacob Zidek, Lisa Smith, Ph.D., Jack Chen, Ph.D.
hCoV-19/USA/AK-PHL8502/2021	EPI_ISL_2008995	4/28/2021	Alaska State Virology Laboratory	Alaska State Virology Laboratory	Stephanie DeRonde, Elva House, Jacob Zidek, Lisa Smith, Ph.D., Jack Chen, Ph.D.
hCoV-19/USA/AK-PHL8498/2021	EPI_ISL_2009002	4/19/2021	Alaska State Virology Laboratory	Alaska State Virology Laboratory	Stephanie DeRonde, Elva House, Jacob Zidek, Lisa Smith, Ph.D., Jack Chen, Ph.D.
hCoV-19/USA/AK-PHL8503/2021	EPI_ISL_2009003	4/30/2021	Alaska State Virology Laboratory	Alaska State Virology Laboratory	Stephanie DeRonde, Elva House, Jacob Zidek, Lisa Smith, Ph.D., Jack Chen, Ph.D.
hCoV-19/USA/AK-PHL8518/2021	EPI_ISL_2009010	4/27/2021	Alaska State Virology Laboratory	Alaska State Virology Laboratory	Stephanie DeRonde, Elva House, Jacob Zidek, Lisa Smith, Ph.D., Jack Chen, Ph.D.
hCoV-19/USA/AK-PHL8521/2021	EPI_ISL_2009011	4/28/2021	Alaska State Virology Laboratory	Alaska State Virology Laboratory	Stephanie DeRonde, Elva House, Jacob Zidek, Lisa Smith, Ph.D., Jack Chen, Ph.D.
hCoV-19/USA/AK-PHL8523/2021	EPI_ISL_2009012	4/28/2021	Alaska State Virology Laboratory	Alaska State Virology Laboratory	Stephanie DeRonde, Elva House, Jacob Zidek, Lisa Smith, Ph.D., Jack Chen, Ph.D.
hCoV-19/USA/AK-PHL8867/2021	EPI_ISL_2384145	5/11/2021	Alaska State Virology Lab, Alaska State Public Health Labs	Alaska State Virology Lab, Alaska State Public Health Labs	DeRonde,S., House,E., Zidek,J., Redlinger,M., Smith,L., Chen,J.
hCoV-19/USA/AK-CDC-2-4306934/2021	EPI_ISL_2018259	4/12/2021	AK State Public Health Lab, State Health Department	Centers for Disease Control and Prevention Division of Viral Diseases, Pathogen Discovery	Mili Sheth, Sarah Nobles, Jasmine Padilla, Mark Burroughs, Shoshona Le, Katie Dillon, Peter Cook, Clinton R. Paden, Dhwani Batra, Krista Queen, Kristen Knipe, Dakota Howard, Yvette Unoarumhi, Darlene Wagner, Matthew Schmerer, Ben L. Rambo-Martin, Kristine Lacek, Sam Shepard, Alison Laufer Halpin, Dave Wentworth, Vivien Dugan, Suxiang Tong, Justin Lee
hCoV-19/USA/AK-PHL8513/2021	EPI_ISL_2008990	5/1/2021	Alaska State Virology Laboratory	Alaska State Virology Laboratory	Stephanie DeRonde, Elva House, Jacob Zidek, Lisa Smith, Ph.D., Jack Chen, Ph.D.
hCoV-19/USA/AK-PHL8490/2021	EPI_ISL_2153713	4/26/2021	Alaska State Virology Laboratory	Alaska State Virology Laboratory	Stephanie DeRonde, Elva House, Jacob Zidek, Lisa Smith, Ph.D., Jack Chen, Ph.D.
hCoV-19/USA/AK-PHL8387/2021	EPI_ISL_2153714	4/23/2021	Alaska State Virology Laboratory	Alaska State Virology Laboratory	Stephanie DeRonde, Elva House, Jacob Zidek, Lisa Smith, Ph.D., Jack Chen, Ph.D.
hCoV-19/USA/AK-PHL8750/2021	EPI_ISL_2154066	5/10/2021	Alaska State Virology Laboratory	Alaska State Virology Laboratory	Stephanie DeRonde, Elva House, Jacob Zidek, Lisa Smith, Ph.D., Jack Chen, Ph.D.
hCoV-19/USA/AK-CDC-2-4356531/2021	EPI_ISL_2229045	4/27/2021	AK State Public Health Lab, State Health Department	Centers for Disease Control and Prevention Division of Viral Diseases, Pathogen Discovery	Mili Sheth, Sarah Nobles, Jasmine Padilla, Mark Burroughs, Shoshona Le, Katie Dillon, Peter Cook, Clinton R. Paden, Dhwani Batra, Krista Queen, Kristen Knipe, Dakota Howard, Yvette Unoarumhi, Darlene Wagner, Matthew Schmerer, Ben L. Rambo-Martin, Kristine Lacek, Sam Shepard, Alison Laufer Halpin, Dave Wentworth, Vivien Dugan, Suxiang Tong, Justin Lee
hCoV-19/USA/AK-PHL9387/2021	EPI_ISL_2626688	5/25/2021	Alaska State Virology Laboratory	Alaska State Virology Laboratory	Stephanie DeRonde, Elva House, Jacob Zidek, Lisa Smith, Ph.D., Jack Chen, Ph.D.
hCoV-19/USA/AK-PHL9386/2021	EPI_ISL_2626687	5/25/2021	Alaska State Virology Laboratory	Alaska State Virology Laboratory	Stephanie DeRonde, Elva House, Jacob Zidek, Lisa Smith, Ph.D., Jack Chen, Ph.D.
hCoV-19/USA/AK-PHL9407/2021	EPI_ISL_2626700	4/28/2021	Alaska State Virology Laboratory	Alaska State Virology Laboratory	Stephanie DeRonde, Elva House, Jacob Zidek, Lisa Smith, Ph.D., Jack Chen, Ph.D.
hCoV-19/USA/AK-PHL9409/2021	EPI_ISL_2626701	5/4/2021	Alaska State Virology Laboratory	Alaska State Virology Laboratory	Stephanie DeRonde, Elva House, Jacob Zidek, Lisa Smith, Ph.D., Jack Chen, Ph.D.
hCoV-19/USA/AK-PHL9426/2021	EPI_ISL_2626707	6/2/2021	Alaska State Virology Laboratory	Alaska State Virology Laboratory	Stephanie DeRonde, Elva House, Jacob Zidek, Lisa Smith, Ph.D., Jack Chen, Ph.D.
hCoV-19/USA/AK-PHL9421/2021	EPI_ISL_2626702	6/8/2021	Alaska State Virology Laboratory	Alaska State Virology Laboratory	Stephanie DeRonde, Elva House, Jacob Zidek, Lisa Smith, Ph.D., Jack Chen, Ph.D.
hCoV-19/USA/AK-PHL9427/2021	EPI_ISL_2626708	6/3/2021	Alaska State Virology Laboratory	Alaska State Virology Laboratory	Stephanie DeRonde, Elva House, Jacob Zidek, Lisa Smith, Ph.D., Jack Chen, Ph.D.
hCoV-19/USA/AK-PHL9616/2021	EPI_ISL_2626712	6/7/2021	Alaska State Virology Laboratory	Alaska State Virology Laboratory	Stephanie DeRonde, Elva House, Jacob Zidek, Lisa Smith, Ph.D., Jack Chen, Ph.D.
hCoV-19/USA/AK-PHL9617/2021	EPI_ISL_2626713	6/6/2021	Alaska State Virology Laboratory	Alaska State Virology Laboratory	Stephanie DeRonde, Elva House, Jacob Zidek, Lisa Smith, Ph.D., Jack Chen, Ph.D.
hCoV-19/USA/AK-PHL9618/2021	EPI_ISL_2626714	6/7/2021	Alaska State Virology Laboratory	Alaska State Virology Laboratory	Stephanie DeRonde, Elva House, Jacob Zidek, Lisa Smith, Ph.D., Jack Chen, Ph.D.
hCoV-19/USA/AK-PHL9621/2021	EPI_ISL_2626717	6/6/2021	Alaska State Virology Laboratory	Alaska State Virology Laboratory	Stephanie DeRonde, Elva House, Jacob Zidek, Lisa Smith, Ph.D., Jack Chen, Ph.D.
hCoV-19/USA/AK-PHL9622/2021	EPI_ISL_2626718	6/7/2021	Alaska State Virology Laboratory	Alaska State Virology Laboratory	Stephanie DeRonde, Elva House, Jacob Zidek, Lisa Smith, Ph.D., Jack Chen, Ph.D.
hCoV-19/USA/AK-PHL9624/2021	EPI_ISL_2626720	6/7/2021	Alaska State Virology Laboratory	Alaska State Virology Laboratory	Stephanie DeRonde, Elva House, Jacob Zidek, Lisa Smith, Ph.D., Jack Chen, Ph.D.
hCoV-19/USA/AK-PHL9629/2021	EPI_ISL_2626725	6/8/2021	Alaska State Virology Laboratory	Alaska State Virology Laboratory	Stephanie DeRonde, Elva House, Jacob Zidek, Lisa Smith, Ph.D., Jack Chen, Ph.D.
hCoV-19/USA/AK-PHL9642/2021	EPI_ISL_2626738	6/12/2021	Alaska State Virology Laboratory	Alaska State Virology Laboratory	Stephanie DeRonde, Elva House, Jacob Zidek, Lisa Smith, Ph.D., Jack Chen, Ph.D.
hCoV-19/USA/AK-PHL9643/2021	EPI_ISL_2626739	6/11/2021	Alaska State Virology Laboratory	Alaska State Virology Laboratory	Stephanie DeRonde, Elva House, Jacob Zidek, Lisa Smith, Ph.D., Jack Chen, Ph.D.
hCoV-19/USA/AK-PHL9644/2021	EPI_ISL_2626740	6/11/2021	Alaska State Virology Laboratory	Alaska State Virology Laboratory	Stephanie DeRonde, Elva House, Jacob Zidek, Lisa Smith, Ph.D., Jack Chen, Ph.D.

hCoV-19/USA/AK-PHL9654/2021	EPI_ISL_2626749	6/13/2021	Alaska State Virology Laboratory	Alaska State Virology Laboratory	Stephanie DeRonde, Elva House, Jacob Zidek, Lisa Smith, Ph.D., Jack Chen, Ph.D.
hCoV-19/USA/AK-PHL9661/2021	EPI_ISL_2626755	6/13/2021	Alaska State Virology Laboratory	Alaska State Virology Laboratory	Stephanie DeRonde, Elva House, Jacob Zidek, Lisa Smith, Ph.D., Jack Chen, Ph.D.
hCoV-19/USA/AK-CDC-ASC210034741/2021	EPI_ISL_2042495	4/26/2021	Aegis Sciences Corporation	Centers for Disease Control and Prevention Division of Viral Diseases, Pathogen Discovery	Dakota Howard, Dhwani Batra, Peter W. Cook, Kara Moser, Adrian Paskey, Jason Caravas, Benjamin Rambo-Martin, Shatavia Morrison, Christopher Gulvick, Scott Sammons, Yvette Unoarumhi, Darlene Wagner, Matthew Schmerer, Cyndi Clark, Patrick Campbell, Rob Case, Vikramsinha Ghorpade, Holly Houdeshell, Ola Kvalvaag, Dillon Nall, Ethan Sanders, Alec Vest, Shaun Westlund, Matthew Hardison, Clinton R. Paden, Duncan MacCannell
hCoV-19/USA/AK-PHL8646/2021	EPI_ISL_2213169	5/4/2021	Alaska State Virology Laboratory	Alaska State Virology Laboratory	Stephanie DeRonde, Elva House, Jacob Zidek, Lisa Smith, Ph.D., Jack Chen, Ph.D.
hCoV-19/USA/AK-PHL8664/2021	EPI_ISL_2213201	4/22/2021	Alaska State Virology Laboratory	Alaska State Virology Laboratory	Stephanie DeRonde, Elva House, Jacob Zidek, Lisa Smith, Ph.D., Jack Chen, Ph.D.
hCoV-19/USA/AK-CDC-LC0056155/2021	EPI_ISL_2046452	4/17/2021	Laboratory Corporation of America	Centers for Disease Control and Prevention Division of Viral Diseases, Pathogen Discovery	Dakota Howard, Dhwani Batra, Peter W. Cook, Kara Moser, Adrian Paskey, Jason Caravas, Benjamin Rambo-Martin, Shatavia Morrison, Christopher Gulvick, Scott Sammons, Yvette Unoarumhi, Darlene Wagner, Matthew Schmerer, Minoo Agarwal, Eyad Almasri, Debbie Boles, Ayla Burns, Nuthawin Charoensri, Oren Cohen, Susan Countryman, Mary Ann Cristobal, Bobbi Croy, Suzanne Dale, Hrushikesh Deshmukh, Amanda Douglas, Vincent Drouillon, Marcia Eisenberg, Howard Engler, Rama Ghatti, Prashant Gupta, Susan Hicks, Jake Humphrey, Lax Iyer, Manoj Jain, Mohan Kolli, Brian Krueger, Tim Kuphal, Stanley Letovsky, Michael Levandoski, Craig Lukasik, Jonathan Meltzer, Brian Norvell, Mindy Nye, Scott Parker, Christos Petropoulos, John Pruitt, Steven Ragan, Scott Ryan, Mike Sapeta, Jana Schroth, Suresh Babu Selvaraju, Goran Stevovic, Amanda Suchanek, Andrea Throop, Lyndon Tilson, Thomas Urban, Joe Voshell, Kimberly Wagner, Jonathan Williams, Mary Williamson, Qian Zeng, Tricia Zwiefelhofer, Clinton R. Paden, Duncan MacCannell
hCoV-19/USA/AK-PHL8442/2021	EPI_ISL_2429085	4/26/2021	Alaska State Virology Laboratory	Alaska State Virology Laboratory	Stephanie DeRonde, Elva House, Jacob Zidek, Lisa Smith, Ph.D., Jack Chen, Ph.D.
hCoV-19/USA/AK-PHL8445/2021	EPI_ISL_2429088	4/26/2021	Alaska State Virology Laboratory	Alaska State Virology Laboratory	Stephanie DeRonde, Elva House, Jacob Zidek, Lisa Smith, Ph.D., Jack Chen, Ph.D.
hCoV-19/USA/AK-PHL8446/2021	EPI_ISL_2429089	4/26/2021	Alaska State Virology Laboratory	Alaska State Virology Laboratory	Stephanie DeRonde, Elva House, Jacob Zidek, Lisa Smith, Ph.D., Jack Chen, Ph.D.
hCoV-19/USA/AK-PHL8452/2021	EPI_ISL_2429095	4/27/2021	Alaska State Virology Laboratory	Alaska State Virology Laboratory	Stephanie DeRonde, Elva House, Jacob Zidek, Lisa Smith, Ph.D., Jack Chen, Ph.D.
hCoV-19/USA/AK-PHL8461/2021	EPI_ISL_2429104	4/26/2021	Alaska State Virology Laboratory	Alaska State Virology Laboratory	Stephanie DeRonde, Elva House, Jacob Zidek, Lisa Smith, Ph.D., Jack Chen, Ph.D.
hCoV-19/USA/AK-PHL8462/2021	EPI_ISL_2429105	4/26/2021	Alaska State Virology Laboratory	Alaska State Virology Laboratory	Stephanie DeRonde, Elva House, Jacob Zidek, Lisa Smith, Ph.D., Jack Chen, Ph.D.
hCoV-19/USA/AK-PHL8465/2021	EPI_ISL_2429108	4/18/2021	Alaska State Virology Laboratory	Alaska State Virology Laboratory	Stephanie DeRonde, Elva House, Jacob Zidek, Lisa Smith, Ph.D., Jack Chen, Ph.D.
hCoV-19/USA/AK-PHL8468/2021	EPI_ISL_2429111	4/22/2021	Alaska State Virology Laboratory	Alaska State Virology Laboratory	Stephanie DeRonde, Elva House, Jacob Zidek, Lisa Smith, Ph.D., Jack Chen, Ph.D.
hCoV-19/USA/AK-PHL8471/2021	EPI_ISL_2429114	4/18/2021	Alaska State Virology Laboratory	Alaska State Virology Laboratory	Stephanie DeRonde, Elva House, Jacob Zidek, Lisa Smith, Ph.D., Jack Chen, Ph.D.
hCoV-19/USA/AK-PHL8472/2021	EPI_ISL_2429115	4/19/2021	Alaska State Virology Laboratory	Alaska State Virology Laboratory	Stephanie DeRonde, Elva House, Jacob Zidek, Lisa Smith, Ph.D., Jack Chen, Ph.D.
hCoV-19/USA/AK-PHL8474/2021	EPI_ISL_2429117	4/22/2021	Alaska State Virology Laboratory	Alaska State Virology Laboratory	Stephanie DeRonde, Elva House, Jacob Zidek, Lisa Smith, Ph.D., Jack Chen, Ph.D.
hCoV-19/USA/AK-PHL8685/2021	EPI_ISL_2136431	5/6/2021	Alaska State Virology Laboratory	Alaska State Virology Laboratory	Stephanie DeRonde, Elva House, Jacob Zidek, Lisa Smith, Ph.D., Jack Chen, Ph.D.
hCoV-19/USA/AK-PHL8686/2021	EPI_ISL_2136432	5/6/2021	Alaska State Virology Laboratory	Alaska State Virology Laboratory	Stephanie DeRonde, Elva House, Jacob Zidek, Lisa Smith, Ph.D., Jack Chen, Ph.D.
hCoV-19/USA/AK-PHL8687/2021	EPI_ISL_2136433	5/6/2021	Alaska State Virology Laboratory	Alaska State Virology Laboratory	Stephanie DeRonde, Elva House, Jacob Zidek, Lisa Smith, Ph.D., Jack Chen, Ph.D.
hCoV-19/USA/AK-PHL8692/2021	EPI_ISL_2136437	5/6/2021	Alaska State Virology Laboratory	Alaska State Virology Laboratory	Stephanie DeRonde, Elva House, Jacob Zidek, Lisa Smith, Ph.D., Jack Chen, Ph.D.
hCoV-19/USA/AK-PHL8696/2021	EPI_ISL_2136441	5/6/2021	Alaska State Virology Laboratory	Alaska State Virology Laboratory	Stephanie DeRonde, Elva House, Jacob Zidek, Lisa Smith, Ph.D., Jack Chen, Ph.D.
hCoV-19/USA/AK-PHL8693/2021	EPI_ISL_2136438	5/6/2021	Alaska State Virology Laboratory	Alaska State Virology Laboratory	Stephanie DeRonde, Elva House, Jacob Zidek, Lisa Smith, Ph.D., Jack Chen, Ph.D.
hCoV-19/USA/AK-PHL8694/2021	EPI_ISL_2136439	5/6/2021	Alaska State Virology Laboratory	Alaska State Virology Laboratory	Stephanie DeRonde, Elva House, Jacob Zidek, Lisa Smith, Ph.D., Jack Chen, Ph.D.
hCoV-19/USA/AK-PHL8702/2021	EPI_ISL_2136445	5/7/2021	Alaska State Virology Laboratory	Alaska State Virology Laboratory	Stephanie DeRonde, Elva House, Jacob Zidek, Lisa Smith, Ph.D., Jack Chen, Ph.D.
hCoV-19/USA/AK-PHL8724/2021	EPI_ISL_2136465	5/4/2021	Alaska State Virology Laboratory	Alaska State Virology Laboratory	Stephanie DeRonde, Elva House, Jacob Zidek, Lisa Smith, Ph.D., Jack Chen, Ph.D.
hCoV-19/USA/AK-PHL8705/2021	EPI_ISL_2136447	5/8/2021	Alaska State Virology Laboratory	Alaska State Virology Laboratory	Stephanie DeRonde, Elva House, Jacob Zidek, Lisa Smith, Ph.D., Jack Chen, Ph.D.
hCoV-19/USA/AK-PHL8710/2021	EPI_ISL_2136452	5/5/2021	Alaska State Virology Laboratory	Alaska State Virology Laboratory	Stephanie DeRonde, Elva House, Jacob Zidek, Lisa Smith, Ph.D., Jack Chen, Ph.D.
hCoV-19/USA/AK-PHL8720/2021	EPI_ISL_2136461	5/4/2021	Alaska State Virology Laboratory	Alaska State Virology Laboratory	Stephanie DeRonde, Elva House, Jacob Zidek, Lisa Smith, Ph.D., Jack Chen, Ph.D.
hCoV-19/USA/AK-PHL8722/2021	EPI_ISL_2136463	5/5/2021	Alaska State Virology Laboratory	Alaska State Virology Laboratory	Stephanie DeRonde, Elva House, Jacob Zidek, Lisa Smith, Ph.D., Jack Chen, Ph.D.
hCoV-19/USA/AK-PHL8574/2021	EPI_ISL_2136747	5/1/2021	Alaska State Virology Laboratory	Alaska State Virology Laboratory	Stephanie DeRonde, Elva House, Jacob Zidek, Lisa Smith, Ph.D., Jack Chen, Ph.D.
hCoV-19/USA/AK-PHL8590/2021	EPI_ISL_2136762	5/3/2021	Alaska State Virology Laboratory	Alaska State Virology Laboratory	Stephanie DeRonde, Elva House, Jacob Zidek, Lisa Smith, Ph.D., Jack Chen, Ph.D.
hCoV-19/USA/AK-PHL8591/2021	EPI_ISL_2136763	5/3/2021	Alaska State Virology Laboratory	Alaska State Virology Laboratory	Stephanie DeRonde, Elva House, Jacob Zidek, Lisa Smith, Ph.D., Jack Chen, Ph.D.
hCoV-19/USA/AK-PHL8592/2021	EPI_ISL_2136764	5/3/2021	Alaska State Virology Laboratory	Alaska State Virology Laboratory	Stephanie DeRonde, Elva House, Jacob Zidek, Lisa Smith, Ph.D., Jack Chen, Ph.D.
hCoV-19/USA/AK-PHL8594/2021	EPI_ISL_2136766	5/3/2021	Alaska State Virology Laboratory	Alaska State Virology Laboratory	Stephanie DeRonde, Elva House, Jacob Zidek, Lisa Smith, Ph.D., Jack Chen, Ph.D.

hCoV-19/USA/AK-PHL8600/2021	EPI_ISL_2136772	4/28/2021	Alaska State Virology Laboratory	Alaska State Virology Laboratory	Stephanie DeRonde, Elva House, Jacob Zidek, Lisa Smith, Ph.D., Jack Chen, Ph.D.
hCoV-19/USA/AK-PHL8605/2021	EPI_ISL_2136776	4/28/2021	Alaska State Virology Laboratory	Alaska State Virology Laboratory	Stephanie DeRonde, Elva House, Jacob Zidek, Lisa Smith, Ph.D., Jack Chen, Ph.D.
hCoV-19/USA/AK-PHL8607/2021	EPI_ISL_2136777	4/27/2021	Alaska State Virology Laboratory	Alaska State Virology Laboratory	Stephanie DeRonde, Elva House, Jacob Zidek, Lisa Smith, Ph.D., Jack Chen, Ph.D.
hCoV-19/USA/AK-PHL8613/2021	EPI_ISL_2136783	5/3/2021	Alaska State Virology Laboratory	Alaska State Virology Laboratory	Stephanie DeRonde, Elva House, Jacob Zidek, Lisa Smith, Ph.D., Jack Chen, Ph.D.
hCoV-19/USA/AK-PHL8615/2021	EPI_ISL_2136785	4/30/2021	Alaska State Virology Laboratory	Alaska State Virology Laboratory	Stephanie DeRonde, Elva House, Jacob Zidek, Lisa Smith, Ph.D., Jack Chen, Ph.D.
hCoV-19/USA/AK-CDC-2-4504090/2021	EPI_ISL_2689874	5/17/2021	AK State Public Health Lab, State Health Department	Centers for Disease Control and Prevention Division of Viral Diseases, Pathogen Discovery	Mili Sheth, Sarah Nobles, Jasmine Padilla, Mark Burroughs, Shoshona Le, Katie Dillon, Peter Cook, Clinton R. Paden, Dhwani Batra, Krista Queen, Kristen Knipe, Dakota Howard, Yvette Unoarumhi, Darlene Wagner, Matthew Schmerer, Ben L. Rambo-Martin, Kristine Lacek, Sam Shepard, Alison Laufer Halpin, Dave Wentworth, Vivien Dugan, Suxiang Tong, Justin Lee
hCoV-19/USA/AK-CDC-2-4504189/2021	EPI_ISL_2689870	5/17/2021	AK State Public Health Lab, State Health Department	Centers for Disease Control and Prevention Division of Viral Diseases, Pathogen Discovery	Mili Sheth, Sarah Nobles, Jasmine Padilla, Mark Burroughs, Shoshona Le, Katie Dillon, Peter Cook, Clinton R. Paden, Dhwani Batra, Krista Queen, Kristen Knipe, Dakota Howard, Yvette Unoarumhi, Darlene Wagner, Matthew Schmerer, Ben L. Rambo-Martin, Kristine Lacek, Sam Shepard, Alison Laufer Halpin, Dave Wentworth, Vivien Dugan, Suxiang Tong, Justin Lee
hCoV-19/USA/AK-CDC-2-4504160/2021	EPI_ISL_2689871	5/18/2021	AK State Public Health Lab, State Health Department	Centers for Disease Control and Prevention Division of Viral Diseases, Pathogen Discovery	Mili Sheth, Sarah Nobles, Jasmine Padilla, Mark Burroughs, Shoshona Le, Katie Dillon, Peter Cook, Clinton R. Paden, Dhwani Batra, Krista Queen, Kristen Knipe, Dakota Howard, Yvette Unoarumhi, Darlene Wagner, Matthew Schmerer, Ben L. Rambo-Martin, Kristine Lacek, Sam Shepard, Alison Laufer Halpin, Dave Wentworth, Vivien Dugan, Suxiang Tong, Justin Lee
hCoV-19/USA/AK-PHL9217/2021	EPI_ISL_2454562	5/27/2021	Alaska State Virology Laboratory	Alaska State Virology Laboratory	Stephanie DeRonde, Elva House, Jacob Zidek, Lisa Smith, Ph.D., Jack Chen, Ph.D.
hCoV-19/USA/AK-PHL9207/2021	EPI_ISL_2454556	5/26/2021	Alaska State Virology Laboratory	Alaska State Virology Laboratory	Stephanie DeRonde, Elva House, Jacob Zidek, Lisa Smith, Ph.D., Jack Chen, Ph.D.
hCoV-19/USA/AK-PHL9234/2021	EPI_ISL_2454574	5/28/2021	Alaska State Virology Laboratory	Alaska State Virology Laboratory	Stephanie DeRonde, Elva House, Jacob Zidek, Lisa Smith, Ph.D., Jack Chen, Ph.D.
hCoV-19/USA/AK-PHL9236/2021	EPI_ISL_2454576	5/28/2021	Alaska State Virology Laboratory	Alaska State Virology Laboratory	Stephanie DeRonde, Elva House, Jacob Zidek, Lisa Smith, Ph.D., Jack Chen, Ph.D.
hCoV-19/USA/AK-PHL9237/2021	EPI_ISL_2454577	5/29/2021	Alaska State Virology Laboratory	Alaska State Virology Laboratory	Stephanie DeRonde, Elva House, Jacob Zidek, Lisa Smith, Ph.D., Jack Chen, Ph.D.
hCoV-19/USA/AK-PHL9244/2021	EPI_ISL_2454581	5/27/2021	Alaska State Virology Laboratory	Alaska State Virology Laboratory	Stephanie DeRonde, Elva House, Jacob Zidek, Lisa Smith, Ph.D., Jack Chen, Ph.D.
hCoV-19/USA/AK-PHL8569/2021	EPI_ISL_2101751	5/3/2021	Alaska State Virology Laboratory	Alaska State Virology Laboratory	Stephanie DeRonde, Elva House, Jacob Zidek, Lisa Smith, Ph.D., Jack Chen, Ph.D.
hCoV-19/USA/AK-PHL8557/2021	EPI_ISL_2101757	4/30/2021	Alaska State Virology Laboratory	Alaska State Virology Laboratory	Stephanie DeRonde, Elva House, Jacob Zidek, Lisa Smith, Ph.D., Jack Chen, Ph.D.
hCoV-19/USA/AK-PHL8541/2021	EPI_ISL_2101765	4/13/2021	Alaska State Virology Laboratory	Alaska State Virology Laboratory	Stephanie DeRonde, Elva House, Jacob Zidek, Lisa Smith, Ph.D., Jack Chen, Ph.D.
hCoV-19/USA/AK-PHL8554/2021	EPI_ISL_2101769	4/29/2021	Alaska State Virology Laboratory	Alaska State Virology Laboratory	Stephanie DeRonde, Elva House, Jacob Zidek, Lisa Smith, Ph.D., Jack Chen, Ph.D.
hCoV-19/USA/AK-PHL8828/2021	EPI_ISL_2272525	5/12/2021	Alaska State Virology Laboratory	Alaska State Virology Laboratory	Stephanie DeRonde, Elva House, Jacob Zidek, Lisa Smith, Ph.D., Jack Chen, Ph.D.
hCoV-19/USA/AK-PHL8845/2021	EPI_ISL_2272539	5/11/2021	Alaska State Virology Laboratory	Alaska State Virology Laboratory	Stephanie DeRonde, Elva House, Jacob Zidek, Lisa Smith, Ph.D., Jack Chen, Ph.D.
hCoV-19/USA/AK-PHL8836/2021	EPI_ISL_2272531	5/12/2021	Alaska State Virology Laboratory	Alaska State Virology Laboratory	Stephanie DeRonde, Elva House, Jacob Zidek, Lisa Smith, Ph.D., Jack Chen, Ph.D.
hCoV-19/USA/AK-PHL8852/2021	EPI_ISL_2272544	5/12/2021	Alaska State Virology Laboratory	Alaska State Virology Laboratory	Stephanie DeRonde, Elva House, Jacob Zidek, Lisa Smith, Ph.D., Jack Chen, Ph.D.
hCoV-19/USA/AK-PHL8853/2021	EPI_ISL_2272545	5/12/2021	Alaska State Virology Laboratory	Alaska State Virology Laboratory	Stephanie DeRonde, Elva House, Jacob Zidek, Lisa Smith, Ph.D., Jack Chen, Ph.D.
hCoV-19/USA/AK-CDC-2-4449385/2021	EPI_ISL_2451942	5/16/2021	AK State Public Health Lab, State Health Department	Centers for Disease Control and Prevention Division of Viral Diseases, Pathogen Discovery	Mili Sheth, Sarah Nobles, Jasmine Padilla, Mark Burroughs, Shoshona Le, Katie Dillon, Peter Cook, Clinton R. Paden, Dhwani Batra, Krista Queen, Kristen Knipe, Dakota Howard, Yvette Unoarumhi, Darlene Wagner, Matthew Schmerer, Ben L. Rambo-Martin, Kristine Lacek, Sam Shepard, Alison Laufer Halpin, Dave Wentworth, Vivien Dugan, Suxiang Tong, Justin Lee
hCoV-19/USA/AK-PHL7921/2021	EPI_ISL_2227458	4/3/2021	Alaska State Virology Laboratory	Alaska State Virology Laboratory	Stephanie DeRonde, Elva House, Lisa Smith, Ph.D., Jack Chen, Ph.D.
hCoV-19/USA/AK-PHL8662/2021	EPI_ISL_2227464	5/4/2021	Alaska State Virology Laboratory	Alaska State Virology Laboratory	Stephanie DeRonde, Elva House, Jacob Zidek, Lisa Smith, Ph.D., Jack Chen, Ph.D.
hCoV-19/USA/AK-PHL7935/2021	EPI_ISL_2230702	4/2/2021	Alaska State Virology Laboratory	Alaska State Virology Laboratory	Stephanie DeRonde, Elva House, Lisa Smith, Ph.D., Jack Chen, Ph.D.
hCoV-19/USA/AK-PHL9111/2021	EPI_ISL_2421685	5/23/2021	Alaska State Virology Laboratory	Alaska State Virology Laboratory	Stephanie DeRonde, Elva House, Jacob Zidek, Lisa Smith, Ph.D., Jack Chen, Ph.D.
hCoV-19/USA/AK-PHL9120/2021	EPI_ISL_2421699	5/24/2021	Alaska State Virology Laboratory	Alaska State Virology Laboratory	Stephanie DeRonde, Elva House, Jacob Zidek, Lisa Smith, Ph.D., Jack Chen, Ph.D.
hCoV-19/USA/AK-PHL9113/2021	EPI_ISL_2421688	5/23/2021	Alaska State Virology Laboratory	Alaska State Virology Laboratory	Stephanie DeRonde, Elva House, Jacob Zidek, Lisa Smith, Ph.D., Jack Chen, Ph.D.
hCoV-19/USA/AK-PHL9117/2021	EPI_ISL_2421694	5/24/2021	Alaska State Virology Laboratory	Alaska State Virology Laboratory	Stephanie DeRonde, Elva House, Jacob Zidek, Lisa Smith, Ph.D., Jack Chen, Ph.D.
hCoV-19/USA/AK-PHL9118/2021	EPI_ISL_2421696	5/24/2021	Alaska State Virology Laboratory	Alaska State Virology Laboratory	Stephanie DeRonde, Elva House, Jacob Zidek, Lisa Smith, Ph.D., Jack Chen, Ph.D.
hCoV-19/USA/AK-PHL9138/2021	EPI_ISL_2421714	5/2/2021	Alaska State Virology Laboratory	Alaska State Virology Laboratory	Stephanie DeRonde, Elva House, Jacob Zidek, Lisa Smith, Ph.D., Jack Chen, Ph.D.
hCoV-19/USA/AK-PHL9124/2021	EPI_ISL_2421701	5/21/2021	Alaska State Virology Laboratory	Alaska State Virology Laboratory	Stephanie DeRonde, Elva House, Jacob Zidek, Lisa Smith, Ph.D., Jack Chen, Ph.D.
hCoV-19/USA/AK-PHL9125/2021	EPI_ISL_2421703	5/21/2021	Alaska State Virology Laboratory	Alaska State Virology Laboratory	Stephanie DeRonde, Elva House, Jacob Zidek, Lisa Smith, Ph.D., Jack Chen, Ph.D.
hCoV-19/USA/AK-PHL9135/2021	EPI_ISL_2421712	5/24/2021	Alaska State Virology Laboratory	Alaska State Virology Laboratory	Stephanie DeRonde, Elva House, Jacob Zidek, Lisa Smith, Ph.D., Jack Chen, Ph.D.
hCoV-19/USA/AK-PHL9139/2021	EPI_ISL_2421715	4/12/2021	Alaska State Virology Laboratory	Alaska State Virology Laboratory	Stephanie DeRonde, Elva House, Jacob Zidek, Lisa Smith, Ph.D., Jack Chen, Ph.D.

hCoV-19/USA/AK-PHL9146/2021	EPI_ISL_2421721	5/4/2021	Alaska State Virology Laboratory	Alaska State Virology Laboratory	Stephanie DeRonde, Elva House, Jacob Zidek, Lisa Smith, Ph.D., Jack Chen, Ph.D.
hCoV-19/USA/AK-PHL9148/2021	EPI_ISL_2421723	5/10/2021	Alaska State Virology Laboratory	Alaska State Virology Laboratory	Stephanie DeRonde, Elva House, Jacob Zidek, Lisa Smith, Ph.D., Jack Chen, Ph.D.
hCoV-19/USA/AK-PHL9150/2021	EPI_ISL_2421724	5/7/2021	Alaska State Virology Laboratory	Alaska State Virology Laboratory	Stephanie DeRonde, Elva House, Jacob Zidek, Lisa Smith, Ph.D., Jack Chen, Ph.D.
hCoV-19/USA/AK-PHL8923/2021	EPI_ISL_2422060	5/15/2021	Alaska State Virology Laboratory	Alaska State Virology Laboratory	Stephanie DeRonde, Elva House, Jacob Zidek, Lisa Smith, Ph.D., Jack Chen, Ph.D.
hCoV-19/USA/AK-PHL9154/2021	EPI_ISL_2421727	5/20/2021	Alaska State Virology Laboratory	Alaska State Virology Laboratory	Stephanie DeRonde, Elva House, Jacob Zidek, Lisa Smith, Ph.D., Jack Chen, Ph.D.
hCoV-19/USA/AK-PHL8914/2021	EPI_ISL_2422048	5/16/2021	Alaska State Virology Laboratory	Alaska State Virology Laboratory	Stephanie DeRonde, Elva House, Jacob Zidek, Lisa Smith, Ph.D., Jack Chen, Ph.D.
hCoV-19/USA/AK-PHL8915/2021	EPI_ISL_2422050	5/16/2021	Alaska State Virology Laboratory	Alaska State Virology Laboratory	Stephanie DeRonde, Elva House, Jacob Zidek, Lisa Smith, Ph.D., Jack Chen, Ph.D.
hCoV-19/USA/AK-PHL8933/2021	EPI_ISL_2422075	5/17/2021	Alaska State Virology Laboratory	Alaska State Virology Laboratory	Stephanie DeRonde, Elva House, Jacob Zidek, Lisa Smith, Ph.D., Jack Chen, Ph.D.
hCoV-19/USA/AK-PHL8938/2021	EPI_ISL_2422083	5/17/2021	Alaska State Virology Laboratory	Alaska State Virology Laboratory	Stephanie DeRonde, Elva House, Jacob Zidek, Lisa Smith, Ph.D., Jack Chen, Ph.D.
hCoV-19/USA/AK-PHL8939/2021	EPI_ISL_2422085	5/17/2021	Alaska State Virology Laboratory	Alaska State Virology Laboratory	Stephanie DeRonde, Elva House, Jacob Zidek, Lisa Smith, Ph.D., Jack Chen, Ph.D.
hCoV-19/USA/AK-PHL8942/2021	EPI_ISL_2422089	5/16/2021	Alaska State Virology Laboratory	Alaska State Virology Laboratory	Stephanie DeRonde, Elva House, Jacob Zidek, Lisa Smith, Ph.D., Jack Chen, Ph.D.
hCoV-19/USA/AK-PHL8945/2021	EPI_ISL_2422094	5/17/2021	Alaska State Virology Laboratory	Alaska State Virology Laboratory	Stephanie DeRonde, Elva House, Jacob Zidek, Lisa Smith, Ph.D., Jack Chen, Ph.D.
hCoV-19/USA/AK-PHL8949/2021	EPI_ISL_2422097	5/16/2021	Alaska State Virology Laboratory	Alaska State Virology Laboratory	Stephanie DeRonde, Elva House, Jacob Zidek, Lisa Smith, Ph.D., Jack Chen, Ph.D.
hCoV-19/USA/AK-PHL8951/2021	EPI_ISL_2422100	5/17/2021	Alaska State Virology Laboratory	Alaska State Virology Laboratory	Stephanie DeRonde, Elva House, Jacob Zidek, Lisa Smith, Ph.D., Jack Chen, Ph.D.
hCoV-19/USA/AK-PHL8953/2021	EPI_ISL_2422103	5/14/2021	Alaska State Virology Laboratory	Alaska State Virology Laboratory	Stephanie DeRonde, Elva House, Jacob Zidek, Lisa Smith, Ph.D., Jack Chen, Ph.D.
hCoV-19/USA/AK-PHL8957/2021	EPI_ISL_2422106	5/17/2021	Alaska State Virology Laboratory	Alaska State Virology Laboratory	Stephanie DeRonde, Elva House, Jacob Zidek, Lisa Smith, Ph.D., Jack Chen, Ph.D.
hCoV-19/USA/AK-PHL9147/2021	EPI_ISL_2423416	3/31/2021	Alaska State Virology Laboratory	Alaska State Virology Laboratory	Stephanie DeRonde, Elva House, Jacob Zidek, Lisa Smith, Ph.D., Jack Chen, Ph.D.
hCoV-19/USA/AK-PHL9153/2021	EPI_ISL_2423417	5/21/2021	Alaska State Virology Laboratory	Alaska State Virology Laboratory	Stephanie DeRonde, Elva House, Jacob Zidek, Lisa Smith, Ph.D., Jack Chen, Ph.D.
hCoV-19/USA/AK-PHL6558/2020	EPI_ISL_2348955	12/8/2020	Alaska State Virology Lab, Alaska State Public Health Labs	Alaska State Virology Lab, Alaska State Public Health Labs	DeRonde,S., House,E., Zidek,J., Redlinger,M., Smith,L., Chen,J.
hCoV-19/USA/AK-PHL9018/2021	EPI_ISL_2375802	5/18/2021	Alaska State Virology Laboratory	Alaska State Virology Laboratory	Stephanie DeRonde, Elva House, Jacob Zidek, Lisa Smith, Ph.D., Jack Chen, Ph.D.
hCoV-19/USA/AK-PHL9250/2021	EPI_ISL_2455419	5/30/2021	Alaska State Virology Laboratory	Alaska State Virology Laboratory	Stephanie DeRonde, Elva House, Jacob Zidek, Lisa Smith, Ph.D., Jack Chen, Ph.D.
hCoV-19/USA/AK-PHL9253/2021	EPI_ISL_2455422	5/24/2021	Alaska State Virology Laboratory	Alaska State Virology Laboratory	Stephanie DeRonde, Elva House, Jacob Zidek, Lisa Smith, Ph.D., Jack Chen, Ph.D.
hCoV-19/USA/AK-PHL9255/2021	EPI_ISL_2455423	5/22/2021	Alaska State Virology Laboratory	Alaska State Virology Laboratory	Stephanie DeRonde, Elva House, Jacob Zidek, Lisa Smith, Ph.D., Jack Chen, Ph.D.
hCoV-19/USA/AK-PHL9258/2021	EPI_ISL_2455424	5/19/2021	Alaska State Virology Laboratory	Alaska State Virology Laboratory	Stephanie DeRonde, Elva House, Jacob Zidek, Lisa Smith, Ph.D., Jack Chen, Ph.D.
hCoV-19/USA/AK-PHL9259/2021	EPI_ISL_2455425	5/22/2021	Alaska State Virology Laboratory	Alaska State Virology Laboratory	Stephanie DeRonde, Elva House, Jacob Zidek, Lisa Smith, Ph.D., Jack Chen, Ph.D.
hCoV-19/USA/AK-PHL9260/2021	EPI_ISL_2455426	5/22/2021	Alaska State Virology Laboratory	Alaska State Virology Laboratory	Stephanie DeRonde, Elva House, Jacob Zidek, Lisa Smith, Ph.D., Jack Chen, Ph.D.
hCoV-19/USA/AK-PHL9271/2021	EPI_ISL_2455435	5/29/2021	Alaska State Virology Laboratory	Alaska State Virology Laboratory	Stephanie DeRonde, Elva House, Jacob Zidek, Lisa Smith, Ph.D., Jack Chen, Ph.D.
hCoV-19/USA/AK-PHL9277/2021	EPI_ISL_2455438	5/9/2021	Alaska State Virology Laboratory	Alaska State Virology Laboratory	Stephanie DeRonde, Elva House, Jacob Zidek, Lisa Smith, Ph.D., Jack Chen, Ph.D.
hCoV-19/USA/AK-PHL9278/2021	EPI_ISL_2455439	5/5/2021	Alaska State Virology Laboratory	Alaska State Virology Laboratory	Stephanie DeRonde, Elva House, Jacob Zidek, Lisa Smith, Ph.D., Jack Chen, Ph.D.
hCoV-19/USA/AK-PHL9290/2021	EPI_ISL_2455449	5/5/2021	Alaska State Virology Laboratory	Alaska State Virology Laboratory	Stephanie DeRonde, Elva House, Jacob Zidek, Lisa Smith, Ph.D., Jack Chen, Ph.D.
hCoV-19/USA/AK-PHL8578/2021	EPI_ISL_2499752	4/30/2021	Alaska State Virology Laboratory	Alaska State Virology Laboratory	Stephanie DeRonde, Elva House, Jacob Zidek, Lisa Smith, Ph.D., Jack Chen, Ph.D.
hCoV-19/USA/AK-PHL8533/2021	EPI_ISL_2499778	5/1/2021	Alaska State Virology Laboratory	Alaska State Virology Laboratory	Stephanie DeRonde, Elva House, Jacob Zidek, Lisa Smith, Ph.D., Jack Chen, Ph.D.
hCoV-19/USA/AK-PHL8997/2021	EPI_ISL_2499927	5/13/2021	Alaska State Virology Laboratory	Alaska State Virology Laboratory	Stephanie DeRonde, Elva House, Jacob Zidek, Lisa Smith, Ph.D., Jack Chen, Ph.D.
hCoV-19/USA/AK-PHL8365/2021	EPI_ISL_2499918	4/23/2021	Alaska State Virology Laboratory	Alaska State Virology Laboratory	Stephanie DeRonde, Elva House, Jacob Zidek, Lisa Smith, Ph.D., Jack Chen, Ph.D.
hCoV-19/USA/AK-PHL8963/2021	EPI_ISL_2499923	5/14/2021	Alaska State Virology Laboratory	Alaska State Virology Laboratory	Stephanie DeRonde, Elva House, Jacob Zidek, Lisa Smith, Ph.D., Jack Chen, Ph.D.
hCoV-19/USA/AK-PHL8827/2021	EPI_ISL_2499928	5/11/2021	Alaska State Virology Laboratory	Alaska State Virology Laboratory	Stephanie DeRonde, Elva House, Jacob Zidek, Lisa Smith, Ph.D., Jack Chen, Ph.D.
hCoV-19/USA/AK-PHL8134/2021	EPI_ISL_2499931	4/15/2021	Alaska State Virology Laboratory	Alaska State Virology Laboratory	Stephanie DeRonde, Elva House, Jacob Zidek, Lisa Smith, Ph.D., Jack Chen, Ph.D.
hCoV-19/USA/AK-PHL9342/2021	EPI_ISL_2503978	6/1/2021	Alaska State Virology Laboratory	Alaska State Virology Laboratory	Stephanie DeRonde, Elva House, Jacob Zidek, Lisa Smith, Ph.D., Jack Chen, Ph.D.
hCoV-19/USA/AK-PHL9343/2021	EPI_ISL_2503979	6/1/2021	Alaska State Virology Laboratory	Alaska State Virology Laboratory	Stephanie DeRonde, Elva House, Jacob Zidek, Lisa Smith, Ph.D., Jack Chen, Ph.D.
hCoV-19/USA/AK-PHL9346/2021	EPI_ISL_2503982	6/1/2021	Alaska State Virology Laboratory	Alaska State Virology Laboratory	Stephanie DeRonde, Elva House, Jacob Zidek, Lisa Smith, Ph.D., Jack Chen, Ph.D.
hCoV-19/USA/AK-PHL9350/2021	EPI_ISL_2503984	6/2/2021	Alaska State Virology Laboratory	Alaska State Virology Laboratory	Stephanie DeRonde, Elva House, Jacob Zidek, Lisa Smith, Ph.D., Jack Chen, Ph.D.
hCoV-19/USA/AK-PHL9352/2021	EPI_ISL_2503986	6/1/2021	Alaska State Virology Laboratory	Alaska State Virology Laboratory	Stephanie DeRonde, Elva House, Jacob Zidek, Lisa Smith, Ph.D., Jack Chen, Ph.D.
hCoV-19/USA/AK-PHL9354/2021	EPI_ISL_2503988	6/2/2021	Alaska State Virology Laboratory	Alaska State Virology Laboratory	Stephanie DeRonde, Elva House, Jacob Zidek, Lisa Smith, Ph.D., Jack Chen, Ph.D.
hCoV-19/USA/AK-PHL9359/2021	EPI_ISL_2503991	6/2/2021	Alaska State Virology Laboratory	Alaska State Virology Laboratory	Stephanie DeRonde, Elva House, Jacob Zidek, Lisa Smith, Ph.D., Jack Chen, Ph.D.
hCoV-19/USA/AK-PHL9363/2021	EPI_ISL_2503994	5/30/2021	Alaska State Virology Laboratory	Alaska State Virology Laboratory	Stephanie DeRonde, Elva House, Jacob Zidek, Lisa Smith, Ph.D., Jack Chen, Ph.D.
hCoV-19/USA/AK-PHL9372/2021	EPI_ISL_2504002	6/4/2021	Alaska State Virology Laboratory	Alaska State Virology Laboratory	Stephanie DeRonde, Elva House, Jacob Zidek, Lisa Smith, Ph.D., Jack Chen, Ph.D.
hCoV-19/USA/AK-PHL9373/2021	EPI_ISL_2504003	6/4/2021	Alaska State Virology Laboratory	Alaska State Virology Laboratory	Stephanie DeRonde, Elva House, Jacob Zidek, Lisa Smith, Ph.D., Jack Chen, Ph.D.
hCoV-19/USA/AK-PHL9378/2021	EPI_ISL_2504007	6/3/2021	Alaska State Virology Laboratory	Alaska State Virology Laboratory	Stephanie DeRonde, Elva House, Jacob Zidek, Lisa Smith, Ph.D., Jack Chen, Ph.D.
hCoV-19/USA/AK-PHL9375/2021	EPI_ISL_2504005	6/2/2021	Alaska State Virology Laboratory	Alaska State Virology Laboratory	Stephanie DeRonde, Elva House, Jacob Zidek, Lisa Smith, Ph.D., Jack Chen, Ph.D.
hCoV-19/USA/AK-PHL9385/2021	EPI_ISL_2504013	5/6/2021	Alaska State Virology Laboratory	Alaska State Virology Laboratory	Stephanie DeRonde, Elva House, Jacob Zidek, Lisa Smith, Ph.D., Jack Chen, Ph.D.
hCoV-19/USA/AK-PHL6684/2020	EPI_ISL_2716844	12/30/2020	Alaska State Virology Laboratory	Alaska State Virology Laboratory	Stephanie DeRonde, Elva House, Jacob Zidek, Lisa Smith, Ph.D., Jack Chen, Ph.D.

hCoV-19/USA/AK-PHL6685/2020	EPI_ISL_2716845	12/23/2020	Alaska State Virology Laboratory	Alaska State Virology Laboratory	Stephanie DeRonde, Elva House, Jacob Zidek, Lisa Smith, Ph.D., Jack Chen, Ph.D.
hCoV-19/USA/AK-PHL6686/2021	EPI_ISL_2716846	1/4/2021	Alaska State Virology Laboratory	Alaska State Virology Laboratory	Stephanie DeRonde, Elva House, Jacob Zidek, Lisa Smith, Ph.D., Jack Chen, Ph.D.
hCoV-19/USA/AK-PHL6687/2020	EPI_ISL_2716847	12/29/2020	Alaska State Virology Laboratory	Alaska State Virology Laboratory	Stephanie DeRonde, Elva House, Jacob Zidek, Lisa Smith, Ph.D., Jack Chen, Ph.D.
hCoV-19/USA/AK-PHL6688/2021	EPI_ISL_2716848	1/6/2021	Alaska State Virology Laboratory	Alaska State Virology Laboratory	Stephanie DeRonde, Elva House, Jacob Zidek, Lisa Smith, Ph.D., Jack Chen, Ph.D.
hCoV-19/USA/AK-PHL6689/2020	EPI_ISL_2716849	12/26/2020	Alaska State Virology Laboratory	Alaska State Virology Laboratory	Stephanie DeRonde, Elva House, Jacob Zidek, Lisa Smith, Ph.D., Jack Chen, Ph.D.
hCoV-19/USA/AK-PHL6690/2021	EPI_ISL_2716850	1/5/2021	Alaska State Virology Laboratory	Alaska State Virology Laboratory	Stephanie DeRonde, Elva House, Jacob Zidek, Lisa Smith, Ph.D., Jack Chen, Ph.D.
hCoV-19/USA/AK-PHL6691/2021	EPI_ISL_2716851	1/7/2021	Alaska State Virology Laboratory	Alaska State Virology Laboratory	Stephanie DeRonde, Elva House, Jacob Zidek, Lisa Smith, Ph.D., Jack Chen, Ph.D.
hCoV-19/USA/AK-PHL6693/2021	EPI_ISL_2716852	1/11/2021	Alaska State Virology Laboratory	Alaska State Virology Laboratory	Stephanie DeRonde, Elva House, Jacob Zidek, Lisa Smith, Ph.D., Jack Chen, Ph.D.
hCoV-19/USA/AK-PHL6694/2021	EPI_ISL_2716853	1/6/2021	Alaska State Virology Laboratory	Alaska State Virology Laboratory	Stephanie DeRonde, Elva House, Jacob Zidek, Lisa Smith, Ph.D., Jack Chen, Ph.D.
hCoV-19/USA/AK-PHL6695/2021	EPI_ISL_2716854	1/11/2021	Alaska State Virology Laboratory	Alaska State Virology Laboratory	Stephanie DeRonde, Elva House, Jacob Zidek, Lisa Smith, Ph.D., Jack Chen, Ph.D.
hCoV-19/USA/AK-PHL6696/2021	EPI_ISL_2716855	1/11/2021	Alaska State Virology Laboratory	Alaska State Virology Laboratory	Stephanie DeRonde, Elva House, Jacob Zidek, Lisa Smith, Ph.D., Jack Chen, Ph.D.
hCoV-19/USA/AK-PHL6697/2021	EPI_ISL_2716856	1/4/2021	Alaska State Virology Laboratory	Alaska State Virology Laboratory	Stephanie DeRonde, Elva House, Jacob Zidek, Lisa Smith, Ph.D., Jack Chen, Ph.D.
hCoV-19/USA/AK-PHL6698/2021	EPI_ISL_2716857	1/5/2021	Alaska State Virology Laboratory	Alaska State Virology Laboratory	Stephanie DeRonde, Elva House, Jacob Zidek, Lisa Smith, Ph.D., Jack Chen, Ph.D.
hCoV-19/USA/AK-PHL6700/2021	EPI_ISL_2716858	1/5/2021	Alaska State Virology Laboratory	Alaska State Virology Laboratory	Stephanie DeRonde, Elva House, Jacob Zidek, Lisa Smith, Ph.D., Jack Chen, Ph.D.
hCoV-19/USA/AK-PHL6701/2021	EPI_ISL_2716859	1/8/2021	Alaska State Virology Laboratory	Alaska State Virology Laboratory	Stephanie DeRonde, Elva House, Jacob Zidek, Lisa Smith, Ph.D., Jack Chen, Ph.D.
hCoV-19/USA/AK-PHL6702/2021	EPI_ISL_2716860	1/6/2021	Alaska State Virology Laboratory	Alaska State Virology Laboratory	Stephanie DeRonde, Elva House, Jacob Zidek, Lisa Smith, Ph.D., Jack Chen, Ph.D.
hCoV-19/USA/AK-PHL6703/2021	EPI_ISL_2716861	1/6/2021	Alaska State Virology Laboratory	Alaska State Virology Laboratory	Stephanie DeRonde, Elva House, Jacob Zidek, Lisa Smith, Ph.D., Jack Chen, Ph.D.
hCoV-19/USA/AK-PHL6704/2021	EPI_ISL_2716862	1/8/2021	Alaska State Virology Laboratory	Alaska State Virology Laboratory	Stephanie DeRonde, Elva House, Jacob Zidek, Lisa Smith, Ph.D., Jack Chen, Ph.D.
hCoV-19/USA/AK-PHL6706/2021	EPI_ISL_2716863	1/8/2021	Alaska State Virology Laboratory	Alaska State Virology Laboratory	Stephanie DeRonde, Elva House, Jacob Zidek, Lisa Smith, Ph.D., Jack Chen, Ph.D.
hCoV-19/USA/AK-PHL6707/2021	EPI_ISL_2716864	1/7/2021	Alaska State Virology Laboratory	Alaska State Virology Laboratory	Stephanie DeRonde, Elva House, Jacob Zidek, Lisa Smith, Ph.D., Jack Chen, Ph.D.
hCoV-19/USA/AK-PHL6708/2021	EPI_ISL_2716865	1/5/2021	Alaska State Virology Laboratory	Alaska State Virology Laboratory	Stephanie DeRonde, Elva House, Jacob Zidek, Lisa Smith, Ph.D., Jack Chen, Ph.D.
hCoV-19/USA/AK-PHL6710/2020	EPI_ISL_2716867	12/7/2020	Alaska State Virology Laboratory	Alaska State Virology Laboratory	Stephanie DeRonde, Elva House, Jacob Zidek, Lisa Smith, Ph.D., Jack Chen, Ph.D.
hCoV-19/USA/AK-PHL6712/2020	EPI_ISL_2716868	12/7/2020	Alaska State Virology Laboratory	Alaska State Virology Laboratory	Stephanie DeRonde, Elva House, Jacob Zidek, Lisa Smith, Ph.D., Jack Chen, Ph.D.
hCoV-19/USA/AK-PHL6713/2020	EPI_ISL_2716869	12/6/2020	Alaska State Virology Laboratory	Alaska State Virology Laboratory	Stephanie DeRonde, Elva House, Jacob Zidek, Lisa Smith, Ph.D., Jack Chen, Ph.D.
hCoV-19/USA/AK-CDC-ASC210110528/2021	EPI_ISL_2785037	6/21/2021	Aegis Sciences Corporation	Centers for Disease Control and Prevention Division of Viral Diseases, Pathogen Discovery	Dakota Howard, Dhwani Batra, Peter W. Cook, Kara Moser, Adrian Paskey, Jason Caravas, Benjamin Rambo-Martin, Shatavia Morrison, Christopher Gulvick, Scott Sammons, Yvette Unoarumhi, Darlene Wagner, Matthew Schmerer, Cyndi Clark, Patrick Campbell, Rob Case, Vikramsinha Ghorpade, Holly Houdeshell, Ola Kvalvaag, Dillon Nall, Ethan Sanders, Alec Vest, Shaun Westlund, Matthew Hardison, Clinton R. Paden, Duncan MacCannell
hCoV-19/USA/AK-CDC-FG-038624/2021	EPI_ISL_2785924	6/24/2021	Fulgent Genetics	Centers for Disease Control and Prevention Division of Viral Diseases, Pathogen Discovery	Dakota Howard, Dhwani Batra, Peter W. Cook, Kara Moser, Adrian Paskey, Jason Caravas, Benjamin Rambo-Martin, Shatavia Morrison, Christopher Gulvick, Scott Sammons, Yvette Unoarumhi, Darlene Wagner, Matthew Schmerer, Harry Gao, Mickey Li, John Gao, Joseph Fierro, Benafsh Sapra, Becky Tsai, Yan Meng, Doreen Ng, James Xie, Clinton R. Paden, Duncan MacCannell
hCoV-19/USA/AK-CDC-2-4523885/2021	EPI_ISL_2787511	5/18/2021	AK State Public Health Lab, State Health Department	Centers for Disease Control and Prevention Division of Viral Diseases, Pathogen Discovery	Mili Sheth, Sarah Nobles, Jasmine Padilla, Mark Burroughs, Shoshona Le, Katie Dillon, Peter Cook, Clinton R. Paden, Dhwani Batra, Krista Queen, Kristen Knipe, Dakota Howard, Yvette Unoarumhi, Darlene Wagner, Matthew Schmerer, Ben L. Rambo-Martin, Kristine Lacek, Sam Shepard, Alison Laufer Halpin, Dave Wentworth, Vivien Dugan, Suxiang Tong, Justin Lee
hCoV-19/USA/AK-PHL9662/2021	EPI_ISL_2790762	6/15/2021	Alaska State Virology Laboratory	Alaska State Virology Laboratory	Stephanie DeRonde, Elva House, Jacob Zidek, Lisa Smith, Ph.D., Jack Chen, Ph.D.
hCoV-19/USA/AK-PHL9663/2021	EPI_ISL_2790763	6/19/2021	Alaska State Virology Laboratory	Alaska State Virology Laboratory	Stephanie DeRonde, Elva House, Jacob Zidek, Lisa Smith, Ph.D., Jack Chen, Ph.D.
hCoV-19/USA/AK-PHL9664/2021	EPI_ISL_2790764	6/19/2021	Alaska State Virology Laboratory	Alaska State Virology Laboratory	Stephanie DeRonde, Elva House, Jacob Zidek, Lisa Smith, Ph.D., Jack Chen, Ph.D.
hCoV-19/USA/AK-PHL9665/2021	EPI_ISL_2790765	6/19/2021	Alaska State Virology Laboratory	Alaska State Virology Laboratory	Stephanie DeRonde, Elva House, Jacob Zidek, Lisa Smith, Ph.D., Jack Chen, Ph.D.
hCoV-19/USA/AK-PHL9666/2021	EPI_ISL_2790766	6/18/2021	Alaska State Virology Laboratory	Alaska State Virology Laboratory	Stephanie DeRonde, Elva House, Jacob Zidek, Lisa Smith, Ph.D., Jack Chen, Ph.D.
hCoV-19/USA/AK-PHL9667/2021	EPI_ISL_2790767	6/18/2021	Alaska State Virology Laboratory	Alaska State Virology Laboratory	Stephanie DeRonde, Elva House, Jacob Zidek, Lisa Smith, Ph.D., Jack Chen, Ph.D.
hCoV-19/USA/AK-PHL9668/2021	EPI_ISL_2790768	6/17/2021	Alaska State Virology Laboratory	Alaska State Virology Laboratory	Stephanie DeRonde, Elva House, Jacob Zidek, Lisa Smith, Ph.D., Jack Chen, Ph.D.
hCoV-19/USA/AK-PHL9669/2021	EPI_ISL_2790769	6/18/2021	Alaska State Virology Laboratory	Alaska State Virology Laboratory	Stephanie DeRonde, Elva House, Jacob Zidek, Lisa Smith, Ph.D., Jack Chen, Ph.D.
hCoV-19/USA/AK-PHL9670/2021	EPI_ISL_2790770	6/18/2021	Alaska State Virology Laboratory	Alaska State Virology Laboratory	Stephanie DeRonde, Elva House, Jacob Zidek, Lisa Smith, Ph.D., Jack Chen, Ph.D.
hCoV-19/USA/AK-PHL9671/2021	EPI_ISL_2790771	6/18/2021	Alaska State Virology Laboratory	Alaska State Virology Laboratory	Stephanie DeRonde, Elva House, Jacob Zidek, Lisa Smith, Ph.D., Jack Chen, Ph.D.
hCoV-19/USA/AK-PHL9673/2021	EPI_ISL_2790772	6/17/2021	Alaska State Virology Laboratory	Alaska State Virology Laboratory	Stephanie DeRonde, Elva House, Jacob Zidek, Lisa Smith, Ph.D., Jack Chen, Ph.D.
hCoV-19/USA/AK-PHL9675/2021	EPI_ISL_2790773	6/16/2021	Alaska State Virology Laboratory	Alaska State Virology Laboratory	Stephanie DeRonde, Elva House, Jacob Zidek, Lisa Smith, Ph.D., Jack Chen, Ph.D.

hCoV-19/USA/AK-PHL9676/2021	EPI_ISL_2790774	6/18/2021	Alaska State Virology Laboratory	Alaska State Virology Laboratory	Stephanie DeRonde, Elva House, Jacob Zidek, Lisa Smith, Ph.D., Jack Chen, Ph.D.
hCoV-19/USA/AK-PHL9677/2021	EPI_ISL_2790775	6/17/2021	Alaska State Virology Laboratory	Alaska State Virology Laboratory	Stephanie DeRonde, Elva House, Jacob Zidek, Lisa Smith, Ph.D., Jack Chen, Ph.D.
hCoV-19/USA/AK-PHL9678/2021	EPI_ISL_2790776	6/17/2021	Alaska State Virology Laboratory	Alaska State Virology Laboratory	Stephanie DeRonde, Elva House, Jacob Zidek, Lisa Smith, Ph.D., Jack Chen, Ph.D.
hCoV-19/USA/AK-PHL9679/2021	EPI_ISL_2790777	6/18/2021	Alaska State Virology Laboratory	Alaska State Virology Laboratory	Stephanie DeRonde, Elva House, Jacob Zidek, Lisa Smith, Ph.D., Jack Chen, Ph.D.
hCoV-19/USA/AK-PHL9680/2021	EPI_ISL_2790778	6/18/2021	Alaska State Virology Laboratory	Alaska State Virology Laboratory	Stephanie DeRonde, Elva House, Jacob Zidek, Lisa Smith, Ph.D., Jack Chen, Ph.D.
hCoV-19/USA/AK-PHL9681/2021	EPI_ISL_2790779	6/18/2021	Alaska State Virology Laboratory	Alaska State Virology Laboratory	Stephanie DeRonde, Elva House, Jacob Zidek, Lisa Smith, Ph.D., Jack Chen, Ph.D.
hCoV-19/USA/AK-PHL9684/2021	EPI_ISL_2790780	6/18/2021	Alaska State Virology Laboratory	Alaska State Virology Laboratory	Stephanie DeRonde, Elva House, Jacob Zidek, Lisa Smith, Ph.D., Jack Chen, Ph.D.
hCoV-19/USA/AK-PHL9685/2021	EPI_ISL_2790781	6/18/2021	Alaska State Virology Laboratory	Alaska State Virology Laboratory	Stephanie DeRonde, Elva House, Jacob Zidek, Lisa Smith, Ph.D., Jack Chen, Ph.D.
hCoV-19/USA/AK-PHL9686/2021	EPI_ISL_2790782	6/18/2021	Alaska State Virology Laboratory	Alaska State Virology Laboratory	Stephanie DeRonde, Elva House, Jacob Zidek, Lisa Smith, Ph.D., Jack Chen, Ph.D.
hCoV-19/USA/AK-PHL9688/2021	EPI_ISL_2790783	6/18/2021	Alaska State Virology Laboratory	Alaska State Virology Laboratory	Stephanie DeRonde, Elva House, Jacob Zidek, Lisa Smith, Ph.D., Jack Chen, Ph.D.
hCoV-19/USA/AK-PHL9689/2021	EPI_ISL_2790784	6/20/2021	Alaska State Virology Laboratory	Alaska State Virology Laboratory	Stephanie DeRonde, Elva House, Jacob Zidek, Lisa Smith, Ph.D., Jack Chen, Ph.D.
hCoV-19/USA/AK-PHL9690/2021	EPI_ISL_2790785	6/20/2021	Alaska State Virology Laboratory	Alaska State Virology Laboratory	Stephanie DeRonde, Elva House, Jacob Zidek, Lisa Smith, Ph.D., Jack Chen, Ph.D.
hCoV-19/USA/AK-PHL9691/2021	EPI_ISL_2790786	6/20/2021	Alaska State Virology Laboratory	Alaska State Virology Laboratory	Stephanie DeRonde, Elva House, Jacob Zidek, Lisa Smith, Ph.D., Jack Chen, Ph.D.
hCoV-19/USA/AK-PHL9692/2021	EPI_ISL_2790787	6/20/2021	Alaska State Virology Laboratory	Alaska State Virology Laboratory	Stephanie DeRonde, Elva House, Jacob Zidek, Lisa Smith, Ph.D., Jack Chen, Ph.D.
hCoV-19/USA/AK-PHL9693/2021	EPI_ISL_2790788	6/20/2021	Alaska State Virology Laboratory	Alaska State Virology Laboratory	Stephanie DeRonde, Elva House, Jacob Zidek, Lisa Smith, Ph.D., Jack Chen, Ph.D.
hCoV-19/USA/AK-PHL9695/2021	EPI_ISL_2790789	6/20/2021	Alaska State Virology Laboratory	Alaska State Virology Laboratory	Stephanie DeRonde, Elva House, Jacob Zidek, Lisa Smith, Ph.D., Jack Chen, Ph.D.
hCoV-19/USA/AK-PHL9696/2021	EPI_ISL_2790790	6/16/2021	Alaska State Virology Laboratory	Alaska State Virology Laboratory	Stephanie DeRonde, Elva House, Jacob Zidek, Lisa Smith, Ph.D., Jack Chen, Ph.D.
hCoV-19/USA/AK-PHL9697/2021	EPI_ISL_2790791	6/16/2021	Alaska State Virology Laboratory	Alaska State Virology Laboratory	Stephanie DeRonde, Elva House, Jacob Zidek, Lisa Smith, Ph.D., Jack Chen, Ph.D.
hCoV-19/USA/AK-PHL9698/2021	EPI_ISL_2790792	6/15/2021	Alaska State Virology Laboratory	Alaska State Virology Laboratory	Stephanie DeRonde, Elva House, Jacob Zidek, Lisa Smith, Ph.D., Jack Chen, Ph.D.
hCoV-19/USA/AK-PHL9699/2021	EPI_ISL_2790793	6/15/2021	Alaska State Virology Laboratory	Alaska State Virology Laboratory	Stephanie DeRonde, Elva House, Jacob Zidek, Lisa Smith, Ph.D., Jack Chen, Ph.D.
hCoV-19/USA/AK-PHL9700/2021	EPI_ISL_2790794	6/15/2021	Alaska State Virology Laboratory	Alaska State Virology Laboratory	Stephanie DeRonde, Elva House, Jacob Zidek, Lisa Smith, Ph.D., Jack Chen, Ph.D.
hCoV-19/USA/AK-PHL9701/2021	EPI_ISL_2790795	6/17/2021	Alaska State Virology Laboratory	Alaska State Virology Laboratory	Stephanie DeRonde, Elva House, Jacob Zidek, Lisa Smith, Ph.D., Jack Chen, Ph.D.
hCoV-19/USA/AK-PHL9703/2021	EPI_ISL_2790796	6/13/2021	Alaska State Virology Laboratory	Alaska State Virology Laboratory	Stephanie DeRonde, Elva House, Jacob Zidek, Lisa Smith, Ph.D., Jack Chen, Ph.D.
hCoV-19/USA/AK-PHL9704/2021	EPI_ISL_2790797	6/10/2021	Alaska State Virology Laboratory	Alaska State Virology Laboratory	Stephanie DeRonde, Elva House, Jacob Zidek, Lisa Smith, Ph.D., Jack Chen, Ph.D.
hCoV-19/USA/AK-PHL9705/2021	EPI_ISL_2790798	6/17/2021	Alaska State Virology Laboratory	Alaska State Virology Laboratory	Stephanie DeRonde, Elva House, Jacob Zidek, Lisa Smith, Ph.D., Jack Chen, Ph.D.
hCoV-19/USA/AK-PHL9706/2021	EPI_ISL_2790799	6/11/2021	Alaska State Virology Laboratory	Alaska State Virology Laboratory	Stephanie DeRonde, Elva House, Jacob Zidek, Lisa Smith, Ph.D., Jack Chen, Ph.D.
hCoV-19/USA/AK-PHL9388/2021	EPI_ISL_2791455	5/25/2021	Alaska State Virology Laboratory	Alaska State Virology Laboratory	Stephanie DeRonde, Elva House, Jacob Zidek, Lisa Smith, Ph.D., Jack Chen, Ph.D.
hCoV-19/USA/AK-PHL9390/2021	EPI_ISL_2791456	5/18/2021	Alaska State Virology Laboratory	Alaska State Virology Laboratory	Stephanie DeRonde, Elva House, Jacob Zidek, Lisa Smith, Ph.D., Jack Chen, Ph.D.
hCoV-19/USA/AK-PHL9410/2021	EPI_ISL_2791457	5/12/2021	Alaska State Virology Laboratory	Alaska State Virology Laboratory	Stephanie DeRonde, Elva House, Jacob Zidek, Lisa Smith, Ph.D., Jack Chen, Ph.D.
hCoV-19/USA/AK-PHL9411/2021	EPI_ISL_2791458	5/5/2021	Alaska State Virology Laboratory	Alaska State Virology Laboratory	Stephanie DeRonde, Elva House, Jacob Zidek, Lisa Smith, Ph.D., Jack Chen, Ph.D.
hCoV-19/USA/AK-PHL9413/2021	EPI_ISL_2791459	5/16/2021	Alaska State Virology Laboratory	Alaska State Virology Laboratory	Stephanie DeRonde, Elva House, Jacob Zidek, Lisa Smith, Ph.D., Jack Chen, Ph.D.
hCoV-19/USA/AK-PHL9414/2021	EPI_ISL_2791460	5/18/2021	Alaska State Virology Laboratory	Alaska State Virology Laboratory	Stephanie DeRonde, Elva House, Jacob Zidek, Lisa Smith, Ph.D., Jack Chen, Ph.D.
hCoV-19/USA/AK-PHL9415/2021	EPI_ISL_2791461	5/20/2021	Alaska State Virology Laboratory	Alaska State Virology Laboratory	Stephanie DeRonde, Elva House, Jacob Zidek, Lisa Smith, Ph.D., Jack Chen, Ph.D.
hCoV-19/USA/AK-PHL9417/2021	EPI_ISL_2791462	3/29/2021	Alaska State Virology Laboratory	Alaska State Virology Laboratory	Stephanie DeRonde, Elva House, Jacob Zidek, Lisa Smith, Ph.D., Jack Chen, Ph.D.
hCoV-19/USA/AK-PHL9707/2021	EPI_ISL_2791463	6/14/2021	Alaska State Virology Laboratory	Alaska State Virology Laboratory	Stephanie DeRonde, Elva House, Jacob Zidek, Lisa Smith, Ph.D., Jack Chen, Ph.D.
hCoV-19/USA/AK-PHL9709/2021	EPI_ISL_2791464	6/15/2021	Alaska State Virology Laboratory	Alaska State Virology Laboratory	Stephanie DeRonde, Elva House, Jacob Zidek, Lisa Smith, Ph.D., Jack Chen, Ph.D.
hCoV-19/USA/AK-PHL9710/2021	EPI_ISL_2791465	6/15/2021	Alaska State Virology Laboratory	Alaska State Virology Laboratory	Stephanie DeRonde, Elva House, Jacob Zidek, Lisa Smith, Ph.D., Jack Chen, Ph.D.
hCoV-19/USA/AK-PHL9711/2021	EPI_ISL_2791466	6/15/2021	Alaska State Virology Laboratory	Alaska State Virology Laboratory	Stephanie DeRonde, Elva House, Jacob Zidek, Lisa Smith, Ph.D., Jack Chen, Ph.D.
hCoV-19/USA/AK-PHL9712/2021	EPI_ISL_2791467	6/15/2021	Alaska State Virology Laboratory	Alaska State Virology Laboratory	Stephanie DeRonde, Elva House, Jacob Zidek, Lisa Smith, Ph.D., Jack Chen, Ph.D.
hCoV-19/USA/AK-PHL9713/2021	EPI_ISL_2791468	6/16/2021	Alaska State Virology Laboratory	Alaska State Virology Laboratory	Stephanie DeRonde, Elva House, Jacob Zidek, Lisa Smith, Ph.D., Jack Chen, Ph.D.
hCoV-19/USA/AK-PHL9715/2021	EPI_ISL_2791469	6/15/2021	Alaska State Virology Laboratory	Alaska State Virology Laboratory	Stephanie DeRonde, Elva House, Jacob Zidek, Lisa Smith, Ph.D., Jack Chen, Ph.D.
hCoV-19/USA/AK-PHL9716/2021	EPI_ISL_2791470	6/15/2021	Alaska State Virology Laboratory	Alaska State Virology Laboratory	Stephanie DeRonde, Elva House, Jacob Zidek, Lisa Smith, Ph.D., Jack Chen, Ph.D.
hCoV-19/USA/AK-PHL9717/2021	EPI_ISL_2791471	6/15/2021	Alaska State Virology Laboratory	Alaska State Virology Laboratory	Stephanie DeRonde, Elva House, Jacob Zidek, Lisa Smith, Ph.D., Jack Chen, Ph.D.
hCoV-19/USA/AK-PHL9718/2021	EPI_ISL_2791472	6/16/2021	Alaska State Virology Laboratory	Alaska State Virology Laboratory	Stephanie DeRonde, Elva House, Jacob Zidek, Lisa Smith, Ph.D., Jack Chen, Ph.D.
hCoV-19/USA/AK-PHL9720/2021	EPI_ISL_2791473	6/16/2021	Alaska State Virology Laboratory	Alaska State Virology Laboratory	Stephanie DeRonde, Elva House, Jacob Zidek, Lisa Smith, Ph.D., Jack Chen, Ph.D.
hCoV-19/USA/AK-PHL9721/2021	EPI_ISL_2791474	6/16/2021	Alaska State Virology Laboratory	Alaska State Virology Laboratory	Stephanie DeRonde, Elva House, Jacob Zidek, Lisa Smith, Ph.D., Jack Chen, Ph.D.
hCoV-19/USA/AK-PHL9722/2021	EPI_ISL_2791475	6/15/2021	Alaska State Virology Laboratory	Alaska State Virology Laboratory	Stephanie DeRonde, Elva House, Jacob Zidek, Lisa Smith, Ph.D., Jack Chen, Ph.D.
hCoV-19/USA/AK-PHL9723/2021	EPI_ISL_2791476	6/16/2021	Alaska State Virology Laboratory	Alaska State Virology Laboratory	Stephanie DeRonde, Elva House, Jacob Zidek, Lisa Smith, Ph.D., Jack Chen, Ph.D.
hCoV-19/USA/AK-PHL9724/2021	EPI_ISL_2791477	6/15/2021	Alaska State Virology Laboratory	Alaska State Virology Laboratory	Stephanie DeRonde, Elva House, Jacob Zidek, Lisa Smith, Ph.D., Jack Chen, Ph.D.
hCoV-19/USA/AK-PHL9725/2021	EPI_ISL_2791478	6/16/2021	Alaska State Virology Laboratory	Alaska State Virology Laboratory	Stephanie DeRonde, Elva House, Jacob Zidek, Lisa Smith, Ph.D., Jack Chen, Ph.D.
hCoV-19/USA/AK-PHL9726/2021	EPI_ISL_2791479	6/17/2021	Alaska State Virology Laboratory	Alaska State Virology Laboratory	Stephanie DeRonde, Elva House, Jacob Zidek, Lisa Smith, Ph.D., Jack Chen, Ph.D.

hCoV-19/USA/AK-PHL9728/2021	EPI_ISL_2791480	6/8/2021	Alaska State Virology Laboratory	Alaska State Virology Laboratory	Stephanie DeRonde, Elva House, Jacob Zidek, Lisa Smith, Ph.D., Jack Chen, Ph.D.
hCoV-19/USA/AK-PHL9729/2021	EPI_ISL_2791481	6/14/2021	Alaska State Virology Laboratory	Alaska State Virology Laboratory	Stephanie DeRonde, Elva House, Jacob Zidek, Lisa Smith, Ph.D., Jack Chen, Ph.D.
hCoV-19/USA/AK-PHL9730/2021	EPI_ISL_2791482	6/15/2021	Alaska State Virology Laboratory	Alaska State Virology Laboratory	Stephanie DeRonde, Elva House, Jacob Zidek, Lisa Smith, Ph.D., Jack Chen, Ph.D.
hCoV-19/USA/AK-PHL9731/2021	EPI_ISL_2791483	6/15/2021	Alaska State Virology Laboratory	Alaska State Virology Laboratory	Stephanie DeRonde, Elva House, Jacob Zidek, Lisa Smith, Ph.D., Jack Chen, Ph.D.
hCoV-19/USA/AK-PHL9732/2021	EPI_ISL_2791484	6/14/2021	Alaska State Virology Laboratory	Alaska State Virology Laboratory	Stephanie DeRonde, Elva House, Jacob Zidek, Lisa Smith, Ph.D., Jack Chen, Ph.D.
hCoV-19/USA/AK-PHL9734/2021	EPI_ISL_2791485	6/7/2021	Alaska State Virology Laboratory	Alaska State Virology Laboratory	Stephanie DeRonde, Elva House, Jacob Zidek, Lisa Smith, Ph.D., Jack Chen, Ph.D.
hCoV-19/USA/AK-PHL9735/2021	EPI_ISL_2791486	6/14/2021	Alaska State Virology Laboratory	Alaska State Virology Laboratory	Stephanie DeRonde, Elva House, Jacob Zidek, Lisa Smith, Ph.D., Jack Chen, Ph.D.
hCoV-19/USA/AK-PHL9736/2021	EPI_ISL_2791487	6/7/2021	Alaska State Virology Laboratory	Alaska State Virology Laboratory	Stephanie DeRonde, Elva House, Jacob Zidek, Lisa Smith, Ph.D., Jack Chen, Ph.D.
hCoV-19/USA/AK-PHL9804/2021	EPI_ISL_2893319	6/13/2021	Alaska State Virology Laboratory	Alaska State Virology Laboratory	Stephanie DeRonde, Elva House, Jacob Zidek, Lisa Smith, Ph.D., Jack Chen, Ph.D.
hCoV-19/USA/AK-PHL9805/2021	EPI_ISL_2893320	6/13/2021	Alaska State Virology Laboratory	Alaska State Virology Laboratory	Stephanie DeRonde, Elva House, Jacob Zidek, Lisa Smith, Ph.D., Jack Chen, Ph.D.
hCoV-19/USA/AK-PHL9809/2021	EPI_ISL_2893321	6/13/2021	Alaska State Virology Laboratory	Alaska State Virology Laboratory	Stephanie DeRonde, Elva House, Jacob Zidek, Lisa Smith, Ph.D., Jack Chen, Ph.D.
hCoV-19/USA/AK-PHL9789/2021	EPI_ISL_2893322	6/21/2021	Alaska State Virology Laboratory	Alaska State Virology Laboratory	Stephanie DeRonde, Elva House, Jacob Zidek, Lisa Smith, Ph.D., Jack Chen, Ph.D.
hCoV-19/USA/AK-PHL9810/2021	EPI_ISL_2893323	6/13/2021	Alaska State Virology Laboratory	Alaska State Virology Laboratory	Stephanie DeRonde, Elva House, Jacob Zidek, Lisa Smith, Ph.D., Jack Chen, Ph.D.
hCoV-19/USA/AK-PHL9811/2021	EPI_ISL_2893324	6/14/2021	Alaska State Virology Laboratory	Alaska State Virology Laboratory	Stephanie DeRonde, Elva House, Jacob Zidek, Lisa Smith, Ph.D., Jack Chen, Ph.D.
hCoV-19/USA/AK-PHL9790/2021	EPI_ISL_2893325	6/18/2021	Alaska State Virology Laboratory	Alaska State Virology Laboratory	Stephanie DeRonde, Elva House, Jacob Zidek, Lisa Smith, Ph.D., Jack Chen, Ph.D.
hCoV-19/USA/AK-PHL9791/2021	EPI_ISL_2893326	6/18/2021	Alaska State Virology Laboratory	Alaska State Virology Laboratory	Stephanie DeRonde, Elva House, Jacob Zidek, Lisa Smith, Ph.D., Jack Chen, Ph.D.
hCoV-19/USA/AK-PHL9792/2021	EPI_ISL_2893327	6/18/2021	Alaska State Virology Laboratory	Alaska State Virology Laboratory	Stephanie DeRonde, Elva House, Jacob Zidek, Lisa Smith, Ph.D., Jack Chen, Ph.D.
hCoV-19/USA/AK-PHL9793/2021	EPI_ISL_2893328	6/17/2021	Alaska State Virology Laboratory	Alaska State Virology Laboratory	Stephanie DeRonde, Elva House, Jacob Zidek, Lisa Smith, Ph.D., Jack Chen, Ph.D.
hCoV-19/USA/AK-PHL9826/2021	EPI_ISL_2893329	4/25/2021	Alaska State Virology Laboratory	Alaska State Virology Laboratory	Stephanie DeRonde, Elva House, Jacob Zidek, Lisa Smith, Ph.D., Jack Chen, Ph.D.
hCoV-19/USA/AK-PHL9799/2021	EPI_ISL_2893330	6/9/2021	Alaska State Virology Laboratory	Alaska State Virology Laboratory	Stephanie DeRonde, Elva House, Jacob Zidek, Lisa Smith, Ph.D., Jack Chen, Ph.D.
hCoV-19/USA/AK-PHL9786/2021	EPI_ISL_2893331	6/21/2021	Alaska State Virology Laboratory	Alaska State Virology Laboratory	Stephanie DeRonde, Elva House, Jacob Zidek, Lisa Smith, Ph.D., Jack Chen, Ph.D.
hCoV-19/USA/AK-PHL9800/2021	EPI_ISL_2893332	6/9/2021	Alaska State Virology Laboratory	Alaska State Virology Laboratory	Stephanie DeRonde, Elva House, Jacob Zidek, Lisa Smith, Ph.D., Jack Chen, Ph.D.
hCoV-19/USA/AK-PHL9813/2021	EPI_ISL_2893333	6/14/2021	Alaska State Virology Laboratory	Alaska State Virology Laboratory	Stephanie DeRonde, Elva House, Jacob Zidek, Lisa Smith, Ph.D., Jack Chen, Ph.D.
hCoV-19/USA/AK-PHL9815/2021	EPI_ISL_2893334	6/14/2021	Alaska State Virology Laboratory	Alaska State Virology Laboratory	Stephanie DeRonde, Elva House, Jacob Zidek, Lisa Smith, Ph.D., Jack Chen, Ph.D.
hCoV-19/USA/AK-PHL9785/2021	EPI_ISL_2893335	6/21/2021	Alaska State Virology Laboratory	Alaska State Virology Laboratory	Stephanie DeRonde, Elva House, Jacob Zidek, Lisa Smith, Ph.D., Jack Chen, Ph.D.
hCoV-19/USA/AK-PHL9787/2021	EPI_ISL_2893336	6/21/2021	Alaska State Virology Laboratory	Alaska State Virology Laboratory	Stephanie DeRonde, Elva House, Jacob Zidek, Lisa Smith, Ph.D., Jack Chen, Ph.D.
hCoV-19/USA/AK-PHL9788/2021	EPI_ISL_2893337	6/21/2021	Alaska State Virology Laboratory	Alaska State Virology Laboratory	Stephanie DeRonde, Elva House, Jacob Zidek, Lisa Smith, Ph.D., Jack Chen, Ph.D.
hCoV-19/USA/AK-PHL9784/2021	EPI_ISL_2893338	6/21/2021	Alaska State Virology Laboratory	Alaska State Virology Laboratory	Stephanie DeRonde, Elva House, Jacob Zidek, Lisa Smith, Ph.D., Jack Chen, Ph.D.
hCoV-19/USA/AK-PHL9814/2021	EPI_ISL_2893339	6/14/2021	Alaska State Virology Laboratory	Alaska State Virology Laboratory	Stephanie DeRonde, Elva House, Jacob Zidek, Lisa Smith, Ph.D., Jack Chen, Ph.D.
hCoV-19/USA/AK-PHL9806/2021	EPI_ISL_2893340	6/13/2021	Alaska State Virology Laboratory	Alaska State Virology Laboratory	Stephanie DeRonde, Elva House, Jacob Zidek, Lisa Smith, Ph.D., Jack Chen, Ph.D.
hCoV-19/USA/AK-PHL9808/2021	EPI_ISL_2893341	6/13/2021	Alaska State Virology Laboratory	Alaska State Virology Laboratory	Stephanie DeRonde, Elva House, Jacob Zidek, Lisa Smith, Ph.D., Jack Chen, Ph.D.
hCoV-19/USA/AK-PHL9812/2021	EPI_ISL_2893342	6/14/2021	Alaska State Virology Laboratory	Alaska State Virology Laboratory	Stephanie DeRonde, Elva House, Jacob Zidek, Lisa Smith, Ph.D., Jack Chen, Ph.D.
hCoV-19/USA/AK-PHL9794/2021	EPI_ISL_2893343	6/7/2021	Alaska State Virology Laboratory	Alaska State Virology Laboratory	Stephanie DeRonde, Elva House, Jacob Zidek, Lisa Smith, Ph.D., Jack Chen, Ph.D.
hCoV-19/USA/AK-PHL9797/2021	EPI_ISL_2893344	6/7/2021	Alaska State Virology Laboratory	Alaska State Virology Laboratory	Stephanie DeRonde, Elva House, Jacob Zidek, Lisa Smith, Ph.D., Jack Chen, Ph.D.
hCoV-19/USA/AK-PHL9795/2021	EPI_ISL_2893345	6/8/2021	Alaska State Virology Laboratory	Alaska State Virology Laboratory	Stephanie DeRonde, Elva House, Jacob Zidek, Lisa Smith, Ph.D., Jack Chen, Ph.D.
hCoV-19/USA/AK-PHL9796/2021	EPI_ISL_2893346	6/8/2021	Alaska State Virology Laboratory	Alaska State Virology Laboratory	Stephanie DeRonde, Elva House, Jacob Zidek, Lisa Smith, Ph.D., Jack Chen, Ph.D.
hCoV-19/USA/AK-PHL9803/2021	EPI_ISL_2893347	6/12/2021	Alaska State Virology Laboratory	Alaska State Virology Laboratory	Stephanie DeRonde, Elva House, Jacob Zidek, Lisa Smith, Ph.D., Jack Chen, Ph.D.
hCoV-19/USA/AK-PHL9817/2021	EPI_ISL_2893348	4/26/2021	Alaska State Virology Laboratory	Alaska State Virology Laboratory	Stephanie DeRonde, Elva House, Jacob Zidek, Lisa Smith, Ph.D., Jack Chen, Ph.D.
hCoV-19/USA/AK-PHL9818/2021	EPI_ISL_2893349	4/16/2021	Alaska State Virology Laboratory	Alaska State Virology Laboratory	Stephanie DeRonde, Elva House, Jacob Zidek, Lisa Smith, Ph.D., Jack Chen, Ph.D.
hCoV-19/USA/AK-PHL9819/2021	EPI_ISL_2893350	4/7/2021	Alaska State Virology Laboratory	Alaska State Virology Laboratory	Stephanie DeRonde, Elva House, Jacob Zidek, Lisa Smith, Ph.D., Jack Chen, Ph.D.
hCoV-19/USA/AK-PHL9820/2021	EPI_ISL_2893351	4/24/2021	Alaska State Virology Laboratory	Alaska State Virology Laboratory	Stephanie DeRonde, Elva House, Jacob Zidek, Lisa Smith, Ph.D., Jack Chen, Ph.D.
hCoV-19/USA/AK-PHL9823/2021	EPI_ISL_2893352	4/17/2021	Alaska State Virology Laboratory	Alaska State Virology Laboratory	Stephanie DeRonde, Elva House, Jacob Zidek, Lisa Smith, Ph.D., Jack Chen, Ph.D.
hCoV-19/USA/AK-PHL9825/2021	EPI_ISL_2893353	4/26/2021	Alaska State Virology Laboratory	Alaska State Virology Laboratory	Stephanie DeRonde, Elva House, Jacob Zidek, Lisa Smith, Ph.D., Jack Chen, Ph.D.
hCoV-19/USA/AK-PHL9828/2021	EPI_ISL_2893354	4/26/2021	Alaska State Virology Laboratory	Alaska State Virology Laboratory	Stephanie DeRonde, Elva House, Jacob Zidek, Lisa Smith, Ph.D., Jack Chen, Ph.D.
hCoV-19/USA/AK-PHL9749/2021	EPI_ISL_2893355	6/22/2021	Alaska State Virology Laboratory	Alaska State Virology Laboratory	Stephanie DeRonde, Elva House, Jacob Zidek, Lisa Smith, Ph.D., Jack Chen, Ph.D.
hCoV-19/USA/AK-PHL9756/2021	EPI_ISL_2893356	6/22/2021	Alaska State Virology Laboratory	Alaska State Virology Laboratory	Stephanie DeRonde, Elva House, Jacob Zidek, Lisa Smith, Ph.D., Jack Chen, Ph.D.
hCoV-19/USA/AK-PHL9757/2021	EPI_ISL_2893357	6/23/2021	Alaska State Virology Laboratory	Alaska State Virology Laboratory	Stephanie DeRonde, Elva House, Jacob Zidek, Lisa Smith, Ph.D., Jack Chen, Ph.D.
hCoV-19/USA/AK-PHL9766/2021	EPI_ISL_2893358	6/24/2021	Alaska State Virology Laboratory	Alaska State Virology Laboratory	Stephanie DeRonde, Elva House, Jacob Zidek, Lisa Smith, Ph.D., Jack Chen, Ph.D.
hCoV-19/USA/AK-PHL9772/2021	EPI_ISL_2893359	6/24/2021	Alaska State Virology Laboratory	Alaska State Virology Laboratory	Stephanie DeRonde, Elva House, Jacob Zidek, Lisa Smith, Ph.D., Jack Chen, Ph.D.
hCoV-19/USA/AK-PHL9774/2021	EPI_ISL_2893360	6/24/2021	Alaska State Virology Laboratory	Alaska State Virology Laboratory	Stephanie DeRonde, Elva House, Jacob Zidek, Lisa Smith, Ph.D., Jack Chen, Ph.D.
hCoV-19/USA/AK-PHL9777/2021	EPI_ISL_2893361	6/24/2021	Alaska State Virology Laboratory	Alaska State Virology Laboratory	Stephanie DeRonde, Elva House, Jacob Zidek, Lisa Smith, Ph.D., Jack Chen, Ph.D.

hCoV-19/USA/AK-PHL9780/2021	EPI_ISL_2893362	6/25/2021	Alaska State Virology Laboratory	Alaska State Virology Laboratory	Stephanie DeRonde, Elva House, Jacob Zidek, Lisa Smith, Ph.D., Jack Chen, Ph.D.
hCoV-19/USA/AK-PHL9743/2021	EPI_ISL_2893363	6/21/2021	Alaska State Virology Laboratory	Alaska State Virology Laboratory	Stephanie DeRonde, Elva House, Jacob Zidek, Lisa Smith, Ph.D., Jack Chen, Ph.D.
hCoV-19/USA/AK-PHL9748/2021	EPI_ISL_2893364	6/22/2021	Alaska State Virology Laboratory	Alaska State Virology Laboratory	Stephanie DeRonde, Elva House, Jacob Zidek, Lisa Smith, Ph.D., Jack Chen, Ph.D.
hCoV-19/USA/AK-PHL9751/2021	EPI_ISL_2893365	6/22/2021	Alaska State Virology Laboratory	Alaska State Virology Laboratory	Stephanie DeRonde, Elva House, Jacob Zidek, Lisa Smith, Ph.D., Jack Chen, Ph.D.
hCoV-19/USA/AK-PHL9752/2021	EPI_ISL_2893366	6/22/2021	Alaska State Virology Laboratory	Alaska State Virology Laboratory	Stephanie DeRonde, Elva House, Jacob Zidek, Lisa Smith, Ph.D., Jack Chen, Ph.D.
hCoV-19/USA/AK-PHL9755/2021	EPI_ISL_2893367	6/23/2021	Alaska State Virology Laboratory	Alaska State Virology Laboratory	Stephanie DeRonde, Elva House, Jacob Zidek, Lisa Smith, Ph.D., Jack Chen, Ph.D.
hCoV-19/USA/AK-PHL9761/2021	EPI_ISL_2893368	6/23/2021	Alaska State Virology Laboratory	Alaska State Virology Laboratory	Stephanie DeRonde, Elva House, Jacob Zidek, Lisa Smith, Ph.D., Jack Chen, Ph.D.
hCoV-19/USA/AK-PHL9763/2021	EPI_ISL_2893369	6/15/2021	Alaska State Virology Laboratory	Alaska State Virology Laboratory	Stephanie DeRonde, Elva House, Jacob Zidek, Lisa Smith, Ph.D., Jack Chen, Ph.D.
hCoV-19/USA/AK-PHL9764/2021	EPI_ISL_2893370	6/5/2021	Alaska State Virology Laboratory	Alaska State Virology Laboratory	Stephanie DeRonde, Elva House, Jacob Zidek, Lisa Smith, Ph.D., Jack Chen, Ph.D.
hCoV-19/USA/AK-PHL9778/2021	EPI_ISL_2893371	6/25/2021	Alaska State Virology Laboratory	Alaska State Virology Laboratory	Stephanie DeRonde, Elva House, Jacob Zidek, Lisa Smith, Ph.D., Jack Chen, Ph.D.
hCoV-19/USA/AK-PHL9738/2021	EPI_ISL_2893373	6/21/2021	Alaska State Virology Laboratory	Alaska State Virology Laboratory	Stephanie DeRonde, Elva House, Jacob Zidek, Lisa Smith, Ph.D., Jack Chen, Ph.D.
hCoV-19/USA/AK-PHL9770/2021	EPI_ISL_2893374	6/24/2021	Alaska State Virology Laboratory	Alaska State Virology Laboratory	Stephanie DeRonde, Elva House, Jacob Zidek, Lisa Smith, Ph.D., Jack Chen, Ph.D.
hCoV-19/USA/AK-PHL9739/2021	EPI_ISL_2893375	6/21/2021	Alaska State Virology Laboratory	Alaska State Virology Laboratory	Stephanie DeRonde, Elva House, Jacob Zidek, Lisa Smith, Ph.D., Jack Chen, Ph.D.
hCoV-19/USA/AK-PHL9753/2021	EPI_ISL_2893376	6/22/2021	Alaska State Virology Laboratory	Alaska State Virology Laboratory	Stephanie DeRonde, Elva House, Jacob Zidek, Lisa Smith, Ph.D., Jack Chen, Ph.D.
hCoV-19/USA/AK-PHL9741/2021	EPI_ISL_2893377	6/21/2021	Alaska State Virology Laboratory	Alaska State Virology Laboratory	Stephanie DeRonde, Elva House, Jacob Zidek, Lisa Smith, Ph.D., Jack Chen, Ph.D.
hCoV-19/USA/AK-PHL9740/2021	EPI_ISL_2893378	6/21/2021	Alaska State Virology Laboratory	Alaska State Virology Laboratory	Stephanie DeRonde, Elva House, Jacob Zidek, Lisa Smith, Ph.D., Jack Chen, Ph.D.
hCoV-19/USA/AK-PHL9744/2021	EPI_ISL_2893379	6/21/2021	Alaska State Virology Laboratory	Alaska State Virology Laboratory	Stephanie DeRonde, Elva House, Jacob Zidek, Lisa Smith, Ph.D., Jack Chen, Ph.D.
hCoV-19/USA/AK-PHL9745/2021	EPI_ISL_2893380	6/21/2021	Alaska State Virology Laboratory	Alaska State Virology Laboratory	Stephanie DeRonde, Elva House, Jacob Zidek, Lisa Smith, Ph.D., Jack Chen, Ph.D.
hCoV-19/USA/AK-PHL9746/2021	EPI_ISL_2893381	6/20/2021	Alaska State Virology Laboratory	Alaska State Virology Laboratory	Stephanie DeRonde, Elva House, Jacob Zidek, Lisa Smith, Ph.D., Jack Chen, Ph.D.
hCoV-19/USA/AK-PHL9750/2021	EPI_ISL_2893382	6/22/2021	Alaska State Virology Laboratory	Alaska State Virology Laboratory	Stephanie DeRonde, Elva House, Jacob Zidek, Lisa Smith, Ph.D., Jack Chen, Ph.D.
hCoV-19/USA/AK-PHL9758/2021	EPI_ISL_2893383	6/23/2021	Alaska State Virology Laboratory	Alaska State Virology Laboratory	Stephanie DeRonde, Elva House, Jacob Zidek, Lisa Smith, Ph.D., Jack Chen, Ph.D.
hCoV-19/USA/AK-PHL9760/2021	EPI_ISL_2893384	6/23/2021	Alaska State Virology Laboratory	Alaska State Virology Laboratory	Stephanie DeRonde, Elva House, Jacob Zidek, Lisa Smith, Ph.D., Jack Chen, Ph.D.
hCoV-19/USA/AK-PHL9762/2021	EPI_ISL_2893385	6/23/2021	Alaska State Virology Laboratory	Alaska State Virology Laboratory	Stephanie DeRonde, Elva House, Jacob Zidek, Lisa Smith, Ph.D., Jack Chen, Ph.D.
hCoV-19/USA/AK-PHL9765/2021	EPI_ISL_2893386	6/22/2021	Alaska State Virology Laboratory	Alaska State Virology Laboratory	Stephanie DeRonde, Elva House, Jacob Zidek, Lisa Smith, Ph.D., Jack Chen, Ph.D.
hCoV-19/USA/AK-PHL9771/2021	EPI_ISL_2893387	6/24/2021	Alaska State Virology Laboratory	Alaska State Virology Laboratory	Stephanie DeRonde, Elva House, Jacob Zidek, Lisa Smith, Ph.D., Jack Chen, Ph.D.
hCoV-19/USA/AK-PHL9776/2021	EPI_ISL_2893388	6/24/2021	Alaska State Virology Laboratory	Alaska State Virology Laboratory	Stephanie DeRonde, Elva House, Jacob Zidek, Lisa Smith, Ph.D., Jack Chen, Ph.D.
hCoV-19/USA/AK-PHL9759/2021	EPI_ISL_2893389	6/22/2021	Alaska State Virology Laboratory	Alaska State Virology Laboratory	Stephanie DeRonde, Elva House, Jacob Zidek, Lisa Smith, Ph.D., Jack Chen, Ph.D.
hCoV-19/USA/AK-PHL9773/2021	EPI_ISL_2893390	6/24/2021	Alaska State Virology Laboratory	Alaska State Virology Laboratory	Stephanie DeRonde, Elva House, Jacob Zidek, Lisa Smith, Ph.D., Jack Chen, Ph.D.
hCoV-19/USA/AK-PHL9779/2021	EPI_ISL_2893391	6/25/2021	Alaska State Virology Laboratory	Alaska State Virology Laboratory	Stephanie DeRonde, Elva House, Jacob Zidek, Lisa Smith, Ph.D., Jack Chen, Ph.D.
hCoV-19/USA/AK-PHL9742/2021	EPI_ISL_2893392	6/21/2021	Alaska State Virology Laboratory	Alaska State Virology Laboratory	Stephanie DeRonde, Elva House, Jacob Zidek, Lisa Smith, Ph.D., Jack Chen, Ph.D.
hCoV-19/USA/AK-PHL9775/2021	EPI_ISL_2893393	6/24/2021	Alaska State Virology Laboratory	Alaska State Virology Laboratory	Stephanie DeRonde, Elva House, Jacob Zidek, Lisa Smith, Ph.D., Jack Chen, Ph.D.
hCoV-19/USA/AK-PHL9837/2021	EPI_ISL_2966425	6/26/2021	Alaska State Virology Laboratory	Alaska State Virology Laboratory	Stephanie DeRonde, Elva House, Jacob Zidek, Lisa Smith, Ph.D., Jack Chen, Ph.D.
hCoV-19/USA/AK-PHL9841/2021	EPI_ISL_2966428	6/26/2021	Alaska State Virology Laboratory	Alaska State Virology Laboratory	Stephanie DeRonde, Elva House, Jacob Zidek, Lisa Smith, Ph.D., Jack Chen, Ph.D.
hCoV-19/USA/AK-PHL9842/2021	EPI_ISL_2966429	6/26/2021	Alaska State Virology Laboratory	Alaska State Virology Laboratory	Stephanie DeRonde, Elva House, Jacob Zidek, Lisa Smith, Ph.D., Jack Chen, Ph.D.
hCoV-19/USA/AK-PHL9843/2021	EPI_ISL_2966430	6/26/2021	Alaska State Virology Laboratory	Alaska State Virology Laboratory	Stephanie DeRonde, Elva House, Jacob Zidek, Lisa Smith, Ph.D., Jack Chen, Ph.D.
hCoV-19/USA/AK-PHL9848/2021	EPI_ISL_2966433	6/26/2021	Alaska State Virology Laboratory	Alaska State Virology Laboratory	Stephanie DeRonde, Elva House, Jacob Zidek, Lisa Smith, Ph.D., Jack Chen, Ph.D.
hCoV-19/USA/AK-PHL9906/2021	EPI_ISL_2969602	6/20/2021	Alaska State Virology Laboratory	Alaska State Virology Laboratory	Stephanie DeRonde, Elva House, Jacob Zidek, Lisa Smith, Ph.D., Jack Chen, Ph.D.
hCoV-19/USA/AK-PHL9907/2021	EPI_ISL_2969603	6/18/2021	Alaska State Virology Laboratory	Alaska State Virology Laboratory	Stephanie DeRonde, Elva House, Jacob Zidek, Lisa Smith, Ph.D., Jack Chen, Ph.D.
hCoV-19/USA/AK-PHL9908/2021	EPI_ISL_2969604	6/24/2021	Alaska State Virology Laboratory	Alaska State Virology Laboratory	Stephanie DeRonde, Elva House, Jacob Zidek, Lisa Smith, Ph.D., Jack Chen, Ph.D.
hCoV-19/USA/AK-PHL9909/2021	EPI_ISL_2969605	6/26/2021	Alaska State Virology Laboratory	Alaska State Virology Laboratory	Stephanie DeRonde, Elva House, Jacob Zidek, Lisa Smith, Ph.D., Jack Chen, Ph.D.
hCoV-19/USA/AK-PHL9913/2021	EPI_ISL_2969609	6/25/2021	Alaska State Virology Laboratory	Alaska State Virology Laboratory	Stephanie DeRonde, Elva House, Jacob Zidek, Lisa Smith, Ph.D., Jack Chen, Ph.D.
hCoV-19/USA/AK-PHL9914/2021	EPI_ISL_2969610	6/15/2021	Alaska State Virology Laboratory	Alaska State Virology Laboratory	Stephanie DeRonde, Elva House, Jacob Zidek, Lisa Smith, Ph.D., Jack Chen, Ph.D.
hCoV-19/USA/AK-PHL9915/2021	EPI_ISL_2969611	6/12/2021	Alaska State Virology Laboratory	Alaska State Virology Laboratory	Stephanie DeRonde, Elva House, Jacob Zidek, Lisa Smith, Ph.D., Jack Chen, Ph.D.
hCoV-19/USA/AK-PHL9918/2021	EPI_ISL_2969613	6/25/2021	Alaska State Virology Laboratory	Alaska State Virology Laboratory	Stephanie DeRonde, Elva House, Jacob Zidek, Lisa Smith, Ph.D., Jack Chen, Ph.D.
hCoV-19/USA/AK-PHL9919/2021	EPI_ISL_2969614	6/25/2021	Alaska State Virology Laboratory	Alaska State Virology Laboratory	Stephanie DeRonde, Elva House, Jacob Zidek, Lisa Smith, Ph.D., Jack Chen, Ph.D.
hCoV-19/USA/AK-PHL9920/2021	EPI_ISL_2969615	6/21/2021	Alaska State Virology Laboratory	Alaska State Virology Laboratory	Stephanie DeRonde, Elva House, Jacob Zidek, Lisa Smith, Ph.D., Jack Chen, Ph.D.
hCoV-19/USA/AK-PHL9973/2021	EPI_ISL_2987144	6/21/2021	Alaska State Virology Laboratory	Alaska State Virology Laboratory	Stephanie DeRonde, Elva House, Jacob Zidek, Lisa Smith, Ph.D., Jack Chen, Ph.D.
hCoV-19/USA/AK-PHL9930/2021	EPI_ISL_2987153	6/25/2021	Alaska State Virology Laboratory	Alaska State Virology Laboratory	Stephanie DeRonde, Elva House, Jacob Zidek, Lisa Smith, Ph.D., Jack Chen, Ph.D.
hCoV-19/USA/AK-PHL9933/2021	EPI_ISL_2987154	6/22/2021	Alaska State Virology Laboratory	Alaska State Virology Laboratory	Stephanie DeRonde, Elva House, Jacob Zidek, Lisa Smith, Ph.D., Jack Chen, Ph.D.
hCoV-19/USA/AK-PHL9936/2021	EPI_ISL_2987155	6/21/2021	Alaska State Virology Laboratory	Alaska State Virology Laboratory	Stephanie DeRonde, Elva House, Jacob Zidek, Lisa Smith, Ph.D., Jack Chen, Ph.D.
hCoV-19/USA/AK-PHL9948/2021	EPI_ISL_2987156	6/21/2021	Alaska State Virology Laboratory	Alaska State Virology Laboratory	Stephanie DeRonde, Elva House, Jacob Zidek, Lisa Smith, Ph.D., Jack Chen, Ph.D.

hCoV-19/USA/AK-PHL9949/2021	EPI_ISL_2987157	6/21/2021 Alaska State Virology Laboratory	Alaska State Virology Laboratory	Stephanie DeRonde, Elva House, Jacob Zidek, Lisa Smith, Ph.D., Jack Chen, Ph.D.
hCoV-19/USA/AK-PHL9950/2021	EPI_ISL_2987158	6/21/2021 Alaska State Virology Laboratory	Alaska State Virology Laboratory	Stephanie DeRonde, Elva House, Jacob Zidek, Lisa Smith, Ph.D., Jack Chen, Ph.D.
hCoV-19/USA/AK-PHL9974/2021	EPI_ISL_2987160	6/21/2021 Alaska State Virology Laboratory	Alaska State Virology Laboratory	Stephanie DeRonde, Elva House, Jacob Zidek, Lisa Smith, Ph.D., Jack Chen, Ph.D.
hCoV-19/USA/AK-PHL9931/2021	EPI_ISL_2987161	6/25/2021 Alaska State Virology Laboratory	Alaska State Virology Laboratory	Stephanie DeRonde, Elva House, Jacob Zidek, Lisa Smith, Ph.D., Jack Chen, Ph.D.
hCoV-19/USA/AK-PHL9971/2021	EPI_ISL_2987163	6/25/2021 Alaska State Virology Laboratory	Alaska State Virology Laboratory	Stephanie DeRonde, Elva House, Jacob Zidek, Lisa Smith, Ph.D., Jack Chen, Ph.D.
hCoV-19/USA/AK-PHL9964/2021	EPI_ISL_2987166	4/27/2021 Alaska State Virology Laboratory	Alaska State Virology Laboratory	Stephanie DeRonde, Elva House, Jacob Zidek, Lisa Smith, Ph.D., Jack Chen, Ph.D.
hCoV-19/USA/AK-PHL9956/2021	EPI_ISL_2987175	4/26/2021 Alaska State Virology Laboratory	Alaska State Virology Laboratory	Stephanie DeRonde, Elva House, Jacob Zidek, Lisa Smith, Ph.D., Jack Chen, Ph.D.
hCoV-19/USA/AK-PHL9957/2021	EPI_ISL_2987176	4/26/2021 Alaska State Virology Laboratory	Alaska State Virology Laboratory	Stephanie DeRonde, Elva House, Jacob Zidek, Lisa Smith, Ph.D., Jack Chen, Ph.D.
hCoV-19/USA/AK-PHL9958/2021	EPI_ISL_2987177	4/27/2021 Alaska State Virology Laboratory	Alaska State Virology Laboratory	Stephanie DeRonde, Elva House, Jacob Zidek, Lisa Smith, Ph.D., Jack Chen, Ph.D.
hCoV-19/USA/AK-PHL9959/2021	EPI_ISL_2987178	4/26/2021 Alaska State Virology Laboratory	Alaska State Virology Laboratory	Stephanie DeRonde, Elva House, Jacob Zidek, Lisa Smith, Ph.D., Jack Chen, Ph.D.
hCoV-19/USA/AK-PHL9960/2021	EPI_ISL_2987179	4/27/2021 Alaska State Virology Laboratory	Alaska State Virology Laboratory	Stephanie DeRonde, Elva House, Jacob Zidek, Lisa Smith, Ph.D., Jack Chen, Ph.D.
hCoV-19/USA/AK-PHL9961/2021	EPI_ISL_2987180	4/27/2021 Alaska State Virology Laboratory	Alaska State Virology Laboratory	Stephanie DeRonde, Elva House, Jacob Zidek, Lisa Smith, Ph.D., Jack Chen, Ph.D.
hCoV-19/USA/AK-PHL9962/2021	EPI_ISL_2987181	4/27/2021 Alaska State Virology Laboratory	Alaska State Virology Laboratory	Stephanie DeRonde, Elva House, Jacob Zidek, Lisa Smith, Ph.D., Jack Chen, Ph.D.
hCoV-19/USA/AK-PHL9963/2021	EPI_ISL_2987182	4/27/2021 Alaska State Virology Laboratory	Alaska State Virology Laboratory	Stephanie DeRonde, Elva House, Jacob Zidek, Lisa Smith, Ph.D., Jack Chen, Ph.D.
hCoV-19/USA/AK-PHL10002/2021	EPI_ISL_2987183	4/24/2021 Alaska State Virology Laboratory	Alaska State Virology Laboratory	Stephanie DeRonde, Elva House, Jacob Zidek, Lisa Smith, Ph.D., Jack Chen, Ph.D.
hCoV-19/USA/AK-PHL10003/2021	EPI_ISL_2987184	4/24/2021 Alaska State Virology Laboratory	Alaska State Virology Laboratory	Stephanie DeRonde, Elva House, Jacob Zidek, Lisa Smith, Ph.D., Jack Chen, Ph.D.
hCoV-19/USA/AK-PHL10004/2021	EPI_ISL_2987185	4/24/2021 Alaska State Virology Laboratory	Alaska State Virology Laboratory	Stephanie DeRonde, Elva House, Jacob Zidek, Lisa Smith, Ph.D., Jack Chen, Ph.D.
hCoV-19/USA/AK-PHL10005/2021	EPI_ISL_2987186	4/24/2021 Alaska State Virology Laboratory	Alaska State Virology Laboratory	Stephanie DeRonde, Elva House, Jacob Zidek, Lisa Smith, Ph.D., Jack Chen, Ph.D.
hCoV-19/USA/AK-PHL10006/2021	EPI_ISL_2987187	4/24/2021 Alaska State Virology Laboratory	Alaska State Virology Laboratory	Stephanie DeRonde, Elva House, Jacob Zidek, Lisa Smith, Ph.D., Jack Chen, Ph.D.
hCoV-19/USA/AK-PHL10007/2021	EPI_ISL_2987188	4/24/2021 Alaska State Virology Laboratory	Alaska State Virology Laboratory	Stephanie DeRonde, Elva House, Jacob Zidek, Lisa Smith, Ph.D., Jack Chen, Ph.D.
hCoV-19/USA/AK-PHL10008/2021	EPI_ISL_2987189	4/23/2021 Alaska State Virology Laboratory	Alaska State Virology Laboratory	Stephanie DeRonde, Elva House, Jacob Zidek, Lisa Smith, Ph.D., Jack Chen, Ph.D.
hCoV-19/USA/AK-PHL10009/2021	EPI_ISL_2987190	4/26/2021 Alaska State Virology Laboratory	Alaska State Virology Laboratory	Stephanie DeRonde, Elva House, Jacob Zidek, Lisa Smith, Ph.D., Jack Chen, Ph.D.
hCoV-19/USA/AK-PHL10010/2021	EPI_ISL_2987191	4/26/2021 Alaska State Virology Laboratory	Alaska State Virology Laboratory	Stephanie DeRonde, Elva House, Jacob Zidek, Lisa Smith, Ph.D., Jack Chen, Ph.D.
hCoV-19/USA/AK-PHL10011/2021	EPI_ISL_2987192	4/27/2021 Alaska State Virology Laboratory	Alaska State Virology Laboratory	Stephanie DeRonde, Elva House, Jacob Zidek, Lisa Smith, Ph.D., Jack Chen, Ph.D.
hCoV-19/USA/AK-CDC-LC0091083/2021	EPI_ISL_3017837	6/15/2021 Laboratory Corporation of America	Centers for Disease Control and Prevention Division of Viral Diseases, Pathogen Discovery	Dakota Howard, Dhwani Batra, Peter W. Cook, Kara Moser, Adrian Paskey, Jason Caravas, Benjamin Rambo-Martin, Shatavia Morrison, Christopher Gulvick, Scott Sammons, Yvette Unoarumhi, Darlene Wagner, Matthew Schmerer, Minoo Agarwal, Eyad Almasri, Debbie Boles, Ayla Burns, Nuthawin Charoensri, Oren Cohen, Susan Countryman, Mary Ann Cristobal, Bobbi Croy, Suzanne Dale, Hrushikesh Deshmukh, Amanda Douglas, Vincent Drouillon, Marcia Eisenberg, Howard Engler, Rama Ghatti, Prashant Gupta, Susan Hicks, Jake Humphrey, Lax Iyer, Lisa Pfefferle, Manoj Jain, Matthew Robinson, Mohan Kolli, Brian Krueger, Tim Kuphal, Stanley Letovsky, Michael Levandoski, Craig Lukasik, Jonathan Meltzer, Brian Norvell, Mindy Nye, Scott Parker, Christos Petropoulos, John Pruitt, Steven Ragan, Scott Ryan, Mike Sapeta, Jana Schroth, Suresh Babu Selvaraju, Goran Stevovic, Amanda Suchanek, Andrea Throop, Lyndon Tilson, Thomas Urban, Joe Voshell, Kimberly Wagner, Jonathan Williams, Mary Williamson, Qian Zeng, Tricia Zwiefelhofer, Clinton R. Paden, Duncan MacCannell
hCoV-19/USA/AK-PHL10253/2021	EPI_ISL_3067572	4/24/2021 Alaska State Virology Laboratory	Alaska State Virology Laboratory	Stephanie DeRonde, Elva House, Jacob Zidek, Lisa Smith, Ph.D., Jack Chen, Ph.D.
hCoV-19/USA/AK-PHL10129/2021	EPI_ISL_3118591	4/26/2021 Alaska State Virology Laboratory	Alaska State Virology Laboratory	Stephanie DeRonde, Elva House, Jacob Zidek, Lisa Smith, Ph.D., Jack Chen, Ph.D.
hCoV-19/USA/AK-PHL10130/2021	EPI_ISL_3118592	4/26/2021 Alaska State Virology Laboratory	Alaska State Virology Laboratory	Stephanie DeRonde, Elva House, Jacob Zidek, Lisa Smith, Ph.D., Jack Chen, Ph.D.
hCoV-19/USA/AK-PHL10131/2021	EPI_ISL_3118593	4/26/2021 Alaska State Virology Laboratory	Alaska State Virology Laboratory	Stephanie DeRonde, Elva House, Jacob Zidek, Lisa Smith, Ph.D., Jack Chen, Ph.D.
hCoV-19/USA/AK-PHL10132/2021	EPI_ISL_3118594	4/26/2021 Alaska State Virology Laboratory	Alaska State Virology Laboratory	Stephanie DeRonde, Elva House, Jacob Zidek, Lisa Smith, Ph.D., Jack Chen, Ph.D.
hCoV-19/USA/AK-PHL10133/2021	EPI_ISL_3118595	4/26/2021 Alaska State Virology Laboratory	Alaska State Virology Laboratory	Stephanie DeRonde, Elva House, Jacob Zidek, Lisa Smith, Ph.D., Jack Chen, Ph.D.
hCoV-19/USA/AK-PHL10134/2021	EPI_ISL_3118596	4/26/2021 Alaska State Virology Laboratory	Alaska State Virology Laboratory	Stephanie DeRonde, Elva House, Jacob Zidek, Lisa Smith, Ph.D., Jack Chen, Ph.D.
hCoV-19/USA/AK-PHL10135/2021	EPI_ISL_3118597	4/26/2021 Alaska State Virology Laboratory	Alaska State Virology Laboratory	Stephanie DeRonde, Elva House, Jacob Zidek, Lisa Smith, Ph.D., Jack Chen, Ph.D.
hCoV-19/USA/AK-PHL10136/2021	EPI_ISL_3118598	4/26/2021 Alaska State Virology Laboratory	Alaska State Virology Laboratory	Stephanie DeRonde, Elva House, Jacob Zidek, Lisa Smith, Ph.D., Jack Chen, Ph.D.
hCoV-19/USA/AK-PHL10137/2021	EPI_ISL_3118599	4/26/2021 Alaska State Virology Laboratory	Alaska State Virology Laboratory	Stephanie DeRonde, Elva House, Jacob Zidek, Lisa Smith, Ph.D., Jack Chen, Ph.D.
hCoV-19/USA/AK-PHL10138/2021	EPI_ISL_3118600	4/26/2021 Alaska State Virology Laboratory	Alaska State Virology Laboratory	Stephanie DeRonde, Elva House, Jacob Zidek, Lisa Smith, Ph.D., Jack Chen, Ph.D.
hCoV-19/USA/AK-PHL10139/2021	EPI_ISL_3118601	4/21/2021 Alaska State Virology Laboratory	Alaska State Virology Laboratory	Stephanie DeRonde, Elva House, Jacob Zidek, Lisa Smith, Ph.D., Jack Chen, Ph.D.
hCoV-19/USA/AK-PHL10140/2021	EPI_ISL_3118602	4/26/2021 Alaska State Virology Laboratory	Alaska State Virology Laboratory	Stephanie DeRonde, Elva House, Jacob Zidek, Lisa Smith, Ph.D., Jack Chen, Ph.D.
hCoV-19/USA/AK-PHL10141/2021	EPI_ISL_3118603	4/26/2021 Alaska State Virology Laboratory	Alaska State Virology Laboratory	Stephanie DeRonde, Elva House, Jacob Zidek, Lisa Smith, Ph.D., Jack Chen, Ph.D.

hCoV-19/USA/AK-PHL10142/2021	EPI_ISL_3118604	4/26/2021	Alaska State Virology Laboratory	Alaska State Virology Laboratory	Stephanie DeRonde, Elva House, Jacob Zidek, Lisa Smith, Ph.D., Jack Chen, Ph.D.
hCoV-19/USA/AK-PHL10143/2021	EPI_ISL_3118605	4/26/2021	Alaska State Virology Laboratory	Alaska State Virology Laboratory	Stephanie DeRonde, Elva House, Jacob Zidek, Lisa Smith, Ph.D., Jack Chen, Ph.D.
hCoV-19/USA/AK-PHL10144/2021	EPI_ISL_3118606	4/26/2021	Alaska State Virology Laboratory	Alaska State Virology Laboratory	Stephanie DeRonde, Elva House, Jacob Zidek, Lisa Smith, Ph.D., Jack Chen, Ph.D.
hCoV-19/USA/AK-PHL10145/2021	EPI_ISL_3118607	4/26/2021	Alaska State Virology Laboratory	Alaska State Virology Laboratory	Stephanie DeRonde, Elva House, Jacob Zidek, Lisa Smith, Ph.D., Jack Chen, Ph.D.
hCoV-19/USA/AK-PHL10146/2021	EPI_ISL_3118608	4/26/2021	Alaska State Virology Laboratory	Alaska State Virology Laboratory	Stephanie DeRonde, Elva House, Jacob Zidek, Lisa Smith, Ph.D., Jack Chen, Ph.D.
hCoV-19/USA/AK-PHL10147/2021	EPI_ISL_3118609	4/26/2021	Alaska State Virology Laboratory	Alaska State Virology Laboratory	Stephanie DeRonde, Elva House, Jacob Zidek, Lisa Smith, Ph.D., Jack Chen, Ph.D.
hCoV-19/USA/AK-PHL10148/2021	EPI_ISL_3118610	4/25/2021	Alaska State Virology Laboratory	Alaska State Virology Laboratory	Stephanie DeRonde, Elva House, Jacob Zidek, Lisa Smith, Ph.D., Jack Chen, Ph.D.
hCoV-19/USA/AK-PHL10149/2021	EPI_ISL_3118611	4/26/2021	Alaska State Virology Laboratory	Alaska State Virology Laboratory	Stephanie DeRonde, Elva House, Jacob Zidek, Lisa Smith, Ph.D., Jack Chen, Ph.D.
hCoV-19/USA/AK-PHL10150/2021	EPI_ISL_3118612	4/26/2021	Alaska State Virology Laboratory	Alaska State Virology Laboratory	Stephanie DeRonde, Elva House, Jacob Zidek, Lisa Smith, Ph.D., Jack Chen, Ph.D.
hCoV-19/USA/AK-PHL9927/2021	EPI_ISL_3118614	6/23/2021	Alaska State Virology Laboratory	Alaska State Virology Laboratory	Stephanie DeRonde, Elva House, Jacob Zidek, Lisa Smith, Ph.D., Jack Chen, Ph.D.
hCoV-19/USA/AK-PHL9937/2021	EPI_ISL_3118615	6/21/2021	Alaska State Virology Laboratory	Alaska State Virology Laboratory	Stephanie DeRonde, Elva House, Jacob Zidek, Lisa Smith, Ph.D., Jack Chen, Ph.D.
hCoV-19/USA/AK-PHL9944/2021	EPI_ISL_3118616	6/25/2021	Alaska State Virology Laboratory	Alaska State Virology Laboratory	Stephanie DeRonde, Elva House, Jacob Zidek, Lisa Smith, Ph.D., Jack Chen, Ph.D.
hCoV-19/USA/AK-PHL9947/2021	EPI_ISL_3118617	6/25/2021	Alaska State Virology Laboratory	Alaska State Virology Laboratory	Stephanie DeRonde, Elva House, Jacob Zidek, Lisa Smith, Ph.D., Jack Chen, Ph.D.
hCoV-19/USA/AK-PHL9923/2021	EPI_ISL_3118619	6/23/2021	Alaska State Virology Laboratory	Alaska State Virology Laboratory	Stephanie DeRonde, Elva House, Jacob Zidek, Lisa Smith, Ph.D., Jack Chen, Ph.D.
hCoV-19/USA/AK-PHL9924/2021	EPI_ISL_3118620	6/22/2021	Alaska State Virology Laboratory	Alaska State Virology Laboratory	Stephanie DeRonde, Elva House, Jacob Zidek, Lisa Smith, Ph.D., Jack Chen, Ph.D.
hCoV-19/USA/AK-PHL9925/2021	EPI_ISL_3118621	6/22/2021	Alaska State Virology Laboratory	Alaska State Virology Laboratory	Stephanie DeRonde, Elva House, Jacob Zidek, Lisa Smith, Ph.D., Jack Chen, Ph.D.
hCoV-19/USA/AK-PHL9928/2021	EPI_ISL_3118622	6/25/2021	Alaska State Virology Laboratory	Alaska State Virology Laboratory	Stephanie DeRonde, Elva House, Jacob Zidek, Lisa Smith, Ph.D., Jack Chen, Ph.D.
hCoV-19/USA/AK-PHL9938/2021	EPI_ISL_3118623	6/22/2021	Alaska State Virology Laboratory	Alaska State Virology Laboratory	Stephanie DeRonde, Elva House, Jacob Zidek, Lisa Smith, Ph.D., Jack Chen, Ph.D.
hCoV-19/USA/AK-PHL9940/2021	EPI_ISL_3118624	6/22/2021	Alaska State Virology Laboratory	Alaska State Virology Laboratory	Stephanie DeRonde, Elva House, Jacob Zidek, Lisa Smith, Ph.D., Jack Chen, Ph.D.
hCoV-19/USA/AK-PHL9941/2021	EPI_ISL_3118625	6/22/2021	Alaska State Virology Laboratory	Alaska State Virology Laboratory	Stephanie DeRonde, Elva House, Jacob Zidek, Lisa Smith, Ph.D., Jack Chen, Ph.D.
hCoV-19/USA/AK-PHL9942/2021	EPI_ISL_3118626	6/22/2021	Alaska State Virology Laboratory	Alaska State Virology Laboratory	Stephanie DeRonde, Elva House, Jacob Zidek, Lisa Smith, Ph.D., Jack Chen, Ph.D.
hCoV-19/USA/AK-PHL9965/2021	EPI_ISL_3118627	4/27/2021	Alaska State Virology Laboratory	Alaska State Virology Laboratory	Stephanie DeRonde, Elva House, Jacob Zidek, Lisa Smith, Ph.D., Jack Chen, Ph.D.
hCoV-19/USA/AK-PHL9966/2021	EPI_ISL_3118628	4/27/2021	Alaska State Virology Laboratory	Alaska State Virology Laboratory	Stephanie DeRonde, Elva House, Jacob Zidek, Lisa Smith, Ph.D., Jack Chen, Ph.D.
hCoV-19/USA/AK-PHL9972/2021	EPI_ISL_3118637	6/17/2021	Alaska State Virology Laboratory	Alaska State Virology Laboratory	Stephanie DeRonde, Elva House, Jacob Zidek, Lisa Smith, Ph.D., Jack Chen, Ph.D.
hCoV-19/USA/AK-PHL10353/2021	EPI_ISL_3159501	6/14/2021	Alaska State Virology Laboratory	Alaska State Virology Laboratory	Stephanie DeRonde, Elva House, Jacob Zidek, Lisa Smith, Ph.D., Jack Chen, Ph.D.
hCoV-19/USA/CA-SEARCH-32732/2021	EPI_ISL_3215644	5/5/2021	EXCITE Lab	Andersen lab at Scripps Research	David Pride, Sharon Reed, Chip Schooley, Angela Scioscia, Natasha Martin Cheryl Anderson, Sawyer Farmer, Abigail Schnapper, Helena Tubb, Tommy Valles + SEARCH
hCoV-19/USA/AK-CDC-2-3845923/2021	EPI_ISL_3353731	1/15/2021	AK State Public Health Lab, State Health Department	Centers for Disease Control and Prevention Division of Viral Diseases, Pathogen Discovery	Mili Sheth,Sarah Nobles,Jasmine Padilla,Alex Burgin,Meghan Bentz,Mark Burroughs,Peter Cook,Clinton Paden,Dhwani Batra,Krista Queen,Kristen Knipe,Dakota Howard,Yvette Unoarumhi,Matthew Schmerer,Ben Rambo-Martin,Kristine Lacek,Sam Shepard,Dave Wentworth,Vivien Dugan,Suxiang Tong,Justin Lee
hCoV-19/USA/AK-CDC-2-3845944/2021	EPI_ISL_3353734	1/11/2021	AK State Public Health Lab, State Health Department	Centers for Disease Control and Prevention Division of Viral Diseases, Pathogen Discovery	Mili Sheth,Sarah Nobles,Jasmine Padilla,Alex Burgin,Meghan Bentz,Mark Burroughs,Peter Cook,Clinton Paden,Dhwani Batra,Krista Queen,Kristen Knipe,Dakota Howard,Yvette Unoarumhi,Matthew Schmerer,Ben Rambo-Martin,Kristine Lacek,Sam Shepard,Dave Wentworth,Vivien Dugan,Suxiang Tong,Justin Lee
hCoV-19/USA/AK-CDC-2-3925257/2021	EPI_ISL_3354027	2/8/2021	AK State Public Health Lab, State Health Department	Centers for Disease Control and Prevention Division of Viral Diseases, Pathogen Discovery	Mili Sheth,Sarah Nobles,Jasmine Padilla,Alex Burgin,Meghan Bentz,Mark Burroughs,Peter Cook,Clinton Paden,Dhwani Batra,Krista Queen,Kristen Knipe,Dakota Howard,Yvette Unoarumhi,Matthew Schmerer,Ben Rambo-Martin,Kristine Lacek,Sam Shepard,Dave Wentworth,Vivien Dugan,Suxiang Tong,Justin Lee
hCoV-19/USA/AK-CDC-2-3980576/2021	EPI_ISL_3354174	2/22/2021	AK State Public Health Lab, State Health Department	Centers for Disease Control and Prevention Division of Viral Diseases, Pathogen Discovery	Mili Sheth,Sarah Nobles,Jasmine Padilla,Alex Burgin,Meghan Bentz,Mark Burroughs,Peter Cook,Clinton Paden,Dhwani Batra,Krista Queen,Kristen Knipe,Dakota Howard,Yvette Unoarumhi,Matthew Schmerer,Ben Rambo-Martin,Kristine Lacek,Sam Shepard,Dave Wentworth,Vivien Dugan,Suxiang Tong,Justin Lee
hCoV-19/USA/AK-CDC-2-4043802/2021	EPI_ISL_3354314	3/9/2021	AK State Public Health Lab, State Health Department	Centers for Disease Control and Prevention Division of Viral Diseases, Pathogen Discovery	Mili Sheth,Sarah Nobles,Jasmine Padilla,Alex Burgin,Meghan Bentz,Mark Burroughs,Peter Cook,Clinton Paden,Dhwani Batra,Krista Queen,Kristen Knipe,Dakota Howard,Yvette Unoarumhi,Matthew Schmerer,Ben Rambo-Martin,Kristine Lacek,Sam Shepard,Dave Wentworth,Vivien Dugan,Suxiang Tong,Justin Lee

hCoV-19/USA/AK-CDC-2-4242677/2021	EPI_ISL_3354794	4/1/2021	AK State Public Health Lab, State Health Department	Centers for Disease Control and Prevention Division of Viral Diseases, Pathogen Discovery	Mili Sheth,Sarah Nobles,Jasmine Padilla,Alex Burgin,Meghan Bentz,Mark Burroughs,Peter Cook,Clinton Paden,Dhwani Batra,Krista Queen,Kristen Knipe,Dakota Howard,Yvette Unoarumhi,Matthew Schmerer,Ben Rambo-Martin,Kristine Lacek,Sam Shepard,Dave Wentworth,Vivien Dugan,Suxiang Tong,Justin Lee
hCoV-19/USA/AK-CDC-2-4523971/2021	EPI_ISL_3355081	5/18/2021	AK State Public Health Lab, State Health Department	Centers for Disease Control and Prevention Division of Viral Diseases, Pathogen Discovery	Mili Sheth,Sarah Nobles,Jasmine Padilla,Alex Burgin,Meghan Bentz,Mark Burroughs,Peter Cook,Clinton Paden,Dhwani Batra,Krista Queen,Kristen Knipe,Dakota Howard,Yvette Unoarumhi,Matthew Schmerer,Ben Rambo-Martin,Kristine Lacek,Sam Shepard,Dave Wentworth,Vivien Dugan,Suxiang Tong,Justin Lee
hCoV-19/USA/AK-PHL11275/2021	EPI_ISL_3668698	6/18/2021	Alaska State Virology Laboratory	Alaska State Virology Laboratory	Stephanie DeRonde, Elva House, Jacob Zidek, Lisa Smith, Ph.D., Jack Chen, Ph.D.
hCoV-19/USA/AK-PHL12647/2021	EPI_ISL_4298582	6/15/2021	Alaska State Virology Laboratory	Alaska State Virology Laboratory	Stephanie DeRonde, Elva House, Jacob Zidek, Lisa Smith, Ph.D., Jack Chen, Ph.D.
hCoV-19/USA/AK-CDC-STM-000038524/2021	EPI_ISL_4346709	3/18/2021	Helix/Illumina	Centers for Disease Control and Prevention Division of Viral Diseases, Pathogen Discovery	Dakota Howard,Dhwani Batra,Peter Cook,Kara Moser,Adrian Paskey,Jason Caravas,Benjamin Rambo-Martin,Shatavia Morrison,Christopher Gulvick,Scott Sammons,Yvette Unoarumhi,Darlene Wagner,Matthew Schmerer,Eileen De Feo,Jan Antico,Chrstine Tran,Matthew Tolentino,Shannon Wickline,Kim Gietzen,Brad Sickler,Jingtao Liu,Eric Allen,Phil Febbo,Nicole Washington,Simon White,Geraint Levan,Kelly Barrett,Elizabeth Cirulli,Alexandre Bolze,Ary Ascencio,Charlotte Rivera-Garcia,Ryan Cho,Jason Nguyen,Sherry Wang,Jimmy Ramirez,Tyler Cassens,Efren Sandoval,Magnus Isaksson,William Lee,David Becker,Marc Laurent,James Lu,Clinton Paden,Duncan MacCannell,Kristine Lacek
hCoV-19/USA/AK-CDC-ASC210065587/2021	EPI_ISL_4361363	5/19/2021	Aegis Sciences Corporation	Centers for Disease Control and Prevention Division of Viral Diseases, Pathogen Discovery	Dakota Howard,Dhwani Batra,Peter Cook,Jason Caravas,Benjamin Rambo-Martin,Scott Sammons,Yvette Unoarumhi,Matthew Schmerer,Kristine Lacek,Tymeckia Kendall,Victoria Caban Figueroa,Shatavia Morrison,Christopher Gulvick,Erisa Sula,Cyndi Clark,Patrick Campbell,Rob Case,Vikramsinha Ghorpade,Holly Houdeshell,Ola Kvalvaag,Dillon Nall,Ethan Sanders,Alec Vest,Shaun Westlund,Matthew Hardison,Clinton Paden,Duncan MacCannell
hCoV-19/USA/AK-CDC-QDX25930999/2021	EPI_ISL_4369913	6/16/2021	Quest Diagnostics Incorporated	Centers for Disease Control and Prevention Division of Viral Diseases, Pathogen Discovery	Dakota Howard,Dhwani Batra,Peter Cook,Jason Caravas,Benjamin Rambo-Martin,Scott Sammons,Yvette Unoarumhi,Matthew Schmerer,Kristine Lacek,Tymeckia Kendall,Victoria Caban Figueroa,Shatavia Morrison,Christopher Gulvick,Erisa Sula,S. Rosenthal,A. Gerasimova,R. Kagan,B. Anderson,M. Hua,Y. Liu,L. Bernstein,K. Livingston,A. Perez,I. Shlyakhter,R. Rolando,R. Owen,P. Tanpaiboon,F. Lacbawan,Clinton Paden,Duncan MacCannell
hCoV-19/USA/AK-CDC-LC0069159/2021	EPI_ISL_4373364	6/1/2021	Laboratory Corporation of America	Centers for Disease Control and Prevention Division of Viral Diseases, Pathogen Discovery	Dakota Howard,Dhwani Batra,Peter Cook,Jason Caravas,Benjamin Rambo-Martin,Scott Sammons,Yvette Unoarumhi,Matthew Schmerer,Kristine Lacek,Tymeckia Kendall,Victoria Caban Figueroa,Shatavia Morrison,Christopher Gulvick,Minoo Agarwal,Eyad Almasri,Debbie Boles,Ayla Burns,Nuthawin Charoensri,Oren Cohen,Susan Countryman,Mary Cristobal,Bobbi Croy,Suzanne Dale,Hrushikesh Deshmukh,Amanda Douglas,Vincent Drouillon,Marcia Eisenberg,Howard Engler,Rama Ghatti,Prashant Gupta,Susan Hicks,Jake Humphrey,Lax Iyer,Lisa Pfefferle,Manoj Jain,Matthew Robinson,Mohan Kolli,Brian Krueger,Tim Kuphal,Stanley Letovsky,Michael Levandoski,Craig Lukasik,Jonathan Meltzer,Brian Norvell,Mindy Nye,Scott Parker,Christos Petropoulos,John Pruitt,Steven Ragan,Scott Ryan,Mike Sapeta,Jana Schroth,Suresh Selvaraju,Goran Stevovic,Amanda Suchanek,Andrea Throop,Lyndon Tilson,Thomas Urban,Joe Voshell,Kimberly Wagner,Jonathan Williams,Mary Williamson,Qian Zeng,Tricia Zwiefelhofer,Clinton Paden,Duncan MacCannell

hCoV-19/USA/AK-CDC-LC0073596/2021	EPI_ISL_4373811	6/14/2021	Laboratory Corporation of America	Centers for Disease Control and Prevention Division of Viral Diseases, Pathogen Discovery	Dakota Howard,Dhwani Batra,Peter Cook,Jason Caravas,Benjamin Rambo-Martin,Scott Sammons,Yvette Unoarumhi,Matthew Schmerer,Kristine Lacek,Tymeckia Kendall,Victoria Caban Figueroa,Shatavia Morrison,Christopher Gulvick,Minoo Agarwal,Eyad Almasri,Debbie Boles,Ayla Burns,Nuthawin Charoensri,Oren Cohen,Susan Countryman,Mary Cristobal,Bobbi Croy,Suzanne Dale,Hrushikesh Deshmukh,Amanda Douglas,Vincent Drouillon,Marcia Eisenberg,Howard Engler,Rama Ghatti,Prashant Gupta,Susan Hicks,Jake Humphrey,Lax Iyer,Lisa Pfefferle,Manoj Jain,Matthew Robinson,Mohan Kolli,Brian Krueger,Tim Kuphal,Stanley Letovsky,Michael Levandoski,Craig Lukasik,Jonathan Meltzer,Brian Norvell,Mindy Nye,Scott Parker,Christos Petropoulos,John Pruitt,Steven Ragan,Scott Ryan,Mike Sapeta,Jana Schroth,Suresh Selvaraju,Goran Stevovic,Amanda Suchanek,Andrea Throop,Lyndon Tilson,Thomas Urban,Joe Voshell,Kimberly Wagner,Jonathan Williams,Mary Williamson,Qian Zeng,Tricia Zwiefelhofer,Clinton Paden,Duncan MacCannell
hCoV-19/USA/AK-CDC-LC0071119/2021	EPI_ISL_4376278	6/7/2021	Laboratory Corporation of America	Centers for Disease Control and Prevention Division of Viral Diseases, Pathogen Discovery	Dakota Howard,Dhwani Batra,Peter Cook,Jason Caravas,Benjamin Rambo-Martin,Scott Sammons,Yvette Unoarumhi,Matthew Schmerer,Kristine Lacek,Tymeckia Kendall,Victoria Caban Figueroa,Shatavia Morrison,Christopher Gulvick,Minoo Agarwal,Eyad Almasri,Debbie Boles,Ayla Burns,Nuthawin Charoensri,Oren Cohen,Susan Countryman,Mary Cristobal,Bobbi Croy,Suzanne Dale,Hrushikesh Deshmukh,Amanda Douglas,Vincent Drouillon,Marcia Eisenberg,Howard Engler,Rama Ghatti,Prashant Gupta,Susan Hicks,Jake Humphrey,Lax Iyer,Lisa Pfefferle,Manoj Jain,Matthew Robinson,Mohan Kolli,Brian Krueger,Tim Kuphal,Stanley Letovsky,Michael Levandoski,Craig Lukasik,Jonathan Meltzer,Brian Norvell,Mindy Nye,Scott Parker,Christos Petropoulos,John Pruitt,Steven Ragan,Scott Ryan,Mike Sapeta,Jana Schroth,Suresh Selvaraju,Goran Stevovic,Amanda Suchanek,Andrea Throop,Lyndon Tilson,Thomas Urban,Joe Voshell,Kimberly Wagner,Jonathan Williams,Mary Williamson,Qian Zeng,Tricia Zwiefelhofer,Clinton Paden,Duncan MacCannell
hCoV-19/USA/AK-CDC-IBX178701677804/2021	EPI_ISL_4388562	4/24/2021	Infinity Biologix	Centers for Disease Control and Prevention Division of Viral Diseases, Pathogen Discovery	Dakota Howard,Dhwani Batra,Peter Cook,Jason Caravas,Benjamin Rambo-Martin,Scott Sammons,Yvette Unoarumhi,Matthew Schmerer,Kristine Lacek,Tymeckia Kendall,Victoria Caban Figueroa,Shatavia Morrison,Christopher Gulvick,Erisa Sula,Christian Bixby,Yihe Wang,Jonathan Schultz,Chirayu Goswami,Russ Hager,Robin Grimwood,Clinton Paden,Duncan MacCannell
hCoV-19/USA/AK-ASU19578/2021	EPI_ISL_5644516	1/24/2021	Arizona State University	Arizona State University	Ajeet Bains, LaRinda A. Holland, Matthew F. Smith, Regan A. Sullins, Nicholas J. Mellor, Nathaniel Johnson, Joshua LaBaer, Vel Murugan, Efrem S. Lim
